# CD28 Costimulation Regulates Genome-Wide Effects on Alternative Splicing

**DOI:** 10.1371/journal.pone.0040032

**Published:** 2012-06-29

**Authors:** Manish J. Butte, Sun Jung Lee, Jonathan Jesneck, Mary E. Keir, W. Nicholas Haining, Arlene H. Sharpe

**Affiliations:** 1 Department of Microbiology and Immunobiology, Harvard Medical School, Boston, Massachusetts, United States of America; 2 Department of Pediatric Oncology, Dana Farber Cancer Institute, Boston, Massachusetts, United States of America and Division of Pediatric Hematology/Oncology, Children’s Hospital, Boston, Massachusetts, United States of America; 3 Department of Pathology, Brigham and Women’s Hospital, Boston, Massachusetts, United States of America; La Jolla Institute for Allergy and Immunology, United States of America

## Abstract

CD28 is the major costimulatory receptor required for activation of naïve T cells, yet CD28 costimulation affects the expression level of surprisingly few genes over those altered by TCR stimulation alone. Alternate splicing of genes adds diversity to the proteome and contributes to tissue-specific regulation of genes. Here we demonstrate that CD28 costimulation leads to major changes in alternative splicing during activation of naïve T cells, beyond the effects of TCR alone. CD28 costimulation affected many more genes through modulation of alternate splicing than by modulation of transcription. Different families of biological processes are over-represented among genes alternatively spliced in response to CD28 costimulation compared to those genes whose transcription is altered, suggesting that alternative splicing regulates distinct biological effects. Moreover, genes dependent upon hnRNPLL, a global regulator of splicing in activated T cells, were enriched in T cells activated through TCR plus CD28 as compared to TCR alone. We show that hnRNPLL expression is dependent on CD28 signaling, providing a mechanism by which CD28 can regulate splicing in T cells and insight into how hnRNPLL can influence signal-induced alternative splicing in T cells. The effects of CD28 on alternative splicing provide a newly appreciated means by which CD28 can regulate T cell responses.

## Introduction

Effective activation of naïve T cells requires both T cell receptor (TCR) stimulation and CD28 costimulation. Signals through CD28 promote expression of growth and survival factors, and glucose metabolism, enabling T cell expansion and differentiation. Although CD28 is the major costimulatory receptor for activation of naïve T cells, previous studies have found few CD28-specific changes in gene transcription upon TCR and CD28 co-engagement [1], [2]. Thus, CD28 costimulation is thought to mainly amplify TCR signals rather than have specific effects on the cell state. Recent studies have revealed that alternative splicing (AS), as well as gene-level transcription, play important regulatory roles in T cell biology [3]. AS can increase proteome diversity by increasing the number of distinct mRNA transcripts from a single gene locus. Transcript variation can modify protein interaction networks by removing or inserting protein domains, altering subcellular localization, or regulating gene expression in different cell types and cell states. AS can regulate gene expression by eliminating binding sites for translational repression by microRNAs and by targeting mRNAs for nonsense-mediated decay [4]. Although the biologic effects of AS are only beginning to be appreciated, recent studies have revealed roles for AS in regulating stem cell pluripotency and differentiation, as well as neuronal differentiation, diversity and plasticity [5]. AS also regulates genes important for immune cell differentiation and function [6]. These findings led us to hypothesize that CD28 may exert some of its regulatory effects through AS.

To test this hypothesis, we compared genome-wide AS in naïve T cells following stimulation through TCR alone or TCR plus CD28 costimulaton. For our genomic analyses, we used rigorously naïve T cells to circumvent issues that have complicated the interpretation of previous studies, which used human peripheral blood T cells or T cell lines to identify genes responsive to the activation of naïve T cells. Studies with human peripheral T cells have been confounded by the unintentional admixture of previously activated or memory T cells [1], [2], [7], which differ from naïve T cells in their requirements for activation [8]. In addition, studies of human T cells stimulated with PMA or PHA cannot distinguish the effects of TCR versus CD28 signaling [7]. Microarray studies using T cell lines, such as Jurkat cells, may be difficult to extend to primary cells because of aberrant signaling in Jurkat cells [9], [10]. Therefore, use of rigorously naïve T cells enabled analyses of specific effects of TCR and CD28 during initial T cell activation.

Using exon microarrays, we identified CD28-specific changes in transcription and AS across diverse gene families. Remarkably, CD28 costimulation affected many more genes through alternative splicing than by altering transcription level. While the expression levels of only 140 transcripts were significantly altered in a CD28-specific fashion, the splicing of 1,047 transcripts was altered by TCR plus CD28 activation as compared to TCR activation alone. The marked influence of CD28 costimulation on splicing in T cells led us to investigate whether CD28 signaling promotes expression of factors that regulate splicing. We focused on the global splicing regulatory factor hnRNPLL because recent work has identified hnRNPLL as a regulator of splicing in activated T cells. We determined that the expression of hnRNPLL is CD28 dependent, providing a mechanism by which CD28 can control splicing in T cells and new insight into the function of hnRNPLL as a mediator of signal-induced alternative splicing in T cells [11].

## Results

### Transcripts Affected by TCR and TCR/CD28 Activation

**Figure 1:**
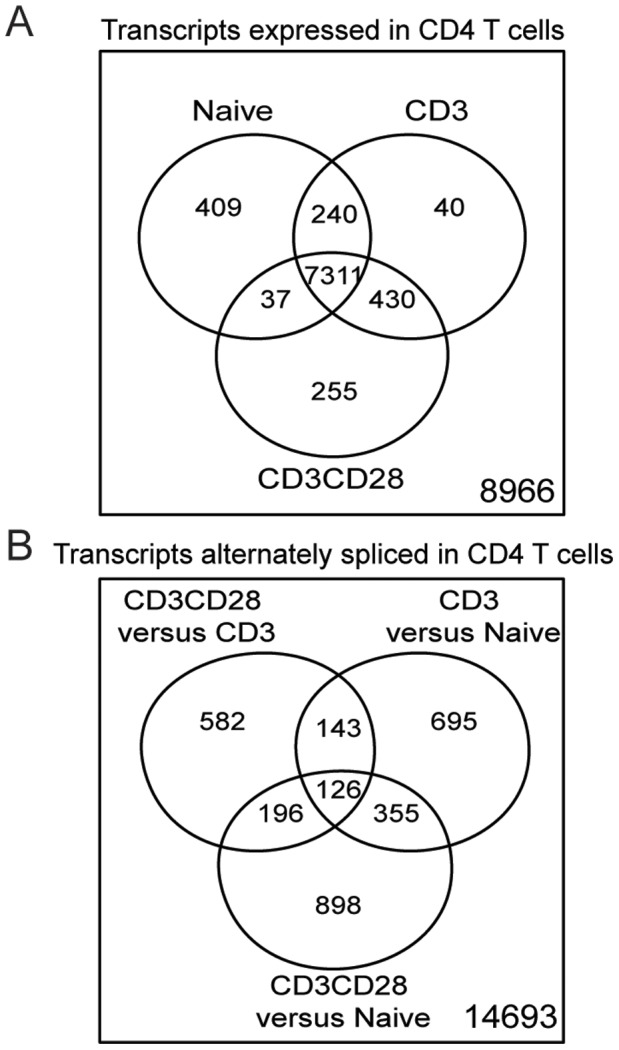
Genes are both differentially expressed and alternately spliced following TCR and CD28 activation. (A) Venn diagram showing the number of transcripts expressed in naïve, TCR-activated, and TCR/CD28-activated T cells. The unique transcripts for each group are provided in Table S1. These unique transcripts are enriched for Gene Ontology biological processes, provided in Table S2. (B) Venn diagram showing the number of transcripts differentially spliced in naïve, TCR-activated, and TCR/CD28-activated T cells. The unique transcripts (non-overlapping parts of the Venn diagram) are listed in Table S4.

To model T cell activation, we cultured rigorously naïve CD4 T cells with beads coated with anti-CD3 plus either anti-CD28 (TCR/CD28) or a control Ig (TCR) for 24 hours. We then isolated and reverse-transcribed RNA, and hybridized the resulting DNA to exon microarrays. Analysis of transcript abundance showed that 8,966 of the 17,689 transcripts assessed were designated as “present” (i.e., the transcripts were expressed at a detectable level above background). Most (7,311) of these transcripts were shared among the naïve, TCR-activated, and TCR/CD28-activated T cells (Figure 1A). A few unique transcripts were identified that were not seen in the other states: 409 in naïve T cells, 40 in TCR-activated T cells, and 255 in TCR/CD28-activated T cells. The unique transcripts for each condition (naive, TCR-activated, and TCR/CD28-activated) are listed in Table S1. We used the Gene Ontology framework to categorize the biological processes for these unique transcripts and determine whether some biological functions might be over-represented in upregulated transcripts. We found that these unique transcripts were enriched for processes associated with cell cycle and chemotaxis in the TCR/CD28-activated group. All statistically enriched biological processes for these unique transcripts are listed in Table S2.

We next analyzed changes in gene transcription level, using a cutoff of 2-fold differences and measuring significance using empirical Bayesian testing [12]. 1,481 genes showed a greater than 2-fold change in expression level in T cells activated with anti-CD3 alone compared to naïve cells, and 3,806 genes showed transcription-level differences in T cells activated by anti-CD3 plus anti-CD28 compared to naïve cells. We found only 140 genes with greater than 2-fold transcriptional differences in T cells activated through TCR/CD28 as compared to T cells activated through TCR alone, a relatively small number of genes, but consistent with previous work [1], [2]. The transcripts with the largest fold changes (up- and down-regulation) are shown with independent replicates (Figure S1). Volcano plots summarize both fold-change and t-test criteria and highlight some of the transcripts with significant differences in expression (Figure S2). All transcripts with expression-level changes greater than 2-fold are provided in Table S3. As expected, these differences included characteristic changes of naïve to effector differentiation including up-regulation of Tbet (*Tbx21*), CD25 (*Il2ra*), and down-regulation of IL-7R alpha chain (CD127, *Il7r*).

We then investigated changes in splicing, and measured significance using two statistical tests for splicing (MADS and MIDAS) [13]. We considered only differences in splicing that had statistically significant *p* values in both statistical tests. Using this criterion, we found 1,319 genes with alterations in splicing when T cells were activated through TCR alone compared to naïve T cells, and 1,575 transcripts with alterations in splicing when naïve T cells were activated through TCR/CD28 compared to naïve T cells. In contrast to its effects on gene-level transcription of only 140 genes, TCR/CD28 activation altered the splicing of 1,047 transcripts, as compared to T cells activated through TCR alone (Figure 1B). The unique transcripts showing AS in each condition are detailed in Table S4. Thus, activation of naïve T cells by TCR plus CD28 exerts effects on nearly 8 times as many genes by modulating splicing than by modulating transcription, when compared to TCR activation alone.

### Distinct Biological Processes are Affected by Transcriptional Regulation versus Alternate Splicing in Response to CD28 Costimulation

**Figure 2:**
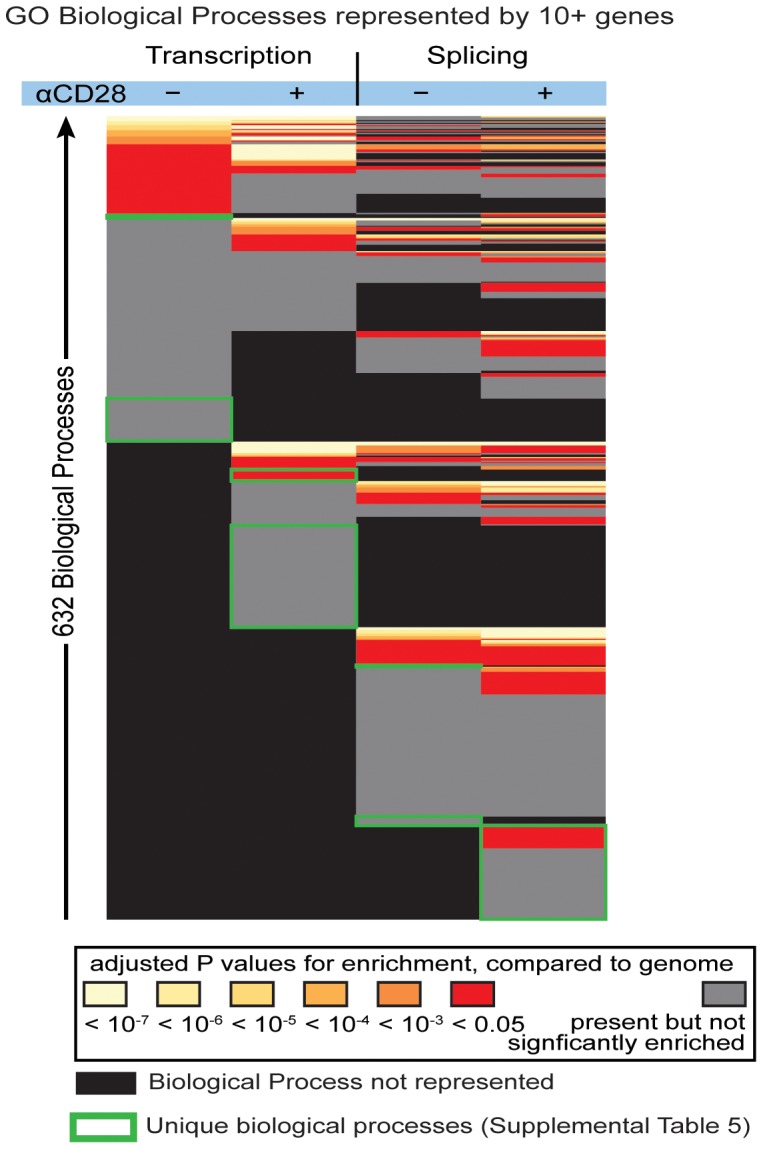
Functional families for genes whose transcription or splicing is affected by TCR alone or TCR/CD28. Biological processes, as defined by Gene Ontology, were counted if at least 10 genes comprising that process were present and were clustered in four groups: transcription or splicing in T cells activated through TCR alone or through TCR and CD28. The biological processes that were unique to each of the four conditions are indicated by the green boxes, and are listed in Tables S5 and S6. If biological processes were statistically enriched compared to the whole genome, color indicates adjusted *p* values of enrichment <0.05 (shades of red), *p*>0.05 (grey).

Regulation of distinct biological processes can be detected by the coordinate regulation of sets of genes related to each process. We asked whether the genes altered by AS or by transcriptional level were the same or different. We compared the Gene Ontology annotations for genes with altered transcription levels under conditions of TCR versus TCR/CD28 activation (as compared to naïve cells), or alternately spliced genes under conditions of TCR versus TCR/CD28 activation (as compared to naïve cells) (Figure 2). We found 632 biological processes of the Gene Ontology framework represented by the genes altered by splicing, transcription, and activation method (TCR or TCR/CD28). We categorized these biological processes into the following four groups of genes (each compared to naive T cells): those altered in splicing with TCR activation, altered in splicing with TCR/CD28 activation, altered in transcription level with TCR activation, and altered in transcription level with TCR/CD28 activation (Figure 2).

Of the 632 biological processes seen across these conditions, some were seen in a subset of conditions, and some were uniquely seen in only one condition; 187 biological processes were unique to transcription, 230 were unique to AS, and 215 were shared between AS and transcription. For example, the biological process called “regulation of protein amino acid phosphorylation” was seen in all four conditions. The biological processes that were unique to each of the four conditions (boxed in green in Figure 2) are listed in Tables S5 and S6. Unique biological processes related to RNA processing were seen among the genes altered by transcription in TCR/CD28-activated cells as compared to naive cells, and unique biological processes related to RNA splicing were seen among the genes altered by splicing in TCR/CD28-activated cells as compared to naive cells (Table S5). We also examined the biological processes for all the genes that were differentially expressed and those alternately spliced when comparing TCR and TCR/CD28 activation (Table S6). Some of the functionally-related families of genes that are extensively affected by CD28-induced alternative splicing (e.g. regulation of purine metabolism, regulators of mitotic cell cycle) are consistent with the known biological effects of CD28 on T cell expansion. However, other families are novel (e.g., RNA processing, RNA splicing, DNA metabolic process). Thus, we again found enrichment of biological processes related to RNA processing and RNA splicing in the genes differentially spliced between TCR-activated T cells and TCR/CD28-activated T cells. Analysis of these functionally-related families of genes showed that they are densely interconnected based on prior co-expression, physical interaction and co-localization data (Figure S3). Thus the function of these genes may be co-ordinately regulated not only at the level of transcript abundance, but also by alternative splicing. It is interesting, but not immediately apparent why biological processes related to splicing should be the function of some of the genes themselves altered by splicing upon T cell activation. Thus, our studies indicate that CD28 costimulation can influence specific biological processes through alterations of transcription expression level or AS.

### Transcriptional Changes Mediated by CD28 Costimulation

**Figure 3:**
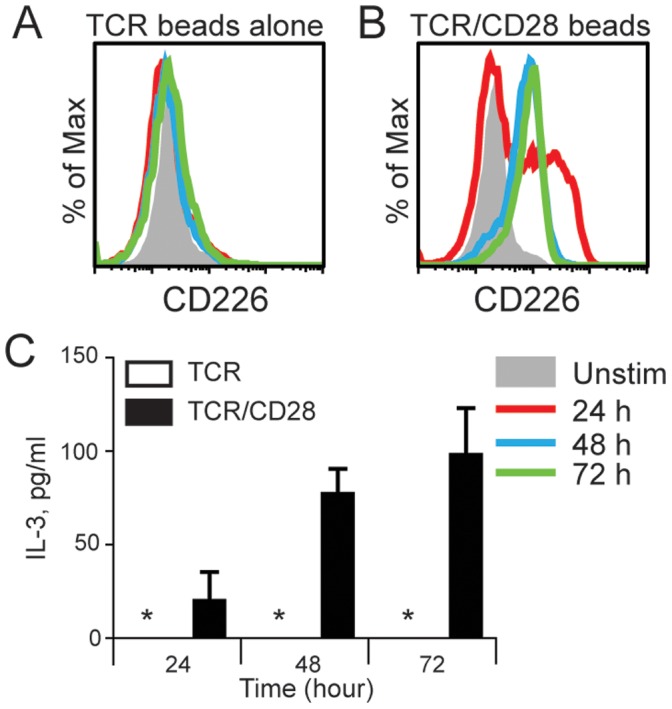
CD28 signaling increases CD226 expression and IL-3 secretion in CD4 T cells. Sorted naïve CD4+CD25- T cells from C57BL/6 wild type mice were stimulated with bead-bound anti-CD3 alone (A) or anti-CD3 plus anti-CD28 (B) for the indicated times. Cells were stained for CD226 surface expression (A, B) and supernatants were used to quantify IL-3 secretion (C). Data were acquired by flow cytometry and analyzed using FlowJo software. Asterisk (*) indicates below detection limit (1.1 pg/mL). These results are representative of at least 3 independent experiments.

To validate transcriptional changes identified by the exon microarrays, we compared the mRNA and protein expression of several genes greatly altered in a CD28-specific fashion. (i.e., genes greatly affected by TCR stimulation plus CD28 costimulation as compared to TCR stimulation alone). CD226, a costimulatory receptor in the CD226/TIGIT pathway, and the cytokine IL-3 were among the genes whose transcription were most upregulated in a CD28-specific fashion. Transcript levels of the CD226 and IL-3 genes were increased by ∼6 fold upon treatment of naïve T cells with anti-CD3 plus anti-CD28 as compared with CD3 alone (Table S3). To investigate the extent to which CD226 and IL-3 protein expression are CD28-dependent, we analyzed expression of CD226 protein by flow cytometry and production of IL-3 by cytokine bead array following activation of naive CD4 T cells over a 72 hour period (Figure 3). When naïve CD4 T cells were stimulated only with anti-CD3, there was negligible expression of CD226 at 24, 48 or 72 hr. In contrast, CD4 T cells stimulated with anti-CD3 plus anti-CD28 showed increased expression of CD226 at 24 hrs, and CD226 expression was sustained at even higher levels at 48 and 72 hours (Figures 3A and 3B). Thus, induction of CD226 expression in CD4 T cells is dependent upon ligation of CD28. Similarly, the production of IL-3 was CD28 dependent. IL-3 was produced only when naïve CD4 T cells were stimulated with anti-CD3 plus CD28 (Figure 3C). When naïve CD4 T cells were stimulated with anti-CD3 alone, IL-3 production was negligible (below the detection limit of our assay). The same results were seen using naive OT-II^+/+^ TCRα^−/−^ T cells or naïve CD4 T cells from C57BL/6 mice.

**Figure 4:**
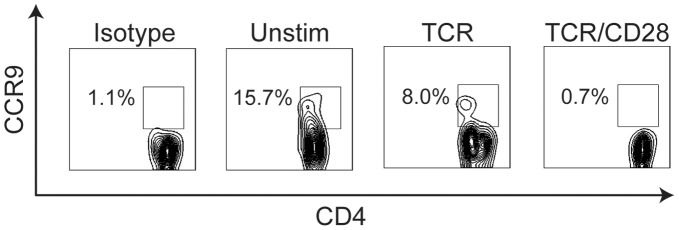
CD28 signaling downregulates CCR9 surface expression on CD4 T cells. Sorted naïve CD4+CD25- T cells from wild type C57BL/6 mice were stimulated with bead-bound anti-CD3 alone or anti-CD3 plus anti-CD28 for 72 h. CCR9 surface expression was examined by flow cytometry. These results are representative of at least 3 independent experiments.

The chemokine receptor CCR9, was identified as a gene whose transcription was downregulated in a CD28-specific fashion. To validate this, we analyzed CCR9 protein expression by flow cytometry following activation of naïve CD4 T cells over a period of 72 hours. Naïve CD4 T cells expressed CCR9 protein. Stimulation of naïve CD4 T cells with anti-CD3 reduced expression of CCR9 protein by ∼50%. When naïve CD4 T cells were stimulated with anti-CD3 plus anti-CD28, CCR9 expression was no longer detected (Figure 4). The greater effects of anti-CD3 plus anti-CD28 stimulation as compared to anti-CD3 alone on CCR9 cell surface expression are consistent with the transcription data (CCR9 mRNA levels decreased ∼9 fold with anti-CD3, and 36-fold with anti-CD3 plus ant-CD28). Taken together, our studies of CD226, IL-3 and CCR9 protein expression validate our exon microarray data. These analyses demonstrate that CD226, IL-3 and CCR9 protein expression are among the relatively small number of genes regulated in a CD28-specific fashion. Together, these results also validate that the exon microarray can successfully identify changes in gene expression.

### CD28 Stimulation Regulates Expression of the Splicing Factor hnRNPLL

**Figure 5:**
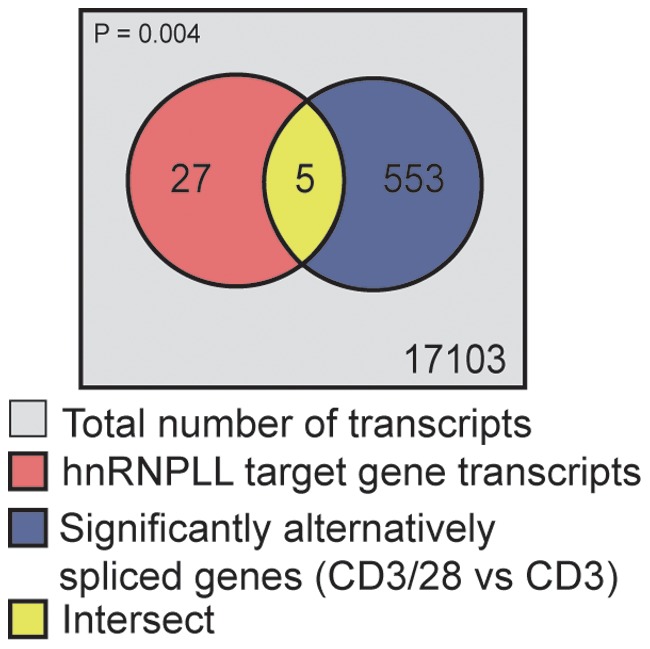
Enrichment of hnRNPLL splicing targets in genes alternatively spliced in T cells activated with TCR/CD28. Representation of the overlap of hnRNPLL target gene transcripts from Oberdoerffer et al. [14] with the genes displaying significant alternative splicing in anti-CD3/CD28 versus anti-CD3 stimulation conditions. Statistical significance of the overlap was calculated using the hypergeometric distribution.

The dramatic effects of CD28 costimulation on AS led us to hypothesize that CD28 costimulation might regulate global splicing factors in T cells. Because recent data have shown the splicing factor hnRNPLL to be critical for alternative splicing following T cell activation and identified a set of genes that require hnRNPLL to undergo alternative splicing [14], we investigated whether hnRNPLL was involved in CD28-dependent AS using two complementary approaches. First, we reasoned that if hnRNPLL were involved in specifying the set of genes that underwent alternative splicing with TCR/CD28 stimulation versus TCR stimulation alone, then genes that are known to depend on hnRNPLL for splicing should be enriched among those alternatively spliced in the TCR/CD28 condition. We, therefore, used the set of 33 functionally validated hnRNPLL “splicing targets” from Oberdoerffer et al. (Table 1) as a query set of genes to ask whether hnRNPLL might dictate part of the alternative splicing seen in TCR/CD28 versus TCR conditions. We calculated the overlap of these validated hnRNPLL target genes with the genes displaying significant alternative splicing in CD3/CD28 versus CD3 conditions. Statistical significance of the overlap was calculated using the hypergeometric distribution. We found that the 5 of 33 hnRNPLL target genes were alternatively spliced following TCR/CD28 stimulation compared to CD3 stimulation alone, a number higher than expected by chance alone (*p* = 0.004) (Figure 5). This result suggested that the difference in alternative splicing in the presence of CD28 costimulation may be due, in part, to the effects of hnRNPLL.

**Table 1:** Mouse orthologs of genes depending on hnRNPLL for alternative splicing [14].

Gene Symbol	Gene Name
**Ptprc**	**protein tyrosine phosphatase, receptor type, C**
Wac	WW domain containing adaptor with coiled-coil
**Cd44**	**CD44 antigen**
Rad54b	RAD54 homolog B (S. cerevisiae)
Stat5a	signal transducer and activator of transcription 5A
Scd2	stearoyl-Coenzyme A desaturase 2
Snrpa1	small nuclear ribonucleoprotein polypeptide A’
**Cflar**	**CASP8 and FADD-like apoptosis regulator**
Txndc5	thioredoxin domain containing 5
Fasn	fatty acid synthase
Parg	poly (ADP-ribose) glycohydrolase
Eif3d	eukaryotic translation initiation factor 3, subunit D
Pxdn	peroxidasin homolog (Drosophila)
Eif4g3	eukaryotic translation initiation factor 4 gamma, 3
Hspa14	heat shock protein 14
Gstm2	glutathione S-transferase, mu 2
Dusp2	dual specificity phosphatase 2
**Traf 1**	**TNF receptor-associated factor 1**
Ywhaz	tyrosine 3-monooxygenase/tryptophan 5-monooxygenase activation protein, zeta polypeptide
Chordc1	cysteine and histidine-rich domain (CHORD)-containing, zinc-binding protein 1
Rbm14	RNA binding motif protein 14
Tuba1b	tubulin, alpha 1B
Sirt5	sirtuin 5 (silent mating type information regulation 2 homolog) 5 (S. cerevisiae)
Tcfe3	transcription factor E3
**Nap 1l1**	**nucleosome assembly protein 1-like 1**
Alg11	asparagine-linked glycosylation 11 homolog (yeast, alpha-1,2-mannosyltransferase)
Ppan	peter pan homolog (Drosophila)
**Myst4**	**MYST histone acetyltransferase monocytic leukemia 4**
Cd96	CD96 antigen
Runx3	runt related transcription factor 3
Usp20	ubiquitin specific peptidase 20
Myg1	melanocyte proliferating gene 1
Tnfsf8	tumor necrosis factor (ligand) superfamily, member 8

Emboldened lines indicate overlap with genes significantly alternatively spliced in CD3/CD28 vs. CD3 comparison.

**Figure 6:**
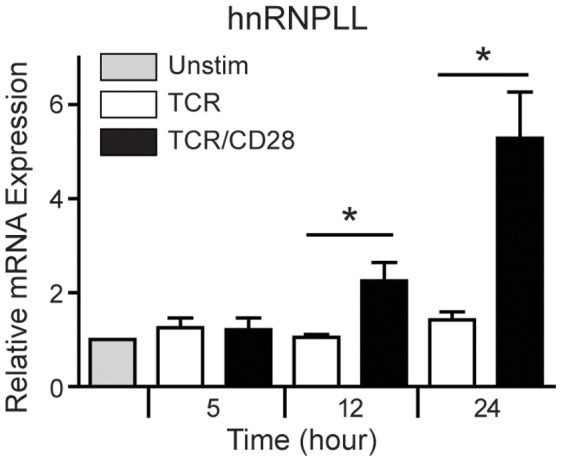
CD28 costimulation upregulates hnRNPLL mRNA expression in CD4 T cells. Sorted naïve CD4+CD25- T cells from wild type C57BL/6 mice were stimulated with bead-bound anti-CD3 alone or anti-CD3 plus anti-CD28 for the indicated times. Levels of mRNA were measured by real-time PCR. The relative expression is shown as the mean and SD of the fold induction above unstimulated T cells from three independent experiments. *indicates <0.05 in Student’s t test.

We next examined whether CD28 regulated hnRNPLL expression. We compared hnRNPLL mRNA and protein expression in naïve T cells stimulated with anti-CD3 alone or anti-CD3 plus anti-CD28. For analyses of hnRNPLL mRNA, we cultured sorted naïve CD4 T cells with anti-CD3 alone or anti-CD3 plus anti-CD28 and analyzed hnRNPLL mRNA levels at 5, 12 and 24 hours by qPCR. We found that hnRNPLL mRNA expression is increased in a CD28-specific fashion (Figure 6). The levels of hnRNPLL mRNA remained comparably low in unstimulated CD4 T cells and T cells cultured with anti-CD3 alone. In contrast, hnRNPLL mRNA levels were significantly increased by TCR/CD28 stimulation at 12 hr after stimulation and increased further by 24 hr after stimulation (2 fold and 5 fold, respectively).

**Figure 7:**
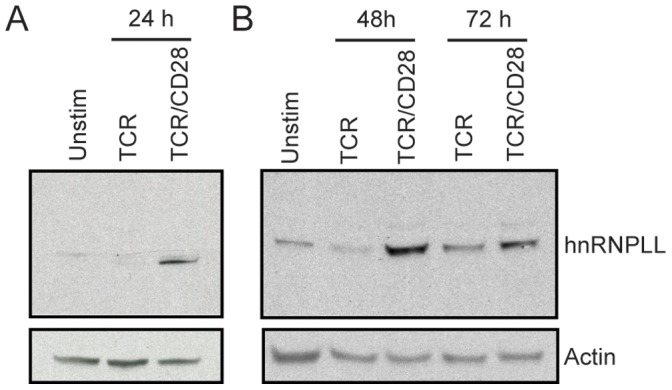
CD28 costimulation promotes and sustains hnRNPLL protein expression in CD4 T cells. Sorted naïve CD4+CD25- T cells from wild type mice were stimulated with bead-bound anti-CD3 alone or anti-CD3 plus anti-CD28 for the indicated times. (A, B) These results are representative of at least 4 independent experiments.

To analyze expression of hnRNPLL protein, we cultured sorted naïve CD4 T cells with anti-CD3 or anti-CD3 plus anti-CD28, and analyzed hnRNPLL protein expression 24, 48 and 72 hours later by Western blotting. The expression of hnRNPLL protein was negligible in unstimulated T cells and was only detected in T cells stimulated with anti-CD3 alone after 72 hr of stimulation (Figure 7). In contrast, in T cells stimulated with anti-CD3 plus anti-CD28, hnRNPLL protein was induced by 24 hours, increased to higher levels by 48 hours and sustained at 72 hr. Thus, CD28 costimulation is a critical inducer of hnRNPLL expression, and hnRNPLL splicing targets are enriched in those genes undergoing AS following CD28 costimulation. These findings suggest that one mechanism by which CD28 may exert its effects on splicing could be through promoting and sustaining expression of hnRNPLL.

## Discussion

CD28 is the major costimulatory receptor involved in activation of naïve T cells, but the molecular processes activated by CD28 and how these integrate with TCR are still not well understood. Here we identify a novel means by which CD28 exerts its immunoregulatory effects. We show that signals through CD28 lead to profound changes in splicing in naïve T cells upon their activation. CD28 costimulation induces specific effects on splicing in T cells, beyond the effects of TCR stimulation alone. CD28 costimulation affects nearly 8 times as many genes through regulation of alternate splicing than by regulation of gene transcription. While it is well established that CD28 can increase gene transcription and mRNA stability, there are relatively few CD28-specific changes in gene transcription upon TCR and CD28 co-engagement. The effects of CD28 on alternate splicing provide a previously unappreciated means by which CD28 can regulate T cell responses.

Our findings emphasize that examination of transcript abundance alone provides only a partial picture of signal-driven gene regulation, and that AS may have broad effects on T cell function. Studies of alternate splicing have demonstrated that >70% of genes are alternately spliced [3], but analyses of signal-induced splicing in T cells thus far have focused on only a handful of genes (e.g., CD45, CD44) [6]. Recent work from genome-wide association studies also has pointed to the significance of alternative splicing in immune-mediated diseases. For example, splice variants of the T cell inhibitory receptor CTLA-4 are associated with susceptibility to type 1 diabetes and autoimmune thyroid disease [15], [16], [17]. Our results give impetus to further studies of splice variants important for T cell biology.

Recent studies have identified hnRNPLL to be an important regulator of splicing in the nervous system and the immune system. The immune system and the nervous system both have the capacity to respond to a variety of stimuli. Alternative splicing appears to be an important mediator of neuronal differentiation, diversity and plasticity. Recent studies of *thunder* mutant mice, which contain a missense mutation in hnRNPLL and exhibit defects in splicing, have revealed an important role for hnRNPLL in survival of naïve and memory αβ T cells [18]. Studies of *thunder* mice revealed that hnRNPLL controls the accumulation and longevity of circulating T cells, but the mechanism by which hnRNPLL exerts these effects is not yet clear. CD28 is important for promoting T cell survival, and our study links CD28 costimulation, hnRNPLL and T cell survival. Further studies of *thunder* mice showed that hnRNPLL is necessary for appropriate AS of CD45 [19], consistent with the study of Oberdoerffer et al [14]. *Thunder* mice also have been utilized to identify hnRNPLL-dependent splicing events by using exon microarrays to compare exon usage in wild type and *thunder* mutant thymocytes and T cells [18]. The striking overlap of exons showing hnRNPLL-dependent alternative splicing in T cells and in the nervous system cells suggests that hnRNPLL may serve a master regulator of alternative splicing in a variety of cell types.

Biological processes tend to perturb groups of genes rather than individual genes; therefore computational approaches have been developed to identify the unusual overrepresentation of sets of genes of interest that might point to underlying biological mechanisms. This approach – termed “enrichment analysis” – has been used to query expression profiling datasets for the coordinate up-(or down-) regulation of a set of genes that share a common biology [20], [21]. For example, the activity of the polycomb transcriptional complex during stem cell differentiation was identified, in part, by analysis of microarray data that showed the coordinate upregulation of a set of polycomb target genes during stem cell differentiation [22]. Analogous to gene-set enrichment analysis, we developed a new method for identifying splice factors active during T cell activation and differentiation by integrating data from our exon arrays with information from an entirely different source [14]. This novel analytical approach identified a putative role for hnRNPLL in CD28-induced splicing. Thus, our data suggest that genome-wide analysis of AS can be used similarly to identify the “footprint” of alternatively spliced genes resulting from the signaling of the T cell receptor, and due to a specific splicing factor – in this case hnRNPLL. We anticipate that it should be possible to assemble – either through laboratory or analytic experiments – a compendium of sets of genes that are subject to splicing by a library of specific splicing factors. Such a compendium may prove a useful tool in the analysis of genome-wide surveys of alternative splicing to identify the role of defined splicing factors under specific biological conditions.

Further studies are needed to determine whether our finding that more genes are alternately spliced than differentially transcribed upon T cell activation is a general property of a single cell type in different activation states, or rather, a specific feature of T cells. Our findings may have broad applicability to the study of derangements of T cell function. For example, aged T cells are well-known to have different outcomes following activation, including limited cytokine production and proliferative responses [23], [24]. Whether these effects can be attributed to age-dependent changes in alternate splicing [25], as was recently shown to be the case for neuronal calcium channels [26], dopamine channels [27], and estrogen receptor alpha [28], is an exciting line of future investigation. In addition, emerging data suggest that the identification of disease-specific splice variants may provide novel biomarkers and targets for therapy. Yet, little is known about the factors that regulate alternative splicing. Our work has provided insight into the regulation of expression of hnRNPLL, a key orchestrator of splicing, in T cells and gives impetus to studies that use bioinformatics and functional approaches to investigate the impact of alternative splicing on T cell activation, differentiation, and function.

## Materials and Methods

### Mice

C57BL/6 mice (The Jackson Laboratory) and OT-II^+/+^ TCRα^−/−^ mice (courtesy of Dr. Anjana Rao) were maintained in a pathogen-free facility and used according to institutional and National Institutes of Health guidelines. Harvard Medical School is accredited by the American Association of Accreditation of Laboratory Animal Care.

#### Ethics statement

The work was performed under a protocol approved by the Harvard Standing Committee on Animals in strict accordance with the recommendations in the Guide for the care and use of Laboratory Animals of the National Institutes of Health. The Harvard Medical School animal management program is accredited by the Association for the Assessment and Accreditation of Laboratory Animal Care, International (AAALAC) (accreditation #000009), and meets National Institutes of Health standards as set forth in the Guide for the Care and Use of Laboratory Animals. The institution also accepts as mandatory the PHS Policy on Humane Care and Use of Laboratory Animals by Awardee Institutions and NIH Principles for the Utilization and Care of Vertebrate Animals Used in Testing, Research, and Training. There is on file with the Office of Laboratory Animal Welfare (OLAW) an approved Assurance of Compliance (A3431-01). Animals with euthanized using C02 and every effort was made to minimize suffering.

### T Cell Activation and RNA Preparation for Microarray Analyses

M450 glycidyl ether beads (Dynal) were covalently coupled with anti-CD3ε (clone 2C11) plus either anti-CD28 (clone 37.51) or hamster IgG isotype control (Ebioscience) as previously described [29]. 10^7^ beads were coated with 1 µg of anti-CD3ε (20% of total protein) plus either 4 µg of control hamster IgG (80% of total protein) or 1.5 µg of anti-CD28 (30% of total protein) and 2.5 µg of control hamster IgG (50% of total protein). CD4 T cells were purified from spleens of OTII^+/+^ TCRα^−/−^ mice using CD4 microbeads and positive selection (Mitenyi Biotec), and plated (10^5^ cells and 10^6^ beads/well in 0.2 mL) in U-bottom tissue culture wells for 24 hours in RPMI 1640 (Invitrogen) supplemented with 10% FBS, 2 mM L-glutamine, 10 mM HEPES, 1% penicillin/streptomycin, and 50 µM 2-mercaptoethanol (R-10 media). RNA was prepared from 10^7^ T cells (RNEasy kit, Qiagen) and further processed according to the manufacturer’s directions (Mouse Exon Array 1.0, Affymetrix) at the Dana-Farber Cancer Institute Microarray Core. Four independent replicates were performed for each condition (naive, TCR-activated, and TCR/CD28 activated).

### Exon Array Data Analysis

Analysis of mouse exon array data provides levels of expression for 4,578,603 probes making up 1,198,032 probesets dispersed among the exons of 269,283 transcripts. We corrected the expression level of each probe, after eliminating cross-hybridizing probes, using ProbeEffects software [13]. We then used selected probes to calculate expression levels for each transcript using Jetta software [13], [30], [31]. The expression level for each transcript was calculated as the average expression level of all probes comprising that transcript. For expression level, we used a cutoff of 2 fold differences and empirical Bayesian testing, Limma software package [12], to measure significance.

To calculate splicing, we followed the approach of Xing and colleagues [32] to determine a splicing index for each probe, after preprocessing to remove errant probes. The splicing index is the ratio of each probe’s background-corrected probe intensity to the average expression level of all probes for that transcript. Different splicing indices between treatment conditions would indicate differential splicing. We used the microarray analysis of differential splicing (MADS) and microarray detection of alternative splicing (MIDAS) software to calculate probability values of differential splicing for each probeset [13]. For splicing differences to be considered as significant, transcripts had to have statistically significant different *p* values (*p*<0.01) in both statistical tests.

Over-representation of Gene Ontology biological processes was analyzed using the NIH DAVID Functional Annotation web server [33], [34]. Significance values were corrected for multiple testing [35].

We identified the list of 36 genes dependent on hnRNPLL for alternative splicing from previously published data [14]. To integrate this human dataset with our mouse dataset, we first mapped the human genes to their mouse homologs using the JAX lab informatics database [http://www.informatics.jax.org/], leaving 33 unique mouse genes. We then calculated the overlap of these hnRNPLL target genes with the genes displaying significant alternative splicing in TCR/CD28 versus CD3 conditions. Statistical significance of the overlap was calculated using the hypergeometric distribution [36].

### Quantitative RT-PCR Analyses

Sorted naïve CD4+ CD25- T cells from C57BL/6 mice (>99% pure) were activated with either anti-CD3 plus isotype or anti-CD3 plus anti-CD28 mAb-coated beads in 96-well flat-bottom tissue culture plates for the indicated time points in R-10 media. Total cellular RNA was isolated by Trizol (Invitrogen), and 1 µg RNA was used to reverse-transcribe mRNA into cDNA using Reverse Transcription System (Promega), according to the manufacturer’s protocol. The level of hnRNPLL mRNA was determined by quantitative PCR using 5–10 ng cDNA in an ABI 7500 Fast System (Applied Biosystems). We normalized qPCR data by β2- microglobulin using the ΔΔC_t_ method for relative quantitation. TaqMan probes and primers were obtained from Applied Biosystems. Statistical significance was determined using the unpaired two-tailed *t* test.

### Flow Cytometry

Sorted naïve CD4+CD25- cells from either OTII^+/+^ TCRα^−/−^ or C57BL/6 mice were activated with either anti-CD3 mAb plus isotype or anti-CD3 plus anti-CD28 mAb-coated beads in 96-well flat-bottom tissue culture plates for the indicated time points in R-10 media. Cells were stained with mAbs to CD226 (Biolegend, clone 10E5) or CCR9 (Biolegend, clone 9B1), and supernatants used for analyses of IL-3 secretion by cytometric bead array (BD Biosciences). The theoretical limit of IL-3 detection is 1.1 pg/ml by this assay. Data were acquired on a LSR II (Beckton Dickinson) and analyzed with FlowJo software.

### Western Blot Analyses

Sorted naïve CD4+CD25- T cells from C57BL/6 mice were activated with either anti-CD3 plus isotype or anti-CD3 plus anti-CD28 mAb-coated beads in 96-well flat-bottom tissue culture plates for the indicated time points in R-10 media. Whole cell lysates were prepared in ice-cold lysis buffer containing 1% Triton X-100, 50 mM Tris-HCl (pH 7.5), 150 mM NaCl, 2 mM EDTA, protease inhibitor cocktail (Roche), and phosphatase inhibitor cocktail (Pierce). Protein concentration was measured by the BCA method (Pierce). Twenty µg of whole cell lysates were boiled in sample buffer, separated on 10% SDS-PAGE and analyzed by Western blotting using a hnRNPLL-specific polyclonal Ab (Abcam). The membrane was stripped and reprobed with actin Ab (Sigma). HRP-conjugated anti-rabbit or anti-mouse secondary Ab (GE Health) was used, and ECL (GE Health) was used for detection of bound antibody.

## Supporting Information

Figure S1 **Transcript-level expression for the genes most upregulated or downregulated upon activation of T cells.** Expression is shown on a log_2_ scale on the vertical axis. Colors are given for convenience of interpretation: naïve (red), TCR-activated (green) and TCR/CD28-activated (blue). Replicates shown for each transcript are fully independent across mice, days, and microarray batches. (A) Transcripts showing most differential expression comparing naïve and TCR-activated T cells. (B) Transcripts showing most differential expression comparing naïve and TCR/CD28-activated T cells. (C) Transcripts showing most differential expression comparing TCR-activated and TCR/CD28-activated T cells.
(TIF)

Figure S2 **Volcano plots showing differential expression between naïve T cells, TCR-activated T cells, and TCR/CD28-activated T cells.**
(TIF)

Figure S3 **Network visualization of functionally-related groups of genes affected by CD28-induced alternate splicing.** Relationships between of functionally-related groups of genes identified by enrichment analysis in TCR/CD28-activated T cells compared to TCR-activated T cells were visualized using GeneMANIA**.** (A) DNA metabolic process (B) RNA processing (C) RNA splicing.
(TIF)

Table S1 Transcripts expressed only in naïve T cells, TCR-activated, or TCR/CD28 Activated T cells.
(DOC)

Table S2 Biological processes enriched in transcripts expressed only in Naïve T cells or TCR/CD28 activated T cells.
(DOC)

Table S3 All genes with greater than 1.4 fold changes in transcript-level expression in TCR-activated compared to naïve T cells and TCR/CD28 activated compared to naïve T cells or TCR/CD28 activated compared to TCR-activated T cells.
(DOC)

Table S4 Unique transcripts spliced only in naïve T cells, TCR-activated T cells, or TCR/CD28 T cells.
(DOC)

Table S5 Unique Biological processes in transcripts differentially expressed or spliced between naïve T cells and TCR activated T cells, naïve T cells and TCR/CD28-activated T cells.
(DOC)

Table S6 Biological processes of the 1,047 transcripts differentially spliced between TCR-activated and TCR/CD28-activated T cells.
(DOC)
