# Supplementary figures and images for: CD28 Costimulation Regulates Genome-Wide Effects on Alternative Splicing
Source: PLoS One. 2012 Jun 29;7(6):e40032. doi: 10.1371/journal.pone.0040032 (PMC3386953; doi:10.1371/journal.pone.0040032)

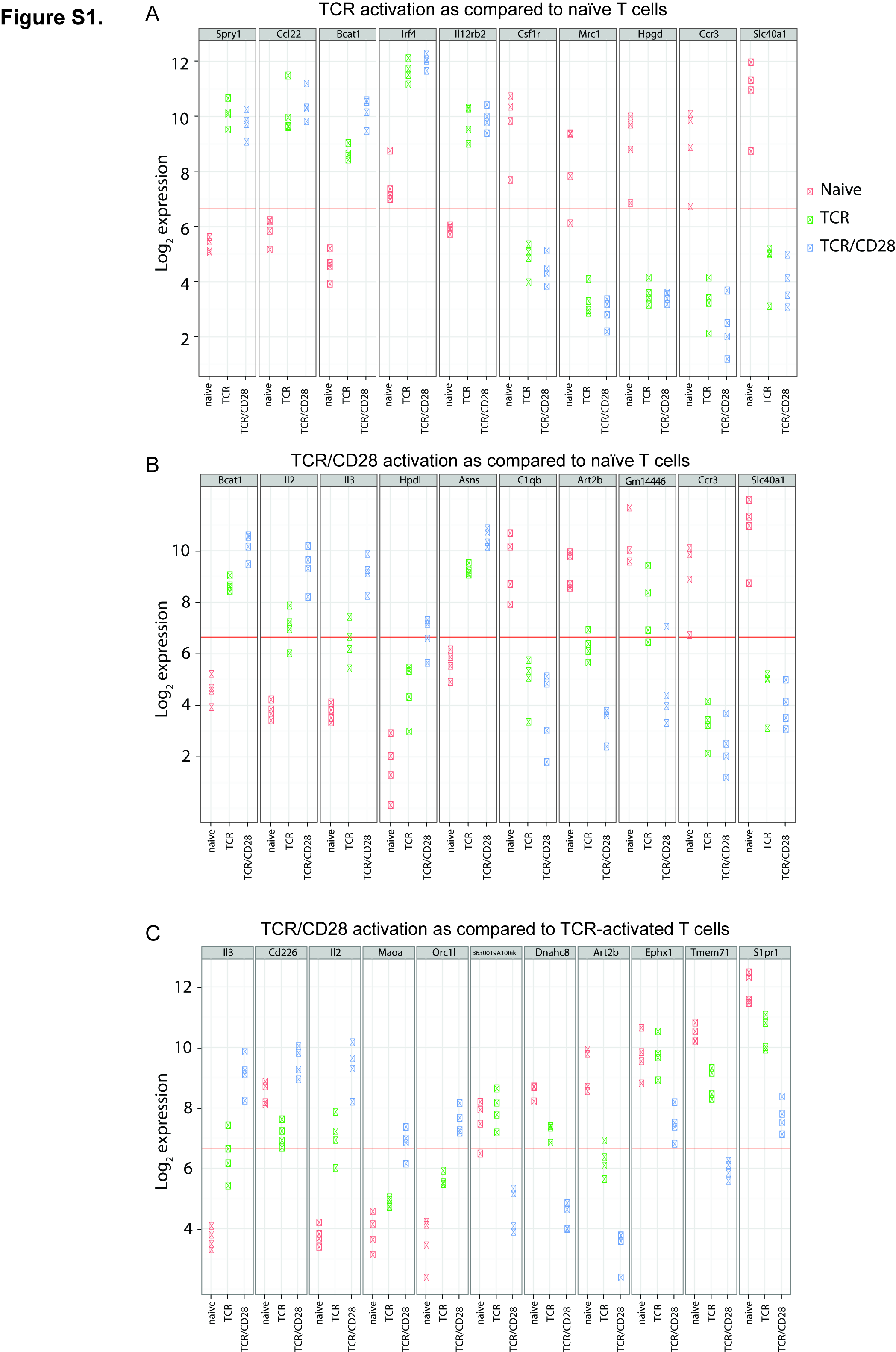

Supplement: Figure S1 — Transcript-level expression for the genes most upregulated or downregulated upon activation of T cells. Expression is shown on a log2 scale on the vertical axis. Colors are given for convenience of interpretation: naïve (red), TCR-activated (green) and TCR/CD28-activated (blue). Replicates shown for each transcript are fully independent across mice, days, and microarray batches. (A) Transcripts showing most differential expression comparing naïve and TCR-activated T cells. (B) Transcripts showing most differential expression comparing naïve and TCR/CD28-activated T cells. (C) Transcripts showing most differential expression comparing TCR-activated and TCR/CD28-activated T cells. (TIF) [file pone.0040032.s001.tif]

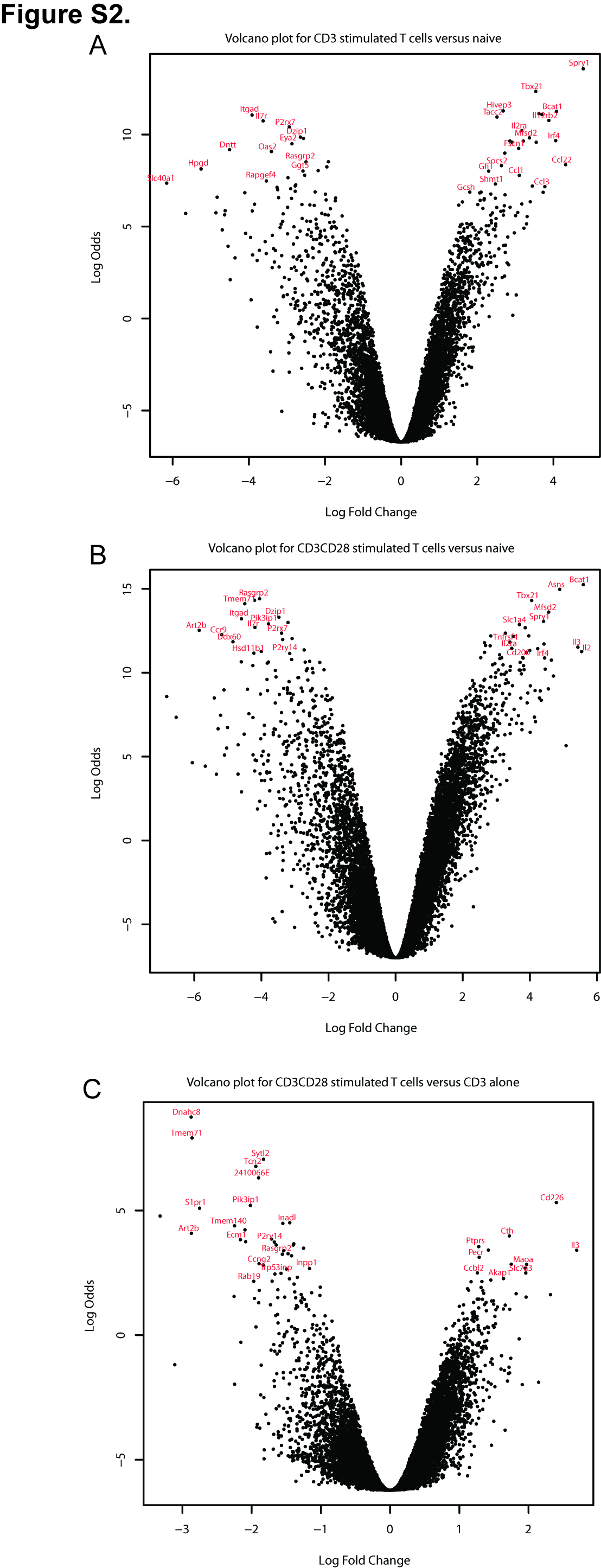

Supplement: Figure S2 — Volcano plots showing differential expression between naïve T cells, TCR-activated T cells, and TCR/CD28-activated T cells. (TIF) [file pone.0040032.s002.tif]

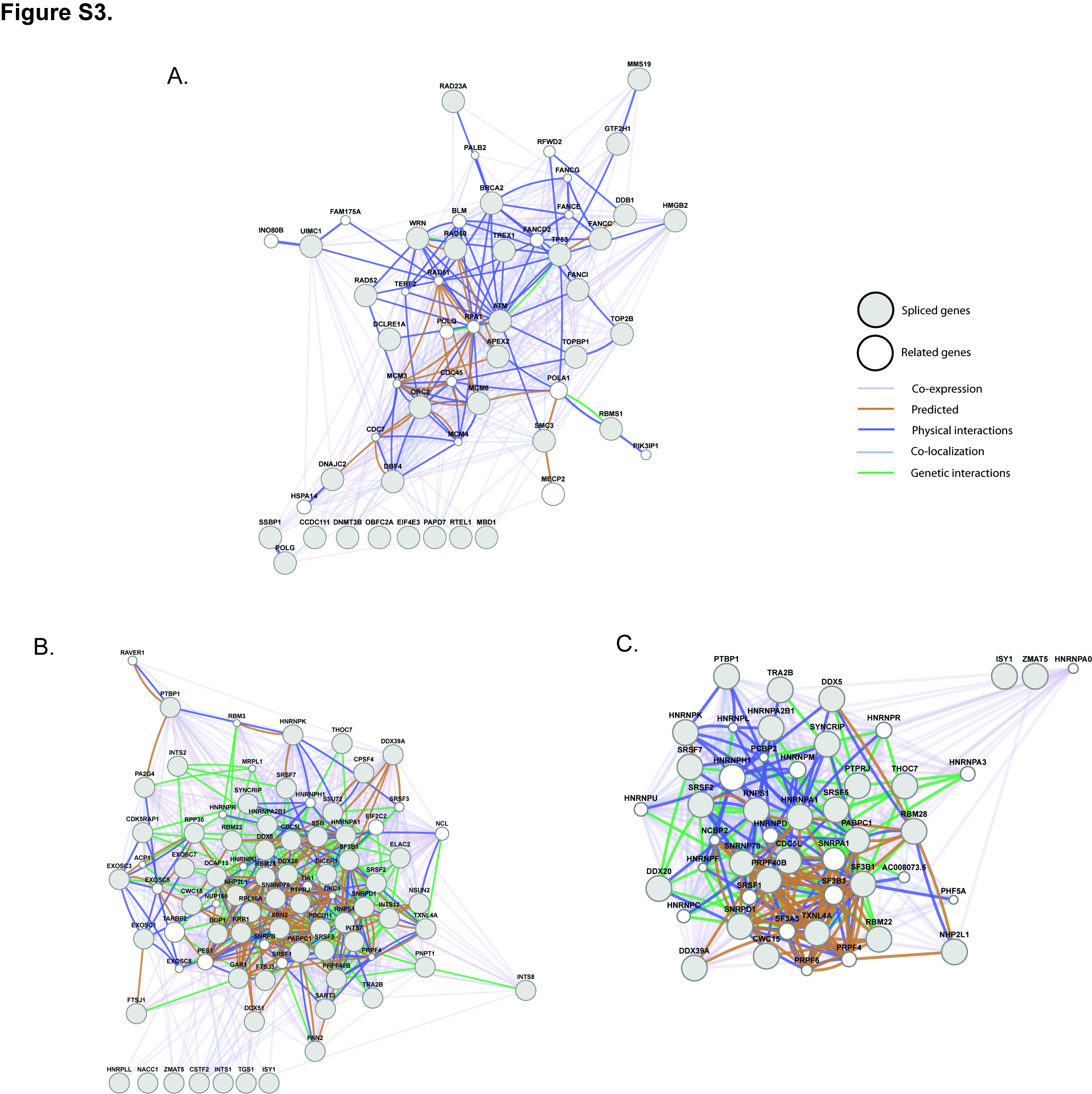

Supplement: Figure S3 — Network visualization of functionally-related groups of genes affected by CD28-induced alternate splicing. Relationships between of functionally-related groups of genes identified by enrichment analysis in TCR/CD28-activated T cells compared to TCR-activated T cells were visualized using GeneMANIA. (A) DNA metabolic process (B) RNA processing (C) RNA splicing. (TIF) [file pone.0040032.s003.tif]
