# Supplementary material for: CD28 Costimulation Regulates Genome-Wide Effects on Alternative Splicing
Source: PLoS One. 2012 Jun 29;7(6):e40032. doi: 10.1371/journal.pone.0040032 (PMC3386953; doi:10.1371/journal.pone.0040032)
Supplement: Table S1 — Transcripts expressed only in naïve T cells, TCR-activated, or TCR/CD28 Activated T cells. (DOC) [file pone.0040032.s004.doc]

**Table S1.**

**Transcripts expressed only in naïve T cells (409)**

| **TCID** | **Symbol** |
| --- | --- |
| 6747986 | Paqr8 |
| 6748553 | Arhgef4 |
| 6748886 | Il1rl2 |
| 6748893 | Il18rap |
| 6749804 | Carf |
| 6749933 | Adam23 |
| 6749935 | Adam23 |
| 6750519 | Il8rb |
| 6750546 | Slc11a1 |
| 6750566 | Cyp27a1 |
| 6751535 | Ramp1 |
| 6751623 | Gpr35 |
| 6751640 | Dusp28 |
| 6753067 | Ctse |
| 6753089 | Slc45a3 |
| 6755146 | Fcgr4 |
| 6755189 | Cd244 |
| 6755237 | Kcnj10 |
| 6758435 | Slc40a1 |
| 6759718 | Tns1 |
| 6760518 | Arl4c |
| 6760678 | Hes6 |
| 6761964 | Tmem163 |
| 6762429 | Nav1 |
| 6762804 | Rgs18 |
| 6763090 | Rgl1 |
| 6763196 | Cacna1e |
| 6763208 | Mr1 |
| 6763777 | Mpzl1 |
| 6764040 | Fcgr3 |
| 6764578 | Cabc1 |
| 6764832 | Hlx |
| 6765281 | Rcor3 |
| 6765325 | Hsd11b1 |
| 6765327 | G0s2 |
| 6766455 | Sgk1 |
| 6767088 | Tspyl4 |
| 6768207 | Prf1 |
| 6768572 | Tmem26 |
| 6768868 | Ggt5 |
| 6768898 | Vpreb3 |
| 6772009 | Sash1 |
| 6772104 | Rab32 |
| 6773174 | Dse |
| 6774784 | A930033H14Rik |
| 6775146 | Susd2 |
| 6775236 | Trpm2 |
| 6775466 | Nfic |
| 6775559 | Slc41a2 |
| 6775576 | Appl2 |
| 6775864 | Spic |
| 6777249 | Kcnmb4 |
| 6777310 | Lyz1 |
| 6777957 | Lrp1 |
| 6781111 | Slc36a1 |
| 6782286 | Itgae |
| 6782422 | Rtn4rl1 |
| 6782430 | Smyd4 |
| 6783005 | Slfn4 |
| 6783040 | OTTMUSG00000000971 |
| 6783259 | Tubd1 |
| 6783785 | Gngt2 |
| 6784042 | Nr1d1 |
| 6784280 | Tmem106a |
| 6784283 | Rdm1 |
| 6784587 | Ace3 |
| 6784765 | Axin2 |
| 6784829 | Map2k6 |
| 6785079 | Ttyh2 |
| 6785111 | Cd300a |
| 6785306 | Afmid |
| 6785317 | Socs3 |
| 6785369 | Slc26a11 |
| 6787918 | Psme2 |
| 6788393 | Ccdc69 |
| 6788741 | Tom1l2 |
| 6788928 | Zfp287 |
| 6789544 | Spns3 |
| 6790290 | Ccl6 |
| 6790944 | Abcc3 |
| 6791233 | Cacnb1 |
| 6791422 | Jup |
| 6791437 | Dhx58 |
| 6791561 | Slc4a1 |
| 6791696 | Arhgap27 |
| 6792367 | Cd300lb |
| 6792371 | 4732429D16Rik |
| 6792390 | RP23 |
| 6792392 | Cd300e |
| 6792406 | Cd300lf |
| 6792606 | Tha1 |
| 6792832 | Cd7 |
| 6793961 | Cmpk2 |
| 6795779 | Klhdc1 |
| 6795936 | Daam1 |
| 6796027 | Slc38a6 |
| 6796339 | Rdh12 |
| 6796691 | Fos |
| 6798213 | Tnfaip2 |
| 6798271 | Adssl1 |
| 6798313 | Crip2 |
| 6799525 | Rsad2 |
| 6800020 | Hdac9 |
| 6800228 | Arl4a |
| 6801506 | Pygl |
| 6803102 | Rps6ka5 |
| 6805108 | Naip3 |
| 6806073 | Nqo2 |
| 6806080 | Bphl |
| 6807156 | Hrh2 |
| 6807336 | Tgfbi |
| 6809032 | Arsb |
| 6809551 | Ccdc125 |
| 6811694 | Hfe |
| 6811977 | Sox4 |
| 6812065 | Hus1b |
| 6812250 | 1300014I06Rik |
| 6813474 | Tifab |
| 6813880 | Cdc14b |
| 6814451 | Arsk |
| 6814996 | Atg10 |
| 6815522 | Naip2 |
| 6815523 | Naip5 |
| 6815529 | Birc1f |
| 6816087 | Ncf2 |
| 6817334 | Nudt13 |
| 6819143 | Abhd4 |
| 6824363 | D14Ertd436e |
| 6824728 | Slc7a7 |
| 6824825 | 1500005A01Rik |
| 6827820 | Dzip1 |
| 6828125 | D14Ertd668e |
| 6828403 | C6 |
| 6829612 | Pgcp |
| 6832132 | Tef |
| 6833305 | Acvrl1 |
| 6837375 | Cyp2d22 |
| 6837415 | Nfam1 |
| 6837787 | Plxnb2 |
| 6838455 | C230021P08Rik |
| 6838563 | Csnrp2 |
| 6838565 | Pou6f1 |
| 6838713 | Csad |
| 6838717 | Rarg |
| 6840432 | BC022623 |
| 6840637 | Itgb5 |
| 6841136 | Cd200r1 |
| 6843550 | Mx1 |
| 6844530 | 2510009E07Rik |
| 6844531 | Ehhadh |
| 6844601 | BC106179 |
| 6848199 | Mx1 |
| 6848721 | D17Ertd663e |
| 6849622 | Fgd2 |
| 6849891 | Cyp4f16 |
| 6849950 | Adamts10 |
| 6849951 | Myo1f |
| 6850534 | Pla2g7 |
| 6850552 | Enpp5 |
| 6850763 | Trerf1 |
| 6850819 | Trem3 |
| 6850821 | Treml4 |
| 6850831 | A530064D06Rik |
| 6851186 | Emr4 |
| 6851324 | Emr1 |
| 6851897 | Epb4 |
| 6852471 | Galm |
| 6852836 | Epas1 |
| 6854043 | Fpr1 |
| 6854231 | A630033E08Rik |
| 6854930 | Zfp101 |
| 6855727 | A330017A19Rik |
| 6856203 | Lrg1 |
| 6856245 | AI662250 |
| 6856290 | C3 |
| 6856676 | Rab31 |
| 6857065 | Emilin2 |
| 6857183 | Xdh |
| 6857415 | Fez2 |
| 6861358 | Csf1r |
| 6862627 | Zfp516 |
| 6866112 | Fech |
| 6867618 | Aldh3b2 |
| 6869223 | B430203M17Rik |
| 6869327 | Ifit3 |
| 6869691 | Dntt |
| 6871206 | Ppp2r5b |
| 6872051 | Gda |
| 6872528 | Insl6 |
| 6873153 | Frat2 |
| 6873237 | Dnmbp |
| 6873503 | Sh3pxd2a |
| 6875181 | Mrc1 |
| 6875421 | Msrb2 |
| 6875637 | Il1f9 |
| 6876944 | 2310010M24Rik |
| 6877139 | Fmnl2 |
| 6877909 | Dhrs9 |
| 6878038 | Rapgef4 |
| 6878045 | Rapgef4 |
| 6880683 | Sord |
| 6880776 | Sema6d |
| 6881087 | Mertk |
| 6881101 | Zc3h6 |
| 6883125 | Mmp9 |
| 6883186 | Eya2 |
| 6884235 | 9230112E08Rik |
| 6884520 | Cugbp2 |
| 6885432 | Fcna |
| 6885873 | Lcn2 |
| 6885930 | Ralgps1 |
| 6886039 | Stom |
| 6888752 | Nr1h3 |
| 6890127 | Bmf |
| 6890636 | Hdc |
| 6890838 | Il1b |
| 6890981 | Siglec1 |
| 6892579 | Tgm2 |
| 6892699 | Mafb |
| 6892899 | Slpi |
| 6892964 | Pltp |
| 6893057 | Sulf2 |
| 6895856 | Car2 |
| 6898477 | Serpini1 |
| 6898972 | Cd1d2 |
| 6898995 | Cd5l |
| 6899372 | S100a4 |
| 6899374 | S100a6 |
| 6899667 | 4930504E06Rik |
| 6900287 | ENSMUSG00000074335 |
| 6900385 | Sort1 |
| 6900975 | Arhgap29 |
| 6901196 | Tifa |
| 6901460 | C030007I09Rik |
| 6902179 | Mcoln3 |
| 6903360 | Sirpb1 |
| 6905408 | P2ry14 |
| 6905422 | P2ry13 |
| 6906912 | S100a9 |
| 6907810 | Magi3 |
| 6907945 | Chi3l3 |
| 6908137 | Gpsm2 |
| 6908486 | Vcam1 |
| 6910592 | Ifi44 |
| 6911010 | Sort1 |
| 6911212 | Rusc1 |
| 6913985 | Rgs3 |
| 6914190 | Tlr4 |
| 6915844 | Dnajc6 |
| 6916322 | 2310026E23Rik |
| 6916815 | Cited4 |
| 6917120 | Csf3r |
| 6917549 | Fgr |
| 6917656 | Paqr7 |
| 6918015 | Pla2g2d |
| 6918125 | Padi2 |
| 6918349 | Dhrs3 |
| 6920754 | Mobkl2b |
| 6922541 | Megf9 |
| 6925519 | Tlr12 |
| 6926165 | C1qb |
| 6926166 | C1qc |
| 6926167 | C1qa |
| 6927426 | Dhrs3 |
| 6929655 | Khk |
| 6929920 | Afap1 |
| 6931759 | Kit |
| 6932367 | Ppbp |
| 6933139 | C230066G23Rik |
| 6933441 | A630023P12Rik |
| 6933625 | Oasl2 |
| 6933812 | Tesc |
| 6933973 | Slc24a6 |
| 6933997 | Oas1b |
| 6934162 | P2rx7 |
| 6934972 | Rasa4 |
| 6935296 | Amz1 |
| 6935701 | Alox5ap |
| 6938631 | Rell1 |
| 6938679 | Tlr6 |
| 6940363 | Hpse |
| 6940431 | Wdfy3 |
| 6940432 | Wdfy3 |
| 6941146 | Adrbk2 |
| 6941647 | Oas2 |
| 6941844 | Gpr109a |
| 6942579 | Pilra |
| 6942580 | Pilrb1 |
| 6942739 | Iqce |
| 6943476 | Rbm47 |
| 6945623 | Adck2 |
| 6945775 | Gstk1 |
| 6945967 | A230106D06Rik |
| 6946339 | Chn2 |
| 6946370 | 2410066E13Rik |
| 6946920 | Cd8a |
| 6947939 | Slc41a3 |
| 6949086 | Ttll3 |
| 6949722 | Clec4a1 |
| 6949727 | Clec4a3 |
| 6949744 | Clec4n |
| 6949746 | Clec4d |
| 6949766 | Cd163 |
| 6949856 | Vamp1 |
| 6950137 | Clec12a |
| 6950170 | Klrd1 |
| 6952926 | Parp12 |
| 6953800 | Nod1 |
| 6954269 | Ptgds2 |
| 6954988 | Sema4f |
| 6955137 | Sfxn5 |
| 6955778 | Frmd4b |
| 6956679 | <No gene symbol, 6q22.1> |
| 6957025 | Klrg1 |
| 6957427 | Klri1 |
| 6957758 | Art4 |
| 6958974 | Pglyrp1 |
| 6959474 | Rasgrp4 |
| 6959584 | Tyrobp |
| 6960404 | Dbp |
| 6960516 | Tmem86a |
| 6960834 | Siglech |
| 6961108 | Mtmr10 |
| 6961766 | Klhl25 |
| 6961767 | Klhl25 |
| 6962027 | Zscan2 |
| 6962779 | Pak1 |
| 6962951 | D930046H04Rik |
| 6963456 | Ampd3 |
| 6963566 | A630005I04Rik |
| 6963898 | 2610020H08Rik |
| 6964172 | D430042O09Rik |
| 6964247 | Tbx6 |
| 6964380 | Itgam |
| 6964382 | Itgad |
| 6964798 | Ptpre |
| 6965146 | Ifitm1 |
| 6965430 | Zc3h3 |
| 6965885 | Cd177 |
| 6965950 | Ceacam2 |
| 6966203 | Sipa1l3 |
| 6966282 | Hcst |
| 6966808 | Cd33 |
| 6966818 | Siglece |
| 6966935 | Fcgrt |
| 6968735 | Anpep |
| 6968780 | Fes |
| 6969878 | Art2b |
| 6970060 | A530023O14Rik |
| 6970952 | Igsf6 |
| 6971323 | Qprt |
| 6972106 | Adam8 |
| 6972491 | Ccnd1 |
| 6973527 | Lair1 |
| 6974198 | Ifitm1 |
| 6975052 | Rnf122 |
| 6976237 | Hpgd |
| 6976609 | Ddx60 |
| 6977648 | Tbc1d9 |
| 6977758 | Lyl1 |
| 6978232 | Capns2 |
| 6978817 | Fbxl8 |
| 6978883 | Lypla3 |
| 6978937 | Sntb2 |
| 6979196 | Terf2ip |
| 6979743 | Rab4a |
| 6980091 | Cd209a |
| 6980378 | Rab20 |
| 6980606 | 2610019F03Rik |
| 6983299 | Cyp4f18 |
| 6983604 | Mmaa |
| 6983790 | D830024N08Rik |
| 6984966 | Fhod1 |
| 6985252 | Hp |
| 6986733 | Mmp8 |
| 6987324 | 2210010B09Rik |
| 6987891 | Prdm10 |
| 6988711 | Amica1 |
| 6989917 | Parp16 |
| 6991531 | Paqr9 |
| 6992380 | Pfkfb4 |
| 6992436 | Ngp |
| 6992482 | Ltf |
| 6992849 | Eomes |
| 6992855 | Itga9 |
| 6992994 | Vipr1 |
| 6993138 | Ccr9 |
| 6993151 | Ccr3 |
| 6993153 | Ccr2 |
| 6993878 | Zfp810 |
| 6995233 | Cep164 |
| 6996438 | Aph1c |
| 6998069 | BC043934 |
| 6998094 | Acpl2 |
| 6998397 | Trf |
| 6998676 | Tcta |
| 6998893 | Tmie |
| 6999682 | Fyco1 |
| 7010645 | Il13ra1 |
| 7013185 | Tlr13 |
| 7014799 | <No gene symbol, Xq28> |
| 7015398 | Ppp1r3f |
| 7017600 | L1cam |
| 7017629 | 1810037C20Rik |
| 7017663 | Gab3 |
| 7017678 | Mtcp1 |
| 7018290 | 2810002O09Rik |
| 7019499 | Xkrx |
| 7023069 | Jarid1d |

**Transcripts expressed only in TCR-activated T cells (and not naïve or TCR/CD28-activated T cells) (40)**

| **TCID** | **Symbol** |
| --- | --- |
| 6749911 | Nrp2 |
| 6776152 | 4932415G12Rik |
| 6778047 | Ikzf4 |
| 6785746 | Polm |
| 6789420 | Cxcl16 |
| 6800229 | Scin |
| 6802745 | Ston2 |
| 6806038 | Serpinb9b |
| 6810697 | Akr1c18 |
| 6824768 | Homez |
| 6830697 | Trmt12 |
| 6842009 | Pros1 |
| 6848688 | Agpat4 |
| 6852882 | Tacstd1 |
| 6855067 | Hspa1a |
| 6869436 | Hectd2 |
| 6871062 | Npas4 |
| 6871181 | Tm7sf2 |
| 6874947 | Itih5 |
| 6883034 | Pkig |
| 6904066 | Gnb4 |
| 6911729 | Cdh17 |
| 6918382 | OTTMUSG00000010173 |
| 6918577 | LOC433762 |
| 6919741 | ENSMUSG00000073995 |
| 6921258 | Zbtb5 |
| 6928496 | ENSMUSG00000053178 |
| 6933990 | Rasal1 |
| 6934854 | Gats |
| 6946055 | Gpnmb |
| 6957083 | Clec4e |
| 6969765 | Neu3 |
| 6972415 | Tnfrsf23 |
| 6977775 | Dnase2a |
| 6987379 | 8030498B09Rik |
| 6993465 | Endod1 |
| 6994353 | St14 |
| 6994883 | Crtam |
| 7017604 | Renbp |
| 7020765 | Rab9 |

**Transcripts expressed only in TCR/CD28-activated T cells (and not naïve or TCR-activated T cells) (255)**

| **TCID** | **Symbol** |
| --- | --- |
| 6748503 | Rab23 |
| 6749044 | AI597479 |
| 6749473 | Osgepl1 |
| 6749701 | Sgol2 |
| 6749727 | Ndufb3 |
| 6751215 | Fbxo36 |
| 6751645 | Capn10 |
| 6754666 | Kifap3 |
| 6755233 | Igsf8 |
| 6755679 | Lin9 |
| 6756345 | Nsl1 |
| 6757744 | Bag2 |
| 6759613 | Bard1 |
| 6759648 | Pecr |
| 6760009 | Serpine2 |
| 6760771 | Mterfd2 |
| 6763972 | Nuf2 |
| 6764353 | Chml |
| 6765716 | Lrp11 |
| 6766381 | 2610016C23Rik |
| 6767235 | Tube1 |
| 6767527 | Magmas |
| 6768114 | Ankrd57 |
| 6769366 | Chst11 |
| 6773622 | 1700021F05Rik |
| 6775206 | Adarb1 |
| 6775830 | 4930547N16Rik |
| 6776688 | BC067068 |
| 6777879 | Xrcc6bp1 |
| 6779834 | Mpg |
| 6780782 | Mgat4b |
| 6782005 | A030009H04Rik |
| 6783682 | Cdc34 |
| 6784329 | BC030867 |
| 6785307 | Birc5 |
| 6785348 | D230014K01Rik |
| 6785641 | 1700020C11Rik |
| 6787020 | Chac2 |
| 6787100 | Rhbdf1 |
| 6788291 | Il13 |
| 6788333 | Il3 |
| 6789351 | Nlgn2 |
| 6789525 | Med31 |
| 6789693 | Sgsm2 |
| 6789863 | Ankrd13b |
| 6790648 | Akap1 |
| 6790966 | Eme1 |
| 6791644 | 3000004C01Rik |
| 6795068 | Nubpl |
| 6795599 | Fancm |
| 6797507 | D230037D09Rik |
| 6798234 | Zfyve21 |
| 6798315 | 4930427A07Rik |
| 6801335 | C79407 |
| 6801451 | 1110034A24Rik |
| 6803862 | Xrcc3 |
| 6804667 | Bzw1 |
| 6804940 | AW209491 |
| 6805245 | Hist1h2bn |
| 6807022 | Cks2 |
| 6807812 | Zfp759 |
| 6807899 | Fastkd3 |
| 6811526 | Hist1h2bl |
| 6812770 | Tbc1d7 |
| 6815518 | Mccc2 |
| 6815551 | Mrps36 |
| 6815555 | Cenph |
| 6816248 | Itga1 |
| 6817956 | Nek4 |
| 6817970 | Nt5dc2 |
| 6819883 | Pbk |
| 6822173 | Rap2a |
| 6823221 | Comtd1 |
| 6824974 | F630043A04Rik |
| 6825436 | Esco2 |
| 6829759 | Polr2k |
| 6830638 | Wdr67 |
| 6831628 | Cks2 |
| 6831849 | Pdxp |
| 6832086 | Sgsm3 |
| 6832395 | Trmu |
| 6833404 | Espl1 |
| 6834728 | 3021401C12Rik |
| 6835776 | Dscc1 |
| 6836602 | Ptk2 |
| 6836723 | Ly6i |
| 6836849 | Recql4 |
| 6837131 | Cbx6 |
| 6838410 | Rhebl1 |
| 6838809 | Itga5 |
| 6838926 | Pdxp |
| 6839847 | Gnb1l |
| 6840677 | Ccdc14 |
| 6844184 | 2410018G20Rik |
| 6844250 | 2610318N02Rik |
| 6844254 | Ccdc116 |
| 6846575 | A930013N22Rik |
| 6849294 | 1700012G19Rik |
| 6850637 | Aars2 |
| 6850646 | Slc35b2 |
| 6851292 | Alkbh7 |
| 6854389 | Ndufb10 |
| 6854430 | Chtf18 |
| 6854449 | 9530058B02Rik |
| 6854988 | Rps18 |
| 6856096 | Sgol1 |
| 6857078 | Ndc80 |
| 6861707 | Tubb6 |
| 6865229 | Ticam2 |
| 6866305 | Spire1 |
| 6867593 | 1810055G02Rik |
| 6867626 | Nudt8 |
| 6867707 | Slc29a2 |
| 6867825 | Znhit2 |
| 6867885 | Macrod1 |
| 6867917 | 2700081O15Rik |
| 6870010 | 5330431N19Rik |
| 6870068 | Tmem180 |
| 6870102 | 2010012O05Rik |
| 6871192 | Sac3d1 |
| 6871493 | 2810441K11Rik |
| 6871771 | Cep78 |
| 6873083 | Tctn3 |
| 6874954 | Sfmbt2 |
| 6875094 | Pter |
| 6876044 | Slc39a1 |
| 6876089 | Usp20 |
| 6878712 | Slc43a1 |
| 6879512 | Commd9 |
| 6880468 | D2Ertd750e |
| 6880553 | Mapkbp1 |
| 6881340 | Mcm8 |
| 6883114 | Ube2c |
| 6883526 | Pfdn4 |
| 6884294 | Tcea2 |
| 6884339 | Rpp38 |
| 6885728 | Wdr34 |
| 6885912 | Stxbp1 |
| 6886017 | Phf19 |
| 6888011 | Sestd1 |
| 6888299 | Tfpi |
| 6889585 | Mett5d1 |
| 6890205 | Dnajc17 |
| 6890382 | Hisppd2a |
| 6892285 | Cdk5rap1 |
| 6895679 | Myef2 |
| 6896852 | Bbs12 |
| 6897908 | P2ry1 |
| 6899034 | Crabp2 |
| 6899052 | Gpatch4 |
| 6899578 | Tdrkh |
| 6901732 | Rg9mtd2 |
| 6906830 | Adam15 |
| 6908078 | Gstm1 |
| 6910938 | Cth |
| 6911719 | E130016E03Rik |
| 6913569 | Tmem38b |
| 6915843 | Ak3l1 |
| 6916190 | Orc1l |
| 6916483 | Stil |
| 6916762 | Lepre1 |
| 6917180 | Clspn |
| 6917496 | Sesn2 |
| 6917630 | Ubxd5 |
| 6917835 | Luzp1 |
| 6919200 | C1qdc2 |
| 6919748 | 6720467C03Rik |
| 6921067 | 2810432D09Rik |
| 6924813 | Hpdl |
| 6924834 | Kif2c |
| 6926225 | Alpl |
| 6930883 | Tbc1d19 |
| 6933697 | Cit |
| 6934310 | 6330548G22Rik |
| 6935060 | Ufsp1 |
| 6935756 | Brca2 |
| 6936611 | Rpl22l1 |
| 6936780 | Xrcc2 |
| 6942694 | Psmg3 |
| 6944982 | 2310016C08Rik |
| 6945818 | Olfr449 |
| 6946365 | Plekha8 |
| 6947558 | Alms1 |
| 6949206 | Tsen2 |
| 6949367 | Zfp239 |
| 6949814 | Cdca3 |
| 6950070 | Foxm1 |
| 6952872 | Zc3hav1l |
| 6955272 | 2010301N04Rik |
| 6957252 | Rad51ap1 |
| 6957330 | Tulp3 |
| 6957754 | Hist4h4 |
| 6960760 | Mrps21 |
| 6961182 | Tarsl2 |
| 6961987 | Prc1 |
| 6963856 | 6330503K22Rik |
| 6964147 | Jmjd5 |
| 6964553 | Pstk |
| 6964635 | 2310007H09Rik |
| 6965072 | Utf1 |
| 6965847 | Zfp109 |
| 6966229 | BC027344 |
| 6966267 | Wdr62 |
| 6966903 | Atf5 |
| 6967211 | E2f8 |
| 6968126 | Arrdc4 |
| 6969899 | Trpc2 |
| 6971277 | Nupr1 |
| 6971320 | Kif22 |
| 6973238 | Rdh13 |
| 6973287 | Zfp446 |
| 6974784 | Thap1 |
| 6977078 | Gtpbp3 |
| 6977153 | 1700030K09Rik |
| 6977814 | Gpt2 |
| 6979652 | Acsf3 |
| 6979941 | 1810063B05Rik |
| 6980944 | Ckap2 |
| 6982649 | BC088983 |
| 6983839 | 2410018C20Rik |
| 6983950 | Neto2 |
| 6985426 | Adat1 |
| 6987137 | BC017612 |
| 6990042 | Car12 |
| 6990948 | Ttk |
| 6992328 | Nicn1 |
| 6992374 | Slc26a6 |
| 6992385 | Ccdc51 |
| 6992430 | Tmem103 |
| 6992452 | Ccdc12 |
| 6993833 | Spc24 |
| 6995041 | Rnf26 |
| 6997990 | Chst2 |
| 6998340 | Cep63 |
| 6998584 | Abhd14a |
| 7000782 | 3110070M22Rik |
| 7009896 | RP23 |
| 7010183 | Maoa |
| 7011040 | Bcorl1 |
| 7011052 | Rbmx2 |
| 7011872 | 2610030H06Rik |
| 7012598 | Apoo |
| 7012842 | Kif4 |
| 7012845 | Dlg3 |
| 7013857 | Cenpi |
| 7013982 | 6530401D17Rik |
| 7014308 | Alg13 |
| 7014652 | Sumo3 |
| 7015028 | Fancb |
| 7015392 | 2010204K13Rik |
| 7018041 | Gyk |
| 7018524 | Slc7a3 |
| 7018582 | Ercc6l |
| 7018915 | Eif4b |
| 7019532 | Armcx6 |
