# Supplementary material for: CD28 Costimulation Regulates Genome-Wide Effects on Alternative Splicing
Source: PLoS One. 2012 Jun 29;7(6):e40032. doi: 10.1371/journal.pone.0040032 (PMC3386953; doi:10.1371/journal.pone.0040032)
Supplement: Table S2 — Biological processes enriched in transcripts expressed only in Naïve T cells or TCR/CD28 activated T cells. (DOC) [file pone.0040032.s005.doc]

Table S2.

Biological processes statistically enriched in the 409 transcripts expressed only in Naïve T cells

| **Gene Ontology Biological Process** | **GO ID** | **p-value of enrichment compared to whole genome** | **Gene symbols** |
| --- | --- | --- | --- |
| immune response | GO:0006955 | 3.42E-12 | C3 Ccr2 Tlr12 Mx1 Cd7 Oas2 P2ry14 Tlr13 Eomes Pglyrp1 Il18rap 4732429D16Rik RP23 Cd300e Fcgrt Mx1 Clec4n C1qa C1qc C1qb Dhx58 Il1f9 Tlr4 Cd300lf Il1rl2 Oas1b Rsad2 Hfe Oasl2 Susd2 Tlr6 Cd8a Ccl6 Ppbp Cd300lb Slc11a1 Myo1f Mr1 Cd1d2 Fcgr3 Il1b Clec4d |
| defense response | GO:0006952 | 1.84E-06 | C3 Ccr2 Tlr12 Mx1 Tlr13 Pglyrp1 Il18rap Nod1 Chi3l3 Fgr Mx1 P2rx7 C1qa C1qc C1qb Lyz1 Dhx58 Pla2g7 Tlr4 Il1rl2 Cd163 Rsad2 Ngp Tlr6 Hdac9 Trf Ggt5 Slc11a1 Myo1f Cd1d2 Fcgr3 Il1b |
| innate immune response | GO:0045087 | 1.69E-05 | Mx1 C3 C1qa C1qc C1qb Dhx58 Tlr12 Mx1 Tlr4 Tlr13 Il1rl2 Il18rap Tlr6 Slc11a1 Cd1d2 |
| inflammatory response | GO:0006954 | 2.99E-04 | C3 Ccr2 C1qa P2rx7 C1qc C1qb Tlr12 Pla2g7 Tlr4 Tlr13 Cd163 Tlr6 Hdac9 Chi3l3 Trf Ggt5 Slc11a1 Il1b Fcgr3 |
| taxis | GO:0042330 | 0.022455395 | Ccr3 Kit Il8rb Itgam Ccl6 Ccr9 S100a9 Fpr1 Il1b Csf3r Fcgr3 |
| chemotaxis | GO:0006935 | 0.022455395 | Ccr3 Kit Il8rb Itgam Ccl6 Ccr9 S100a9 Fpr1 Il1b Csf3r Fcgr3 |
| response to wounding | GO:0009611 | 0.022515954 | C3 Rtn4rl1 Ccr2 C1qa P2rx7 C1qc C1qb Tlr12 Pla2g7 Tlr4 Tlr13 Cd163 Tlr6 Hdac9 Chi3l3 Trf Ggt5 Slc11a1 Il1b Fcgr3 |

Biological processes statistically enriched in the 40 transcripts expressed only in TCR-activated T cells

none

Biological processes statistically enriched in the 255 transcripts expressed only in TCR/CD28-activated T cells

| **Gene Ontology Biological Process** | **GO ID** | **p-value of enrichment compared to whole genome** | **Gene symbols** |
| --- | --- | --- | --- |
| cell cycle | GO:0007049 | 9.15E-09 | Ercc6l Chtf18 Cks2 Nsl1 Prc1 Ndc80 Sgol1 Mcm8 Dscc1 Sgol2 Clspn Lin9 E2f8 Cks2 Spc24 Cenph F630043A04Rik Cit Sgsm3 Birc5 Thap1 Sesn2 Sac3d1 Esco2 Brca2 Ube2c Nuf2 Espl1 C79407 Nek4 Ckap2 Cdca3 |
| cell division | GO:0051301 | 1.51E-06 | F630043A04Rik Cenph Cit Birc5 Ercc6l Nsl1 Cks2 Sac3d1 Prc1 Ube2c Ndc80 Brca2 Nuf2 Sgol1 C79407 Nek4 Sgol2 Cdca3 Cks2 Spc24 |
| M phase | GO:0000279 | 1.13E-06 | F630043A04Rik Cenph Cit Birc5 Ercc6l Nsl1 Cks2 Sac3d1 Ube2c Ndc80 Brca2 Nuf2 Sgol1 Espl1 C79407 Nek4 Sgol2 Cdca3 Cks2 Spc24 |
| cell cycle process | GO:0022402 | 4.93E-06 | F630043A04Rik Cenph Cit Sgsm3 Birc5 Ercc6l Nsl1 Cks2 Sesn2 Sac3d1 Ube2c Ndc80 Brca2 Nuf2 Sgol1 Espl1 C79407 Nek4 Sgol2 Cdca3 Cks2 Spc24 |
| nuclear division | GO:0000280 | 6.33E-06 | F630043A04Rik Cenph Cit Birc5 Ercc6l Nsl1 Sac3d1 Ube2c Ndc80 Nuf2 Sgol1 C79407 Nek4 Cdca3 Spc24 |
| mitosis | GO:0007067 | 6.33E-06 | F630043A04Rik Cenph Cit Birc5 Ercc6l Nsl1 Sac3d1 Ube2c Ndc80 Nuf2 Sgol1 C79407 Nek4 Cdca3 Spc24 |
| cell cycle phase | GO:0022403 | 5.57E-06 | F630043A04Rik Cenph Cit Birc5 Ercc6l Nsl1 Cks2 Sac3d1 Ube2c Ndc80 Brca2 Nuf2 Sgol1 Espl1 C79407 Nek4 Sgol2 Cdca3 Cks2 Spc24 |
| chromosome segregation | GO:0007059 | 5.26E-06 | Cenph F630043A04Rik Cit Nuf2 Birc5 Sgol1 Espl1 Nsl1 Sgol2 Ndc80 |
| M phase of mitotic cell cycle | GO:0000087 | 5.14E-06 | F630043A04Rik Cenph Cit Birc5 Ercc6l Nsl1 Sac3d1 Ube2c Ndc80 Nuf2 Sgol1 C79407 Nek4 Cdca3 Spc24 |
| organelle fission | GO:0048285 | 5.54E-06 | F630043A04Rik Cenph Cit Birc5 Ercc6l Nsl1 Sac3d1 Ube2c Ndc80 Nuf2 Sgol1 C79407 Nek4 Cdca3 Spc24 |
| mitotic cell cycle | GO:0000278 | 6.89E-05 | F630043A04Rik Cenph Cit Birc5 Ercc6l Nsl1 Sac3d1 Ube2c Ndc80 Nuf2 Sgol1 C79407 Nek4 Cdca3 Spc24 |
| DNA metabolic process | GO:0006259 | 1.21E-04 | Rad51ap1 Fancb Chtf18 2010301N04Rik Esco2 Brca2 Mpg Eme1 Xrcc3 Mcm8 Dscc1 Fancm Xrcc2 Clspn Lin9 Trpc2 Kif22 Orc1l Recql4 |
| microtubule-based process | GO:0007017 | 3.96E-04 | Birc5 Kifap3 Ptk2 Tube1 Sac3d1 Kif4 3000004C01Rik Ndc80 Nuf2 Espl1 Kif2c Kif22 Tubb6 |
| DNA repair | GO:0006281 | 6.13E-04 | Rad51ap1 Fancb 2010301N04Rik Brca2 Esco2 Mpg Xrcc3 Eme1 Fancm Clspn Xrcc2 Kif22 Trpc2 |
| response to DNA damage stimulus | GO:0006974 | 0.001586809 | Rad51ap1 Fancb 2010301N04Rik Nupr1 Brca2 Esco2 Mpg Xrcc3 Eme1 Fancm Xrcc2 Clspn Kif22 Trpc2 |
| cellular response to stress | GO:0033554 | 0.012783799 | Rad51ap1 Mapkbp1 Fancb 2010301N04Rik Nupr1 Esco2 Brca2 Mpg Xrcc3 Eme1 Fancm Xrcc2 Clspn Kif22 Trpc2 |
| tRNA metabolic process | GO:0006399 | 0.016585724 | Aars2 Tarsl2 Cdk5rap1 Trmu Rpp38 Adat1 Gtpbp3 Tsen2 |
| regulation of Rab protein signal transduction | GO:0032483 | 0.048163285 | Sgsm3 Tbc1d7 Wdr67 Sgsm2 Tbc1d19 |
| regulation of Rab GTPase activity | GO:0032313 | 0.048163285 | Sgsm3 Tbc1d7 Wdr67 Sgsm2 Tbc1d19 |
