# Supplementary material for: CD28 Costimulation Regulates Genome-Wide Effects on Alternative Splicing
Source: PLoS One. 2012 Jun 29;7(6):e40032. doi: 10.1371/journal.pone.0040032 (PMC3386953; doi:10.1371/journal.pone.0040032)
Supplement: Table S3 — All genes with greater than 1.4 fold changes in transcript-level expression in TCR-activated compared to naïve T cells and TCR/CD28 activated compared to naïve T cells or TCR/CD28 activated compared to TCR-activated T cells. (DOC) [file pone.0040032.s006.doc]

**Table S3**

**1,481 genes with greater than 2-fold change in transcript-level expression: TCR activation as compared to naïve T cells**

| **TCID** | **Symbol** | **Fold change** |
| --- | --- | --- |
| 6896860 | Spry1 | 27.54 |
| 6978332 | Ccl22 | 19.97 |
| 6958193 | Bcat1 | 16.89 |
| 6805825 | Irf4 | 16.68 |
| 6954415 | Il12rb2 | 14.72 |
| 6790294 | Ccl3 | 13.68 |
| 6778391 | Lif | 13.27 |
| 6957144 | Lag3 | 12.96 |
| 6951525 | Asns | 12.28 |
| 6913315 | Nr4a3 | 11.69 |
| 6791171 | Tbx21 | 11.60 |
| 6788329 | Csf2 | 10.90 |
| 6754536 | Tnfsf4 | 10.50 |
| 6925149 | Mfsd2 | 10.33 |
| 6975335 | Dusp4 | 9.91 |
| 6747972 | Il17a | 9.80 |
| 6904309 | Il2 | 9.35 |
| 6754138 | Rgs16 | 9.24 |
| 6994666 | Chek1 | 9.00 |
| 6875038 | Il2ra | 8.99 |
| 6790199 | Ccl1 | 8.60 |
| 6935370 | Fscn1 | 8.49 |
| 6849595 | Cdkn1a | 8.35 |
| 6810697 | Akr1c18 | 8.14 |
| 6753400 | Lad1 | 8.04 |
| 6899034 | Crabp2 | 7.85 |
| 6924813 | Hpdl | 7.64 |
| 6889357 | Prrg4 | 7.52 |
| 6886908 | Nr4a2 | 7.26 |
| 6786473 | Slc1a4 | 7.25 |
| 6804686 | Idi2 | 7.09 |
| 6871768 | Psat1 | 6.97 |
| 7018279 | Spin4 | 6.93 |
| 6901957 | Gbp2 | 6.88 |
| 6883000 | Mybl2 | 6.68 |
| 6788333 | Il3 | 6.66 |
| 6989438 | Sema7a | 6.63 |
| 6868055 | A430093F15Rik | 6.60 |
| 6916663 | Slc6a9 | 6.57 |
| 6765235 | Dtl | 6.55 |
| 6851232 | Uhrf1 | 6.46 |
| 6901944 | Gbp5 | 6.45 |
| 6871062 | Npas4 | 6.44 |
| 6916775 | Hivep3 | 6.41 |
| 6965072 | Utf1 | 6.29 |
| 6776185 | Socs2 | 6.21 |
| 6948913 | Bhlhb2 | 6.14 |
| 6886799 | Idi1 | 5.92 |
| 6964517 | Tacc2 | 5.72 |
| 6845978 | Cd200 | 5.70 |
| 6765218 | Atf3 | 5.67 |
| 6771052 | Ifng | 5.63 |
| 6957145 | Ptms | 5.60 |
| 6946749 | Tnip3 | 5.59 |
| 6918900 | Tnfrsf9 | 5.58 |
| 6788791 | Shmt1 | 5.56 |
| 6784054 | Cdc6 | 5.56 |
| 6976002 | Dctd | 5.55 |
| 7018524 | Slc7a3 | 5.50 |
| 6824880 | Gzmb | 5.48 |
| 6933422 | Pole | 5.36 |
| 6977042 | Il12rb1 | 5.27 |
| 6783035 | Ccl4 | 5.25 |
| 6757278 | Il17f | 5.24 |
| 6763731 | Xcl1 | 5.18 |
| 6954982 | Hk2 | 5.15 |
| 6880497 | Rad51 | 5.12 |
| 6777955 | Shmt2 | 5.12 |
| 6751103 | Ccl20 | 5.11 |
| 6957465 | Csda | 5.10 |
| 6876109 | Freq | 5.02 |
| 6768232 | Ppa1 | 4.97 |
| 6880703 | AA467197 | 4.96 |
| 6869577 | Hells | 4.94 |
| 6833311 | Nr4a1 | 4.93 |
| 6940872 | Gfi1 | 4.92 |
| 6985848 | Gins2 | 4.90 |
| 6844253 | Sdf2l1 | 4.90 |
| 6806036 | Serpinb9 | 4.84 |
| 6790317 | Dusp14 | 4.81 |
| 6789977 | Tmem97 | 4.75 |
| 6901597 | Nhedc2 | 4.71 |
| 6939990 | Cxcl10 | 4.71 |
| 6878053 | Cdca7 | 4.70 |
| 6769891 | Fdps | 4.62 |
| 6919209 | Tnfrsf4 | 4.54 |
| 6786044 | Fignl1 | 4.53 |
| 6785684 | Nefh | 4.52 |
| 6803598 | Wars | 4.46 |
| 6830927 | Myc | 4.44 |
| 6936690 | Pus7 | 4.43 |
| 6791174 | Tbkbp1 | 4.41 |
| 6935927 | Cyp51 | 4.38 |
| 6957133 | Tpi1 | 4.36 |
| 6755387 | Exo1 | 4.32 |
| 6873217 | Got1 | 4.30 |
| 6976520 | Sh3rf1 | 4.29 |
| 6985900 | Slc7a5 | 4.28 |
| 6836959 | Apol7c | 4.28 |
| 6768479 | Egr2 | 4.27 |
| 6800859 | Egln3 | 4.25 |
| 6855087 | Tnf | 4.24 |
| 6994830 | Gramd1b | 4.22 |
| 6918720 | Srm | 4.19 |
| 6917301 | Marcksl1 | 4.18 |
| 6765153 | Smyd2 | 4.18 |
| 6916190 | Orc1l | 4.14 |
| 6926072 | Clic4 | 4.14 |
| 6769255 | Gadd45b | 4.14 |
| 6913080 | Melk | 4.13 |
| 6772594 | Myb | 4.13 |
| 6757282 | Mcm3 | 4.12 |
| 6894454 | Phgdh | 4.12 |
| 6982999 | 1810029B16Rik | 4.12 |
| 6748883 | Il1r2 | 4.08 |
| 6941883 | Rilpl2 | 4.07 |
| 6911213 | Fdps | 4.06 |
| 6768897 | Ndg2 | 4.05 |
| 6849091 | Zfp52 | 4.03 |
| 6947553 | Smyd5 | 3.98 |
| 6884441 | Mcm10 | 3.98 |
| 6855615 | Slc29a1 | 3.93 |
| 6977261 | Mcm5 | 3.93 |
| 6800229 | Scin | 3.92 |
| 6963418 | Wee1 | 3.91 |
| 6990216 | Anxa2 | 3.88 |
| 6793677 | Odc1 | 3.88 |
| 6974850 | Eif4ebp1 | 3.86 |
| 6876209 | St6galnac4 | 3.84 |
| 6782679 | Tlcd1 | 3.84 |
| 6815555 | Cenph | 3.79 |
| 7009795 | Pim2 | 3.78 |
| 7013857 | Cenpi | 3.76 |
| 6851309 | Tnfsf9 | 3.75 |
| 6871476 | Fen1 | 3.72 |
| 6830761 | Sqle | 3.70 |
| 6780430 | Il12b | 3.68 |
| 6792792 | Pycr1 | 3.67 |
| 7000876 | Il22 | 3.66 |
| 6882189 | Gins1 | 3.66 |
| 6992209 | Cish | 3.65 |
| 6768951 | Slc19a1 | 3.64 |
| 6778043 | Pa2g4 | 3.64 |
| 6844598 | Rfc4 | 3.63 |
| 6941040 | Noc4l | 3.61 |
| 6843923 | Litaf | 3.60 |
| 6989974 | 2810417H13Rik | 3.60 |
| 6758743 | Hspd1 | 3.59 |
| 6849098 | BC049807 | 3.59 |
| 6925054 | Ctps | 3.57 |
| 6759642 | Mreg | 3.56 |
| 6806038 | Serpinb9b | 3.55 |
| 6991358 | Plscr1 | 3.54 |
| 6846463 | 2610528E23Rik | 3.54 |
| 6883132 | Cd40 | 3.52 |
| 6764007 | Hsd17b7 | 3.52 |
| 6861308 | Iigp1 | 3.51 |
| 6832530 | Creld2 | 3.50 |
| 6845274 | Umps | 3.50 |
| 6905405 | Gpr171 | 3.50 |
| 6778278 | Eif4enif1 | 3.49 |
| 6769262 | Thop1 | 3.49 |
| 6985659 | Gcsh | 3.49 |
| 6996267 | Zwilch | 3.47 |
| 6844359 | Cdc45l | 3.46 |
| 6840579 | Tfrc | 3.46 |
| 6869032 | Cd274 | 3.45 |
| 6988635 | Hyou1 | 3.44 |
| 6789474 | C1qbp | 3.43 |
| 6895672 | Fabp5 | 3.42 |
| 6924763 | Rad54l | 3.42 |
| 6808948 | Dhfr | 3.42 |
| 6760774 | Pask | 3.42 |
| 6970839 | Coq7 | 3.39 |
| 7015392 | 2010204K13Rik | 3.38 |
| 6977814 | Gpt2 | 3.37 |
| 6917512 | Rcc1 | 3.36 |
| 6943195 | Slc7a1 | 3.36 |
| 6855088 | Lta | 3.35 |
| 6851204 | Chaf1a | 3.35 |
| 6929671 | Cad | 3.35 |
| 6817396 | A430108C13Rik | 3.35 |
| 6989874 | Tipin | 3.35 |
| 6919212 | Tnfrsf18 | 3.34 |
| 7005797 | Hmgcs1 | 3.33 |
| 6892180 | BB166591 | 3.32 |
| 6979123 | Aars | 3.31 |
| 6789369 | Rai12 | 3.31 |
| 6932517 | Sept11 | 3.31 |
| 6904330 | Cetn4 | 3.29 |
| 6749911 | Nrp2 | 3.27 |
| 7024174 | Cenpm | 3.27 |
| 6818950 | Apex1 | 3.27 |
| 6840284 | Ccdc50 | 3.27 |
| 6933598 | Ung | 3.27 |
| 6768928 | Lss | 3.26 |
| 6906840 | Cks1b | 3.25 |
| 6771533 | Mettl1 | 3.25 |
| 6825302 | Fdft1 | 3.25 |
| 6796164 | Mthfd1 | 3.25 |
| 6813326 | D13Wsu177e | 3.25 |
| 6860635 | Eif1a | 3.25 |
| 6953331 | Ezh2 | 3.23 |
| 6962107 | Sh3gl3 | 3.23 |
| 6915843 | Ak3l1 | 3.23 |
| 6919012 | Acot7 | 3.21 |
| 6810166 | Plk2 | 3.20 |
| 6929125 | AI847670 | 3.20 |
| 6950345 | Bcl2l14 | 3.20 |
| 6780863 | Nola2 | 3.19 |
| 6950160 | Gabarapl1 | 3.19 |
| 6870063 | Nfkb2 | 3.19 |
| 6941029 | Pxmp2 | 3.18 |
| 6900180 | Slc16a1 | 3.18 |
| 6858816 | Snrpd1 | 3.18 |
| 6802727 | Dio2 | 3.17 |
| 6792485 | Galk1 | 3.17 |
| 6943448 | Rfc3 | 3.16 |
| 6749773 | Nol5 | 3.16 |
| 6822191 | Ipo5 | 3.16 |
| 6790648 | Akap1 | 3.16 |
| 6890715 | Bub1 | 3.16 |
| 6941768 | Fbxl10 | 3.15 |
| 6815305 | Hmgcr | 3.15 |
| 6946102 | D330028D13Rik | 3.15 |
| 6795780 | Klhdc2 | 3.14 |
| 6793672 | Pdia6 | 3.14 |
| 6782830 | Cdk5r1 | 3.14 |
| 6881340 | Mcm8 | 3.13 |
| 6872916 | Ide | 3.13 |
| 6854386 | Tbl3 | 3.13 |
| 6955025 | Mthfd2 | 3.12 |
| 6909304 | Nola1 | 3.12 |
| 6860670 | Hspe1 | 3.10 |
| 6957252 | Rad51ap1 | 3.10 |
| 6771620 | Prim1 | 3.09 |
| 6766381 | 2610016C23Rik | 3.09 |
| 6878012 | Hat1 | 3.09 |
| 6972410 | Cars | 3.08 |
| 6928457 | Cdk6 | 3.08 |
| 6777929 | Mars | 3.08 |
| 6786262 | Pno1 | 3.07 |
| 6765734 | Zc3h12d | 3.07 |
| 6756474 | A130010J15Rik | 3.07 |
| 6850705 | BC048355 | 3.07 |
| 6967013 | Grwd1 | 3.06 |
| 7019532 | Armcx6 | 3.06 |
| 6762017 | Mcm6 | 3.06 |
| 6863002 | Crem | 3.05 |
| 6823696 | Gnl3 | 3.03 |
| 6762796 | Rgs1 | 3.03 |
| 6755207 | Slamf1 | 3.03 |
| 6979559 | Irf8 | 3.03 |
| 6896997 | Hspa4l | 3.03 |
| 6916954 | Pabpc4 | 3.02 |
| 6942669 | Centa1 | 3.02 |
| 6790902 | Nme1 | 3.01 |
| 6892325 | Eif2s2 | 3.01 |
| 6881897 | Nat5 | 3.00 |
| 6924832 | Plk3 | 3.00 |
| 6966233 | Mif | 3.00 |
| 6884351 | Suv39h2 | 2.99 |
| 7011263 | Phf6 | 2.99 |
| 6974156 | Tfdp1 | 2.98 |
| 6955543 | Chchd4 | 2.98 |
| 6763682 | BC055324 | 2.98 |
| 6961279 | Igf1r | 2.97 |
| 6972882 | Prpf31 | 2.97 |
| 6992229 | Ifrd2 | 2.96 |
| 6930074 | Lyar | 2.96 |
| 6926344 | Mrto4 | 2.96 |
| 6806034 | Serpinb6b | 2.95 |
| 6763853 | Pogk | 2.95 |
| 6927967 | Slc31a1 | 2.95 |
| 6947894 | Txnrd3 | 2.95 |
| 6943485 | Lyar | 2.95 |
| 6955381 | Mcm2 | 2.94 |
| 6951440 | Slc25a13 | 2.94 |
| 6939987 | Cxcl9 | 2.94 |
| 6926976 | Slc25a33 | 2.94 |
| 6901962 | Ccbl2 | 2.94 |
| 6794293 | Nampt | 2.93 |
| 6966166 | Nfkbib | 2.93 |
| 6955228 | Anxa4 | 2.93 |
| 6880322 | Spred1 | 2.92 |
| 6837361 | Cenpm | 2.91 |
| 6869761 | Pgam1 | 2.91 |
| 6864565 | Hspa9 | 2.91 |
| 6899252 | Pmvk | 2.90 |
| 6857022 | Tgif1 | 2.90 |
| 6892496 | Dsn1 | 2.90 |
| 6810782 | Pfkp | 2.90 |
| 6782248 | Mybbp1a | 2.88 |
| 6762679 | Ruvbl1 | 2.88 |
| 6799064 | Ddx1 | 2.88 |
| 6785750 | Pold2 | 2.87 |
| 6917283 | Yars | 2.87 |
| 6961932 | 5730590G19Rik | 2.87 |
| 6791298 | Top2a | 2.87 |
| 6769343 | Tdg | 2.87 |
| 6844327 | Ranbp1 | 2.87 |
| 6829667 | Pop1 | 2.87 |
| 6961201 | Snrpa1 | 2.86 |
| 6975913 | Casp3 | 2.86 |
| 6946396 | Gars | 2.86 |
| 7002980 | Bcl2a1b | 2.86 |
| 6964253 | Hirip3 | 2.86 |
| 6836767 | Top1mt | 2.86 |
| 6900456 | Vav3 | 2.85 |
| 6985117 | Psmd7 | 2.85 |
| 6798403 | Ncapg2 | 2.85 |
| 6787896 | Irgm | 2.85 |
| 6949849 | Nol1 | 2.85 |
| 6771920 | Mthfd1l | 2.84 |
| 6990067 | Rps27l | 2.84 |
| 6769357 | Txnrd1 | 2.84 |
| 6927291 | Atad3a | 2.84 |
| 6792822 | Fasn | 2.83 |
| 6911682 | Ccne2 | 2.83 |
| 6936981 | Tyms | 2.83 |
| 6957462 | Magohb | 2.82 |
| 6928741 | Abcb1b | 2.82 |
| 6865022 | Lars | 2.82 |
| 6947760 | Rpn1 | 2.81 |
| 6792031 | ENSMUSG00000075466 | 2.81 |
| 6765982 | Timm8a1 | 2.81 |
| 6911719 | E130016E03Rik | 2.81 |
| 6916089 | Dhcr24 | 2.81 |
| 6864444 | Stard4 | 2.80 |
| 6992178 | Rrp9 | 2.80 |
| 6992349 | Impdh2 | 2.80 |
| 6875039 | Il15ra | 2.79 |
| 6796048 | Hif1a | 2.79 |
| 6885626 | Gtf3c5 | 2.79 |
| 6985009 | Ddx28 | 2.79 |
| 6812518 | Eef1e1 | 2.79 |
| 6929510 | Insig1 | 2.78 |
| 6791504 | Brca1 | 2.78 |
| 6764457 | Adss | 2.78 |
| 6913962 | Slc31a1 | 2.77 |
| 6985106 | Nqo1 | 2.77 |
| 6918699 | Mad2l2 | 2.76 |
| 6911009 | Psma5 | 2.76 |
| 6871270 | Fkbp2 | 2.76 |
| 6843951 | Rsl1d1 | 2.75 |
| 7018847 | Itm2a | 2.75 |
| 6900383 | Psma5 | 2.75 |
| 6824829 | Ipo4 | 2.75 |
| 6997349 | Hmgn3 | 2.74 |
| 6963049 | Rrm1 | 2.73 |
| 6789941 | Nek8 | 2.73 |
| 6966265 | Tbcb | 2.73 |
| 7019517 | Timm8a1 | 2.73 |
| 6966983 | Ruvbl2 | 2.72 |
| 6826927 | Dis3 | 2.72 |
| 6992306 | Gmppb | 2.72 |
| 6783909 | Mrpl45 | 2.72 |
| 6882232 | Fkbp1a | 2.72 |
| 6844310 | Slc25a1 | 2.71 |
| 6988624 | Dpagt1 | 2.70 |
| 6848553 | Tagap1 | 2.70 |
| 6989399 | Fabp5 | 2.69 |
| 6773537 | Tdg | 2.69 |
| 6867830 | Cdca5 | 2.69 |
| 6982921 | Sc4mol | 2.68 |
| 6930484 | Lap3 | 2.68 |
| 6796799 | 1810035L17Rik | 2.68 |
| 6770013 | Eea1 | 2.68 |
| 6763572 | Fasl | 2.68 |
| 6869021 | Rcl1 | 2.67 |
| 6791978 | Kpna2 | 2.67 |
| 6969429 | 4632434I11Rik | 2.67 |
| 6869537 | Cep55 | 2.67 |
| 6765719 | Nup43 | 2.67 |
| 6866068 | Txnl1 | 2.67 |
| 6919741 | ENSMUSG00000073995 | 2.66 |
| 6774794 | Cdc2a | 2.66 |
| 6921130 | Stoml2 | 2.66 |
| 6835415 | Nudcd1 | 2.66 |
| 6855772 | Bysl | 2.66 |
| 6892139 | Trib3 | 2.65 |
| 6980158 | Shcbp1 | 2.65 |
| 6870027 | Nolc1 | 2.65 |
| 6990948 | Ttk | 2.65 |
| 6843207 | Mrps6 | 2.64 |
| 6866110 | Amd1 | 2.64 |
| 6771619 | Snrpd2 | 2.64 |
| 6978935 | Cirh1a | 2.64 |
| 6896852 | Bbs12 | 2.64 |
| 6836839 | Slc39a4 | 2.63 |
| 6753014 | Il10 | 2.63 |
| 6801488 | L2hgdh | 2.63 |
| 6793674 | Nol10 | 2.62 |
| 6874683 | Nudt5 | 2.62 |
| 6934645 | 2410018M08Rik | 2.62 |
| 6770722 | Phlda1 | 2.62 |
| 6873154 | Rrp12 | 2.61 |
| 6867925 | 5730596K20Rik | 2.61 |
| 6852475 | Gemin6 | 2.61 |
| 7013187 | Pgk1 | 2.61 |
| 6764956 | Rrp15 | 2.61 |
| 6943310 | Hsph1 | 2.61 |
| 6824325 | Wdhd1 | 2.61 |
| 6849626 | Pim1 | 2.60 |
| 6836298 | Snrpc | 2.60 |
| 6984573 | Got2 | 2.60 |
| 6851191 | Ebi3 | 2.60 |
| 6807154 | Sfxn1 | 2.59 |
| 6993098 | Kif15 | 2.59 |
| 6856096 | Sgol1 | 2.59 |
| 6749569 | Hspe1 | 2.59 |
| 6998596 | Armet | 2.59 |
| 6791465 | Psmc3ip | 2.59 |
| 6977687 | Ddx39 | 2.58 |
| 6998584 | Abhd14a | 2.58 |
| 6782564 | Hsp90aa1 | 2.58 |
| 6985427 | Kars | 2.58 |
| 6848504 | Snx9 | 2.57 |
| 6929849 | Sh3bp2 | 2.57 |
| 6835776 | Dscc1 | 2.57 |
| 6807998 | Nsun2 | 2.57 |
| 6781970 | Aurkb | 2.56 |
| 6966922 | Prmt1 | 2.56 |
| 6772217 | Ltv1 | 2.55 |
| 6892393 | BC029722 | 2.55 |
| 6806963 | Iars | 2.55 |
| 6934652 | Tpst1 | 2.55 |
| 6957640 | Dusp16 | 2.55 |
| 6960301 | Il4i1 | 2.55 |
| 6864564 | Etf1 | 2.55 |
| 6925910 | Nudc | 2.55 |
| 6912179 | F730047E07Rik | 2.55 |
| 6944952 | Snd1 | 2.55 |
| 6930838 | Pi4k2b | 2.55 |
| 6966298 | Zbtb32 | 2.54 |
| 6855123 | Ddr1 | 2.54 |
| 6834013 | Tars | 2.54 |
| 6871519 | Ccdc86 | 2.54 |
| 6985006 | Slc12a4 | 2.54 |
| 6758361 | Kdelc1 | 2.53 |
| 6929591 | ENSMUSG00000073107 | 2.53 |
| 6782411 | Tsr1 | 2.53 |
| 6845382 | 2310056P07Rik | 2.53 |
| 6778595 | Ramp3 | 2.53 |
| 6873069 | Aldh18a1 | 2.53 |
| 6968533 | Tdg | 2.52 |
| 6878710 | Timm10 | 2.52 |
| 6852225 | Ttc27 | 2.52 |
| 6942604 | Mcm7 | 2.51 |
| 6775870 | Utp20 | 2.51 |
| 6752409 | Mki67ip | 2.51 |
| 6754696 | Slc19a2 | 2.51 |
| 6985361 | Mlkl | 2.50 |
| 6881115 | Rpo1 | 2.50 |
| 6803780 | 2810452K22Rik | 2.50 |
| 6958991 | Snrpd2 | 2.50 |
| 6800233 | Ifrd1 | 2.50 |
| 6762420 | Timm17a | 2.50 |
| 6958905 | Slc1a5 | 2.49 |
| 7018582 | Ercc6l | 2.49 |
| 6994883 | Crtam | 2.49 |
| 6782224 | Txndc17 | 2.49 |
| 6892209 | Plagl2 | 2.49 |
| 6987350 | Icam1 | 2.49 |
| 6792789 | Mafg | 2.48 |
| 6763894 | Uck2 | 2.48 |
| 6855659 | Vegfa | 2.48 |
| 6978296 | Nup93 | 2.48 |
| 6867955 | Wdr74 | 2.48 |
| 6802124 | Erh | 2.47 |
| 6959004 | Ercc1 | 2.47 |
| 6783740 | Slc35b1 | 2.47 |
| 6768125 | Timm23 | 2.47 |
| 6939320 | Ppat | 2.47 |
| 6801454 | Pole2 | 2.47 |
| 6890658 | Ncaph | 2.47 |
| 6784606 | Psmc5 | 2.47 |
| 6859836 | Wdr36 | 2.47 |
| 6791900 | Ftsj3 | 2.46 |
| 6858352 | Tcp1 | 2.46 |
| 6858126 | Kpna2 | 2.46 |
| 6963017 | 3200002M19Rik | 2.46 |
| 6781248 | Igtp | 2.45 |
| 6859031 | BC003885 | 2.45 |
| 6942673 | 3110082I17Rik | 2.45 |
| 6780544 | Med7 | 2.45 |
| 6749937 | Fastkd2 | 2.45 |
| 6843208 | Slc5a3 | 2.45 |
| 6767610 | Prep | 2.45 |
| 6785774 | Ddx56 | 2.44 |
| 6899104 | Cct3 | 2.44 |
| 6783137 | Myo19 | 2.44 |
| 6863299 | Usp14 | 2.44 |
| 6944293 | Mdfic | 2.44 |
| 6785299 | Syngr2 | 2.44 |
| 6868676 | Klf9 | 2.44 |
| 7014702 | Acot10 | 2.44 |
| 7020410 | Sms | 2.44 |
| 6793255 | Wdr35 | 2.44 |
| 6875662 | Zmynd19 | 2.43 |
| 7011907 | Nsdhl | 2.43 |
| 6803759 | Hsp90aa1 | 2.43 |
| 6825216 | Kpna3 | 2.43 |
| 6912923 | Nudt2 | 2.43 |
| 6758995 | Als2 | 2.43 |
| 6836782 | Pycrl | 2.43 |
| 6819662 | Rnaseh2b | 2.43 |
| 6977016 | Isyna1 | 2.43 |
| 6845909 | Qtrtd1 | 2.43 |
| 6760777 | Hdlbp | 2.43 |
| 6985925 | Mvd | 2.43 |
| 6812983 | Tpmt | 2.42 |
| 6822772 | Psmd6 | 2.42 |
| 6967882 | Mphosph10 | 2.42 |
| 6912213 | 1110007M04Rik | 2.42 |
| 6787283 | Rars | 2.42 |
| 6971272 | Eif3c | 2.41 |
| 6775148 | Ddt | 2.41 |
| 6866233 | Lman1 | 2.41 |
| 6754027 | Niban | 2.41 |
| 6977804 | Orc6l | 2.41 |
| 6892307 | E2f1 | 2.41 |
| 6858291 | Tcp1 | 2.41 |
| 6884339 | Rpp38 | 2.41 |
| 6916483 | Stil | 2.41 |
| 6977768 | Syce2 | 2.41 |
| 6847190 | Samsn1 | 2.40 |
| 6935269 | Nudt1 | 2.40 |
| 6790475 | Brip1 | 2.40 |
| 6873025 | Noc3l | 2.40 |
| 6935116 | Cops6 | 2.40 |
| 6894152 | Psma7 | 2.40 |
| 6983950 | Neto2 | 2.40 |
| 6831891 | Maff | 2.39 |
| 7016317 | Nkrf | 2.39 |
| 6924801 | Nasp | 2.39 |
| 6782368 | 1300001I01Rik | 2.38 |
| 6860903 | Srfbp1 | 2.38 |
| 6776667 | Tmtc2 | 2.38 |
| 6790478 | Ints2 | 2.38 |
| 6815870 | Ndufaf2 | 2.38 |
| 6849845 | Rrp1b | 2.38 |
| 6781240 | Larp1 | 2.38 |
| 6942939 | Jtv1 | 2.38 |
| 6759648 | Pecr | 2.38 |
| 6963578 | Mlstd2 | 2.38 |
| 6767519 | AK122525 | 2.38 |
| 6768065 | Hsf2 | 2.37 |
| 6978869 | Nutf2 | 2.37 |
| 6905333 | Pgk1 | 2.37 |
| 6807022 | Cks2 | 2.37 |
| 6791419 | Atp5g1 | 2.37 |
| 6850071 | Rdbp | 2.37 |
| 6855145 | Mrps18b | 2.37 |
| 6859837 | Wdr36 | 2.36 |
| 7019640 | Morf4l2 | 2.36 |
| 6789805 | Gemin4 | 2.36 |
| 6784039 | Psmd3 | 2.36 |
| 6757732 | Prim2 | 2.36 |
| 6887819 | Ola1 | 2.36 |
| 6783674 | Lrrc59 | 2.36 |
| 6809812 | Cenpk | 2.36 |
| 6866116 | Nars | 2.36 |
| 6876079 | 2610205E22Rik | 2.36 |
| 6784709 | Psmd12 | 2.35 |
| 6847880 | Gart | 2.35 |
| 6839334 | Nubp1 | 2.35 |
| 6856269 | Gtf2f1 | 2.35 |
| 6766863 | Echdc1 | 2.35 |
| 6858456 | Cul2 | 2.35 |
| 6937334 | Nol14 | 2.35 |
| 6815329 | Plp2 | 2.35 |
| 6820047 | Tnfrsf10b | 2.35 |
| 6966137 | Timm50 | 2.34 |
| 6759800 | Dnpep | 2.34 |
| 6992409 | Nme6 | 2.34 |
| 6809447 | Mrps27 | 2.34 |
| 6840086 | Dnajb11 | 2.34 |
| 6888937 | Hsd17b12 | 2.34 |
| 6950394 | Gprc5a | 2.34 |
| 6907623 | Ptgfrn | 2.33 |
| 6901953 | Gbp3 | 2.33 |
| 6782141 | Eno3 | 2.33 |
| 6897002 | Plk4 | 2.33 |
| 6990859 | Irak1bp1 | 2.32 |
| 6778463 | Xbp1 | 2.32 |
| 6765716 | Lrp11 | 2.32 |
| 6871361 | Slc3a2 | 2.32 |
| 7015993 | Uxt | 2.31 |
| 6964913 | Glrx3 | 2.31 |
| 6936585 | Dnajc2 | 2.31 |
| 6833216 | Larp4 | 2.31 |
| 6840860 | Tmem39a | 2.31 |
| 6951247 | 3010003L21Rik | 2.31 |
| 6967006 | Sult2b1 | 2.31 |
| 6926655 | Tnfrsf8 | 2.31 |
| 6833972 | Bxdc2 | 2.31 |
| 6966030 | Snrpa | 2.31 |
| 6820372 | Nufip1 | 2.31 |
| 6837189 | Rps19bp1 | 2.31 |
| 6960298 | Nup62 | 2.31 |
| 6769509 | Pwp1 | 2.30 |
| 6966108 | Psmc4 | 2.30 |
| 6992414 | Cdc25a | 2.30 |
| 6995074 | Hmbs | 2.30 |
| 6844250 | 2610318N02Rik | 2.30 |
| 6865709 | Aldh7a1 | 2.30 |
| 6753710 | Uchl5 | 2.29 |
| 6837279 | St13 | 2.29 |
| 6962751 | Alg8 | 2.29 |
| 6899028 | Hdgf | 2.29 |
| 6858868 | Rbbp8 | 2.29 |
| 6911925 | Nbn | 2.29 |
| 6913777 | Akap2 | 2.29 |
| 6795233 | 1110008L16Rik | 2.29 |
| 6956964 | Cecr6 | 2.29 |
| 6780996 | Irf1 | 2.29 |
| 6917180 | Clspn | 2.28 |
| 6832079 | Adsl | 2.28 |
| 6760804 | Pdcd1 | 2.28 |
| 6987403 | Ldlr | 2.28 |
| 6881172 | Nol5a | 2.28 |
| 6867930 | Hrasls3 | 2.28 |
| 6749851 | Ctla4 | 2.28 |
| 6889301 | Nat10 | 2.27 |
| 6819737 | C230072K23 | 2.27 |
| 6837322 | Polr3h | 2.27 |
| 6926881 | Fbxo6 | 2.27 |
| 6946011 | Repin1 | 2.27 |
| 6963413 | Ipo7 | 2.26 |
| 6912499 | Mdn1 | 2.26 |
| 6950555 | Strap | 2.26 |
| 6922244 | Pole3 | 2.26 |
| 6791992 | Nol11 | 2.26 |
| 6904074 | Mrpl47 | 2.26 |
| 6789237 | Rangrf | 2.26 |
| 6844177 | Mcm4 | 2.26 |
| 6842009 | Pros1 | 2.26 |
| 6796310 | Eif2s1 | 2.26 |
| 6987343 | Ppan | 2.26 |
| 6841097 | Nat13 | 2.26 |
| 6978884 | Slc7a6 | 2.26 |
| 6929152 | Psmc2 | 2.25 |
| 6824346 | Dlg7 | 2.25 |
| 6869035 | Pdcd1lg2 | 2.25 |
| 6934584 | Ran | 2.25 |
| 6838853 | Il2rb | 2.25 |
| 6966193 | Psmd8 | 2.25 |
| 6922901 | 1810054D07Rik | 2.25 |
| 6978948 | Nip7 | 2.24 |
| 6836341 | Sla | 2.24 |
| 6894898 | Pdss1 | 2.24 |
| 6987580 | Sept7 | 2.24 |
| 6778352 | Pes1 | 2.24 |
| 6840849 | Cd80 | 2.23 |
| 6916722 | Ebna1bp2 | 2.23 |
| 6849974 | Cd320 | 2.23 |
| 6913499 | Smc2 | 2.23 |
| 6953522 | Cycs | 2.23 |
| 6880853 | Dut | 2.23 |
| 6763493 | Cacybp | 2.23 |
| 6893636 | Slmo2 | 2.23 |
| 6774404 | Ddx21 | 2.22 |
| 6985642 | Cdyl2 | 2.22 |
| 6898162 | Gfm1 | 2.22 |
| 6825544 | Cdca2 | 2.22 |
| 6802507 | Gstz1 | 2.22 |
| 6837122 | Josd1 | 2.21 |
| 6856689 | Twsg1 | 2.21 |
| 6837021 | Il2rb | 2.21 |
| 6907204 | Ecm1 | 2.21 |
| 6760915 | Hisppd1 | 2.21 |
| 6989237 | AY074887 | 2.21 |
| 6929604 | D5Wsu178e | 2.21 |
| 6951304 | Bet1 | 2.21 |
| 6768783 | Zwint | 2.21 |
| 6954935 | Mrpl19 | 2.21 |
| 6876342 | Hspa5 | 2.21 |
| 7018220 | Pola1 | 2.21 |
| 6899146 | Ssr2 | 2.21 |
| 6899025 | Sh2d2a | 2.20 |
| 6768891 | Derl3 | 2.20 |
| 6986032 | Nup133 | 2.20 |
| 6781396 | Med9 | 2.20 |
| 6792497 | Mrpl38 | 2.20 |
| 6950334 | Etv6 | 2.20 |
| 6947394 | Gcs1 | 2.20 |
| 6864709 | Hars | 2.20 |
| 6935273 | Eif3b | 2.20 |
| 6973762 | 2010309E21Rik | 2.19 |
| 6811762 | Gmnn | 2.19 |
| 6909516 | Gstcd | 2.19 |
| 6839947 | Ece2 | 2.19 |
| 6830510 | Mtbp | 2.19 |
| 6970396 | Tmem41b | 2.19 |
| 6781032 | Fnip1 | 2.19 |
| 6785943 | Polr2c | 2.19 |
| 6921124 | Vcp | 2.19 |
| 6981616 | Erh | 2.19 |
| 6934401 | Aacs | 2.19 |
| 6804849 | Heatr1 | 2.19 |
| 6917437 | Mecr | 2.18 |
| 6917270 | Ak2 | 2.18 |
| 6854393 | Fahd1 | 2.18 |
| 6815390 | Utp15 | 2.18 |
| 7011852 | Hmgb3 | 2.18 |
| 6880661 | Eif3j | 2.18 |
| 6977971 | Heatr3 | 2.18 |
| 6804608 | Pitrm1 | 2.18 |
| 6789851 | Efcab5 | 2.18 |
| 6847632 | Cct8 | 2.18 |
| 6760364 | Ncl | 2.18 |
| 6813877 | Zfp367 | 2.17 |
| 7015403 | Plp2 | 2.17 |
| 6971393 | Bcl7c | 2.17 |
| 6875197 | Arl5b | 2.17 |
| 6959397 | Fbl | 2.16 |
| 6791541 | Lsm12 | 2.16 |
| 6790231 | Nle1 | 2.16 |
| 6952766 | Creb3l2 | 2.16 |
| 6949206 | Tsen2 | 2.16 |
| 6850780 | Mrps10 | 2.16 |
| 6939986 | Sdad1 | 2.16 |
| 6765723 | Katna1 | 2.15 |
| 6913222 | Anp32b | 2.15 |
| 6868516 | 2410127L17Rik | 2.15 |
| 6760794 | Thap4 | 2.15 |
| 6885901 | Fpgs | 2.15 |
| 6901334 | Pla2g12a | 2.15 |
| 6896519 | Skil | 2.15 |
| 6966875 | Pold1 | 2.15 |
| 6917190 | Psmb2 | 2.14 |
| 6985408 | Cfdp1 | 2.14 |
| 6869414 | Pcgf5 | 2.14 |
| 6765130 | Pdcd5 | 2.14 |
| 6809524 | Smn1 | 2.14 |
| 6915504 | Ift74 | 2.14 |
| 6933602 | Mvk | 2.14 |
| 6817930 | Tkt | 2.14 |
| 6982580 | Spcs3 | 2.14 |
| 6876219 | Ptrh1 | 2.14 |
| 6848146 | Psmg1 | 2.14 |
| 6987132 | Josd3 | 2.13 |
| 6909160 | Larp7 | 2.13 |
| 6798930 | Pgk1 | 2.13 |
| 6989042 | D630004A14Rik | 2.13 |
| 6867593 | 1810055G02Rik | 2.13 |
| 6775411 | Lmnb2 | 2.13 |
| 6865108 | Eif3j | 2.13 |
| 6839607 | Abcc1 | 2.13 |
| 6850711 | Mrpl2 | 2.13 |
| 6881837 | Sec23b | 2.13 |
| 6970650 | Psma1 | 2.12 |
| 6756815 | Cops5 | 2.12 |
| 6911287 | Msto1 | 2.12 |
| 6832532 | Pim3 | 2.12 |
| 6872426 | D19Bwg1357e | 2.12 |
| 6917048 | Yrdc | 2.12 |
| 6849456 | Ergic1 | 2.12 |
| 6917069 | Gnl2 | 2.12 |
| 6921986 | Txn1 | 2.12 |
| 6839952 | Psmd2 | 2.12 |
| 6941751 | Atp2a2 | 2.12 |
| 6795025 | Hmgb1 | 2.11 |
| 6896804 | Exosc9 | 2.11 |
| 6909648 | Nfkb1 | 2.11 |
| 6756989 | Lactb2 | 2.11 |
| 6830770 | Trib1 | 2.11 |
| 6912477 | Bach2 | 2.11 |
| 6964214 | Tufm | 2.11 |
| 6941423 | Rfc5 | 2.11 |
| 6962921 | 2610209A20Rik | 2.10 |
| 6881341 | Crls1 | 2.10 |
| 6838797 | Cbx5 | 2.10 |
| 6877587 | Psmd14 | 2.10 |
| 6930864 | Rbpj | 2.10 |
| 6860001 | Ctnna1 | 2.10 |
| 6815792 | Ipo11 | 2.10 |
| 6998614 | Hemk1 | 2.10 |
| 6821431 | Uchl3 | 2.10 |
| 6884277 | Tpd52l2 | 2.10 |
| 6792779 | P4hb | 2.10 |
| 7019519 | Gla | 2.09 |
| 6934053 | C330023M02Rik | 2.09 |
| 6998583 | Acy1 | 2.09 |
| 6901055 | Sec24d | 2.09 |
| 6966600 | Ccne1 | 2.09 |
| 6839685 | Yars2 | 2.09 |
| 6850678 | Mrps18a | 2.08 |
| 6887081 | Ly75 | 2.08 |
| 6901757 | Eif4e | 2.08 |
| 6853910 | AV024533 | 2.08 |
| 6971028 | Ndufab1 | 2.08 |
| 6825696 | Polr3d | 2.08 |
| 6995393 | Rexo2 | 2.08 |
| 6783063 | Acaca | 2.08 |
| 6806893 | A830005F24Rik | 2.07 |
| 6775758 | Hsp90b1 | 2.07 |
| 7018225 | Pdk3 | 2.07 |
| 6835932 | 9130401M01Rik | 2.07 |
| 6868823 | Cycs | 2.07 |
| 6797387 | Psmc1 | 2.07 |
| 6751082 | Hrb | 2.07 |
| 6942694 | Psmg3 | 2.07 |
| 6878713 | Slc43a3 | 2.07 |
| 6913223 | Nans | 2.07 |
| 6788020 | Canx | 2.07 |
| 6879489 | Traf6 | 2.07 |
| 6909375 | 2310008M10Rik | 2.07 |
| 6775298 | Ppap2c | 2.06 |
| 6773235 | Amd1 | 2.06 |
| 6770718 | Nap1l1 | 2.06 |
| 6916127 | Tmem48 | 2.06 |
| 6844468 | Alg3 | 2.05 |
| 6873421 | Arl3 | 2.05 |
| 6941472 | Fbxw8 | 2.05 |
| 6915734 | Usp1 | 2.05 |
| 6824007 | Ghitm | 2.05 |
| 6809550 | Taf9 | 2.05 |
| 6791207 | E130012A19Rik | 2.05 |
| 6774395 | Supv3l1 | 2.05 |
| 6775206 | Adarb1 | 2.05 |
| 6890453 | Slc30a4 | 2.04 |
| 6878296 | Mtx2 | 2.04 |
| 6806544 | Hivep1 | 2.04 |
| 6750413 | Xrcc5 | 2.04 |
| 6957150 | Cops7a | 2.04 |
| 6841739 | Tomm70a | 2.04 |
| 6950413 | Emp1 | 2.04 |
| 6813246 | Nfil3 | 2.04 |
| 6893558 | Pmepa1 | 2.03 |
| 6771560 | Ddit3 | 2.03 |
| 6769461 | AI597468 | 2.03 |
| 6855801 | Foxp4 | 2.03 |
| 6906807 | Msto1 | 2.03 |
| 6942554 | Pop7 | 2.03 |
| 6989209 | Idh3a | 2.03 |
| 6782553 | Blmh | 2.03 |
| 6906564 | 9930021J17Rik | 2.03 |
| 6798640 | Ubxd4 | 2.03 |
| 6987638 | Thyn1 | 2.03 |
| 6799800 | Acp1 | 2.02 |
| 6782704 | Poldip2 | 2.02 |
| 6883273 | Ddx27 | 2.02 |
| 6808680 | Ccnh | 2.02 |
| 6785808 | Tbrg4 | 2.02 |
| 6833100 | Cacnb3 | 2.02 |
| 6839754 | Smpd4 | 2.02 |
| 6780933 | Vdac1 | 2.01 |
| 6996440 | Rab8b | 2.01 |
| 6861722 | Psmg2 | 2.01 |
| 6788264 | Hspa4 | 2.01 |
| 6904367 | EG381438 | 2.01 |
| 6987378 | Ilf3 | 2.00 |
| 7011603 | Eif4e | 2.00 |
| 6935756 | Brca2 | 2.00 |
| 6817944 | Sfmbt1 | 2.00 |
| 6931217 | Klf3 | 0.50 |
| 6809030 | Arsb | 0.50 |
| 6758861 | Hsfy2 | 0.50 |
| 6849481 | Itpr3 | 0.50 |
| 6960157 | BC043301 | 0.50 |
| 6880718 | Sqrdl | 0.50 |
| 6815522 | Naip2 | 0.50 |
| 6894907 | Camk1d | 0.50 |
| 6857415 | Fez2 | 0.50 |
| 6868098 | Ms4a6c | 0.50 |
| 6753068 | 5430435G22Rik | 0.50 |
| 6931529 | Atp10d | 0.49 |
| 6978937 | Sntb2 | 0.49 |
| 6935082 | Lrch4 | 0.49 |
| 6978843 | 2310066E14Rik | 0.49 |
| 6785139 | Armc7 | 0.49 |
| 6849991 | Rgl2 | 0.49 |
| 6755210 | Cd84 | 0.49 |
| 6763706 | Atp1b1 | 0.49 |
| 6965314 | Tspan32 | 0.49 |
| 6781689 | Hs3st3a1 | 0.49 |
| 6845079 | Apod | 0.49 |
| 6838808 | Zfp385a | 0.49 |
| 6946920 | Cd8a | 0.49 |
| 6837143 | Cbx7 | 0.49 |
| 6933997 | Oas1b | 0.49 |
| 6792496 | Trim65 | 0.49 |
| 6898972 | Cd1d2 | 0.49 |
| 6933973 | Slc24a6 | 0.49 |
| 6759718 | Tns1 | 0.49 |
| 6921158 | E130306D19Rik | 0.49 |
| 6941173 | Cmklr1 | 0.49 |
| 6936088 | Rundc3b | 0.49 |
| 6767402 | Sesn1 | 0.48 |
| 6782125 | Arrb2 | 0.48 |
| 6804898 | Lyst | 0.48 |
| 6983255 | Abhd8 | 0.48 |
| 6775576 | Appl2 | 0.48 |
| 6899016 | Arhgef11 | 0.48 |
| 6908330 | Amy2 | 0.48 |
| 6782273 | Atp2a3 | 0.48 |
| 6966358 | Gramd1a | 0.48 |
| 6928740 | Abcb1a | 0.48 |
| 6815535 | Naip1 | 0.48 |
| 6755714 | Tmem63a | 0.48 |
| 6803102 | Rps6ka5 | 0.48 |
| 6921379 | Trim14 | 0.48 |
| 6978355 | Gpr97 | 0.48 |
| 6791230 | Arl5c | 0.48 |
| 6957406 | Clec7a | 0.48 |
| 6850055 | Gpsm3 | 0.48 |
| 6796158 | Syne2 | 0.48 |
| 6968314 | Mctp2 | 0.48 |
| 6969631 | Aqp11 | 0.48 |
| 6788928 | Zfp287 | 0.48 |
| 6966282 | Hcst | 0.47 |
| 6969837 | P2ry6 | 0.47 |
| 6869543 | Gpr120 | 0.47 |
| 6765276 | Gstp1 | 0.47 |
| 6988643 | Bcl9l | 0.47 |
| 6798271 | Adssl1 | 0.47 |
| 6844601 | BC106179 | 0.47 |
| 6832719 | Cntn1 | 0.47 |
| 6788393 | Ccdc69 | 0.47 |
| 6946558 | Herc3 | 0.47 |
| 6937466 | Sorcs2 | 0.47 |
| 6918349 | Dhrs3 | 0.47 |
| 6905424 | P2ry12 | 0.47 |
| 6998192 | Pik3cb | 0.47 |
| 6975307 | Tmem66 | 0.46 |
| 6866852 | Slc14a1 | 0.46 |
| 6960578 | Nav2 | 0.46 |
| 6782102 | Mgl2 | 0.46 |
| 6877441 | Tanc1 | 0.46 |
| 6857797 | Zfp36l2 | 0.46 |
| 7015398 | Ppp1r3f | 0.46 |
| 6870980 | Tbc1d10c | 0.46 |
| 6828472 | Dab2 | 0.46 |
| 6791696 | Arhgap27 | 0.46 |
| 6925165 | Macf1 | 0.46 |
| 6962880 | Arrb1 | 0.46 |
| 6880683 | Sord | 0.46 |
| 6748886 | Il1rl2 | 0.46 |
| 6791229 | Plxdc1 | 0.46 |
| 6834560 | BC052328 | 0.46 |
| 6813096 | Fgd3 | 0.46 |
| 6950391 | Cdkn1b | 0.46 |
| 6889273 | Ehf | 0.46 |
| 6783321 | Sept4 | 0.46 |
| 6987586 | Clpb | 0.46 |
| 6957365 | Klrb1b | 0.46 |
| 6940236 | Antxr2 | 0.46 |
| 6784042 | Nr1d1 | 0.46 |
| 6781214 | Galnt10 | 0.45 |
| 6792606 | Tha1 | 0.45 |
| 6937288 | Mxd4 | 0.45 |
| 6779855 | Stk10 | 0.45 |
| 6968780 | Fes | 0.45 |
| 6929655 | Khk | 0.45 |
| 6996432 | Aph1b | 0.45 |
| 6887196 | Ifih1 | 0.45 |
| 6888891 | Tspan18 | 0.45 |
| 6928742 | Abcb4 | 0.45 |
| 6771884 | Esr1 | 0.45 |
| 6993761 | Cdkn2d | 0.45 |
| 6802315 | Entpd5 | 0.45 |
| 6949865 | Tnfrsf1a | 0.45 |
| 6850763 | Trerf1 | 0.45 |
| 6980016 | Nrp1 | 0.45 |
| 6990427 | Ccpg1 | 0.45 |
| 6908348 | Amy2 | 0.45 |
| 6978695 | Cdh5 | 0.45 |
| 6782777 | Rab11fip4 | 0.45 |
| 6943142 | Flt3 | 0.44 |
| 6953956 | V1rc22 | 0.44 |
| 6875666 | Pnpla7 | 0.44 |
| 6881028 | Acoxl | 0.44 |
| 6792544 | St6galnac2 | 0.44 |
| 6799897 | Pik3cg | 0.44 |
| 6902179 | Mcoln3 | 0.44 |
| 6769272 | Matk | 0.44 |
| 6902880 | Gpr177 | 0.44 |
| 6817217 | Thrb | 0.44 |
| 6886678 | Rnd3 | 0.44 |
| 6949844 | Acrbp | 0.44 |
| 6856676 | Rab31 | 0.43 |
| 6792649 | Timp2 | 0.43 |
| 6919596 | Asph | 0.43 |
| 6771334 | Ppm1h | 0.43 |
| 6877139 | Fmnl2 | 0.43 |
| 6962027 | Zscan2 | 0.43 |
| 6993845 | Rab3d | 0.43 |
| 6770201 | Kitl | 0.43 |
| 6753417 | Kif21b | 0.43 |
| 6922541 | Megf9 | 0.43 |
| 6784363 | Adam11 | 0.43 |
| 6838565 | Pou6f1 | 0.43 |
| 6994773 | Olfr938 | 0.43 |
| 6907784 | Olfml3 | 0.43 |
| 6763208 | Mr1 | 0.43 |
| 6812212 | Serpinb6a | 0.43 |
| 6785173 | 2210020M01Rik | 0.43 |
| 6807228 | Rgs14 | 0.43 |
| 6797544 | D12Ertd647e | 0.43 |
| 6885431 | Tmem141 | 0.43 |
| 6849950 | Adamts10 | 0.43 |
| 6754798 | Creg1 | 0.42 |
| 6765327 | G0s2 | 0.42 |
| 6790244 | Slfn8 | 0.42 |
| 6813887 | Ctsl | 0.42 |
| 6789484 | Nlrp1a | 0.42 |
| 6847540 | App | 0.42 |
| 6873503 | Sh3pxd2a | 0.42 |
| 6844530 | 2510009E07Rik | 0.42 |
| 6970857 | Gprc5b | 0.42 |
| 6768867 | Ggt1 | 0.42 |
| 6845933 | Sidt1 | 0.42 |
| 6986722 | Mmp12 | 0.42 |
| 6936719 | Kcnh2 | 0.42 |
| 7017663 | Gab3 | 0.42 |
| 6962745 | Gab2 | 0.42 |
| 6999682 | Fyco1 | 0.42 |
| 6861751 | D18Ertd653e | 0.42 |
| 6783785 | Gngt2 | 0.41 |
| 6872646 | Asah2 | 0.41 |
| 6783762 | Zfp652 | 0.41 |
| 6870979 | AI790298 | 0.41 |
| 6978781 | BC015286 | 0.41 |
| 6832005 | Grap2 | 0.41 |
| 6829495 | Ctnnd2 | 0.41 |
| 6920754 | Mobkl2b | 0.41 |
| 6875421 | Msrb2 | 0.41 |
| 6958071 | St8sia1 | 0.41 |
| 6747497 | Sgk3 | 0.41 |
| 6977712 | Podnl1 | 0.41 |
| 6941637 | Tpcn1 | 0.41 |
| 6763090 | Rgl1 | 0.41 |
| 6946339 | Chn2 | 0.41 |
| 6908345 | Amy2 | 0.41 |
| 6938631 | Rell1 | 0.41 |
| 6946370 | 2410066E13Rik | 0.40 |
| 6788314 | Pdlim4 | 0.40 |
| 6996438 | Aph1c | 0.40 |
| 6977975 | Adcy7 | 0.40 |
| 6880508 | Spint1 | 0.40 |
| 6903157 | Pag1 | 0.40 |
| 6962779 | Pak1 | 0.40 |
| 6926504 | Tmem51 | 0.40 |
| 6815027 | Rasgrf2 | 0.40 |
| 6988898 | D930028F11Rik | 0.40 |
| 6785317 | Socs3 | 0.40 |
| 6792614 | Socs3 | 0.40 |
| 6993472 | Fut4 | 0.40 |
| 7017600 | L1cam | 0.40 |
| 6764211 | Cadm3 | 0.40 |
| 6849761 | Dnahc8 | 0.40 |
| 6805106 | Tcrg | 0.40 |
| 6950115 | Klrb1f | 0.40 |
| 6939931 | Btc | 0.40 |
| 6782105 | Mgl1 | 0.40 |
| 6780551 | Havcr2 | 0.40 |
| 6758704 | Pgap1 | 0.40 |
| 6791418 | Hap1 | 0.40 |
| 6872783 | Lipa | 0.40 |
| 6783882 | Sp6 | 0.40 |
| 6923694 | Dock7 | 0.39 |
| 6857183 | Xdh | 0.39 |
| 6959968 | 1600014C10Rik | 0.39 |
| 6825679 | 9930012K11Rik | 0.39 |
| 6972660 | Ncr1 | 0.39 |
| 6862922 | Cd226 | 0.39 |
| 6900975 | Arhgap29 | 0.39 |
| 6838580 | Galnt6 | 0.39 |
| 7017601 | Arhgap4 | 0.39 |
| 6941657 | Oas1a | 0.39 |
| 6871545 | Ms4a14 | 0.39 |
| 6848179 | Dscam | 0.38 |
| 6908137 | Gpsm2 | 0.38 |
| 6952284 | Grm8 | 0.38 |
| 6918125 | Padi2 | 0.38 |
| 6973679 | A430078G23Rik | 0.38 |
| 6854231 | A630033E08Rik | 0.38 |
| 6767088 | Tspyl4 | 0.38 |
| 6941146 | Adrbk2 | 0.38 |
| 6752158 | Serpinb10 | 0.38 |
| 6932336 | Afp | 0.38 |
| 6975367 | Ppp1r3b | 0.38 |
| 6850062 | Prrt1 | 0.38 |
| 7010871 | Sh2d1a | 0.38 |
| 6867618 | Aldh3b2 | 0.38 |
| 6820472 | Epsti1 | 0.38 |
| 6917656 | Paqr7 | 0.38 |
| 6960516 | Tmem86a | 0.38 |
| 6978883 | Lypla3 | 0.38 |
| 6926987 | Spsb1 | 0.38 |
| 6869324 | Ifit2 | 0.38 |
| 6835759 | Enpp2 | 0.38 |
| 6884520 | Cugbp2 | 0.37 |
| 6882352 | Hck | 0.37 |
| 6868884 | Smarca2 | 0.37 |
| 6777784 | Slc16a7 | 0.37 |
| 6849474 | Phf1 | 0.37 |
| 6810548 | Emb | 0.37 |
| 6939076 | Txk | 0.37 |
| 6762944 | Pla2g4a | 0.37 |
| 6754143 | Rnasel | 0.37 |
| 6765551 | Syne1 | 0.37 |
| 6807007 | Edg3 | 0.37 |
| 6998069 | BC043934 | 0.37 |
| 6791437 | Dhx58 | 0.37 |
| 6785079 | Ttyh2 | 0.36 |
| 6931759 | Kit | 0.36 |
| 6768207 | Prf1 | 0.36 |
| 6928939 | Hgf | 0.36 |
| 6840400 | Hes1 | 0.36 |
| 6988603 | Thy1 | 0.36 |
| 6806831 | Rnf144b | 0.36 |
| 6957437 | Klra10 | 0.36 |
| 6773174 | Dse | 0.36 |
| 6759621 | Fn1 | 0.36 |
| 6905408 | P2ry14 | 0.36 |
| 6792390 | RP23 | 0.36 |
| 6892486 | Sla2 | 0.36 |
| 6939241 | Kdr | 0.36 |
| 6964274 | AI467606 | 0.35 |
| 6908461 | S1pr1 | 0.35 |
| 6989917 | Parp16 | 0.35 |
| 6998434 | Acpp | 0.35 |
| 6916540 | Pik3r3 | 0.35 |
| 6954988 | Sema4f | 0.35 |
| 6870958 | Aldh3b1 | 0.35 |
| 6818956 | Ang | 0.35 |
| 6878448 | Itga4 | 0.35 |
| 6794552 | Tmem195 | 0.35 |
| 6916023 | Ppap2b | 0.35 |
| 6810063 | Elovl7 | 0.35 |
| 6805108 | Naip3 | 0.35 |
| 6875592 | Apbb1ip | 0.35 |
| 6983799 | Cd97 | 0.35 |
| 6791063 | Abi3 | 0.35 |
| 6781104 | Gm2a | 0.35 |
| 6915619 | Nfia | 0.35 |
| 6942491 | Orai2 | 0.35 |
| 6957443 | Klra10 | 0.35 |
| 6992994 | Vipr1 | 0.34 |
| 6958984 | Dmpk | 0.34 |
| 6881895 | BC039771 | 0.34 |
| 6784527 | Itgb3 | 0.34 |
| 6960404 | Dbp | 0.34 |
| 6995899 | Scamp5 | 0.34 |
| 6907941 | Bclp2 | 0.34 |
| 6964247 | Tbx6 | 0.34 |
| 6896032 | Cp | 0.34 |
| 6959474 | Rasgrp4 | 0.34 |
| 6931107 | G6pd2 | 0.34 |
| 7010645 | Il13ra1 | 0.34 |
| 6965984 | Cyp2s1 | 0.34 |
| 6789325 | Cd68 | 0.33 |
| 6987924 | Grit | 0.33 |
| 6781925 | Pik3r5 | 0.33 |
| 6850552 | Enpp5 | 0.33 |
| 6855825 | A530064D06Rik | 0.33 |
| 6977648 | Tbc1d9 | 0.33 |
| 6805180 | Aoah | 0.33 |
| 6906635 | Cd1d1 | 0.33 |
| 6841140 | Cd200r4 | 0.33 |
| 6816247 | Itga2 | 0.33 |
| 6837375 | Cyp2d22 | 0.33 |
| 6817903 | Chdh | 0.32 |
| 6845366 | Dirc2 | 0.32 |
| 6857639 | AI605517 | 0.32 |
| 6994927 | Sorl1 | 0.32 |
| 6836325 | Tmem71 | 0.32 |
| 6871598 | Olfr1423 | 0.32 |
| 6900287 | ENSMUSG00000074335 | 0.32 |
| 6939985 | Naaa | 0.32 |
| 6957432 | Klra17 | 0.32 |
| 6820219 | P2ry5 | 0.32 |
| 6899374 | S100a6 | 0.32 |
| 6789544 | Spns3 | 0.32 |
| 6957428 | Klri2 | 0.32 |
| 6822443 | Dnase1l3 | 0.32 |
| 6976765 | March1 | 0.32 |
| 6784062 | Igfbp4 | 0.32 |
| 6841201 | Gcet2 | 0.32 |
| 6957442 | Klra10 | 0.32 |
| 6897845 | Sucnr1 | 0.32 |
| 6866021 | Adrb2 | 0.31 |
| 6936076 | Adam22 | 0.31 |
| 6933627 | Oasl1 | 0.31 |
| 6815523 | Naip5 | 0.31 |
| 6774384 | Tspan15 | 0.31 |
| 6785384 | Rnf213 | 0.31 |
| 6969974 | Olfr604 | 0.31 |
| 6828480 | Fyb | 0.31 |
| 6818915 | Tlr11 | 0.31 |
| 6781368 | Nlrp3 | 0.31 |
| 6968735 | Anpep | 0.31 |
| 6949732 | Clec4b1 | 0.31 |
| 6767468 | Scml4 | 0.30 |
| 6941649 | Oas3 | 0.30 |
| 6998094 | Acpl2 | 0.30 |
| 6915818 | Raver2 | 0.30 |
| 6954269 | Ptgds2 | 0.30 |
| 6763146 | Npl | 0.30 |
| 6926023 | Man1c1 | 0.30 |
| 6815529 | Birc1f | 0.30 |
| 6913193 | 1300002K09Rik | 0.30 |
| 6782451 | Slc43a2 | 0.30 |
| 6880776 | Sema6d | 0.30 |
| 6916937 | Mycl1 | 0.30 |
| 6892699 | Mafb | 0.30 |
| 6917389 | Sdc3 | 0.30 |
| 6886039 | Stom | 0.30 |
| 6828326 | Sepp1 | 0.30 |
| 6789329 | Tnfsf12 | 0.30 |
| 6808339 | Mctp1 | 0.30 |
| 6860929 | Snx24 | 0.30 |
| 6957025 | Klrg1 | 0.30 |
| 6889737 | Olfr1309 | 0.30 |
| 6950582 | Mgst1 | 0.30 |
| 6996678 | Aqp9 | 0.30 |
| 6963006 | Pde2a | 0.30 |
| 6833232 | ENSMUSG00000058057 | 0.30 |
| 6869334 | Ifit1 | 0.30 |
| 6852181 | Ehd3 | 0.29 |
| 6778284 | Pik3ip1 | 0.29 |
| 6980103 | Cd209b | 0.29 |
| 6818104 | 8030431A06Rik | 0.29 |
| 6854310 | Tmprss8 | 0.29 |
| 6890448 | Gatm | 0.29 |
| 6940841 | Tgfbr3 | 0.29 |
| 6782979 | Slfn5 | 0.29 |
| 6782808 | Centa2 | 0.28 |
| 6751623 | Gpr35 | 0.28 |
| 6890981 | Siglec1 | 0.28 |
| 6953800 | Nod1 | 0.28 |
| 6806701 | Mylip | 0.28 |
| 6949856 | Vamp1 | 0.28 |
| 6906749 | Sema4a | 0.28 |
| 6976901 | Lpl | 0.28 |
| 6762024 | Cxcr4 | 0.28 |
| 6982102 | Tlr3 | 0.28 |
| 6833305 | Acvrl1 | 0.28 |
| 6969972 | Olfr601 | 0.28 |
| 6837787 | Plxnb2 | 0.28 |
| 6933441 | A630023P12Rik | 0.28 |
| 6952926 | Parp12 | 0.28 |
| 6808221 | Slc12a7 | 0.28 |
| 6965609 | Gpr77 | 0.27 |
| 6843601 | Mefv | 0.27 |
| 6933812 | Tesc | 0.27 |
| 6762429 | Nav1 | 0.27 |
| 6870580 | Tcf7l2 | 0.27 |
| 6892899 | Slpi | 0.27 |
| 6907262 | Fcgr1 | 0.27 |
| 6791233 | Cacnb1 | 0.27 |
| 6899760 | Txnip | 0.27 |
| 6849891 | Cyp4f16 | 0.27 |
| 6776404 | Phxr2 | 0.26 |
| 6811368 | A530099J19Rik | 0.26 |
| 6943476 | Rbm47 | 0.26 |
| 6828522 | Lifr | 0.26 |
| 6935970 | Pftk1 | 0.26 |
| 6775762 | Stab2 | 0.26 |
| 6844210 | Fgd4 | 0.26 |
| 6762784 | Rgs2 | 0.26 |
| 6784765 | Axin2 | 0.26 |
| 6885855 | A130092J06Rik | 0.26 |
| 6768076 | Smpdl3a | 0.26 |
| 6892032 | Acss1 | 0.26 |
| 6782579 | 1300007F04Rik | 0.26 |
| 6892964 | Pltp | 0.25 |
| 6965901 | Atp1a3 | 0.25 |
| 6998893 | Tmie | 0.25 |
| 6791422 | Jup | 0.25 |
| 6753067 | Ctse | 0.25 |
| 6841136 | Cd200r1 | 0.25 |
| 6761701 | Marco | 0.25 |
| 6764040 | Fcgr3 | 0.25 |
| 6963456 | Ampd3 | 0.25 |
| 6823653 | Il17rb | 0.25 |
| 6782422 | Rtn4rl1 | 0.25 |
| 6792373 | AF251705 | 0.25 |
| 6793961 | Cmpk2 | 0.25 |
| 6949202 | Pparg | 0.25 |
| 6811689 | Hist1h2ac | 0.24 |
| 7011413 | Cd40lg | 0.24 |
| 6988855 | Cadm1 | 0.24 |
| 6824728 | Slc7a7 | 0.24 |
| 6774264 | Ddit4 | 0.24 |
| 6816160 | Gzma | 0.24 |
| 6863783 | B4galt6 | 0.24 |
| 6850831 | A530064D06Rik | 0.24 |
| 6769597 | Igf1 | 0.24 |
| 6899372 | S100a4 | 0.24 |
| 6779827 | Nsg2 | 0.24 |
| 6888412 | Olfr1039 | 0.24 |
| 6878035 | Pdk1 | 0.24 |
| 6964798 | Ptpre | 0.24 |
| 6837935 | Abcd2 | 0.24 |
| 6950147 | Clec1b | 0.23 |
| 6849766 | Abcg1 | 0.23 |
| 6960198 | Klk1b27 | 0.23 |
| 6892579 | Tgm2 | 0.23 |
| 6980101 | Cd209d | 0.23 |
| 6755237 | Kcnj10 | 0.23 |
| 6829549 | Cmbl | 0.23 |
| 6915844 | Dnajc6 | 0.23 |
| 6900385 | Sort1 | 0.23 |
| 6993153 | Ccr2 | 0.23 |
| 6852836 | Epas1 | 0.23 |
| 6893057 | Sulf2 | 0.23 |
| 6751535 | Ramp1 | 0.23 |
| 6921157 | Sit1 | 0.22 |
| 6992475 | Als2cl | 0.22 |
| 6993865 | Acp5 | 0.22 |
| 6856756 | Ptprm | 0.22 |
| 6989015 | Il18 | 0.22 |
| 6940431 | Wdfy3 | 0.22 |
| 6991461 | Slc9a9 | 0.22 |
| 6792122 | Abca9 | 0.22 |
| 6760289 | 5033414K04Rik | 0.22 |
| 6824838 | Tgm1 | 0.22 |
| 6911010 | Sort1 | 0.22 |
| 6940658 | Abcg3 | 0.22 |
| 6750566 | Cyp27a1 | 0.22 |
| 6852399 | Qpct | 0.22 |
| 6969874 | Art2a | 0.21 |
| 6923142 | Gdap6 | 0.21 |
| 6757322 | EG241041 | 0.21 |
| 6766455 | Sgk1 | 0.21 |
| 6870622 | Adrb1 | 0.21 |
| 6749935 | Adam23 | 0.21 |
| 6780572 | Timd4 | 0.21 |
| 6748889 | Il18r1 | 0.21 |
| 6755091 | Sh2d1b1 | 0.21 |
| 6980606 | 2610019F03Rik | 0.21 |
| 6920954 | Ddx58 | 0.21 |
| 6966808 | Cd33 | 0.21 |
| 6957679 | Hebp1 | 0.21 |
| 6876944 | 2310010M24Rik | 0.21 |
| 6766577 | Vnn3 | 0.21 |
| 6969753 | Slco2b1 | 0.20 |
| 6783040 | OTTMUSG00000000971 | 0.20 |
| 6882730 | Lbp | 0.20 |
| 6950170 | Klrd1 | 0.20 |
| 7019818 | Tsc22d3 | 0.20 |
| 6965982 | Axl | 0.20 |
| 6927456 | Cnga1 | 0.20 |
| 6830506 | Col14a1 | 0.20 |
| 6855084 | Aif1 | 0.20 |
| 6766839 | A130091G23Rik | 0.20 |
| 6784829 | Map2k6 | 0.19 |
| 6753089 | Slc45a3 | 0.19 |
| 6784587 | Ace3 | 0.19 |
| 6785114 | Rab37 | 0.19 |
| 6939761 | Igj | 0.19 |
| 6957758 | Art4 | 0.19 |
| 6972168 | Ifitm3 | 0.19 |
| 6973587 | Apoe | 0.19 |
| 6846105 | Trat1 | 0.19 |
| 6792367 | Cd300lb | 0.18 |
| 6849951 | Myo1f | 0.18 |
| 6848199 | Mx1 | 0.18 |
| 6792368 | Cd300c | 0.18 |
| 6905422 | P2ry13 | 0.18 |
| 6941645 | Dtx1 | 0.18 |
| 6843550 | Mx1 | 0.18 |
| 6931961 | Lphn3 | 0.18 |
| 6867860 | Rasgrp2 | 0.18 |
| 6992855 | Itga9 | 0.18 |
| 6943168 | Slc46a3 | 0.18 |
| 6949730 | Clec4a4 | 0.18 |
| 6940432 | Wdfy3 | 0.18 |
| 6829612 | Pgcp | 0.17 |
| 6918015 | Pla2g2d | 0.17 |
| 6850534 | Pla2g7 | 0.17 |
| 6939069 | Cnga1 | 0.17 |
| 6888752 | Nr1h3 | 0.17 |
| 6969978 | Olfr610 | 0.17 |
| 6973527 | Lair1 | 0.17 |
| 6957744 | 1100001H23Rik | 0.17 |
| 6768868 | Ggt5 | 0.17 |
| 6957458 | Klra2 | 0.17 |
| 6765325 | Hsd11b1 | 0.17 |
| 6792832 | Cd7 | 0.16 |
| 6945584 | Tbxas1 | 0.16 |
| 6878038 | Rapgef4 | 0.16 |
| 6768618 | Slc16a9 | 0.16 |
| 6820113 | Gfra2 | 0.16 |
| 6792392 | Cd300e | 0.16 |
| 6827820 | Dzip1 | 0.16 |
| 6749933 | Adam23 | 0.16 |
| 6825600 | Adamdec1 | 0.16 |
| 6962133 | A530021J07Rik | 0.16 |
| 6785111 | Cd300a | 0.16 |
| 6837415 | Nfam1 | 0.16 |
| 7008556 | Ear1 | 0.16 |
| 6777957 | Lrp1 | 0.15 |
| 6764049 | Fcer1g | 0.15 |
| 6784844 | Kcnj16 | 0.15 |
| 6917549 | Fgr | 0.15 |
| 6977019 | Lrrc25 | 0.15 |
| 6978232 | Capns2 | 0.15 |
| 6957427 | Klri1 | 0.15 |
| 6811370 | Gpr141 | 0.15 |
| 6972491 | Ccnd1 | 0.15 |
| 6760518 | Arl4c | 0.15 |
| 6921670 | Abca1 | 0.15 |
| 6840637 | Itgb5 | 0.15 |
| 6851897 | Epb4 | 0.14 |
| 7020800 | Tlr8 | 0.14 |
| 6755189 | Cd244 | 0.14 |
| 6768572 | Tmem26 | 0.14 |
| 6972192 | Irf7 | 0.14 |
| 6964380 | Itgam | 0.14 |
| 6933625 | Oasl2 | 0.14 |
| 6824763 | Slc7a8 | 0.14 |
| 6883186 | Eya2 | 0.14 |
| 6960834 | Siglech | 0.14 |
| 6910592 | Ifi44 | 0.14 |
| 6750546 | Slc11a1 | 0.13 |
| 6777310 | Lyz1 | 0.13 |
| 6813474 | Tifab | 0.13 |
| 6934162 | P2rx7 | 0.13 |
| 6840129 | Rtp4 | 0.13 |
| 6869327 | Ifit3 | 0.13 |
| 6872785 | OTTMUSG00000016644 | 0.13 |
| 6950137 | Clec12a | 0.13 |
| 6959584 | Tyrobp | 0.13 |
| 6969878 | Art2b | 0.13 |
| 6790944 | Abcc3 | 0.13 |
| 6775236 | Trpm2 | 0.12 |
| 6949766 | Cd163 | 0.12 |
| 6755146 | Fcgr4 | 0.12 |
| 6881139 | Sirpa | 0.12 |
| 6772009 | Sash1 | 0.12 |
| 6977260 | Hmox1 | 0.11 |
| 6935701 | Alox5ap | 0.11 |
| 6950148 | Clec9a | 0.11 |
| 6966935 | Fcgrt | 0.11 |
| 7013185 | Tlr13 | 0.11 |
| 6993138 | Ccr9 | 0.11 |
| 6976609 | Ddx60 | 0.11 |
| 7006322 | Ear10 | 0.11 |
| 6980091 | Cd209a | 0.10 |
| 6970952 | Igsf6 | 0.10 |
| 6949727 | Clec4a3 | 0.10 |
| 6775864 | Spic | 0.10 |
| 6792371 | 4732429D16Rik | 0.10 |
| 6942580 | Pilrb1 | 0.10 |
| 6811694 | Hfe | 0.10 |
| 6903360 | Sirpb1 | 0.10 |
| 6876430 | Ptgs1 | 0.10 |
| 6941647 | Oas2 | 0.09 |
| 6854043 | Fpr1 | 0.09 |
| 6878045 | Rapgef4 | 0.09 |
| 6828403 | C6 | 0.09 |
| 6807336 | Tgfbi | 0.08 |
| 6833937 | Il7r | 0.08 |
| 6966818 | Siglece | 0.08 |
| 6991531 | Paqr9 | 0.07 |
| 6850821 | Treml4 | 0.07 |
| 6926167 | C1qa | 0.07 |
| 6885432 | Fcna | 0.07 |
| 6851324 | Emr1 | 0.07 |
| 6964382 | Itgad | 0.07 |
| 6777309 | Lyz1 | 0.07 |
| 6908486 | Vcam1 | 0.06 |
| 6881087 | Mertk | 0.06 |
| 6949722 | Clec4a1 | 0.06 |
| 6942579 | Pilra | 0.05 |
| 6926165 | C1qb | 0.04 |
| 6869691 | Dntt | 0.04 |
| 6898995 | Cd5l | 0.04 |
| 6998397 | Trf | 0.04 |
| 6926166 | C1qc | 0.04 |
| 6851186 | Emr4 | 0.04 |
| 6861358 | Csf1r | 0.04 |
| 6875181 | Mrc1 | 0.03 |
| 6976237 | Hpgd | 0.03 |
| 6993151 | Ccr3 | 0.02 |
| 6758435 | Slc40a1 | 0.01 |

**3,806 genes with greater than 2-fold change in transcript-level expression: TCR/CD28 activation as compared to naïve T cells**

| **TCID** | **Symbol** | **Fold change** |
| --- | --- | --- |
| 6958193 | Bcat1 | 48.21 |
| 6904309 | Il2 | 46.58 |
| 6788333 | Il3 | 43.11 |
| 6924813 | Hpdl | 33.79 |
| 6951525 | Asns | 29.65 |
| 6790294 | Ccl3 | 26.03 |
| 6994666 | Chek1 | 24.95 |
| 6925149 | Mfsd2 | 23.59 |
| 6978332 | Ccl22 | 23.43 |
| 6753400 | Lad1 | 21.96 |
| 6805825 | Irf4 | 21.70 |
| 7018524 | Slc7a3 | 21.39 |
| 6896860 | Spry1 | 21.23 |
| 6784054 | Cdc6 | 20.23 |
| 6899034 | Crabp2 | 19.85 |
| 6778391 | Lif | 19.49 |
| 6765235 | Dtl | 18.80 |
| 6965072 | Utf1 | 17.96 |
| 6871768 | Psat1 | 17.81 |
| 6783035 | Ccl4 | 17.58 |
| 6791171 | Tbx21 | 16.61 |
| 6916190 | Orc1l | 16.15 |
| 6916663 | Slc6a9 | 16.09 |
| 6954415 | Il12rb2 | 15.96 |
| 6976002 | Dctd | 15.63 |
| 6788329 | Csf2 | 15.34 |
| 6851232 | Uhrf1 | 15.12 |
| 6954982 | Hk2 | 14.86 |
| 6788791 | Shmt1 | 14.54 |
| 6946749 | Tnip3 | 14.18 |
| 6771052 | Ifng | 14.04 |
| 6789977 | Tmem97 | 13.90 |
| 6845978 | Cd200 | 13.81 |
| 6747972 | Il17a | 13.59 |
| 6975335 | Dusp4 | 13.56 |
| 6755387 | Exo1 | 13.42 |
| 6880497 | Rad51 | 13.27 |
| 6844253 | Sdf2l1 | 13.12 |
| 6869577 | Hells | 13.08 |
| 6786473 | Slc1a4 | 12.89 |
| 6913315 | Nr4a3 | 12.86 |
| 6786044 | Fignl1 | 12.63 |
| 6883000 | Mybl2 | 12.12 |
| 6985848 | Gins2 | 11.55 |
| 6785684 | Nefh | 11.40 |
| 6884441 | Mcm10 | 11.16 |
| 6875038 | Il2ra | 11.02 |
| 6777955 | Shmt2 | 10.96 |
| 6957144 | Lag3 | 10.69 |
| 6754536 | Tnfsf4 | 10.66 |
| 7013857 | Cenpi | 10.61 |
| 6919209 | Tnfrsf4 | 10.57 |
| 6915843 | Ak3l1 | 10.55 |
| 6849595 | Cdkn1a | 10.54 |
| 6933422 | Pole | 10.51 |
| 6878053 | Cdca7 | 10.25 |
| 6850705 | BC048355 | 9.93 |
| 6957465 | Csda | 9.84 |
| 6790648 | Akap1 | 9.82 |
| 6768232 | Ppa1 | 9.81 |
| 6948913 | Bhlhb2 | 9.78 |
| 6765153 | Smyd2 | 9.64 |
| 6977042 | Il12rb1 | 9.64 |
| 6757282 | Mcm3 | 9.55 |
| 6754138 | Rgs16 | 9.48 |
| 6882189 | Gins1 | 9.32 |
| 6977261 | Mcm5 | 9.31 |
| 6957133 | Tpi1 | 9.23 |
| 6925054 | Ctps | 9.19 |
| 6918720 | Srm | 9.16 |
| 6840579 | Tfrc | 9.11 |
| 6894454 | Phgdh | 9.09 |
| 6844359 | Cdc45l | 9.01 |
| 6933598 | Ung | 8.95 |
| 6792792 | Pycr1 | 8.85 |
| 6808948 | Dhfr | 8.64 |
| 6911682 | Ccne2 | 8.63 |
| 6977814 | Gpt2 | 8.60 |
| 6974850 | Eif4ebp1 | 8.57 |
| 7015392 | 2010204K13Rik | 8.56 |
| 6769262 | Thop1 | 8.54 |
| 6936690 | Pus7 | 8.34 |
| 6989874 | Tipin | 8.28 |
| 6790199 | Ccl1 | 8.24 |
| 6768951 | Slc19a1 | 8.21 |
| 6876109 | Freq | 8.21 |
| 6871476 | Fen1 | 8.16 |
| 6886799 | Idi1 | 8.15 |
| 6873217 | Got1 | 8.02 |
| 6985900 | Slc7a5 | 7.97 |
| 6815555 | Cenph | 7.96 |
| 6947553 | Smyd5 | 7.93 |
| 6889357 | Prrg4 | 7.92 |
| 6782679 | Tlcd1 | 7.87 |
| 6996267 | Zwilch | 7.85 |
| 6876209 | St6galnac4 | 7.84 |
| 6844598 | Rfc4 | 7.81 |
| 7010183 | Maoa | 7.77 |
| 6851204 | Chaf1a | 7.77 |
| 6917512 | Rcc1 | 7.65 |
| 6881340 | Mcm8 | 7.58 |
| 6892180 | BB166591 | 7.55 |
| 6792485 | Galk1 | 7.55 |
| 6955543 | Chchd4 | 7.54 |
| 6924763 | Rad54l | 7.50 |
| 6901944 | Gbp5 | 7.45 |
| 6846463 | 2610528E23Rik | 7.45 |
| 6906840 | Cks1b | 7.40 |
| 6913080 | Melk | 7.38 |
| 6901957 | Gbp2 | 7.33 |
| 6895672 | Fabp5 | 7.30 |
| 6832530 | Creld2 | 7.25 |
| 6830927 | Myc | 7.23 |
| 7024174 | Cenpm | 7.17 |
| 6900180 | Slc16a1 | 7.16 |
| 6819883 | Pbk | 7.15 |
| 6970839 | Coq7 | 7.14 |
| 6771620 | Prim1 | 7.13 |
| 6929671 | Cad | 7.13 |
| 6916775 | Hivep3 | 7.12 |
| 6951440 | Slc25a13 | 7.11 |
| 6901962 | Ccbl2 | 7.04 |
| 6796164 | Mthfd1 | 7.00 |
| 6776185 | Socs2 | 6.98 |
| 6941040 | Noc4l | 6.90 |
| 6768897 | Ndg2 | 6.89 |
| 6974156 | Tfdp1 | 6.78 |
| 6963418 | Wee1 | 6.78 |
| 7018279 | Spin4 | 6.71 |
| 6765982 | Timm8a1 | 6.71 |
| 6845274 | Umps | 6.71 |
| 6813326 | D13Wsu177e | 6.70 |
| 6766381 | 2610016C23Rik | 6.67 |
| 6982999 | 1810029B16Rik | 6.66 |
| 6769891 | Fdps | 6.62 |
| 6757278 | Il17f | 6.61 |
| 6765218 | Atf3 | 6.61 |
| 6760774 | Pask | 6.60 |
| 6911719 | E130016E03Rik | 6.52 |
| 7009795 | Pim2 | 6.48 |
| 6992229 | Ifrd2 | 6.48 |
| 7019517 | Timm8a1 | 6.47 |
| 6800859 | Egln3 | 6.47 |
| 6798403 | Ncapg2 | 6.46 |
| 6926344 | Mrto4 | 6.44 |
| 6939990 | Cxcl10 | 6.43 |
| 6929125 | AI847670 | 6.42 |
| 6786262 | Pno1 | 6.42 |
| 6955025 | Mthfd2 | 6.42 |
| 6985659 | Gcsh | 6.40 |
| 6780863 | Nola2 | 6.40 |
| 6824974 | F630043A04Rik | 6.38 |
| 6855087 | Tnf | 6.37 |
| 6766301 | Perp | 6.36 |
| 6943448 | Rfc3 | 6.36 |
| 6955381 | Mcm2 | 6.34 |
| 6774794 | Cdc2a | 6.32 |
| 6930074 | Lyar | 6.31 |
| 6758361 | Kdelc1 | 6.30 |
| 6871471 | Fads2 | 6.30 |
| 6990042 | Car12 | 6.28 |
| 6855088 | Lta | 6.27 |
| 6964253 | Hirip3 | 6.27 |
| 6964517 | Tacc2 | 6.27 |
| 6789474 | C1qbp | 6.26 |
| 6967013 | Grwd1 | 6.26 |
| 6926072 | Clic4 | 6.23 |
| 6988635 | Hyou1 | 6.22 |
| 6926976 | Slc25a33 | 6.20 |
| 6977804 | Orc6l | 6.20 |
| 6890715 | Bub1 | 6.19 |
| 6989438 | Sema7a | 6.17 |
| 6936981 | Tyms | 6.13 |
| 6793677 | Odc1 | 6.13 |
| 6854386 | Tbl3 | 6.11 |
| 6758743 | Hspd1 | 6.09 |
| 6806036 | Serpinb9 | 6.08 |
| 6818950 | Apex1 | 6.08 |
| 6852475 | Gemin6 | 6.07 |
| 6763682 | BC055324 | 6.06 |
| 6943485 | Lyar | 6.05 |
| 6941883 | Rilpl2 | 6.04 |
| 6801488 | L2hgdh | 5.99 |
| 6911213 | Fdps | 5.98 |
| 6837361 | Cenpm | 5.97 |
| 6778043 | Pa2g4 | 5.97 |
| 6949849 | Nol1 | 5.95 |
| 6869761 | Pgam1 | 5.94 |
| 6771533 | Mettl1 | 5.94 |
| 6824325 | Wdhd1 | 5.93 |
| 6836767 | Top1mt | 5.89 |
| 6812518 | Eef1e1 | 5.87 |
| 6917283 | Yars | 5.87 |
| 6824880 | Gzmb | 5.84 |
| 6992178 | Rrp9 | 5.83 |
| 6795768 | Ppil5 | 5.82 |
| 6916954 | Pabpc4 | 5.81 |
| 6966983 | Ruvbl2 | 5.81 |
| 6874683 | Nudt5 | 5.81 |
| 6867830 | Cdca5 | 5.80 |
| 6760009 | Serpine2 | 5.80 |
| 6759648 | Pecr | 5.80 |
| 6822191 | Ipo5 | 5.80 |
| 6777929 | Mars | 5.79 |
| 6785750 | Pold2 | 5.78 |
| 6966600 | Ccne1 | 5.76 |
| 6961932 | 5730590G19Rik | 5.76 |
| 6829667 | Pop1 | 5.75 |
| 6892344 | Ahcy | 5.75 |
| 6991358 | Plscr1 | 5.72 |
| 6989974 | 2810417H13Rik | 5.70 |
| 6935370 | Fscn1 | 5.69 |
| 6992409 | Nme6 | 5.69 |
| 6957252 | Rad51ap1 | 5.68 |
| 6966233 | Mif | 5.67 |
| 6768928 | Lss | 5.67 |
| 6979123 | Aars | 5.66 |
| 6762017 | Mcm6 | 5.65 |
| 6957462 | Magohb | 5.64 |
| 6998584 | Abhd14a | 5.63 |
| 6875662 | Zmynd19 | 5.62 |
| 6807154 | Sfxn1 | 5.61 |
| 6803780 | 2810452K22Rik | 5.61 |
| 6930484 | Lap3 | 5.60 |
| 6762679 | Ruvbl1 | 5.59 |
| 6909304 | Nola1 | 5.54 |
| 6892307 | E2f1 | 5.53 |
| 6919012 | Acot7 | 5.51 |
| 6849845 | Rrp1b | 5.51 |
| 6771920 | Mthfd1l | 5.49 |
| 6836782 | Pycrl | 5.47 |
| 6966922 | Prmt1 | 5.45 |
| 6791504 | Brca1 | 5.44 |
| 6855615 | Slc29a1 | 5.44 |
| 6935927 | Cyp51 | 5.43 |
| 6953331 | Ezh2 | 5.42 |
| 6769255 | Gadd45b | 5.42 |
| 6846011 | Ahcy | 5.42 |
| 6972882 | Prpf31 | 5.38 |
| 6884351 | Suv39h2 | 5.38 |
| 6765719 | Nup43 | 5.37 |
| 6775206 | Adarb1 | 5.36 |
| 6881115 | Rpo1 | 5.36 |
| 6904892 | Slc7a11 | 5.36 |
| 6870027 | Nolc1 | 5.35 |
| 6856096 | Sgol1 | 5.34 |
| 6855772 | Bysl | 5.33 |
| 6764956 | Rrp15 | 5.32 |
| 6791174 | Tbkbp1 | 5.32 |
| 6873154 | Rrp12 | 5.32 |
| 6940872 | Gfi1 | 5.32 |
| 6749773 | Nol5 | 5.31 |
| 6790317 | Dusp14 | 5.30 |
| 6878012 | Hat1 | 5.30 |
| 6860635 | Eif1a | 5.29 |
| 6950160 | Gabarapl1 | 5.29 |
| 6890658 | Ncaph | 5.27 |
| 6789237 | Rangrf | 5.27 |
| 6990859 | Irak1bp1 | 5.27 |
| 6992414 | Cdc25a | 5.27 |
| 6928457 | Cdk6 | 5.27 |
| 6880703 | AA467197 | 5.25 |
| 6789830 | Cpd | 5.25 |
| 6983950 | Neto2 | 5.25 |
| 6804686 | Idi2 | 5.25 |
| 6791419 | Atp5g1 | 5.25 |
| 6793672 | Pdia6 | 5.25 |
| 6823696 | Gnl3 | 5.24 |
| 6807022 | Cks2 | 5.24 |
| 6963049 | Rrm1 | 5.23 |
| 6790902 | Nme1 | 5.22 |
| 6771619 | Snrpd2 | 5.21 |
| 6969429 | 4632434I11Rik | 5.20 |
| 6943195 | Slc7a1 | 5.20 |
| 6961201 | Snrpa1 | 5.19 |
| 6994830 | Gramd1b | 5.19 |
| 6942939 | Jtv1 | 5.18 |
| 6782248 | Mybbp1a | 5.17 |
| 6918900 | Tnfrsf9 | 5.17 |
| 6989399 | Fabp5 | 5.16 |
| 7000876 | Il22 | 5.16 |
| 6843951 | Rsl1d1 | 5.16 |
| 6866110 | Amd1 | 5.16 |
| 6802727 | Dio2 | 5.16 |
| 6942604 | Mcm7 | 5.15 |
| 6789369 | Rai12 | 5.13 |
| 6946396 | Gars | 5.12 |
| 6844327 | Ranbp1 | 5.12 |
| 6972410 | Cars | 5.11 |
| 6835415 | Nudcd1 | 5.11 |
| 6840284 | Ccdc50 | 5.10 |
| 6927291 | Atad3a | 5.10 |
| 6769343 | Tdg | 5.10 |
| 6824829 | Ipo4 | 5.09 |
| 6985117 | Psmd7 | 5.09 |
| 6928741 | Abcb1b | 5.08 |
| 6826927 | Dis3 | 5.08 |
| 6791298 | Top2a | 5.07 |
| 6858816 | Snrpd1 | 5.07 |
| 6878710 | Timm10 | 5.06 |
| 6783137 | Myo19 | 5.06 |
| 6906437 | Mnd1 | 5.04 |
| 6799064 | Ddx1 | 5.04 |
| 6912213 | 1110007M04Rik | 5.03 |
| 6791465 | Psmc3ip | 5.03 |
| 7018582 | Ercc6l | 5.02 |
| 6942673 | 3110082I17Rik | 5.01 |
| 6812313 | Rpp40 | 5.01 |
| 7019532 | Armcx6 | 5.01 |
| 6869537 | Cep55 | 5.01 |
| 6783909 | Mrpl45 | 4.98 |
| 6919212 | Tnfrsf18 | 4.98 |
| 6785774 | Ddx56 | 4.98 |
| 6995074 | Hmbs | 4.98 |
| 6751103 | Ccl20 | 4.97 |
| 6864565 | Hspa9 | 4.96 |
| 6978935 | Cirh1a | 4.96 |
| 6990948 | Ttk | 4.95 |
| 6851309 | Tnfsf9 | 4.95 |
| 6865022 | Lars | 4.94 |
| 6796799 | 1810035L17Rik | 4.94 |
| 6958991 | Snrpd2 | 4.94 |
| 6939320 | Ppat | 4.93 |
| 6917180 | Clspn | 4.92 |
| 6809812 | Cenpk | 4.92 |
| 6873069 | Aldh18a1 | 4.90 |
| 6783674 | Lrrc59 | 4.90 |
| 6978869 | Nutf2 | 4.89 |
| 6947894 | Txnrd3 | 4.89 |
| 6871519 | Ccdc86 | 4.89 |
| 6764007 | Hsd17b7 | 4.88 |
| 6757744 | Bag2 | 4.88 |
| 6886908 | Nr4a2 | 4.88 |
| 6978523 | Impdh2 | 4.87 |
| 6921937 | Gapdh | 4.87 |
| 6892325 | Eif2s2 | 4.87 |
| 6830761 | Sqle | 4.87 |
| 6762420 | Timm17a | 4.87 |
| 6892496 | Dsn1 | 4.82 |
| 6869021 | Rcl1 | 4.82 |
| 6845382 | 2310056P07Rik | 4.82 |
| 6941029 | Pxmp2 | 4.82 |
| 6866233 | Lman1 | 4.81 |
| 6769166 | Wdr18 | 4.81 |
| 6932517 | Sept11 | 4.81 |
| 6792822 | Fasn | 4.80 |
| 6856208 | D17Wsu104e | 4.80 |
| 6847880 | Gart | 4.79 |
| 6757732 | Prim2 | 4.79 |
| 6944293 | Mdfic | 4.78 |
| 6924808 | Mmachc | 4.77 |
| 6835776 | Dscc1 | 4.77 |
| 6773537 | Tdg | 4.76 |
| 6825436 | Esco2 | 4.76 |
| 6912179 | F730047E07Rik | 4.75 |
| 7008131 | Psmb5 | 4.75 |
| 6910938 | Cth | 4.74 |
| 6947760 | Rpn1 | 4.74 |
| 6801454 | Pole2 | 4.73 |
| 6998583 | Acy1 | 4.72 |
| 6992349 | Impdh2 | 4.71 |
| 6979421 | Cenpn | 4.71 |
| 6806963 | Iars | 4.71 |
| 6858126 | Kpna2 | 4.70 |
| 6809447 | Mrps27 | 4.70 |
| 6985703 | Mphosph6 | 4.68 |
| 6803598 | Wars | 4.68 |
| 6980158 | Shcbp1 | 4.67 |
| 6791978 | Kpna2 | 4.67 |
| 6921130 | Stoml2 | 4.67 |
| 6977687 | Ddx39 | 4.67 |
| 6917301 | Marcksl1 | 4.67 |
| 6831869 | Polr2f | 4.65 |
| 6834013 | Tars | 4.65 |
| 6880853 | Dut | 4.64 |
| 7011263 | Phf6 | 4.64 |
| 6815870 | Ndufaf2 | 4.63 |
| 7018220 | Pola1 | 4.63 |
| 6844177 | Mcm4 | 4.63 |
| 6785441 | Mrpl12 | 4.62 |
| 6927967 | Slc31a1 | 4.62 |
| 6977971 | Heatr3 | 4.61 |
| 6843343 | Chaf1b | 4.60 |
| 6790475 | Brip1 | 4.60 |
| 6841372 | C330027C09Rik | 4.60 |
| 6789805 | Gemin4 | 4.60 |
| 6763894 | Uck2 | 4.60 |
| 6916483 | Stil | 4.60 |
| 6845909 | Qtrtd1 | 4.58 |
| 6793255 | Wdr35 | 4.58 |
| 6802433 | 0610007P14Rik | 4.58 |
| 6998596 | Armet | 4.58 |
| 6868055 | A430093F15Rik | 4.58 |
| 6881897 | Nat5 | 4.57 |
| 6924801 | Nasp | 4.57 |
| 6792031 | ENSMUSG00000075466 | 4.57 |
| 6892393 | BC029722 | 4.56 |
| 6791900 | Ftsj3 | 4.56 |
| 6935269 | Nudt1 | 4.55 |
| 6782830 | Cdk5r1 | 4.53 |
| 6876079 | 2610205E22Rik | 4.52 |
| 6971344 | 2410015N17Rik | 4.52 |
| 6925910 | Nudc | 4.52 |
| 6893636 | Slmo2 | 4.51 |
| 6832079 | Adsl | 4.50 |
| 6978948 | Nip7 | 4.50 |
| 6817617 | Ppif | 4.50 |
| 6781970 | Aurkb | 4.50 |
| 6754027 | Niban | 4.48 |
| 6752409 | Mki67ip | 4.47 |
| 6885287 | Mastl | 4.47 |
| 6836298 | Snrpc | 4.47 |
| 6885901 | Fpgs | 4.46 |
| 6918699 | Mad2l2 | 4.46 |
| 6768324 | Dna2 | 4.46 |
| 6810782 | Pfkp | 4.46 |
| 6817970 | Nt5dc2 | 4.46 |
| 6978890 | Prmt7 | 4.45 |
| 6871270 | Fkbp2 | 4.45 |
| 6773235 | Amd1 | 4.44 |
| 6985427 | Kars | 4.44 |
| 6770013 | Eea1 | 4.44 |
| 6913962 | Slc31a1 | 4.44 |
| 6911009 | Psma5 | 4.44 |
| 6993833 | Spc24 | 4.44 |
| 6987343 | Ppan | 4.42 |
| 6769357 | Txnrd1 | 4.42 |
| 6941423 | Rfc5 | 4.42 |
| 6998614 | Hemk1 | 4.41 |
| 6916127 | Tmem48 | 4.41 |
| 6977768 | Syce2 | 4.41 |
| 6966137 | Timm50 | 4.40 |
| 6992209 | Cish | 4.39 |
| 6867955 | Wdr74 | 4.38 |
| 6966166 | Nfkbib | 4.38 |
| 6988624 | Dpagt1 | 4.38 |
| 6978296 | Nup93 | 4.37 |
| 6872916 | Ide | 4.37 |
| 6917496 | Sesn2 | 4.36 |
| 6904074 | Mrpl47 | 4.36 |
| 6917524 | Rpa2 | 4.36 |
| 6892139 | Trib3 | 4.36 |
| 6922244 | Pole3 | 4.35 |
| 6880451 | Bub1b | 4.33 |
| 6772217 | Ltv1 | 4.33 |
| 6880516 | Chac1 | 4.32 |
| 6930838 | Pi4k2b | 4.31 |
| 6962751 | Alg8 | 4.31 |
| 6775870 | Utp20 | 4.30 |
| 6906857 | Hax1 | 4.30 |
| 6852225 | Ttc27 | 4.30 |
| 6828481 | Phb | 4.29 |
| 6860670 | Hspe1 | 4.29 |
| 6976520 | Sh3rf1 | 4.28 |
| 6807998 | Nsun2 | 4.28 |
| 6782368 | 1300001I01Rik | 4.27 |
| 6844250 | 2610318N02Rik | 4.26 |
| 6900383 | Psma5 | 4.26 |
| 6850780 | Mrps10 | 4.26 |
| 7005797 | Hmgcs1 | 4.26 |
| 6897002 | Plk4 | 4.25 |
| 6793674 | Nol10 | 4.25 |
| 6769509 | Pwp1 | 4.24 |
| 6824346 | Dlg7 | 4.23 |
| 7020410 | Sms | 4.22 |
| 6990067 | Rps27l | 4.22 |
| 6812770 | Tbc1d7 | 4.22 |
| 6839947 | Ece2 | 4.21 |
| 6811762 | Gmnn | 4.19 |
| 6773444 | Amd1 | 4.19 |
| 6780266 | Nudcd2 | 4.19 |
| 6949367 | Zfp239 | 4.19 |
| 6802124 | Erh | 4.18 |
| 6810697 | Akr1c18 | 4.18 |
| 6767610 | Prep | 4.18 |
| 6855145 | Mrps18b | 4.18 |
| 6962921 | 2610209A20Rik | 4.18 |
| 6885626 | Gtf3c5 | 4.17 |
| 6754696 | Slc19a2 | 4.17 |
| 6894898 | Pdss1 | 4.16 |
| 6916722 | Ebna1bp2 | 4.16 |
| 6837189 | Rps19bp1 | 4.16 |
| 6785641 | 1700020C11Rik | 4.16 |
| 6765734 | Zc3h12d | 4.16 |
| 6947394 | Gcs1 | 4.14 |
| 6859836 | Wdr36 | 4.13 |
| 6860129 | Wdr55 | 4.13 |
| 6904300 | Ccna2 | 4.13 |
| 6787283 | Rars | 4.12 |
| 6849974 | Cd320 | 4.12 |
| 6899252 | Pmvk | 4.12 |
| 6783761 | Phb | 4.12 |
| 6963017 | 3200002M19Rik | 4.12 |
| 6949206 | Tsen2 | 4.11 |
| 6993098 | Kif15 | 4.11 |
| 6896852 | Bbs12 | 4.11 |
| 6768125 | Timm23 | 4.10 |
| 6760777 | Hdlbp | 4.10 |
| 6926934 | Apitd1 | 4.10 |
| 6876150 | Exosc2 | 4.10 |
| 6992306 | Gmppb | 4.08 |
| 6899028 | Hdgf | 4.08 |
| 6936747 | Abcf2 | 4.08 |
| 6997634 | 2810026P18Rik | 4.08 |
| 6987638 | Thyn1 | 4.08 |
| 6753710 | Uchl5 | 4.08 |
| 6790318 | Tada2l | 4.08 |
| 6820047 | Tnfrsf10b | 4.07 |
| 6819662 | Rnaseh2b | 4.07 |
| 6849456 | Ergic1 | 4.07 |
| 6919184 | Mrpl20 | 4.07 |
| 6942176 | Psph | 4.06 |
| 6830510 | Mtbp | 4.06 |
| 6779392 | Pus10 | 4.05 |
| 6831628 | Cks2 | 4.05 |
| 6966611 | Pop4 | 4.04 |
| 6784329 | BC030867 | 4.04 |
| 6767519 | AK122525 | 4.03 |
| 6783740 | Slc35b1 | 4.03 |
| 6844310 | Slc25a1 | 4.03 |
| 6967882 | Mphosph10 | 4.03 |
| 7019640 | Morf4l2 | 4.03 |
| 6913499 | Smc2 | 4.03 |
| 6825216 | Kpna3 | 4.03 |
| 6960298 | Nup62 | 4.03 |
| 6873025 | Noc3l | 4.02 |
| 6819737 | C230072K23 | 4.02 |
| 6966875 | Pold1 | 4.02 |
| 6866068 | Txnl1 | 4.02 |
| 6795233 | 1110008L16Rik | 4.01 |
| 6780933 | Vdac1 | 4.01 |
| 7016317 | Nkrf | 4.01 |
| 6899104 | Cct3 | 4.01 |
| 6788274 | Uqcrq | 4.00 |
| 6837322 | Polr3h | 4.00 |
| 6901597 | Nhedc2 | 4.00 |
| 6824738 | Prmt5 | 4.00 |
| 6785576 | Sfi1 | 4.00 |
| 6848146 | Psmg1 | 3.99 |
| 6968790 | Blm | 3.99 |
| 6973762 | 2010309E21Rik | 3.98 |
| 6889301 | Nat10 | 3.98 |
| 6968533 | Tdg | 3.98 |
| 6763853 | Pogk | 3.97 |
| 6921074 | Oprs1 | 3.97 |
| 6916089 | Dhcr24 | 3.97 |
| 6865709 | Aldh7a1 | 3.96 |
| 6872426 | D19Bwg1357e | 3.96 |
| 6749937 | Fastkd2 | 3.96 |
| 6961279 | Igf1r | 3.96 |
| 6753058 | 2700049P18Rik | 3.96 |
| 6986032 | Nup133 | 3.95 |
| 6791087 | Atp5g1 | 3.94 |
| 6913132 | Polr1e | 3.94 |
| 6875039 | Il15ra | 3.94 |
| 6963413 | Ipo7 | 3.94 |
| 6794293 | Nampt | 3.93 |
| 6937334 | Nol14 | 3.93 |
| 6749851 | Ctla4 | 3.93 |
| 6977016 | Isyna1 | 3.93 |
| 6822772 | Psmd6 | 3.93 |
| 6802507 | Gstz1 | 3.92 |
| 6782141 | Eno3 | 3.91 |
| 6763591 | 5630401D24Rik | 3.91 |
| 6984573 | Got2 | 3.91 |
| 6903095 | Mrps28 | 3.91 |
| 6942669 | Centa1 | 3.91 |
| 6934584 | Ran | 3.91 |
| 6804849 | Heatr1 | 3.91 |
| 7013187 | Pgk1 | 3.90 |
| 6942694 | Psmg3 | 3.90 |
| 6756474 | A130010J15Rik | 3.90 |
| 6824206 | Ero1l | 3.89 |
| 6804608 | Pitrm1 | 3.89 |
| 6784709 | Psmd12 | 3.89 |
| 6881172 | Nol5a | 3.89 |
| 6977696 | Asf1b | 3.89 |
| 6854690 | Ppil1 | 3.88 |
| 6749569 | Hspe1 | 3.88 |
| 6870063 | Nfkb2 | 3.88 |
| 6917270 | Ak2 | 3.88 |
| 6954935 | Mrpl19 | 3.88 |
| 6882232 | Fkbp1a | 3.88 |
| 6850071 | Rdbp | 3.87 |
| 6784039 | Psmd3 | 3.87 |
| 6851291 | Clpp | 3.87 |
| 6859031 | BC003885 | 3.87 |
| 6856231 | Ptprs | 3.87 |
| 6858352 | Tcp1 | 3.86 |
| 6866116 | Nars | 3.86 |
| 6790478 | Ints2 | 3.86 |
| 6809524 | Smn1 | 3.86 |
| 6966030 | Snrpa | 3.85 |
| 6766863 | Echdc1 | 3.85 |
| 6859837 | Wdr36 | 3.84 |
| 6876219 | Ptrh1 | 3.84 |
| 6989403 | Rpp25 | 3.84 |
| 6917437 | Mecr | 3.84 |
| 6864709 | Hars | 3.83 |
| 6818523 | Ptger2 | 3.83 |
| 6815792 | Ipo11 | 3.82 |
| 6813877 | Zfp367 | 3.82 |
| 6853910 | AV024533 | 3.82 |
| 6833972 | Bxdc2 | 3.82 |
| 6934652 | Tpst1 | 3.81 |
| 6898162 | Gfm1 | 3.81 |
| 6800229 | Scin | 3.81 |
| 6864444 | Stard4 | 3.81 |
| 6955981 | Shq1 | 3.80 |
| 6825302 | Fdft1 | 3.80 |
| 6788797 | Gtlf3a | 3.80 |
| 6861722 | Psmg2 | 3.79 |
| 6775246 | Pwp2 | 3.79 |
| 6843207 | Mrps6 | 3.79 |
| 6792497 | Mrpl38 | 3.79 |
| 6966265 | Tbcb | 3.79 |
| 6768783 | Zwint | 3.78 |
| 6941897 | Eif2b1 | 3.78 |
| 6864564 | Etf1 | 3.77 |
| 6981616 | Erh | 3.77 |
| 6791992 | Nol11 | 3.77 |
| 6875094 | Pter | 3.77 |
| 6858456 | Cul2 | 3.76 |
| 6876022 | Coq4 | 3.76 |
| 6872292 | Cbwd1 | 3.76 |
| 6849091 | Zfp52 | 3.76 |
| 6788563 | Gemin5 | 3.75 |
| 6874215 | Prdx3 | 3.75 |
| 6887819 | Ola1 | 3.75 |
| 6782411 | Tsr1 | 3.75 |
| 6899146 | Ssr2 | 3.75 |
| 6989209 | Idh3a | 3.74 |
| 6838797 | Cbx5 | 3.74 |
| 6778352 | Pes1 | 3.74 |
| 6854813 | Wdr4 | 3.73 |
| 6781240 | Larp1 | 3.73 |
| 6858291 | Tcp1 | 3.72 |
| 6771538 | Cdk4 | 3.72 |
| 6841097 | Nat13 | 3.72 |
| 6854430 | Chtf18 | 3.72 |
| 6971272 | Eif3c | 3.72 |
| 6935982 | Gtpbp10 | 3.71 |
| 6865108 | Eif3j | 3.71 |
| 6815558 | Ccnb1 | 3.71 |
| 6869414 | Pcgf5 | 3.71 |
| 6949800 | Phb2 | 3.70 |
| 6960436 | Nomo1 | 3.70 |
| 7022805 | Nhp2l1 | 3.70 |
| 6769286 | Dohh | 3.70 |
| 6788291 | Il13 | 3.70 |
| 6965197 | Rangrf | 3.70 |
| 6756345 | Nsl1 | 3.69 |
| 6904330 | Cetn4 | 3.69 |
| 6884339 | Rpp38 | 3.69 |
| 6748534 | Imp4 | 3.69 |
| 6844254 | Ccdc116 | 3.68 |
| 6837320 | Phf5a | 3.68 |
| 6785808 | Tbrg4 | 3.68 |
| 6966108 | Psmc4 | 3.68 |
| 6790231 | Nle1 | 3.67 |
| 6854449 | 9530058B02Rik | 3.67 |
| 6763731 | Xcl1 | 3.67 |
| 6835806 | Mrpl13 | 3.67 |
| 6966043 | Pgam1 | 3.66 |
| 6979559 | Irf8 | 3.66 |
| 6855659 | Vegfa | 3.66 |
| 6934053 | C330023M02Rik | 3.66 |
| 6958905 | Slc1a5 | 3.66 |
| 6929604 | D5Wsu178e | 3.66 |
| 6966298 | Zbtb32 | 3.66 |
| 6782498 | Rnmtl1 | 3.66 |
| 6821431 | Uchl3 | 3.65 |
| 7017603 | Ard1 | 3.65 |
| 6763972 | Nuf2 | 3.65 |
| 6992367 | Prkar2a | 3.65 |
| 6944952 | Snd1 | 3.64 |
| 6850678 | Mrps18a | 3.64 |
| 6815390 | Utp15 | 3.64 |
| 6909516 | Gstcd | 3.64 |
| 6787890 | Cdk2ap1 | 3.64 |
| 6781441 | Drg2 | 3.63 |
| 6839685 | Yars2 | 3.63 |
| 6840086 | Dnajb11 | 3.63 |
| 6795780 | Klhdc2 | 3.63 |
| 6838134 | Pus7l | 3.63 |
| 6790585 | Dynll2 | 3.62 |
| 6806034 | Serpinb6b | 3.62 |
| 6758949 | Orc2l | 3.62 |
| 6747471 | Rrs1 | 3.62 |
| 6881341 | Crls1 | 3.62 |
| 6790966 | Eme1 | 3.61 |
| 6798223 | 6720458F09Rik | 3.61 |
| 6816708 | Rpp14 | 3.61 |
| 6774404 | Ddx21 | 3.61 |
| 6844468 | Alg3 | 3.61 |
| 6850711 | Mrpl2 | 3.60 |
| 6876423 | Mrrf | 3.60 |
| 6855122 | Gtf2h4 | 3.60 |
| 6915253 | Mtap | 3.60 |
| 6762796 | Rgs1 | 3.60 |
| 6983570 | Rbmxrt | 3.60 |
| 6957346 | Fkbp4 | 3.59 |
| 6837339 | Nhp2l1 | 3.59 |
| 6980949 | Slc25a15 | 3.59 |
| 6935116 | Cops6 | 3.59 |
| 6854401 | Hn1l | 3.59 |
| 6784606 | Psmc5 | 3.59 |
| 6907534 | Wdr3 | 3.59 |
| 6839754 | Smpd4 | 3.58 |
| 6883273 | Ddx27 | 3.58 |
| 6792601 | Tk1 | 3.58 |
| 6978341 | Polr2c | 3.58 |
| 6836827 | Bop1 | 3.58 |
| 6750552 | Rqcd1 | 3.58 |
| 6764457 | Adss | 3.58 |
| 6971028 | Ndufab1 | 3.58 |
| 6785943 | Polr2c | 3.58 |
| 6924832 | Plk3 | 3.58 |
| 6771636 | Timeless | 3.58 |
| 6935273 | Eif3b | 3.57 |
| 6912923 | Nudt2 | 3.57 |
| 6966308 | Tmem147 | 3.57 |
| 6921559 | Mrpl50 | 3.56 |
| 6880661 | Eif3j | 3.56 |
| 6932570 | Gapdh | 3.56 |
| 6870181 | Gsto1 | 3.56 |
| 7012842 | Kif4 | 3.56 |
| 6941412 | Pebp1 | 3.56 |
| 6778278 | Eif4enif1 | 3.56 |
| 6812143 | Gmds | 3.56 |
| 6985925 | Mvd | 3.56 |
| 6892767 | Ptpla | 3.55 |
| 6776193 | D10Ertd322e | 3.55 |
| 6908092 | Ampd2 | 3.55 |
| 6943310 | Hsph1 | 3.55 |
| 6925579 | Eif3i | 3.55 |
| 6980944 | Ckap2 | 3.54 |
| 6799852 | Dus4l | 3.54 |
| 6766063 | Adat2 | 3.54 |
| 6750351 | Atic | 3.54 |
| 6823780 | Timm23 | 3.54 |
| 6864748 | Uxt | 3.54 |
| 6901757 | Eif4e | 3.53 |
| 6871277 | Stip1 | 3.53 |
| 6808209 | Mrpl36 | 3.53 |
| 6978884 | Slc7a6 | 3.53 |
| 6749720 | Nif3l1 | 3.53 |
| 6925254 | Cdca8 | 3.53 |
| 6777353 | Nup107 | 3.53 |
| 6831867 | Eif3eip | 3.53 |
| 6942554 | Pop7 | 3.52 |
| 6935756 | Brca2 | 3.52 |
| 6768891 | Derl3 | 3.52 |
| 6959397 | Fbl | 3.52 |
| 6946778 | Mad2l1 | 3.52 |
| 6774947 | Cisd1 | 3.51 |
| 6941751 | Atp2a2 | 3.51 |
| 6939986 | Sdad1 | 3.51 |
| 6782224 | Txndc17 | 3.51 |
| 6810893 | Gtpbp4 | 3.50 |
| 6781029 | Acsl6 | 3.50 |
| 6964913 | Glrx3 | 3.50 |
| 6815305 | Hmgcr | 3.50 |
| 6878296 | Mtx2 | 3.50 |
| 6832532 | Pim3 | 3.50 |
| 6998165 | Capza1 | 3.50 |
| 6756815 | Cops5 | 3.50 |
| 6900082 | Tspan2 | 3.49 |
| 6964214 | Tufm | 3.49 |
| 6931835 | Paics | 3.49 |
| 6975913 | Casp3 | 3.49 |
| 6820372 | Nufip1 | 3.49 |
| 6817396 | A430108C13Rik | 3.49 |
| 6850090 | Lsm2 | 3.49 |
| 6923313 | Glrx3 | 3.48 |
| 6796310 | Eif2s1 | 3.48 |
| 6989237 | AY074887 | 3.48 |
| 6785147 | Nup85 | 3.48 |
| 6986086 | 2810004N23Rik | 3.48 |
| 6863299 | Usp14 | 3.48 |
| 6828573 | Nup155 | 3.47 |
| 6924281 | Mrpl37 | 3.47 |
| 6907623 | Ptgfrn | 3.47 |
| 6856269 | Gtf2f1 | 3.47 |
| 6782655 | Dhrs13 | 3.47 |
| 6974490 | Agpat5 | 3.46 |
| 6929298 | Abcb8 | 3.46 |
| 6791493 | Aarsd1 | 3.46 |
| 6917048 | Yrdc | 3.46 |
| 6901055 | Sec24d | 3.46 |
| 6948008 | Lsm3 | 3.46 |
| 6985991 | Fanca | 3.46 |
| 6946511 | Lancl2 | 3.45 |
| 6840860 | Tmem39a | 3.45 |
| 6993719 | Eif3g | 3.45 |
| 6880034 | Nola3 | 3.45 |
| 6985408 | Cfdp1 | 3.45 |
| 6748883 | Il1r2 | 3.45 |
| 7011852 | Hmgb3 | 3.44 |
| 6769076 | Lsm7 | 3.44 |
| 6840837 | Cox17 | 3.44 |
| 6763493 | Cacybp | 3.44 |
| 6775411 | Lmnb2 | 3.44 |
| 6877924 | Bbs5 | 3.44 |
| 6768901 | Zfp280b | 3.43 |
| 6871515 | Tmem109 | 3.43 |
| 7011603 | Eif4e | 3.43 |
| 6782564 | Hsp90aa1 | 3.43 |
| 6775238 | Pfkl | 3.43 |
| 6929816 | Tacc3 | 3.43 |
| 6871097 | Banf1 | 3.43 |
| 6947555 | Cct7 | 3.43 |
| 6850140 | Nhp2l1 | 3.43 |
| 6871808 | Pebp1 | 3.42 |
| 6883132 | Cd40 | 3.42 |
| 6915734 | Usp1 | 3.42 |
| 6837122 | Josd1 | 3.41 |
| 6894152 | Psma7 | 3.41 |
| 6929152 | Psmc2 | 3.41 |
| 6851191 | Ebi3 | 3.41 |
| 6929510 | Insig1 | 3.41 |
| 6782704 | Poldip2 | 3.41 |
| 6847632 | Cct8 | 3.41 |
| 6911287 | Msto1 | 3.41 |
| 6984991 | E130303B06Rik | 3.41 |
| 6896804 | Exosc9 | 3.40 |
| 6791639 | Eftud2 | 3.40 |
| 6774395 | Supv3l1 | 3.40 |
| 7011953 | Ssr4 | 3.40 |
| 6892209 | Plagl2 | 3.40 |
| 6825696 | Polr3d | 3.40 |
| 7014155 | Prps1 | 3.40 |
| 6861308 | Iigp1 | 3.39 |
| 6791541 | Lsm12 | 3.39 |
| 6777898 | Tsfm | 3.39 |
| 7015028 | Fancb | 3.39 |
| 6981497 | Rbm13 | 3.39 |
| 6868516 | 2410127L17Rik | 3.39 |
| 6965668 | Ppp5c | 3.38 |
| 6832394 | Gtse1 | 3.38 |
| 6788657 | Guk1 | 3.38 |
| 6950555 | Strap | 3.38 |
| 6835932 | 9130401M01Rik | 3.38 |
| 6798272 | Siva1 | 3.38 |
| 6761691 | Dbi | 3.37 |
| 6995771 | Wdr61 | 3.37 |
| 6918991 | Dnajc11 | 3.37 |
| 6934258 | Ccdc62 | 3.37 |
| 6803759 | Hsp90aa1 | 3.37 |
| 6824751 | Psmb5 | 3.36 |
| 6849287 | Rnps1 | 3.36 |
| 6876342 | Hspa5 | 3.36 |
| 6781396 | Med9 | 3.36 |
| 6937056 | Slc5a6 | 3.35 |
| 6936585 | Dnajc2 | 3.35 |
| 6781727 | Elac2 | 3.35 |
| 6790367 | Znhit3 | 3.35 |
| 6857212 | Dpy30 | 3.35 |
| 6917069 | Gnl2 | 3.34 |
| 6835934 | Atad2 | 3.34 |
| 6882333 | Tpx2 | 3.34 |
| 6987422 | Prkcsh | 3.34 |
| 6936780 | Xrcc2 | 3.33 |
| 6888937 | Hsd17b12 | 3.33 |
| 6941048 | Pus1 | 3.33 |
| 6759949 | Farsb | 3.32 |
| 6965087 | Mtg1 | 3.32 |
| 7015445 | Suv39h1 | 3.32 |
| 6970396 | Tmem41b | 3.32 |
| 6832153 | Xrcc6 | 3.32 |
| 6881837 | Sec23b | 3.32 |
| 6880043 | Aven | 3.32 |
| 6969788 | Pold3 | 3.32 |
| 6898609 | Ppid | 3.32 |
| 6826546 | Diap3 | 3.32 |
| 6910708 | Rabggtb | 3.32 |
| 6913222 | Anp32b | 3.32 |
| 6782132 | Psmb6 | 3.31 |
| 6839952 | Psmd2 | 3.31 |
| 6819274 | 2610027L16Rik | 3.31 |
| 6855675 | Rpo1 | 3.31 |
| 6831651 | Cyc1 | 3.31 |
| 6867706 | Mrpl11 | 3.31 |
| 7002980 | Bcl2a1b | 3.31 |
| 6782389 | Mett10d | 3.31 |
| 6823051 | Ecd | 3.31 |
| 6957145 | Ptms | 3.30 |
| 6900236 | Wdr77 | 3.30 |
| 6824961 | N6amt2 | 3.30 |
| 6997671 | Tmed3 | 3.30 |
| 6839334 | Nubp1 | 3.30 |
| 6919885 | Otud6b | 3.29 |
| 6859779 | Polr2d | 3.29 |
| 6882397 | Dnmt3b | 3.29 |
| 6972032 | Ndufab1 | 3.29 |
| 6957150 | Cops7a | 3.29 |
| 6759778 | Abcb6 | 3.29 |
| 6814491 | Ankrd32 | 3.29 |
| 6867707 | Slc29a2 | 3.29 |
| 6954584 | Mrpl35 | 3.29 |
| 6843629 | Trap1 | 3.28 |
| 6991383 | Plod2 | 3.28 |
| 6797387 | Psmc1 | 3.28 |
| 6873364 | Npm3 | 3.27 |
| 6856239 | Lonp1 | 3.27 |
| 6858422 | Rpo1 | 3.27 |
| 6764351 | Fh1 | 3.27 |
| 6987391 | Carm1 | 3.27 |
| 6906807 | Msto1 | 3.27 |
| 6765130 | Pdcd5 | 3.27 |
| 6869407 | Rpp30 | 3.27 |
| 6987331 | Pin1 | 3.27 |
| 6987350 | Icam1 | 3.27 |
| 6799800 | Acp1 | 3.26 |
| 6785489 | Gps1 | 3.26 |
| 6801335 | C79407 | 3.26 |
| 6885749 | D2Wsu81e | 3.26 |
| 6839959 | Polr2h | 3.25 |
| 6798429 | Cdca7l | 3.25 |
| 6966494 | Nudt19 | 3.25 |
| 6935613 | Pomp | 3.25 |
| 6785148 | Mrps7 | 3.25 |
| 6985009 | Ddx28 | 3.24 |
| 6791565 | Slc25a39 | 3.24 |
| 6901732 | Rg9mtd2 | 3.24 |
| 6917190 | Psmb2 | 3.24 |
| 6854828 | Rps2 | 3.24 |
| 6868128 | Mrpl16 | 3.24 |
| 7011039 | Utp14a | 3.24 |
| 6750413 | Xrcc5 | 3.24 |
| 6844362 | Mrpl40 | 3.23 |
| 6985108 | Nob1 | 3.23 |
| 6905333 | Pgk1 | 3.23 |
| 6789365 | Eif5a | 3.23 |
| 6871164 | Pola2 | 3.23 |
| 6934923 | Nsun5 | 3.23 |
| 6953522 | Cycs | 3.23 |
| 6976325 | Gapdh | 3.23 |
| 6817930 | Tkt | 3.23 |
| 6836839 | Slc39a4 | 3.22 |
| 6833216 | Larp4 | 3.22 |
| 6909375 | 2310008M10Rik | 3.22 |
| 6783063 | Acaca | 3.22 |
| 6880322 | Spred1 | 3.22 |
| 6880906 | Dtwd1 | 3.22 |
| 7020169 | Tsr2 | 3.22 |
| 6889921 | Atpbd4 | 3.22 |
| 6932821 | Enoph1 | 3.22 |
| 6773164 | Rwdd1 | 3.21 |
| 6785935 | Hus1 | 3.21 |
| 6964734 | Bccip | 3.21 |
| 6800912 | 2700097O09Rik | 3.21 |
| 6821298 | 6720463M24Rik | 3.20 |
| 6941472 | Fbxw8 | 3.20 |
| 6913223 | Nans | 3.20 |
| 6962773 | Clns1a | 3.20 |
| 6836849 | Recql4 | 3.20 |
| 6900388 | Psrc1 | 3.20 |
| 6785485 | Hmga1 | 3.19 |
| 6760804 | Pdcd1 | 3.19 |
| 6982921 | Sc4mol | 3.19 |
| 6930385 | Ppih | 3.19 |
| 6888720 | Ptpmt1 | 3.19 |
| 6867593 | 1810055G02Rik | 3.19 |
| 6809550 | Taf9 | 3.18 |
| 6933927 | Rbm19 | 3.18 |
| 6891070 | Pcna | 3.18 |
| 6926011 | Zfp593 | 3.18 |
| 6827203 | Rnf219 | 3.18 |
| 6825544 | Cdca2 | 3.18 |
| 6834752 | Cct5 | 3.18 |
| 6809935 | Dimt1 | 3.18 |
| 6781138 | G3bp1 | 3.17 |
| 6796777 | Gstz1 | 3.17 |
| 6953443 | Igf2bp3 | 3.17 |
| 6902192 | Ssx2ip | 3.17 |
| 6971393 | Bcl7c | 3.17 |
| 6836983 | Eif3d | 3.17 |
| 6959004 | Ercc1 | 3.17 |
| 6977766 | Farsa | 3.17 |
| 6915504 | Ift74 | 3.17 |
| 6813315 | Thoc3 | 3.17 |
| 6970155 | Mrpl17 | 3.16 |
| 6925236 | Utp11l | 3.16 |
| 6912499 | Mdn1 | 3.16 |
| 6761760 | Ddx18 | 3.15 |
| 7017897 | Gapdh | 3.15 |
| 6782828 | Psmd11 | 3.15 |
| 6946011 | Repin1 | 3.15 |
| 6932853 | Cops4 | 3.15 |
| 6947404 | Bola3 | 3.15 |
| 6934645 | 2410018M08Rik | 3.15 |
| 6989044 | Alg9 | 3.15 |
| 6901334 | Pla2g12a | 3.15 |
| 6966497 | Pdcd5 | 3.14 |
| 6929279 | Nupl2 | 3.14 |
| 6800233 | Ifrd1 | 3.14 |
| 6974195 | Zfp828 | 3.14 |
| 6982580 | Spcs3 | 3.13 |
| 6831654 | Brp16 | 3.13 |
| 6785299 | Syngr2 | 3.13 |
| 6886170 | Psmb7 | 3.12 |
| 6780430 | Il12b | 3.12 |
| 6877587 | Psmd14 | 3.12 |
| 6966193 | Psmd8 | 3.12 |
| 6878730 | Ssrp1 | 3.12 |
| 6935524 | Cdk8 | 3.12 |
| 6870130 | Pdcd11 | 3.12 |
| 6780961 | Zcchc10 | 3.12 |
| 6775148 | Ddt | 3.12 |
| 6760364 | Ncl | 3.11 |
| 6766848 | Pebp1 | 3.11 |
| 6793649 | E2f6 | 3.11 |
| 6903753 | Ect2 | 3.11 |
| 6790901 | Nme2 | 3.11 |
| 6858868 | Rbbp8 | 3.11 |
| 6995508 | Sdhd | 3.11 |
| 6792784 | Thoc4 | 3.11 |
| 6992278 | Traip | 3.11 |
| 6937867 | Qdpr | 3.11 |
| 6843923 | Litaf | 3.11 |
| 6819152 | Mrpl52 | 3.11 |
| 6801560 | Timm9 | 3.11 |
| 6948815 | Trnt1 | 3.10 |
| 6884277 | Tpd52l2 | 3.10 |
| 6770718 | Nap1l1 | 3.10 |
| 6934902 | Tbl2 | 3.10 |
| 7012598 | Apoo | 3.09 |
| 6801703 | Trmt5 | 3.09 |
| 6782553 | Blmh | 3.09 |
| 6759043 | Wdr12 | 3.09 |
| 6783297 | Trim37 | 3.09 |
| 6873960 | Dclre1a | 3.09 |
| 6921124 | Vcp | 3.08 |
| 6783934 | Psmb3 | 3.08 |
| 6836979 | Txn2 | 3.08 |
| 6784257 | Psme3 | 3.08 |
| 6878713 | Slc43a3 | 3.08 |
| 6924960 | Ppih | 3.08 |
| 6837425 | 1110014J01Rik | 3.07 |
| 6843643 | Tcfap4 | 3.07 |
| 6784244 | Tubg1 | 3.07 |
| 6946953 | Rpo1 | 3.07 |
| 6756989 | Lactb2 | 3.07 |
| 6796644 | Fcf1 | 3.07 |
| 6768065 | Hsf2 | 3.07 |
| 6776104 | Metap2 | 3.07 |
| 6812444 | Ssr1 | 3.06 |
| 6917981 | Ddost | 3.06 |
| 6942373 | Wbscr16 | 3.06 |
| 6939987 | Cxcl9 | 3.06 |
| 6899722 | Anp32e | 3.06 |
| 6749813 | Cyp20a1 | 3.06 |
| 6789419 | Pelp1 | 3.06 |
| 6909495 | Scye1 | 3.06 |
| 6979636 | Cdt1 | 3.06 |
| 6929709 | Gpn1 | 3.06 |
| 6860903 | Srfbp1 | 3.06 |
| 6937254 | Slbp | 3.06 |
| 6798930 | Pgk1 | 3.05 |
| 6840278 | Ostn | 3.05 |
| 6779845 | Ubtd2 | 3.05 |
| 6796345 | Rad51l1 | 3.05 |
| 6841739 | Tomm70a | 3.04 |
| 6967211 | E2f8 | 3.04 |
| 6941937 | Dhx37 | 3.04 |
| 6870957 | Ndufs8 | 3.04 |
| 6921986 | Txn1 | 3.04 |
| 6899766 | Rbm8a | 3.04 |
| 7013137 | 2610029G23Rik | 3.03 |
| 6997211 | Cox7a2 | 3.03 |
| 6792789 | Mafg | 3.03 |
| 6885728 | Wdr34 | 3.03 |
| 6995515 | Dlat | 3.03 |
| 6973664 | Actg1 | 3.03 |
| 6788725 | Pemt | 3.03 |
| 6848940 | Riok2 | 3.03 |
| 6750557 | Bcs1l | 3.03 |
| 6769614 | Nup37 | 3.02 |
| 6929591 | ENSMUSG00000073107 | 3.02 |
| 6792779 | P4hb | 3.02 |
| 6833394 | Soat2 | 3.02 |
| 7021573 | Eif1ay | 3.01 |
| 6859411 | Mapre2 | 3.01 |
| 6994667 | Stt3a | 3.01 |
| 6789475 | Dhx33 | 3.01 |
| 6841399 | Ift57 | 3.01 |
| 6840805 | Rabl3 | 3.01 |
| 6963578 | Mlstd2 | 3.01 |
| 6939162 | Usp46 | 3.01 |
| 6785665 | 1110020P15Rik | 3.00 |
| 6869503 | Kif11 | 3.00 |
| 7016713 | Aifm1 | 3.00 |
| 6945032 | Smo | 3.00 |
| 6839928 | Eif2b5 | 3.00 |
| 7020371 | Gm650 | 3.00 |
| 6881735 | Snrpb2 | 3.00 |
| 6791207 | E130012A19Rik | 2.99 |
| 6838730 | Aaas | 2.99 |
| 6923058 | 6230416J20Rik | 2.99 |
| 6951304 | Bet1 | 2.99 |
| 6818556 | Psmc6 | 2.99 |
| 6846437 | Tfg | 2.99 |
| 6847184 | Stch | 2.99 |
| 6787896 | Irgm | 2.99 |
| 6971293 | Aldoa | 2.99 |
| 7015993 | Uxt | 2.99 |
| 6942474 | Srcrb4d | 2.98 |
| 6833404 | Espl1 | 2.98 |
| 6806038 | Serpinb9b | 2.98 |
| 7018510 | Pdzd11 | 2.98 |
| 7020677 | Siah1b | 2.98 |
| 6898309 | Nmd3 | 2.98 |
| 7020870 | Hccs | 2.98 |
| 7011907 | Nsdhl | 2.97 |
| 6875865 | D2Bwg1423e | 2.97 |
| 6934321 | Gtf2h3 | 2.97 |
| 6868823 | Cycs | 2.97 |
| 6880337 | 2610510H03Rik | 2.97 |
| 6947679 | Gfpt1 | 2.97 |
| 6796048 | Hif1a | 2.97 |
| 6905405 | Gpr171 | 2.97 |
| 6882412 | Tomm20 | 2.97 |
| 6771141 | 1190005P17Rik | 2.97 |
| 6860121 | Slc35a4 | 2.96 |
| 6858246 | Acat2 | 2.96 |
| 6876033 | Set | 2.96 |
| 6796299 | Gphn | 2.96 |
| 6863002 | Crem | 2.96 |
| 6949089 | Jagn1 | 2.96 |
| 6765723 | Katna1 | 2.96 |
| 6764372 | Rbm8a | 2.96 |
| 6855725 | Rpl7l1 | 2.96 |
| 6918098 | Rcc2 | 2.96 |
| 6964241 | Bola2 | 2.96 |
| 6837328 | Pmm1 | 2.95 |
| 6983624 | Abce1 | 2.95 |
| 6965620 | Sae1 | 2.95 |
| 6841969 | Tera | 2.95 |
| 6826029 | Armc10 | 2.95 |
| 6959568 | Polr2i | 2.95 |
| 6998396 | Srprb | 2.95 |
| 6934647 | Vkorc1l1 | 2.94 |
| 6977164 | Usmg5 | 2.94 |
| 6868066 | Prpf19 | 2.94 |
| 6984484 | Ciapin1 | 2.94 |
| 6870566 | Acsl5 | 2.94 |
| 6769934 | Ccdc41 | 2.94 |
| 6890617 | Cops2 | 2.94 |
| 6924834 | Kif2c | 2.94 |
| 6849507 | Hmga1 | 2.93 |
| 6975966 | Rwdd4a | 2.93 |
| 6790339 | Aatf | 2.93 |
| 6760794 | Thap4 | 2.93 |
| 6995393 | Rexo2 | 2.93 |
| 6871361 | Slc3a2 | 2.93 |
| 6971678 | Oat | 2.93 |
| 6983172 | Armc6 | 2.93 |
| 6854259 | Thoc6 | 2.93 |
| 6843953 | Gspt1 | 2.93 |
| 6962752 | Ndufc2 | 2.93 |
| 6876188 | Ptges2 | 2.93 |
| 6775758 | Hsp90b1 | 2.93 |
| 6882594 | 2010100O12Rik | 2.93 |
| 6916143 | Lrp8 | 2.92 |
| 6788020 | Canx | 2.92 |
| 6940005 | Nup54 | 2.92 |
| 6992022 | Topbp1 | 2.92 |
| 6788264 | Hspa4 | 2.92 |
| 6755207 | Slamf1 | 2.92 |
| 6764352 | Chml | 2.92 |
| 6883267 | Cse1l | 2.91 |
| 6854384 | Gfer | 2.91 |
| 6889760 | Tmem85 | 2.91 |
| 6790075 | Utp6 | 2.91 |
| 6900659 | Extl2 | 2.91 |
| 6776667 | Tmtc2 | 2.91 |
| 6906814 | Mtx1 | 2.91 |
| 6856262 | Mllt1 | 2.91 |
| 6767845 | Nus1 | 2.91 |
| 6926908 | Ubiad1 | 2.91 |
| 6983234 | Ccdc124 | 2.90 |
| 6882227 | Nsfl1c | 2.90 |
| 6867925 | 5730596K20Rik | 2.90 |
| 7014816 | Eif1ay | 2.90 |
| 6874626 | Sephs1 | 2.90 |
| 6970650 | Psma1 | 2.90 |
| 6892364 | Pigu | 2.90 |
| 6993864 | Elof1 | 2.89 |
| 6756625 | Mrpl15 | 2.89 |
| 6828822 | Gapdh | 2.89 |
| 6838924 | Nol12 | 2.89 |
| 6799849 | Bcap29 | 2.89 |
| 6954585 | Ptcd3 | 2.89 |
| 6997349 | Hmgn3 | 2.89 |
| 6815329 | Plp2 | 2.88 |
| 6897009 | Larp2 | 2.88 |
| 7013856 | Tmem35 | 2.88 |
| 6982158 | Slc25a4 | 2.88 |
| 6780544 | Med7 | 2.88 |
| 6834870 | Hrsp12 | 2.88 |
| 6866305 | Spire1 | 2.88 |
| 6949853 | Mrpl51 | 2.88 |
| 6899052 | Gpatch4 | 2.87 |
| 6913638 | Rad23b | 2.87 |
| 6939353 | 2610024G14Rik | 2.87 |
| 6908121 | Sars | 2.87 |
| 6966903 | Atf5 | 2.87 |
| 6970904 | Thumpd1 | 2.87 |
| 6927317 | Gltpd1 | 2.87 |
| 6941768 | Fbxl10 | 2.86 |
| 6911013 | Psrc1 | 2.86 |
| 6796784 | Ahsa1 | 2.86 |
| 6867748 | Eif1ad | 2.86 |
| 6775830 | 4930547N16Rik | 2.86 |
| 6885869 | 2900010J23Rik | 2.86 |
| 6749701 | Sgol2 | 2.86 |
| 6955604 | Mrps25 | 2.86 |
| 6775159 | Smarcb1 | 2.85 |
| 6877964 | Gorasp2 | 2.85 |
| 6810166 | Plk2 | 2.85 |
| 6866839 | Ccdc5 | 2.85 |
| 6885195 | Arhgap21 | 2.85 |
| 6955347 | Sec61a1 | 2.85 |
| 6837263 | Slc25a17 | 2.85 |
| 6966164 | Mrps12 | 2.85 |
| 6993000 | Deb1 | 2.85 |
| 6802041 | Rdh11 | 2.85 |
| 6983898 | Asna1 | 2.84 |
| 6916147 | Magoh | 2.84 |
| 6833138 | Tuba1c | 2.84 |
| 6812505 | Txndc5 | 2.84 |
| 7020162 | Gnl3l | 2.84 |
| 6839607 | Abcc1 | 2.84 |
| 6831994 | Atf4 | 2.84 |
| 6759642 | Mreg | 2.84 |
| 6963422 | Swap70 | 2.84 |
| 6907115 | Psmb4 | 2.84 |
| 6992430 | Tmem103 | 2.84 |
| 6983154 | Nme2 | 2.83 |
| 6888299 | Tfpi | 2.83 |
| 6810717 | Akr1e1 | 2.83 |
| 6837144 | Pdgfb | 2.83 |
| 6947562 | Ptges3 | 2.82 |
| 6784396 | Nmt1 | 2.82 |
| 6972990 | Ube2m | 2.82 |
| 6848553 | Tagap1 | 2.82 |
| 6952337 | Rbm28 | 2.82 |
| 6879016 | Psmc3 | 2.82 |
| 6871377 | Polr2g | 2.82 |
| 6854983 | H2 | 2.82 |
| 6869032 | Cd274 | 2.82 |
| 6753014 | Il10 | 2.82 |
| 6873469 | Pcgf6 | 2.82 |
| 6887902 | Mrpl23 | 2.81 |
| 6990216 | Anxa2 | 2.81 |
| 6837279 | St13 | 2.81 |
| 6944997 | Calu | 2.81 |
| 6867565 | Mrpl21 | 2.81 |
| 6747837 | Tmem70 | 2.81 |
| 6849626 | Pim1 | 2.81 |
| 6870025 | Pprc1 | 2.81 |
| 6998401 | Cdv3 | 2.80 |
| 6857810 | Lrpprc | 2.80 |
| 6940863 | Glmn | 2.80 |
| 6875961 | Mrps2 | 2.80 |
| 6759800 | Dnpep | 2.80 |
| 6965272 | Mrpl23 | 2.80 |
| 6795234 | Psma6 | 2.79 |
| 6908493 | Rtcd1 | 2.79 |
| 6947570 | Tprkb | 2.79 |
| 6887861 | Atp5g3 | 2.79 |
| 6875825 | Surf2 | 2.79 |
| 6902799 | Lrrc40 | 2.79 |
| 6873158 | Exosc1 | 2.79 |
| 6911176 | Sars | 2.79 |
| 6934401 | Aacs | 2.79 |
| 6992164 | Wdr51a | 2.79 |
| 7006641 | BC085271 | 2.79 |
| 6899353 | Ilf2 | 2.79 |
| 6887450 | Stk39 | 2.79 |
| 6789721 | Dph1 | 2.79 |
| 6916572 | Prdx1 | 2.78 |
| 6885652 | Gapdh | 2.78 |
| 6881008 | Mrps5 | 2.78 |
| 6814666 | Polr3g | 2.78 |
| 6857223 | LOC100048405 | 2.78 |
| 6972181 | Hras1 | 2.78 |
| 6788743 | Atpaf2 | 2.78 |
| 6847627 | Rwdd2b | 2.78 |
| 6926419 | D4Ertd22e | 2.78 |
| 6985006 | Slc12a4 | 2.78 |
| 6873744 | Xpnpep1 | 2.78 |
| 6957161 | Gapdh | 2.78 |
| 6850683 | Xpo5 | 2.78 |
| 6932598 | Mrpl1 | 2.77 |
| 6782127 | Med11 | 2.77 |
| 6970147 | 1500003O22Rik | 2.77 |
| 6909160 | Larp7 | 2.77 |
| 6917039 | Sf3a3 | 2.77 |
| 6955721 | Suclg2 | 2.77 |
| 6987132 | Josd3 | 2.77 |
| 6779359 | Cct4 | 2.77 |
| 6977761 | Gadd45gip1 | 2.77 |
| 7018225 | Pdk3 | 2.77 |
| 6989019 | Timm8b | 2.76 |
| 6954611 | Usp39 | 2.76 |
| 6993475 | Ankrd49 | 2.76 |
| 6907615 | Ttf2 | 2.76 |
| 6823221 | Comtd1 | 2.76 |
| 6824743 | Jub | 2.76 |
| 6769601 | Tyms | 2.76 |
| 6959585 | Nfkbid | 2.76 |
| 6896770 | Acad9 | 2.76 |
| 6854393 | Fahd1 | 2.76 |
| 6875792 | Pmpca | 2.76 |
| 6861175 | Prrc1 | 2.76 |
| 6965134 | Psmd13 | 2.76 |
| 6831859 | Gcat | 2.76 |
| 7011872 | 2610030H06Rik | 2.76 |
| 6974619 | Mrps31 | 2.75 |
| 6934976 | Polr2j | 2.75 |
| 6765716 | Lrp11 | 2.75 |
| 6973624 | Trim28 | 2.75 |
| 6837021 | Il2rb | 2.75 |
| 6963442 | Adm | 2.75 |
| 6756394 | Nek2 | 2.75 |
| 6989406 | Cox5a | 2.75 |
| 6891466 | Esf1 | 2.75 |
| 6854562 | Taf11 | 2.75 |
| 6971303 | Ccdc95 | 2.75 |
| 6897349 | Narg1 | 2.75 |
| 6877297 | Gpd2 | 2.75 |
| 6848504 | Snx9 | 2.74 |
| 6958436 | Tera | 2.74 |
| 6854398 | Nubp2 | 2.74 |
| 6925594 | Tmem39b | 2.74 |
| 6917506 | Med18 | 2.74 |
| 6906964 | Adrm1 | 2.74 |
| 6791151 | Kpnb1 | 2.74 |
| 6824634 | Supt16h | 2.74 |
| 6867728 | Yif1a | 2.74 |
| 6825429 | Elp3 | 2.74 |
| 6831647 | Exosc4 | 2.74 |
| 6782691 | Spag5 | 2.73 |
| 6911925 | Nbn | 2.73 |
| 6760915 | Hisppd1 | 2.73 |
| 6985361 | Mlkl | 2.73 |
| 6956764 | Zfp422 | 2.73 |
| 6814330 | Ndufs6 | 2.73 |
| 6897762 | Eif2a | 2.73 |
| 6964023 | Ubfd1 | 2.73 |
| 6771708 | 1110005A23Rik | 2.73 |
| 6867999 | Pcna | 2.73 |
| 6828862 | Rnasen | 2.73 |
| 6781321 | Mrpl55 | 2.73 |
| 6934308 | Setd8 | 2.73 |
| 6811290 | Vdac3 | 2.73 |
| 6963011 | Clpb | 2.72 |
| 6771309 | Avpr1a | 2.72 |
| 6838415 | Tuba1b | 2.72 |
| 6834728 | 3021401C12Rik | 2.72 |
| 6841882 | Mina | 2.72 |
| 6775098 | D10Ertd322e | 2.72 |
| 6951552 | Rpa3 | 2.72 |
| 6789939 | Traf4 | 2.72 |
| 6784317 | G6pc3 | 2.71 |
| 6986692 | Dcun1d5 | 2.71 |
| 6858344 | Sod2 | 2.71 |
| 6859953 | Kif20a | 2.71 |
| 6905620 | Ssr3 | 2.71 |
| 6755711 | Pycr2 | 2.71 |
| 6884138 | Adrm1 | 2.71 |
| 6838853 | Il2rb | 2.71 |
| 7014929 | Rbbp7 | 2.71 |
| 6965317 | Tssc4 | 2.70 |
| 6832775 | Pphln1 | 2.70 |
| 6816148 | Skiv2l2 | 2.70 |
| 6969811 | Mrpl48 | 2.70 |
| 7013342 | Apool | 2.70 |
| 6959817 | Ankrd27 | 2.70 |
| 6854347 | Ccnf | 2.69 |
| 6996440 | Rab8b | 2.69 |
| 6857310 | 2810405J04Rik | 2.69 |
| 6775310 | Rnf126 | 2.69 |
| 6770743 | Krr1 | 2.69 |
| 6792848 | 1110031I02Rik | 2.69 |
| 6956587 | Sec13 | 2.69 |
| 6956501 | Rad18 | 2.69 |
| 6980959 | Vdac3 | 2.69 |
| 6916748 | Slc2a1 | 2.68 |
| 6957261 | 9630033F20Rik | 2.68 |
| 6854971 | Angptl4 | 2.68 |
| 6957134 | Usp5 | 2.68 |
| 6890453 | Slc30a4 | 2.68 |
| 6891111 | Trmt6 | 2.68 |
| 6849326 | Mrps34 | 2.68 |
| 6762019 | Dars | 2.68 |
| 6849523 | Snrpc | 2.67 |
| 6858410 | Mrpl18 | 2.67 |
| 6935180 | 1110007L15Rik | 2.67 |
| 7013183 | Cox7b | 2.67 |
| 6997513 | Pgm3 | 2.67 |
| 6781248 | Igtp | 2.67 |
| 6979519 | Usp10 | 2.67 |
| 6959448 | Sars2 | 2.67 |
| 7010667 | Slc25a5 | 2.67 |
| 6758943 | Ppil3 | 2.67 |
| 6952168 | Ndufa5 | 2.67 |
| 6874542 | Nmt2 | 2.67 |
| 6789351 | Nlgn2 | 2.67 |
| 6882666 | Rpn2 | 2.67 |
| 6831849 | Pdxp | 2.67 |
| 6824007 | Ghitm | 2.67 |
| 6907912 | Ddx20 | 2.66 |
| 6923317 | Gapdh | 2.66 |
| 6783359 | Sfrs1 | 2.66 |
| 6781487 | Map2k3 | 2.66 |
| 6916762 | Lepre1 | 2.66 |
| 6845471 | Gtf2e1 | 2.66 |
| 6841865 | Cldnd1 | 2.66 |
| 6898775 | Plrg1 | 2.66 |
| 6909153 | Larp7 | 2.66 |
| 6970846 | Gde1 | 2.66 |
| 6782129 | Tm4sf5 | 2.66 |
| 6989320 | Ube2s | 2.66 |
| 6779688 | Pnpt1 | 2.66 |
| 6959409 | Eid2 | 2.66 |
| 6929149 | Pmpcb | 2.66 |
| 6749852 | Icos | 2.66 |
| 6941934 | Scarb1 | 2.66 |
| 6917124 | Mrps15 | 2.66 |
| 6922241 | Alad | 2.66 |
| 6800082 | Bzw2 | 2.65 |
| 6836842 | Nfkbil2 | 2.65 |
| 6846575 | A930013N22Rik | 2.65 |
| 6969017 | 2210412D01Rik | 2.65 |
| 6933602 | Mvk | 2.64 |
| 6767155 | Hdac2 | 2.64 |
| 7009797 | Timm17b | 2.64 |
| 6784553 | Mettl2 | 2.64 |
| 6992426 | ENSMUSG00000074056 | 2.64 |
| 6960562 | Zdhhc13 | 2.64 |
| 6983879 | Calr | 2.64 |
| 6990842 | 4930486G11Rik | 2.64 |
| 6868048 | Ddb1 | 2.64 |
| 6775370 | Mbd3 | 2.64 |
| 6867973 | 1810009A15Rik | 2.64 |
| 6759613 | Bard1 | 2.64 |
| 6919320 | Penk1 | 2.64 |
| 6930883 | Tbc1d19 | 2.63 |
| 6841187 | Btla | 2.63 |
| 6987107 | Mre11a | 2.63 |
| 6947558 | Alms1 | 2.63 |
| 6764056 | Ufc1 | 2.63 |
| 6869561 | Tmem20 | 2.63 |
| 6861774 | Rnmt | 2.63 |
| 6919200 | C1qdc2 | 2.63 |
| 6953318 | Tarbp2 | 2.63 |
| 6993198 | Aasdhppt | 2.63 |
| 6769192 | Atp5d | 2.63 |
| 6980124 | Timm44 | 2.62 |
| 6781516 | B9d1 | 2.62 |
| 6980990 | Agpat6 | 2.62 |
| 6792760 | Nploc4 | 2.62 |
| 6912098 | Coq3 | 2.62 |
| 6912594 | Rars2 | 2.62 |
| 7018756 | Magt1 | 2.62 |
| 6894580 | Tm9sf4 | 2.62 |
| 6995825 | Tspan3 | 2.62 |
| 6945624 | Ndufb2 | 2.62 |
| 6789328 | Senp3 | 2.62 |
| 6755672 | Parp1 | 2.62 |
| 6956981 | Bid | 2.62 |
| 6869035 | Pdcd1lg2 | 2.61 |
| 6900990 | Gclm | 2.61 |
| 6929960 | Grpel1 | 2.61 |
| 6989354 | Imp3 | 2.61 |
| 6824834 | Nedd8 | 2.61 |
| 6893486 | Aurka | 2.61 |
| 7018041 | Gyk | 2.61 |
| 6946138 | Mpp6 | 2.60 |
| 6785591 | Drg1 | 2.60 |
| 6876053 | Nup188 | 2.60 |
| 6897337 | Ccrn4l | 2.60 |
| 6837301 | Rangap1 | 2.60 |
| 6934217 | Psmd9 | 2.60 |
| 6916219 | Kti12 | 2.60 |
| 6881222 | Itpa | 2.60 |
| 6818687 | Mapk1ip1l | 2.60 |
| 6887520 | Spc25 | 2.60 |
| 6989045 | Ppp2r1b | 2.60 |
| 6840517 | Ncbp2 | 2.60 |
| 6840782 | Polq | 2.60 |
| 6820288 | Esd | 2.60 |
| 6844443 | Parl | 2.60 |
| 6760714 | Ndufa10 | 2.60 |
| 6899033 | Isg20l2 | 2.60 |
| 6751082 | Hrb | 2.59 |
| 6896857 | Spata5 | 2.59 |
| 6989985 | Ppib | 2.59 |
| 6882363 | Tm9sf4 | 2.59 |
| 6986211 | Rbm34 | 2.59 |
| 7018249 | Eif2s3x | 2.59 |
| 6878995 | Mtch2 | 2.59 |
| 6921020 | Ubap2 | 2.59 |
| 6934662 | 0610007L01Rik | 2.59 |
| 6777296 | Cct2 | 2.59 |
| 6763025 | Cox7b | 2.59 |
| 6843487 | Wrb | 2.59 |
| 6836959 | Apol7c | 2.59 |
| 7019867 | Acsl4 | 2.59 |
| 6942523 | Ap1s1 | 2.59 |
| 6884460 | Cdc123 | 2.59 |
| 6890835 | Ckap2l | 2.59 |
| 6961203 | H47 | 2.59 |
| 6924959 | Ybx1 | 2.59 |
| 6921029 | Kif24 | 2.58 |
| 6974480 | Mcph1 | 2.58 |
| 7010327 | Phf16 | 2.58 |
| 6976988 | Cope | 2.58 |
| 6789941 | Nek8 | 2.58 |
| 6963856 | 6330503K22Rik | 2.58 |
| 6950770 | Golt1b | 2.58 |
| 6916530 | Lrrc41 | 2.58 |
| 6779373 | Xpo1 | 2.58 |
| 6975247 | Gtf2e2 | 2.58 |
| 6803862 | Xrcc3 | 2.58 |
| 6909648 | Nfkb1 | 2.57 |
| 6807899 | Fastkd3 | 2.57 |
| 6892376 | Gss | 2.57 |
| 6775561 | Aldh1l2 | 2.57 |
| 6998564 | Alas1 | 2.57 |
| 6751698 | Sept2 | 2.57 |
| 6771578 | Tmem194 | 2.57 |
| 6833408 | Myg1 | 2.57 |
| 6953339 | Pdia4 | 2.57 |
| 6905366 | Siah2 | 2.57 |
| 6788702 | Cops3 | 2.57 |
| 6874212 | Eif3s10 | 2.57 |
| 6855116 | Tcf19 | 2.56 |
| 7015463 | Ftsj1 | 2.56 |
| 6858253 | Mrpl18 | 2.56 |
| 6849467 | Bnip1 | 2.56 |
| 6864678 | Pfdn1 | 2.56 |
| 6798946 | Gen1 | 2.56 |
| 6829964 | Wdsof1 | 2.56 |
| 6885489 | Agpat2 | 2.56 |
| 6778055 | Cdk2 | 2.56 |
| 6799836 | Dld | 2.55 |
| 6946365 | Plekha8 | 2.55 |
| 6966985 | Bax | 2.55 |
| 6751505 | Cops8 | 2.55 |
| 6855610 | Hsp90ab1 | 2.55 |
| 6871771 | Cep78 | 2.55 |
| 6905192 | Exosc8 | 2.55 |
| 6867197 | Cndp2 | 2.55 |
| 7009783 | Praf2 | 2.55 |
| 6764011 | Uap1 | 2.54 |
| 6933678 | Gcn1l1 | 2.54 |
| 6970139 | Arfip2 | 2.54 |
| 6789326 | Eif4a1 | 2.54 |
| 7019488 | Tspan6 | 2.54 |
| 6824962 | Xpo4 | 2.54 |
| 6938698 | Rfc1 | 2.54 |
| 6775335 | Polr2e | 2.54 |
| 6871511 | Cd6 | 2.54 |
| 6855149 | Abcf1 | 2.54 |
| 6853388 | 5730437N04Rik | 2.54 |
| 6817416 | Vdac2 | 2.54 |
| 6957249 | Ndufa9 | 2.54 |
| 6886639 | 2010311D03Rik | 2.54 |
| 6942192 | Gusb | 2.53 |
| 6900734 | Frrs1 | 2.53 |
| 6899221 | Krtcap2 | 2.53 |
| 6792820 | Dus1l | 2.53 |
| 6789471 | Nup88 | 2.53 |
| 6979709 | Afg3l1 | 2.53 |
| 6823768 | Dph3 | 2.53 |
| 6978953 | Cyb5b | 2.53 |
| 6762311 | Snrpe | 2.53 |
| 6951684 | Ndufa4 | 2.53 |
| 6778068 | Ormdl2 | 2.53 |
| 6921015 | Nol6 | 2.53 |
| 6817367 | Chchd1 | 2.53 |
| 7019923 | Ammecr1 | 2.53 |
| 6992093 | Mrpl3 | 2.53 |
| 6837335 | D15Wsu75e | 2.53 |
| 6824670 | Psma3 | 2.52 |
| 6807169 | 4732471D19Rik | 2.52 |
| 6859059 | Mrpl27 | 2.52 |
| 6996935 | Mapk6 | 2.52 |
| 6747309 | Tcea1 | 2.52 |
| 6957263 | Ccnd2 | 2.52 |
| 6840819 | Lrrc58 | 2.52 |
| 6885506 | Surf4 | 2.52 |
| 6839956 | Eif4g1 | 2.52 |
| 6885378 | Tubb2c | 2.52 |
| 6764093 | Slamf7 | 2.52 |
| 6870967 | Ndufv1 | 2.52 |
| 6789974 | Tnfaip1 | 2.52 |
| 6871374 | Taf6l | 2.52 |
| 6919193 | Cpsf3l | 2.52 |
| 6840491 | Bdh1 | 2.51 |
| 6819573 | Mipep | 2.51 |
| 6847517 | Mrpl39 | 2.51 |
| 6883641 | Rae1 | 2.51 |
| 6768014 | Gja1 | 2.51 |
| 6957640 | Dusp16 | 2.51 |
| 6997632 | Syncrip | 2.51 |
| 6870172 | 6330577E15Rik | 2.51 |
| 6792699 | Eif4a3 | 2.51 |
| 6755729 | Cnih4 | 2.51 |
| 6952419 | Zc3hc1 | 2.51 |
| 6946948 | Immt | 2.51 |
| 6939266 | Ube2n | 2.51 |
| 6761615 | Tsn | 2.51 |
| 6782515 | Timm22 | 2.51 |
| 6857022 | Tgif1 | 2.50 |
| 6960382 | Bcat2 | 2.50 |
| 6860436 | Tcerg1 | 2.50 |
| 6926950 | Nmnat1 | 2.50 |
| 6840677 | Ccdc14 | 2.50 |
| 6769244 | Dot1l | 2.50 |
| 6884352 | Hspa14 | 2.50 |
| 6766038 | Sf3b5 | 2.50 |
| 6789813 | Nxn | 2.50 |
| 6792965 | Rbj | 2.50 |
| 7017630 | G6pdx | 2.50 |
| 6924387 | Prpf38a | 2.50 |
| 6883722 | Npepl1 | 2.50 |
| 6823002 | 2700060E02Rik | 2.50 |
| 6849992 | Wdr46 | 2.50 |
| 6921913 | Ikbkap | 2.49 |
| 6936621 | Pkm2 | 2.49 |
| 6934310 | 6330548G22Rik | 2.49 |
| 6955698 | Lrig1 | 2.49 |
| 6811554 | Prss16 | 2.49 |
| 6987381 | Qtrt1 | 2.49 |
| 6778463 | Xbp1 | 2.49 |
| 7015403 | Plp2 | 2.49 |
| 6782959 | Lig3 | 2.49 |
| 6868021 | Fads1 | 2.49 |
| 6757988 | Txndc9 | 2.49 |
| 6852389 | 2410091C18Rik | 2.49 |
| 6859471 | Elp2 | 2.49 |
| 6897592 | Spg20 | 2.49 |
| 6977756 | Btbd14b | 2.49 |
| 6753394 | Phlda3 | 2.49 |
| 6800913 | Polr2h | 2.49 |
| 6950334 | Etv6 | 2.49 |
| 6754893 | Aldh9a1 | 2.49 |
| 6905588 | Slc33a1 | 2.49 |
| 6811140 | Mrpl32 | 2.48 |
| 6841862 | Cpox | 2.48 |
| 6791150 | Cdk5rap3 | 2.48 |
| 6884710 | Rbm17 | 2.48 |
| 6937333 | Mfsd10 | 2.48 |
| 6923391 | Plaa | 2.48 |
| 6775449 | Mrpl54 | 2.48 |
| 6932878 | Mrps18c | 2.48 |
| 6838417 | Tuba1a | 2.48 |
| 6894490 | 2010100O12Rik | 2.48 |
| 6948643 | Ube2v2 | 2.48 |
| 6993104 | Exosc7 | 2.48 |
| 6808680 | Ccnh | 2.47 |
| 6792554 | Sfrs2 | 2.47 |
| 6836646 | Sf3b4 | 2.47 |
| 6793498 | 4933425L03Rik | 2.47 |
| 6850594 | Supt3h | 2.47 |
| 6926936 | Pgd | 2.47 |
| 6977796 | BC056474 | 2.47 |
| 6843928 | Txndc11 | 2.47 |
| 6804033 | Wdr60 | 2.47 |
| 6933037 | Nudt9 | 2.47 |
| 6972205 | Lrdd | 2.47 |
| 6845559 | 4930455C21Rik | 2.47 |
| 6924892 | Cdc20 | 2.47 |
| 6758325 | Uxs1 | 2.47 |
| 6857963 | Pigf | 2.47 |
| 6954994 | Htra2 | 2.47 |
| 6988706 | Mpzl2 | 2.47 |
| 6819247 | Pck2 | 2.47 |
| 6988773 | Zfp259 | 2.47 |
| 6940883 | Tmed5 | 2.46 |
| 6813963 | Uqcrb | 2.46 |
| 6955228 | Anxa4 | 2.46 |
| 6790621 | Msi2 | 2.46 |
| 6937073 | Ppm1g | 2.46 |
| 6832086 | Sgsm3 | 2.46 |
| 6831648 | Gpaa1 | 2.46 |
| 6921284 | Exosc3 | 2.46 |
| 6757129 | Tceb1 | 2.46 |
| 6985106 | Nqo1 | 2.46 |
| 6769150 | Ptbp1 | 2.46 |
| 6818053 | Oxnad1 | 2.46 |
| 6896609 | Actl6a | 2.46 |
| 6762355 | Tmem183a | 2.46 |
| 6788120 | Hnrpab | 2.46 |
| 6952529 | Chchd3 | 2.45 |
| 6997990 | Chst2 | 2.45 |
| 7012845 | Dlg3 | 2.45 |
| 6841849 | Dcbld2 | 2.45 |
| 6937069 | Eif2b4 | 2.45 |
| 6993722 | Dnmt1 | 2.45 |
| 6925590 | Txlna | 2.45 |
| 6956886 | Bms1 | 2.45 |
| 6989355 | Snupn | 2.45 |
| 6831891 | Maff | 2.45 |
| 6796699 | Batf | 2.45 |
| 6871199 | Arl2 | 2.45 |
| 6810317 | Dhx29 | 2.44 |
| 6925081 | Zmpste24 | 2.44 |
| 6761998 | Bnip3l | 2.44 |
| 6916712 | Elovl1 | 2.44 |
| 6970851 | 2310008H09Rik | 2.44 |
| 6772231 | Pex3 | 2.44 |
| 6844334 | Comt | 2.44 |
| 6899144 | Ubqln4 | 2.44 |
| 6813110 | Cenpp | 2.44 |
| 6930864 | Rbpj | 2.44 |
| 6946800 | Sod1 | 2.44 |
| 6934631 | Mrps17 | 2.44 |
| 6969770 | Spcs2 | 2.44 |
| 6968781 | Furin | 2.44 |
| 6935094 | Zcwpw1 | 2.44 |
| 6939664 | Uba6 | 2.43 |
| 6989042 | D630004A14Rik | 2.43 |
| 6838753 | Atp5g2 | 2.43 |
| 6755975 | Bpnt1 | 2.43 |
| 6777614 | Tmem5 | 2.43 |
| 6964370 | Bckdk | 2.43 |
| 6926871 | Chchd2 | 2.43 |
| 6983686 | Smarca5 | 2.43 |
| 6934650 | Crcp | 2.43 |
| 6815726 | Sdccag10 | 2.43 |
| 6977795 | Dhps | 2.43 |
| 6910279 | Bxdc5 | 2.43 |
| 6976498 | Nek1 | 2.43 |
| 6951247 | 3010003L21Rik | 2.43 |
| 6824850 | 2310014G06Rik | 2.43 |
| 6769366 | Chst11 | 2.43 |
| 6781247 | Mrpl22 | 2.43 |
| 6795025 | Hmgb1 | 2.43 |
| 6792296 | Slc39a11 | 2.43 |
| 6759816 | Chpf | 2.43 |
| 6811656 | Abt1 | 2.42 |
| 6832117 | L3mbtl2 | 2.42 |
| 6851320 | Trip10 | 2.42 |
| 6825688 | Slc39a14 | 2.42 |
| 6848511 | Gtf2h5 | 2.42 |
| 6798640 | Ubxd4 | 2.42 |
| 6843088 | Sod1 | 2.42 |
| 6833430 | Tarbp2 | 2.42 |
| 6907313 | Gpr89 | 2.42 |
| 6763520 | Dars2 | 2.42 |
| 6978822 | E2f4 | 2.42 |
| 6749817 | Abi2 | 2.42 |
| 6922324 | Tnfsf8 | 2.42 |
| 7021014 | Tspan6 | 2.42 |
| 7017014 | Tmem32 | 2.42 |
| 6895959 | Armc1 | 2.42 |
| 6849098 | BC049807 | 2.42 |
| 6815538 | Gtf2h2 | 2.42 |
| 6790087 | 1110002N22Rik | 2.42 |
| 6893558 | Pmepa1 | 2.41 |
| 6859451 | 2700062C07Rik | 2.41 |
| 6941344 | Cit | 2.41 |
| 6940911 | Atp5k | 2.41 |
| 6879938 | Ccdc34 | 2.41 |
| 7011259 | Rps2 | 2.41 |
| 6983584 | Lsm6 | 2.41 |
| 6961895 | Isg20l1 | 2.41 |
| 6925156 | Ppie | 2.41 |
| 7010334 | Rbm10 | 2.41 |
| 6901745 | Adh4 | 2.41 |
| 6867849 | Ehd1 | 2.41 |
| 6767235 | Tube1 | 2.41 |
| 6984526 | Gtl3 | 2.41 |
| 6987580 | Sept7 | 2.41 |
| 6925974 | Dhdds | 2.40 |
| 6812320 | Lyrm4 | 2.40 |
| 6756383 | Ints7 | 2.40 |
| 6920786 | Mobkl2b | 2.40 |
| 6810051 | Ercc8 | 2.40 |
| 6835104 | Azin1 | 2.40 |
| 6854340 | Tbc1d24 | 2.40 |
| 6934891 | Abhd11 | 2.40 |
| 6999549 | Higd1a | 2.40 |
| 6902976 | Pex2 | 2.40 |
| 6906742 | Pmf1 | 2.40 |
| 6999114 | Crtap | 2.40 |
| 6957162 | Ncapd2 | 2.40 |
| 6905145 | Ufm1 | 2.40 |
| 6985851 | Cox4nb | 2.39 |
| 6854522 | 2900010M23Rik | 2.39 |
| 6985886 | Klhdc4 | 2.39 |
| 7015058 | Gemin8 | 2.39 |
| 6994585 | Dcps | 2.39 |
| 6907205 | Tars2 | 2.39 |
| 7017578 | Uchl5ip | 2.39 |
| 6935043 | Plod3 | 2.39 |
| 6926655 | Tnfrsf8 | 2.39 |
| 6934397 | Bri3bp | 2.39 |
| 6982155 | Snx25 | 2.39 |
| 6991760 | Faim | 2.39 |
| 6883526 | Pfdn4 | 2.39 |
| 6867222 | 1700034H14Rik | 2.39 |
| 6985642 | Cdyl2 | 2.39 |
| 6990526 | Arpp19 | 2.39 |
| 6832142 | Aco2 | 2.38 |
| 6855134 | Tubb5 | 2.38 |
| 6778420 | Thoc5 | 2.38 |
| 6836981 | Foxred2 | 2.38 |
| 6942413 | Wbscr22 | 2.38 |
| 6748726 | Lipt1 | 2.38 |
| 7011264 | Hprt1 | 2.38 |
| 6755174 | Pfdn2 | 2.38 |
| 6866660 | 2810433K01Rik | 2.38 |
| 6919748 | 6720467C03Rik | 2.38 |
| 6858247 | Acat2 | 2.38 |
| 6892418 | Nfs1 | 2.38 |
| 6912581 | Chchd2 | 2.38 |
| 6873282 | Ndufb8 | 2.38 |
| 6824286 | Cnih | 2.38 |
| 6991718 | Copb2 | 2.38 |
| 6934942 | Mdh2 | 2.38 |
| 6882172 | Cst7 | 2.37 |
| 7019519 | Gla | 2.37 |
| 6983893 | Rnaseh2a | 2.37 |
| 6795887 | Psma3 | 2.37 |
| 6783810 | Snf8 | 2.37 |
| 6971548 | Rfwd3 | 2.37 |
| 6872256 | Fxn | 2.37 |
| 6952426 | Tmem209 | 2.37 |
| 6854465 | Snrpg | 2.37 |
| 6886017 | Phf19 | 2.37 |
| 6769657 | Arl1 | 2.37 |
| 6908800 | Abcd3 | 2.37 |
| 6775741 | D10Wsu52e | 2.37 |
| 6955215 | Pcbp1 | 2.37 |
| 6818939 | Parp2 | 2.37 |
| 6995083 | Trappc4 | 2.37 |
| 6942941 | AU022870 | 2.37 |
| 6937190 | Pisd | 2.37 |
| 6861746 | Seh1l | 2.36 |
| 6786666 | 0610010F05Rik | 2.36 |
| 6980126 | Elavl1 | 2.36 |
| 6806618 | Nol7 | 2.36 |
| 6960760 | Mrps21 | 2.36 |
| 6773432 | Slc16a10 | 2.36 |
| 6816371 | Mrps30 | 2.36 |
| 6853438 | Atp5g2 | 2.36 |
| 7018847 | Itm2a | 2.36 |
| 6900993 | Dnttip2 | 2.36 |
| 6785220 | 1110014K08Rik | 2.36 |
| 6901187 | 4930422G04Rik | 2.36 |
| 6869370 | Kif20b | 2.36 |
| 6871058 | Bbs1 | 2.36 |
| 6977669 | Ndufb7 | 2.36 |
| 6924793 | Tmem69 | 2.36 |
| 6977073 | Mrpl34 | 2.36 |
| 6817944 | Sfmbt1 | 2.36 |
| 6918127 | Sdhb | 2.36 |
| 6901316 | Elovl6 | 2.35 |
| 6753033 | Lgtn | 2.35 |
| 6830770 | Trib1 | 2.35 |
| 6760802 | Dtymk | 2.35 |
| 6782005 | A030009H04Rik | 2.35 |
| 6873421 | Arl3 | 2.35 |
| 6850092 | Vars | 2.35 |
| 6830131 | Ttc35 | 2.35 |
| 6863973 | Slc39a6 | 2.35 |
| 6907346 | Chd1l | 2.35 |
| 6768227 | Lrrc20 | 2.35 |
| 6817956 | Nek4 | 2.35 |
| 6972227 | Chid1 | 2.35 |
| 6839847 | Gnb1l | 2.35 |
| 6942492 | Prkrip1 | 2.35 |
| 6854284 | Tceb2 | 2.35 |
| 6976560 | Cbr4 | 2.35 |
| 6866354 | 4933403F05Rik | 2.35 |
| 6832295 | Samm50 | 2.34 |
| 6967480 | Mrps33 | 2.34 |
| 6980991 | Gins4 | 2.34 |
| 6789445 | Spag7 | 2.34 |
| 6840699 | Ptplb | 2.34 |
| 6885463 | Qsox2 | 2.34 |
| 6781440 | 4933439F18Rik | 2.34 |
| 6966990 | Nucb1 | 2.34 |
| 6933662 | Sfrs9 | 2.34 |
| 6966267 | Wdr62 | 2.34 |
| 6759732 | Tmbim1 | 2.34 |
| 6963693 | 1110004F10Rik | 2.34 |
| 6769110 | Olfr1352 | 2.34 |
| 6804703 | U2af2 | 2.34 |
| 6785236 | 1810032O08Rik | 2.34 |
| 6857512 | Hnrpll | 2.34 |
| 6962111 | Eftud1 | 2.33 |
| 6923155 | Mrpl48 | 2.33 |
| 6812321 | Tmed10 | 2.33 |
| 6804938 | Psma2 | 2.33 |
| 6985253 | Dhodh | 2.33 |
| 6813246 | Nfil3 | 2.33 |
| 6899025 | Sh2d2a | 2.33 |
| 6955432 | Chchd6 | 2.33 |
| 6916171 | 2010305A19Rik | 2.33 |
| 6901747 | Adh5 | 2.33 |
| 6836784 | Tsta3 | 2.33 |
| 6837835 | Rabl2a | 2.33 |
| 6946954 | St3gal5 | 2.33 |
| 6754227 | Acbd6 | 2.33 |
| 6785307 | Birc5 | 2.33 |
| 6778375 | Sf3a1 | 2.33 |
| 6866299 | Afg3l2 | 2.33 |
| 6999130 | Ccr4 | 2.32 |
| 6888153 | Nckap1 | 2.32 |
| 6879489 | Traf6 | 2.32 |
| 6871509 | Cd5 | 2.32 |
| 6858784 | Thoc1 | 2.32 |
| 6985373 | Wdr59 | 2.32 |
| 6912820 | Aco1 | 2.32 |
| 6984536 | Csnk2a2 | 2.32 |
| 6955223 | 2610209M04Rik | 2.32 |
| 6808214 | Clptm1l | 2.32 |
| 6867917 | 2700081O15Rik | 2.32 |
| 6782016 | Lsmd1 | 2.32 |
| 6920725 | Orc3l | 2.32 |
| 6982690 | Sap30 | 2.32 |
| 6876010 | Med27 | 2.32 |
| 6892401 | Uqcc | 2.32 |
| 6881820 | 8430406I07Rik | 2.32 |
| 6848580 | Brp44l | 2.32 |
| 7018304 | Las1l | 2.32 |
| 6834744 | Ube2v2 | 2.32 |
| 6960488 | Gtf2h1 | 2.32 |
| 6884305 | Polr3k | 2.31 |
| 6854405 | Telo2 | 2.31 |
| 6992377 | Uqcrc1 | 2.31 |
| 6769996 | Ube2n | 2.31 |
| 6900456 | Vav3 | 2.31 |
| 7011052 | Rbmx2 | 2.31 |
| 6879659 | Cstf3 | 2.31 |
| 7014702 | Acot10 | 2.31 |
| 6901592 | Cenpe | 2.31 |
| 6901695 | H2afz | 2.31 |
| 6781969 | 1500010J02Rik | 2.31 |
| 6966423 | Uba2 | 2.31 |
| 6856695 | Ndufv2 | 2.31 |
| 6765222 | Nenf | 2.31 |
| 6949826 | Mlf2 | 2.31 |
| 6873897 | Zdhhc6 | 2.31 |
| 6815698 | Nln | 2.31 |
| 6933459 | Chek2 | 2.31 |
| 6918998 | Nol9 | 2.30 |
| 6908510 | Slc35a3 | 2.30 |
| 6845044 | Lsg1 | 2.30 |
| 6850661 | Mrpl14 | 2.30 |
| 6979706 | Def8 | 2.30 |
| 6972110 | Tubgcp2 | 2.30 |
| 6789973 | Tmem199 | 2.30 |
| 6898932 | Higd1a | 2.30 |
| 6839839 | Txnrd2 | 2.30 |
| 6778410 | Ascc2 | 2.30 |
| 6825223 | 6330409N04Rik | 2.30 |
| 6993305 | 9230110C19Rik | 2.30 |
| 6838036 | Zcrb1 | 2.30 |
| 6988098 | Srpr | 2.30 |
| 6801929 | Max | 2.30 |
| 6919339 | Impad1 | 2.30 |
| 6798229 | 2810002N01Rik | 2.30 |
| 6927341 | B3galt6 | 2.30 |
| 6808810 | Tmem167 | 2.30 |
| 6790015 | Ksr1 | 2.30 |
| 6847819 | 1110004E09Rik | 2.30 |
| 6987403 | Ldlr | 2.30 |
| 6983869 | Btbd14b | 2.29 |
| 6960622 | Prmt3 | 2.29 |
| 6783367 | Mrps23 | 2.29 |
| 6961917 | Fanci | 2.29 |
| 7020507 | Pdha1 | 2.29 |
| 6975904 | Mlf1ip | 2.29 |
| 6782701 | Slc46a1 | 2.29 |
| 6844184 | 2410018G20Rik | 2.29 |
| 6838469 | Racgap1 | 2.29 |
| 6963384 | D930014E17Rik | 2.29 |
| 6749103 | Bivm | 2.29 |
| 6937106 | Rbks | 2.29 |
| 6925837 | Ppp1r8 | 2.29 |
| 6941186 | Coro1c | 2.29 |
| 6985343 | Ddx19b | 2.28 |
| 6987378 | Ilf3 | 2.28 |
| 6788993 | Cox10 | 2.28 |
| 6762413 | Rnpep | 2.28 |
| 6968647 | Mrpl46 | 2.28 |
| 6794059 | Rnaseh1 | 2.28 |
| 6803775 | Brp44l | 2.28 |
| 6954626 | Mat2a | 2.28 |
| 6763572 | Fasl | 2.28 |
| 6768234 | Sar1a | 2.27 |
| 6812035 | Uqcrfs1 | 2.27 |
| 6880513 | Dll4 | 2.27 |
| 6847928 | Atp5o | 2.27 |
| 6884466 | Dhtkd1 | 2.27 |
| 6948918 | Edem1 | 2.27 |
| 6770722 | Phlda1 | 2.27 |
| 7017595 | Idh3g | 2.27 |
| 6880983 | Stard7 | 2.27 |
| 7015101 | Rpl7a | 2.27 |
| 6771655 | Ankrd52 | 2.27 |
| 6835149 | Slc25a32 | 2.27 |
| 6797474 | Cpsf2 | 2.27 |
| 6768866 | Snrpd3 | 2.27 |
| 6758663 | Asnsd1 | 2.27 |
| 6992864 | Ctdspl | 2.27 |
| 6992347 | Qars | 2.27 |
| 6931261 | Lias | 2.27 |
| 6868519 | D030056L22Rik | 2.26 |
| 6822949 | Ube2e1 | 2.26 |
| 6929648 | 1110039B18Rik | 2.26 |
| 6876044 | Slc39a1 | 2.26 |
| 6934315 | Tmed2 | 2.26 |
| 6787176 | Npm1 | 2.26 |
| 6791139 | Nfe2l1 | 2.26 |
| 6890302 | Zfp106 | 2.26 |
| 6838926 | Pdxp | 2.26 |
| 6915567 | Hook1 | 2.26 |
| 6802749 | Sel1l | 2.26 |
| 6995718 | Cul5 | 2.26 |
| 6823460 | E430028B21Rik | 2.26 |
| 6815551 | Mrps36 | 2.26 |
| 6906894 | Ints3 | 2.26 |
| 6916996 | Mycbp | 2.25 |
| 6882878 | Top1 | 2.25 |
| 6751687 | Ppp1r7 | 2.25 |
| 6917078 | 2310005N01Rik | 2.25 |
| 6916535 | Pomgnt1 | 2.25 |
| 6888968 | Api5 | 2.25 |
| 6915791 | Pgm1 | 2.25 |
| 6985330 | Sf3b3 | 2.25 |
| 6871295 | Otub1 | 2.25 |
| 6777879 | Xrcc6bp1 | 2.25 |
| 6963967 | Polr3e | 2.25 |
| 6771647 | Cs | 2.25 |
| 6844329 | Dgcr8 | 2.25 |
| 6998930 | Mlh1 | 2.25 |
| 6773067 | Trmt11 | 2.25 |
| 6791626 | Ccdc43 | 2.25 |
| 6888029 | BC003993 | 2.25 |
| 6968871 | Hdgfrp3 | 2.25 |
| 6892102 | Psmf1 | 2.24 |
| 6775410 | Timm13 | 2.24 |
| 6796597 | Coq6 | 2.24 |
| 6792945 | Dnmt3a | 2.24 |
| 7018297 | AK129302 | 2.24 |
| 6992918 | Slc25a38 | 2.24 |
| 6876047 | Endog | 2.24 |
| 6989904 | 2010321M09Rik | 2.24 |
| 6838256 | Slc38a1 | 2.24 |
| 6946102 | D330028D13Rik | 2.24 |
| 6826219 | Tnfsf11 | 2.23 |
| 6792502 | Srp68 | 2.23 |
| 6791856 | Cyb561 | 2.23 |
| 6771623 | Ptges3 | 2.23 |
| 6931375 | Tmem33 | 2.23 |
| 6844542 | Tmem41a | 2.23 |
| 6927963 | Prpf4 | 2.23 |
| 6775257 | Rrp1 | 2.23 |
| 6966302 | Cox6b1 | 2.23 |
| 6962762 | Ints4 | 2.23 |
| 6919741 | ENSMUSG00000073995 | 2.23 |
| 6926928 | Pex14 | 2.23 |
| 6913970 | Prpf4 | 2.23 |
| 6850723 | Mea1 | 2.23 |
| 6775258 | Pdxk | 2.23 |
| 6917595 | Gpn2 | 2.23 |
| 6791644 | 3000004C01Rik | 2.23 |
| 6843652 | Magmas | 2.22 |
| 6844176 | Ube2v2 | 2.22 |
| 6853248 | Tfb1m | 2.22 |
| 6848572 | Fgfr1op | 2.22 |
| 6972118 | Echs1 | 2.22 |
| 6833503 | Copz1 | 2.22 |
| 6892852 | Ada | 2.22 |
| 6792097 | Slc16a6 | 2.22 |
| 6922537 | Ywhaq | 2.22 |
| 6755540 | 5830417C01Rik | 2.22 |
| 6776029 | Nedd1 | 2.21 |
| 6918029 | Tmco4 | 2.21 |
| 6871021 | Gm960 | 2.21 |
| 6809522 | Serf1 | 2.21 |
| 6944432 | Lsm8 | 2.21 |
| 6949095 | Creld1 | 2.21 |
| 6979684 | Rps12 | 2.21 |
| 6966229 | BC027344 | 2.21 |
| 6828005 | Tmtc4 | 2.21 |
| 6769245 | Sf3a2 | 2.21 |
| 6912092 | Ccnc | 2.21 |
| 6907126 | Psmd4 | 2.21 |
| 6797707 | Glrx5 | 2.21 |
| 6882630 | 1110008F13Rik | 2.21 |
| 6837445 | Pacsin2 | 2.21 |
| 6918337 | Mcrs1 | 2.20 |
| 6758588 | Obfc2a | 2.20 |
| 6829647 | Mtdh | 2.20 |
| 6790508 | Tmem49 | 2.20 |
| 6834558 | BC087945 | 2.20 |
| 6944385 | St7 | 2.20 |
| 6834729 | 5730557B15Rik | 2.20 |
| 6922883 | Zdhhc21 | 2.20 |
| 6993131 | Sacm1l | 2.20 |
| 6936693 | Tomm7 | 2.20 |
| 6936082 | Dbf4 | 2.20 |
| 6849294 | 1700012G19Rik | 2.19 |
| 6883601 | Cstf1 | 2.19 |
| 6779588 | Fancl | 2.19 |
| 6781083 | Hint1 | 2.19 |
| 6964635 | 2310007H09Rik | 2.19 |
| 6962107 | Sh3gl3 | 2.19 |
| 6796560 | 2410016O06Rik | 2.19 |
| 6881839 | Dtd1 | 2.19 |
| 6955292 | Isy1 | 2.19 |
| 6789584 | Tmem93 | 2.19 |
| 6889367 | Eif3m | 2.19 |
| 6885447 | Ubac1 | 2.19 |
| 6921000 | Bag1 | 2.19 |
| 6831709 | 1110038F14Rik | 2.19 |
| 6933478 | Pitpnb | 2.19 |
| 6890253 | Ndufaf1 | 2.19 |
| 6993735 | Fdx1l | 2.19 |
| 6889510 | 2700007P21Rik | 2.19 |
| 6968803 | Wdr73 | 2.19 |
| 6933451 | Ddx51 | 2.19 |
| 6890802 | Zc3h8 | 2.19 |
| 6955770 | Uba3 | 2.18 |
| 6930610 | 4933428G09Rik | 2.18 |
| 6775415 | Sgta | 2.18 |
| 6843208 | Slc5a3 | 2.18 |
| 6832573 | Ncaph2 | 2.18 |
| 6749911 | Nrp2 | 2.18 |
| 6984909 | Cmtm4 | 2.18 |
| 6880587 | Cep27 | 2.18 |
| 6922021 | AI314180 | 2.18 |
| 6764657 | Nvl | 2.18 |
| 6800917 | Ppp2r3c | 2.18 |
| 6785551 | Tbcd | 2.18 |
| 6887901 | Lnp | 2.18 |
| 6875376 | Bmi1 | 2.18 |
| 6775983 | Slc25a3 | 2.18 |
| 6926916 | Tardbp | 2.18 |
| 6820574 | Sugt1 | 2.18 |
| 6817672 | Arf4 | 2.18 |
| 6748736 | Eif5b | 2.18 |
| 6916148 | 0610037L13Rik | 2.17 |
| 6777902 | Tspan31 | 2.17 |
| 6965777 | Cd3eap | 2.17 |
| 6804667 | Bzw1 | 2.17 |
| 6896611 | Ndufb5 | 2.17 |
| 6769138 | Bsg | 2.17 |
| 6952376 | Tnpo3 | 2.17 |
| 6807251 | Tmed9 | 2.17 |
| 6763537 | Prdx6 | 2.17 |
| 6813410 | Ddx41 | 2.17 |
| 6805206 | Zscan12 | 2.17 |
| 6935496 | Bud31 | 2.17 |
| 6886073 | Ndufa8 | 2.17 |
| 6999675 | Hnrnpa1 | 2.17 |
| 6979097 | Vac14 | 2.17 |
| 6816222 | Ndufs4 | 2.17 |
| 6872704 | Atad1 | 2.17 |
| 6765317 | AA408296 | 2.17 |
| 6875702 | C430004E15Rik | 2.17 |
| 6763144 | Dhx9 | 2.17 |
| 6780940 | 9530068E07Rik | 2.16 |
| 6939063 | Nfxl1 | 2.16 |
| 6994668 | Ei24 | 2.16 |
| 6877936 | Ssb | 2.16 |
| 6887081 | Ly75 | 2.16 |
| 6836779 | Eef1d | 2.16 |
| 6782465 | Ywhae | 2.16 |
| 6878978 | Nup160 | 2.16 |
| 6898897 | Pet112l | 2.16 |
| 6998428 | Uba5 | 2.16 |
| 6958832 | Napa | 2.16 |
| 6862251 | Hdhd2 | 2.16 |
| 6985340 | Ddx19a | 2.16 |
| 6781526 | 2310040C09Rik | 2.16 |
| 6767886 | Asf1a | 2.16 |
| 6996191 | Glce | 2.15 |
| 6757796 | Ccdc115 | 2.15 |
| 6808617 | LOC100043111 | 2.15 |
| 6849433 | Mrpl28 | 2.15 |
| 6970774 | Eef1g | 2.15 |
| 6831851 | Nol12 | 2.15 |
| 6838349 | A130012F09 | 2.15 |
| 6855202 | Ppp1r11 | 2.15 |
| 6859937 | Srp19 | 2.15 |
| 6979631 | BC039210 | 2.15 |
| 6971606 | 2310057M21Rik | 2.15 |
| 6786610 | B3gnt2 | 2.15 |
| 6963946 | Uqcrc2 | 2.15 |
| 6854346 | 1600002H07Rik | 2.15 |
| 6813387 | Lman2 | 2.15 |
| 6844316 | Prodh | 2.15 |
| 6941026 | Pgam5 | 2.15 |
| 6792421 | Fads6 | 2.15 |
| 7013967 | Bhlhb9 | 2.15 |
| 6779857 | Fbxw11 | 2.15 |
| 6784239 | Coasy | 2.15 |
| 6824902 | Pspc1 | 2.15 |
| 6823068 | Anxa7 | 2.15 |
| 6791428 | Acly | 2.15 |
| 6870068 | Tmem180 | 2.14 |
| 6762425 | Ipo9 | 2.14 |
| 6922901 | 1810054D07Rik | 2.14 |
| 7006286 | Vamp7 | 2.14 |
| 6792999 | 0610009D07Rik | 2.14 |
| 6831993 | Smcr7l | 2.14 |
| 6804940 | AW209491 | 2.14 |
| 6852887 | Msh2 | 2.14 |
| 6926126 | 1110049F12Rik | 2.14 |
| 6847525 | Atp5j | 2.14 |
| 6971320 | Kif22 | 2.14 |
| 6844255 | Ube2l3 | 2.14 |
| 6787020 | Chac2 | 2.14 |
| 6768479 | Egr2 | 2.14 |
| 6947034 | Suclg1 | 2.14 |
| 6936855 | Paxip1 | 2.14 |
| 6873252 | Cwf19l1 | 2.14 |
| 6988198 | Sec61g | 2.14 |
| 6989100 | Rdx | 2.14 |
| 6790374 | Appbp2 | 2.13 |
| 6978386 | Gins3 | 2.13 |
| 6933656 | Coq5 | 2.13 |
| 6763754 | Tiprl | 2.13 |
| 6748832 | Pdcl3 | 2.13 |
| 6824624 | Hnrnpc | 2.13 |
| 6847190 | Samsn1 | 2.13 |
| 6828705 | Rad1 | 2.13 |
| 6823105 | Usp54 | 2.13 |
| 6843913 | Socs1 | 2.13 |
| 6764048 | Tomm40l | 2.13 |
| 6836723 | Ly6i | 2.13 |
| 6773438 | Gtf3c6 | 2.13 |
| 6795068 | Nubpl | 2.13 |
| 6885708 | Trub2 | 2.13 |
| 6934126 | Vps29 | 2.13 |
| 6886183 | Nr6a1 | 2.13 |
| 6803871 | 2010107E04Rik | 2.13 |
| 7009947 | Mid1ip1 | 2.13 |
| 6755224 | Pex19 | 2.12 |
| 6886117 | Strbp | 2.12 |
| 6876543 | Nek6 | 2.12 |
| 6777998 | Spryd4 | 2.12 |
| 6835579 | Trps1 | 2.12 |
| 6964573 | Bub3 | 2.12 |
| 6880468 | D2Ertd750e | 2.12 |
| 6967058 | Saal1 | 2.12 |
| 6960255 | 2410002F23Rik | 2.12 |
| 7013843 | Cstf2 | 2.12 |
| 6971316 | Mvp | 2.12 |
| 6769559 | Nt5dc3 | 2.12 |
| 6886768 | Prpf40a | 2.12 |
| 6838399 | Wnt10b | 2.12 |
| 6754205 | Stx6 | 2.12 |
| 6926225 | Alpl | 2.12 |
| 6806441 | Pak1ip1 | 2.12 |
| 6836999 | Rabl4 | 2.12 |
| 6849288 | Dci | 2.12 |
| 7012085 | Vbp1 | 2.12 |
| 6918719 | Exosc10 | 2.12 |
| 6917129 | Stk40 | 2.12 |
| 6979704 | Mc1r | 2.11 |
| 6977682 | Gipc1 | 2.11 |
| 6869885 | Entpd7 | 2.11 |
| 6815188 | Scamp1 | 2.11 |
| 6983878 | Rad23a | 2.11 |
| 6832107 | Rbx1 | 2.11 |
| 6951102 | Stk38l | 2.11 |
| 6840849 | Cd80 | 2.11 |
| 6987137 | BC017612 | 2.11 |
| 6985942 | Aprt | 2.11 |
| 6913286 | Sec61b | 2.11 |
| 7020206 | Ribc1 | 2.11 |
| 6773633 | Aim1 | 2.11 |
| 6916263 | Faf1 | 2.11 |
| 7013881 | Hnrph2 | 2.11 |
| 7014308 | Alg13 | 2.11 |
| 6780707 | Ifi47 | 2.11 |
| 6751300 | Cops7b | 2.11 |
| 6876062 | Ppp2r4 | 2.11 |
| 6949063 | Thumpd3 | 2.11 |
| 6872731 | 6530404N21Rik | 2.11 |
| 6927075 | Park7 | 2.11 |
| 6785675 | Nf2 | 2.11 |
| 6763695 | Blzf1 | 2.10 |
| 6995263 | Pafah1b2 | 2.10 |
| 6885424 | Mamdc4 | 2.10 |
| 6753085 | Nucks1 | 2.10 |
| 6949073 | Mtmr14 | 2.10 |
| 6926881 | Fbxo6 | 2.10 |
| 6925592 | Kpna6 | 2.10 |
| 6830161 | Eny2 | 2.10 |
| 6789863 | Ankrd13b | 2.10 |
| 6840598 | Fyttd1 | 2.10 |
| 6969800 | Ppme1 | 2.10 |
| 6855680 | Tjap1 | 2.10 |
| 6770646 | E2f7 | 2.10 |
| 6874954 | Sfmbt2 | 2.10 |
| 6759729 | Aamp | 2.10 |
| 6933226 | Cdc7 | 2.10 |
| 6854463 | Nme4 | 2.10 |
| 6877428 | Pkp4 | 2.09 |
| 6850175 | Cchcr1 | 2.09 |
| 6899585 | Mrpl9 | 2.09 |
| 6869975 | Peo1 | 2.09 |
| 6888718 | Ndufs3 | 2.09 |
| 6885482 | D2Bwg1335e | 2.09 |
| 6808260 | Ccdc127 | 2.09 |
| 6832332 | Nup50 | 2.09 |
| 6896519 | Skil | 2.09 |
| 6812984 | Dek | 2.09 |
| 6970148 | Taf10 | 2.09 |
| 6945935 | Cul1 | 2.09 |
| 6860666 | Commd10 | 2.09 |
| 6754476 | Mrps14 | 2.09 |
| 6913777 | Akap2 | 2.09 |
| 6792808 | Stra13 | 2.09 |
| 6882305 | H13 | 2.09 |
| 6750560 | Ttll4 | 2.09 |
| 6984908 | ENSMUSG00000051554 | 2.09 |
| 6977782 | Tnpo2 | 2.09 |
| 6943775 | Ccdc132 | 2.09 |
| 6933697 | Cit | 2.09 |
| 6783676 | Mrpl27 | 2.08 |
| 6986034 | Taf5l | 2.08 |
| 6880640 | Pdia3 | 2.08 |
| 6942487 | Lrwd1 | 2.08 |
| 6919932 | Osgin2 | 2.08 |
| 7017627 | Ubl4 | 2.08 |
| 6970393 | Rab6ip1 | 2.08 |
| 6887540 | Fastkd1 | 2.08 |
| 6885502 | Med22 | 2.08 |
| 6862316 | Atp5a1 | 2.08 |
| 6864604 | Gm1614 | 2.08 |
| 6946785 | Gng12 | 2.08 |
| 6865974 | Rps2 | 2.08 |
| 6839826 | Htf9c | 2.08 |
| 6844000 | C530044N13Rik | 2.08 |
| 6792527 | Ube2o | 2.07 |
| 6888311 | Zdhhc5 | 2.07 |
| 6788133 | 0610009B22Rik | 2.07 |
| 6932469 | Uso1 | 2.07 |
| 6870680 | Trub1 | 2.07 |
| 6933987 | Ddx54 | 2.07 |
| 6882050 | Nxt1 | 2.07 |
| 6786206 | Sec61g | 2.07 |
| 6913531 | Nipsnap3a | 2.07 |
| 6924810 | Toe1 | 2.07 |
| 6948274 | Kbtbd8 | 2.07 |
| 6910622 | C030011O14Rik | 2.07 |
| 6966182 | Eif3k | 2.07 |
| 7011996 | Eif1 | 2.07 |
| 6864404 | Gypc | 2.07 |
| 6815708 | Ppwd1 | 2.07 |
| 6955131 | Spr | 2.07 |
| 6775213 | 1810008A18Rik | 2.07 |
| 6889304 | Caprin1 | 2.07 |
| 6862922 | Cd226 | 2.07 |
| 6921479 | Txndc4 | 2.07 |
| 6871493 | 2810441K11Rik | 2.07 |
| 6966148 | Med29 | 2.06 |
| 6958995 | Opa3 | 2.06 |
| 6935577 | Rpo1 | 2.06 |
| 6997142 | 2410127L17Rik | 2.06 |
| 6906808 | Dap3 | 2.06 |
| 7012080 | Fundc2 | 2.06 |
| 6782801 | BC037438 | 2.06 |
| 6791212 | Pcgf2 | 2.06 |
| 6878430 | Ube2e3 | 2.06 |
| 6893630 | Ctsz | 2.06 |
| 6808544 | Cetn3 | 2.06 |
| 6871051 | Rbm14 | 2.06 |
| 6991791 | Dbr1 | 2.06 |
| 6757549 | Lsm5 | 2.06 |
| 6911289 | Dap3 | 2.06 |
| 6854314 | Kctd5 | 2.05 |
| 6775372 | Uqcr | 2.05 |
| 6878049 | B230120H23Rik | 2.05 |
| 6950688 | Aebp2 | 2.05 |
| 6816248 | Itga1 | 2.05 |
| 6933141 | Lrrc8d | 2.05 |
| 6999415 | Oxsr1 | 2.05 |
| 6903480 | Armc1 | 2.05 |
| 6919050 | A430005L14Rik | 2.05 |
| 6896997 | Hspa4l | 2.05 |
| 6749834 | Eif4a1 | 2.05 |
| 6925782 | Ythdf2 | 2.05 |
| 6868831 | Ndufb4 | 2.05 |
| 6815479 | Ptcd2 | 2.05 |
| 6883350 | Pard6b | 2.05 |
| 6951121 | Mrps35 | 2.05 |
| 6922237 | Hdhd3 | 2.05 |
| 6836341 | Sla | 2.05 |
| 6901953 | Gbp3 | 2.05 |
| 6898154 | Rsrc1 | 2.05 |
| 6819885 | Ccdc25 | 2.05 |
| 6831928 | Tomm22 | 2.04 |
| 6806948 | Ippk | 2.04 |
| 6975876 | Ufsp2 | 2.04 |
| 6759248 | Ndufs1 | 2.04 |
| 6791656 | Dcakd | 2.04 |
| 6867975 | Ints5 | 2.04 |
| 6835981 | Tatdn1 | 2.04 |
| 6954767 | Rnf26 | 2.04 |
| 6895838 | Car13 | 2.04 |
| 6805194 | Trim27 | 2.04 |
| 6988774 | Bud13 | 2.04 |
| 6960147 | Etfb | 2.04 |
| 6757331 | Eif3m | 2.04 |
| 6973247 | Nup98 | 2.04 |
| 6786572 | Mdh1 | 2.04 |
| 6833640 | Ptger4 | 2.04 |
| 6862126 | BC031181 | 2.04 |
| 6857440 | Cebpz | 2.04 |
| 6789851 | Efcab5 | 2.04 |
| 6753706 | Glrx2 | 2.04 |
| 6819842 | Ints9 | 2.03 |
| 6849044 | Ppp2r1a | 2.03 |
| 6947737 | Copg | 2.03 |
| 6896518 | Prkci | 2.03 |
| 6790366 | Pigw | 2.03 |
| 6916708 | Med8 | 2.03 |
| 7015425 | Hdac6 | 2.03 |
| 6852676 | Eml4 | 2.03 |
| 6922219 | Cdc26 | 2.03 |
| 6878643 | Zc3h15 | 2.03 |
| 6993872 | BC050092 | 2.03 |
| 6822488 | Ptma | 2.03 |
| 6856270 | Khsrp | 2.03 |
| 6981683 | Thex1 | 2.03 |
| 6987348 | Mrpl4 | 2.03 |
| 6917782 | Fusip1 | 2.03 |
| 6866307 | Ptpn2 | 2.03 |
| 6881146 | Stk35 | 2.03 |
| 6861136 | Rnuxa | 2.03 |
| 6907216 | Prpf3 | 2.03 |
| 6902148 | Odf2l | 2.03 |
| 7013909 | Armcx3 | 2.03 |
| 6781607 | 1810036I24Rik | 2.02 |
| 6940330 | Hnrnpd | 2.02 |
| 6785437 | Ccdc137 | 2.02 |
| 6959228 | Exosc5 | 2.02 |
| 6892806 | D930001I22Rik | 2.02 |
| 6854467 | Arhgdig | 2.02 |
| 6847338 | D16Ertd472e | 2.02 |
| 6931857 | Polr2b | 2.02 |
| 6778976 | A630050E13Rik | 2.02 |
| 6990683 | Ick | 2.02 |
| 6939771 | Grsf1 | 2.02 |
| 6823849 | Mapk8 | 2.02 |
| 6999447 | Gorasp1 | 2.01 |
| 6789324 | Mpdu1 | 2.01 |
| 6802387 | Tmed10 | 2.01 |
| 6845914 | Atp6v1a | 2.01 |
| 6836838 | Cpsf1 | 2.01 |
| 6961889 | Mrps11 | 2.01 |
| 6919154 | Nadk | 2.01 |
| 6770214 | Cep290 | 2.01 |
| 6827790 | Tgds | 2.01 |
| 6886110 | Pdcl | 2.01 |
| 6912477 | Bach2 | 2.01 |
| 6769461 | AI597468 | 2.01 |
| 6838917 | Chrac1 | 2.01 |
| 6854514 | Bak1 | 2.01 |
| 6791540 | Tmem101 | 2.01 |
| 6871297 | Nat11 | 2.01 |
| 6869212 | Minpp1 | 2.01 |
| 6959780 | Pepd | 2.01 |
| 6996792 | Ccpg1 | 2.01 |
| 6850637 | Aars2 | 2.01 |
| 6954529 | Rpia | 2.01 |
| 6994588 | Foxred1 | 2.01 |
| 6750868 | Acsl3 | 2.01 |
| 7019829 | Psmd10 | 2.01 |
| 6751294 | Ptma | 2.01 |
| 6917835 | Luzp1 | 2.00 |
| 6971345 | Sephs2 | 2.00 |
| 6994579 | St3gal4 | 2.00 |
| 6825137 | Nupl1 | 2.00 |
| 6860135 | Zmat2 | 2.00 |
| 6862459 | Txnl4a | 2.00 |
| 6783264 | Ptrh2 | 2.00 |
| 6929714 | Bre | 2.00 |
| 6920988 | Smu1 | 2.00 |
| 6978314 | Herpud1 | 2.00 |
| 6975861 | Sorbs2 | 0.50 |
| 6896835 | 4932438A13Rik | 0.50 |
| 7015432 | Gata1 | 0.50 |
| 6830055 | Oxr1 | 0.50 |
| 6772382 | Ccdc28a | 0.50 |
| 6989556 | Parp6 | 0.50 |
| 6838413 | Lmbr1l | 0.50 |
| 6957436 | Klra19 | 0.50 |
| 6959167 | Arhgef1 | 0.50 |
| 7012860 | Foxo4 | 0.50 |
| 6837252 | Mkl1 | 0.50 |
| 6758754 | Rftn2 | 0.50 |
| 6832115 | Ep300 | 0.50 |
| 6979144 | Znrf1 | 0.50 |
| 6962779 | Pak1 | 0.50 |
| 6995762 | Acsbg1 | 0.50 |
| 6792679 | Cbx8 | 0.50 |
| 6960210 | Klk1b8 | 0.50 |
| 6799462 | Id2 | 0.50 |
| 6965143 | Athl1 | 0.50 |
| 6968778 | Man2a2 | 0.50 |
| 6982094 | Cyp4v3 | 0.50 |
| 7018576 | Rgag4 | 0.50 |
| 6990027 | Herc1 | 0.49 |
| 6907204 | Ecm1 | 0.49 |
| 6978937 | Sntb2 | 0.49 |
| 6791472 | Ezh1 | 0.49 |
| 6908350 | Amy1 | 0.49 |
| 6949865 | Tnfrsf1a | 0.49 |
| 6843198 | Itsn1 | 0.49 |
| 6987954 | Ets1 | 0.49 |
| 6835984 | Mtss1 | 0.49 |
| 6967061 | Saa1 | 0.49 |
| 6981168 | Letm2 | 0.49 |
| 6832010 | AW544981 | 0.49 |
| 6917551 | Ahdc1 | 0.49 |
| 6939830 | Gc | 0.49 |
| 6920754 | Mobkl2b | 0.49 |
| 6960211 | Klk1b11 | 0.49 |
| 6782563 | Ssh2 | 0.49 |
| 6960235 | Klk1b21 | 0.49 |
| 6815599 | Pik3r1 | 0.49 |
| 6879054 | Lrp4 | 0.49 |
| 6903185 | Fabp4 | 0.49 |
| 6948418 | Mitf | 0.49 |
| 6906673 | Pear1 | 0.49 |
| 6888307 | AI225934 | 0.49 |
| 7009775 | Prickle3 | 0.49 |
| 6890935 | 4930402H24Rik | 0.49 |
| 6754681 | Sell | 0.49 |
| 6951401 | Pon3 | 0.49 |
| 6758139 | Mfsd9 | 0.49 |
| 6827123 | Kctd12 | 0.49 |
| 6998752 | Fbxw13 | 0.49 |
| 6888615 | Olfr1230 | 0.49 |
| 6831848 | Sh3bp1 | 0.49 |
| 6784526 | Lin52 | 0.49 |
| 6955010 | Wbp1 | 0.49 |
| 6872528 | Insl6 | 0.49 |
| 6818742 | Peli2 | 0.49 |
| 6915873 | Tctex1d1 | 0.49 |
| 6910275 | Spata1 | 0.49 |
| 6905424 | P2ry12 | 0.49 |
| 6853951 | Prdm9 | 0.49 |
| 6878720 | Prg3 | 0.49 |
| 6897441 | Foxo1 | 0.49 |
| 6898076 | Tiparp | 0.49 |
| 6975307 | Tmem66 | 0.49 |
| 6844819 | Cldn1 | 0.49 |
| 6959453 | 5830482F20Rik | 0.49 |
| 6845416 | Stfa3 | 0.49 |
| 6871627 | Dtx4 | 0.49 |
| 6956550 | Camk1 | 0.49 |
| 6943004 | Zkscan14 | 0.48 |
| 6796027 | Slc38a6 | 0.48 |
| 6983793 | Pkn1 | 0.48 |
| 6852874 | Ttc7 | 0.48 |
| 6923059 | Adfp | 0.48 |
| 6773174 | Dse | 0.48 |
| 6960246 | Klk1b3 | 0.48 |
| 6965556 | V1rg9 | 0.48 |
| 6949841 | Ing4 | 0.48 |
| 6973572 | Isoc2b | 0.48 |
| 6980090 | Clec4g | 0.48 |
| 6747497 | Sgk3 | 0.48 |
| 6970108 | Olfr684 | 0.48 |
| 6931217 | Klf3 | 0.48 |
| 6849056 | V1rf1 | 0.48 |
| 6768326 | Rufy2 | 0.48 |
| 6945684 | Olfr460 | 0.48 |
| 6839957 | 2900046G09Rik | 0.48 |
| 6819602 | Arl11 | 0.48 |
| 6802197 | Map3k9 | 0.48 |
| 6884235 | 9230112E08Rik | 0.48 |
| 6908348 | Amy2 | 0.48 |
| 6959557 | Zfp260 | 0.48 |
| 6894750 | Olfr338 | 0.48 |
| 6777583 | Rassf3 | 0.48 |
| 6987586 | Clpb | 0.48 |
| 6941173 | Cmklr1 | 0.48 |
| 6978923 | Cdh1 | 0.48 |
| 6810066 | Depdc1b | 0.48 |
| 6855225 | Mog | 0.48 |
| 6782102 | Mgl2 | 0.48 |
| 6993055 | Snrk | 0.48 |
| 6816080 | Map3k1 | 0.48 |
| 6961977 | Sema4b | 0.48 |
| 6763706 | Atp1b1 | 0.48 |
| 6949092 | Il17re | 0.48 |
| 6786348 | Meis1 | 0.48 |
| 6949740 | Clec4b2 | 0.48 |
| 6781606 | Fbxw10 | 0.48 |
| 6828125 | D14Ertd668e | 0.48 |
| 6965078 | Prap1 | 0.48 |
| 6960157 | BC043301 | 0.48 |
| 7010434 | Wdr44 | 0.48 |
| 6778984 | Cnrip1 | 0.48 |
| 7010093 | Gpr82 | 0.48 |
| 6844400 | Olfr166 | 0.48 |
| 6894907 | Camk1d | 0.48 |
| 6871156 | Frmd8 | 0.48 |
| 6888446 | Olfr1057 | 0.48 |
| 6922541 | Megf9 | 0.48 |
| 6882293 | Defb20 | 0.48 |
| 6810063 | Elovl7 | 0.47 |
| 6896406 | Pld1 | 0.47 |
| 6780855 | Clk4 | 0.47 |
| 7010330 | Rgn | 0.47 |
| 6998094 | Acpl2 | 0.47 |
| 6977797 | Man2b1 | 0.47 |
| 6840175 | Lpp | 0.47 |
| 6966358 | Gramd1a | 0.47 |
| 6802384 | Acyp1 | 0.47 |
| 6792650 | RP23 | 0.47 |
| 6831606 | Gsdmd | 0.47 |
| 6888891 | Tspan18 | 0.47 |
| 6837784 | Mapk12 | 0.47 |
| 6789908 | Pipox | 0.47 |
| 6951253 | 4833442J19Rik | 0.47 |
| 6846497 | St3gal6 | 0.47 |
| 6952415 | Ube2h | 0.47 |
| 6749444 | 5330401P04Rik | 0.47 |
| 6942737 | Ttyh3 | 0.47 |
| 6797478 | Rin3 | 0.47 |
| 6865221 | Trim36 | 0.47 |
| 6819887 | Clu | 0.47 |
| 6789274 | Chd3 | 0.47 |
| 6929920 | Afap1 | 0.47 |
| 7014024 | Tceal3 | 0.46 |
| 6832132 | Tef | 0.46 |
| 6779264 | Peli1 | 0.46 |
| 6996190 | Paqr5 | 0.46 |
| 6971759 | A130023I24Rik | 0.46 |
| 6748888 | Il1rl1 | 0.46 |
| 6926078 | A330049M08Rik | 0.46 |
| 6803918 | Nudt14 | 0.46 |
| 6825952 | ENSMUSG00000043225 | 0.46 |
| 6850058 | Ager | 0.46 |
| 6899312 | 1700094D03Rik | 0.46 |
| 6929550 | Rnf32 | 0.46 |
| 6845154 | Lrrc33 | 0.46 |
| 6785139 | Armc7 | 0.46 |
| 6950504 | BC049715 | 0.46 |
| 6860204 | Pcdhb19 | 0.46 |
| 6889273 | Ehf | 0.46 |
| 6953920 | Ppm1k | 0.46 |
| 6867853 | Map4k2 | 0.46 |
| 6788728 | 4930412M03Rik | 0.46 |
| 6771884 | Esr1 | 0.46 |
| 6757981 | Tsga10 | 0.46 |
| 6881028 | Acoxl | 0.46 |
| 6905912 | 1110032A04Rik | 0.46 |
| 6953587 | Skap2 | 0.46 |
| 6772193 | Utrn | 0.46 |
| 6945664 | EG434008 | 0.46 |
| 6849303 | Pkd1 | 0.46 |
| 6987374 | Slc44a2 | 0.46 |
| 6889712 | Olfr1277 | 0.46 |
| 6871566 | Ms4a6d | 0.46 |
| 6871607 | Olfr76 | 0.46 |
| 6813094 | Susd3 | 0.46 |
| 6942532 | Trim56 | 0.46 |
| 6964039 | Prkcb1 | 0.46 |
| 6996389 | Fbxl22 | 0.46 |
| 6971981 | Mapk1ip1 | 0.46 |
| 7017553 | Xlr5a | 0.46 |
| 6838580 | Galnt6 | 0.45 |
| 6966249 | Zfp14 | 0.45 |
| 6849551 | E230001N04Rik | 0.45 |
| 6878718 | Prg2 | 0.45 |
| 6866289 | Mppe1 | 0.45 |
| 6890068 | Rasgrp1 | 0.45 |
| 6926938 | Kif1b | 0.45 |
| 6962502 | Dlg2 | 0.45 |
| 6748884 | Il1r1 | 0.45 |
| 6837143 | Cbx7 | 0.45 |
| 6785133 | Kctd2 | 0.45 |
| 7015994 | Zfp182 | 0.45 |
| 6775576 | Appl2 | 0.45 |
| 6935197 | Gpr146 | 0.45 |
| 6810961 | Chrm3 | 0.45 |
| 6967059 | Saa3 | 0.45 |
| 6983255 | Abhd8 | 0.45 |
| 6916611 | Hectd3 | 0.45 |
| 6970340 | Olfr519 | 0.45 |
| 6995504 | Bco2 | 0.45 |
| 6934885 | Wbscr27 | 0.45 |
| 6950485 | Atf7ip | 0.45 |
| 6988711 | Amica1 | 0.45 |
| 6918129 | Atp13a2 | 0.45 |
| 6938891 | Atp8a1 | 0.45 |
| 6760847 | St8sia4 | 0.45 |
| 6988308 | Olfr924 | 0.45 |
| 6843500 | Pcp4 | 0.45 |
| 6944019 | Glcci1 | 0.45 |
| 6953993 | Nap1l5 | 0.45 |
| 6833005 | BC038822 | 0.45 |
| 6816161 | Gzmk | 0.45 |
| 6785306 | Afmid | 0.45 |
| 6788654 | A230051G13Rik | 0.45 |
| 6836949 | Apol9a | 0.45 |
| 6764832 | Hlx | 0.45 |
| 6968796 | Crtc3 | 0.45 |
| 6788741 | Tom1l2 | 0.45 |
| 6945321 | Akr1b7 | 0.45 |
| 6978355 | Gpr97 | 0.45 |
| 6758861 | Hsfy2 | 0.45 |
| 6758027 | Tbc1d8 | 0.45 |
| 6996356 | Plekho2 | 0.45 |
| 6900450 | Slc25a24 | 0.44 |
| 6782273 | Atp2a3 | 0.44 |
| 6957762 | Erp27 | 0.44 |
| 6953956 | V1rc22 | 0.44 |
| 6922026 | Ltb4dh | 0.44 |
| 6866040 | Spink7 | 0.44 |
| 6808612 | ENSMUSG00000074792 | 0.44 |
| 6854341 | Dnase1l2 | 0.44 |
| 6954385 | Gadd45a | 0.44 |
| 6921158 | E130306D19Rik | 0.44 |
| 6908347 | Amy2 | 0.44 |
| 6954418 | Il23r | 0.44 |
| 6960240 | Egfbp2 | 0.44 |
| 6990774 | Myo6 | 0.44 |
| 6792702 | Sgsh | 0.44 |
| 6873153 | Frat2 | 0.44 |
| 6997626 | Snx14 | 0.44 |
| 6803863 | Ppp1r13b | 0.44 |
| 6989553 | Hexa | 0.44 |
| 6900191 | Ppm1j | 0.44 |
| 6798388 | Zfp386 | 0.44 |
| 6784412 | Fmnl1 | 0.44 |
| 6805324 | V1rh13 | 0.44 |
| 6868110 | Ms4a4d | 0.44 |
| 6957789 | Eps8 | 0.44 |
| 6831687 | Kifc2 | 0.44 |
| 6766287 | Nhsl1 | 0.44 |
| 7015995 | Zfp300 | 0.44 |
| 6965932 | Dedd2 | 0.44 |
| 6964036 | Acp1 | 0.44 |
| 6966939 | Flt3l | 0.44 |
| 6850763 | Trerf1 | 0.44 |
| 6983819 | Il27ra | 0.44 |
| 6779360 | 4930430E16Rik | 0.44 |
| 6809551 | Ccdc125 | 0.44 |
| 6785538 | Narf | 0.44 |
| 6924951 | 4930538K18Rik | 0.44 |
| 6788069 | Zfp354a | 0.44 |
| 6838717 | Rarg | 0.44 |
| 6869796 | Marveld1 | 0.43 |
| 6926021 | Sepn1 | 0.43 |
| 6972970 | 4632433K11Rik | 0.43 |
| 6902179 | Mcoln3 | 0.43 |
| 6972418 | Osbpl5 | 0.43 |
| 7016823 | Gpc4 | 0.43 |
| 6782034 | Sat2 | 0.43 |
| 6859304 | Rnf125 | 0.43 |
| 6838492 | Lima1 | 0.43 |
| 6862133 | Smad7 | 0.43 |
| 6768155 | Psap | 0.43 |
| 6927426 | Dhrs3 | 0.43 |
| 6915650 | Inadl | 0.43 |
| 6751536 | Ramp1 | 0.43 |
| 6939931 | Btc | 0.43 |
| 6968314 | Mctp2 | 0.43 |
| 6946920 | Cd8a | 0.43 |
| 7009941 | Tspan7 | 0.43 |
| 7012107 | EG238829 | 0.43 |
| 6860259 | Arhgap26 | 0.43 |
| 6852845 | Rhoq | 0.43 |
| 6789407 | Alox12 | 0.43 |
| 6751620 | Olfr1410 | 0.43 |
| 6924017 | Wdr78 | 0.43 |
| 6957059 | C3ar1 | 0.43 |
| 6784363 | Adam11 | 0.43 |
| 6846543 | Olfr197 | 0.43 |
| 6782811 | Rnf135 | 0.43 |
| 6953429 | Tmem176b | 0.43 |
| 6910668 | St6galnac3 | 0.43 |
| 6792606 | Tha1 | 0.43 |
| 6885431 | Tmem141 | 0.43 |
| 6849515 | Pacsin1 | 0.43 |
| 6783182 | Bcas3 | 0.43 |
| 6980548 | E330037G11Rik | 0.43 |
| 7010345 | Usp11 | 0.43 |
| 6793416 | D12Ertd553e | 0.43 |
| 6858938 | Hrh4 | 0.43 |
| 7020676 | Car5b | 0.43 |
| 6971323 | Qprt | 0.42 |
| 6964245 | Gdpd3 | 0.42 |
| 6769180 | Abca7 | 0.42 |
| 6875421 | Msrb2 | 0.42 |
| 6796193 | Plekhg3 | 0.42 |
| 6962379 | Rab38 | 0.42 |
| 6907869 | Mov10 | 0.42 |
| 6871874 | Gcnt1 | 0.42 |
| 6993878 | Zfp810 | 0.42 |
| 6809436 | Zfp366 | 0.42 |
| 6854872 | A630049H14Rik | 0.42 |
| 6792544 | St6galnac2 | 0.42 |
| 6978781 | BC015286 | 0.42 |
| 6936719 | Kcnh2 | 0.42 |
| 6926029 | Ldlrap1 | 0.42 |
| 6844301 | Car15 | 0.42 |
| 6811524 | Olfr1361 | 0.42 |
| 6897691 | Tm4sf4 | 0.42 |
| 6755310 | 1810030J14Rik | 0.42 |
| 6987924 | Grit | 0.42 |
| 6985984 | Spata2L | 0.42 |
| 6957444 | Klra3 | 0.42 |
| 6942441 | Hip1 | 0.42 |
| 6882256 | Scrt2 | 0.42 |
| 6842008 | Stx19 | 0.42 |
| 6846386 | Cep97 | 0.42 |
| 6839773 | B830017H08Rik | 0.42 |
| 7015398 | Ppp1r3f | 0.42 |
| 6918349 | Dhrs3 | 0.42 |
| 6855478 | Slc25a27 | 0.42 |
| 6871163 | Cdc42ep2 | 0.42 |
| 6913194 | Tdrd7 | 0.42 |
| 6949850 | A930037G23Rik | 0.42 |
| 6780527 | Adam19 | 0.42 |
| 6770201 | Kitl | 0.42 |
| 6769535 | Timp3 | 0.41 |
| 6829495 | Ctnnd2 | 0.41 |
| 6873476 | ENSMUSG00000071525 | 0.41 |
| 6845933 | Sidt1 | 0.41 |
| 7019547 | Zmat1 | 0.41 |
| 6969949 | Olfr559 | 0.41 |
| 6966934 | Rcn3 | 0.41 |
| 6885891 | 9430097D07Rik | 0.41 |
| 6972918 | V1rj3 | 0.41 |
| 6789817 | Abr | 0.41 |
| 6919596 | Asph | 0.41 |
| 6988325 | Olfr242 | 0.41 |
| 6962961 | B930006L02Rik | 0.41 |
| 6783689 | Ppp1r9b | 0.41 |
| 6990438 | BC003885 | 0.41 |
| 6768867 | Ggt1 | 0.41 |
| 6967862 | Klf13 | 0.41 |
| 6753068 | 5430435G22Rik | 0.41 |
| 6782919 | Ccl8 | 0.41 |
| 6876490 | Gpr21 | 0.41 |
| 6781960 | Slc25a35 | 0.41 |
| 6824825 | 1500005A01Rik | 0.41 |
| 6792595 | Tmc6 | 0.41 |
| 6855074 | Ly6g5b | 0.41 |
| 6960205 | Klk1b11 | 0.41 |
| 6969837 | P2ry6 | 0.41 |
| 6993845 | Rab3d | 0.41 |
| 6841701 | Senp7 | 0.41 |
| 6854962 | Rab11b | 0.41 |
| 6966145 | Zfp36 | 0.41 |
| 6977032 | Rab3a | 0.41 |
| 6900237 | Ovgp1 | 0.41 |
| 6752165 | Serpinb8 | 0.41 |
| 6970045 | Trim12 | 0.41 |
| 6970890 | Gp2 | 0.41 |
| 6873368 | Kcnip2 | 0.40 |
| 6771334 | Ppm1h | 0.40 |
| 6789605 | Olfr378 | 0.40 |
| 6890127 | Bmf | 0.40 |
| 6854658 | Stk38 | 0.40 |
| 6803120 | Ccdc88c | 0.40 |
| 6765276 | Gstp1 | 0.40 |
| 6945339 | Tmem140 | 0.40 |
| 6785369 | Slc26a11 | 0.40 |
| 6980107 | Cd209c | 0.40 |
| 6812386 | F13a1 | 0.40 |
| 6804138 | Sp4 | 0.40 |
| 6874085 | 4930506M07Rik | 0.40 |
| 6915231 | Ifna4 | 0.40 |
| 6763937 | Pbx1 | 0.40 |
| 6790536 | Gdpd1 | 0.40 |
| 6871690 | Olfr1504 | 0.40 |
| 6867987 | Ahnak | 0.40 |
| 6856276 | Slc25a23 | 0.40 |
| 6988603 | Thy1 | 0.40 |
| 6805381 | Hist1h1c | 0.40 |
| 6850062 | Prrt1 | 0.40 |
| 6749352 | Sdpr | 0.40 |
| 6748525 | D630036G22Rik | 0.40 |
| 6766772 | Arhgap18 | 0.40 |
| 6806791 | Cap2 | 0.40 |
| 6999530 | Sec22c | 0.40 |
| 6934972 | Rasa4 | 0.40 |
| 7017585 | Pnck | 0.40 |
| 6947939 | Slc41a3 | 0.39 |
| 6908330 | Amy2 | 0.39 |
| 6780837 | Olfr1378 | 0.39 |
| 6933139 | C230066G23Rik | 0.39 |
| 6843653 | Coro7 | 0.39 |
| 6899100 | Mef2d | 0.39 |
| 6953931 | V1rc28 | 0.39 |
| 6880508 | Spint1 | 0.39 |
| 6771733 | Olfr780 | 0.39 |
| 6764578 | Cabc1 | 0.39 |
| 6978843 | 2310066E14Rik | 0.39 |
| 6916836 | Rims3 | 0.39 |
| 6791696 | Arhgap27 | 0.39 |
| 6891879 | Thbd | 0.39 |
| 6850047 | BC051142 | 0.39 |
| 6784787 | Arsg | 0.39 |
| 7015061 | Gpm6b | 0.39 |
| 6926504 | Tmem51 | 0.39 |
| 6810333 | Esm1 | 0.39 |
| 6788393 | Ccdc69 | 0.39 |
| 6880683 | Sord | 0.39 |
| 6755266 | Olfr1404 | 0.39 |
| 6874549 | Phxr1 | 0.39 |
| 6917626 | Lin28a | 0.39 |
| 6871121 | Snx32 | 0.39 |
| 6851103 | Pcaf | 0.39 |
| 6978883 | Lypla3 | 0.39 |
| 6945730 | Ephb6 | 0.39 |
| 6959487 | Ppp1r14a | 0.39 |
| 6901196 | Tifa | 0.39 |
| 6928742 | Abcb4 | 0.39 |
| 6888114 | Neurod1 | 0.39 |
| 6825888 | Itm2b | 0.39 |
| 6872783 | Lipa | 0.39 |
| 6789541 | Spns2 | 0.39 |
| 6818696 | Lgals3 | 0.39 |
| 6809030 | Arsb | 0.39 |
| 6835759 | Enpp2 | 0.39 |
| 6928939 | Hgf | 0.39 |
| 6828472 | Dab2 | 0.39 |
| 6998214 | Mras | 0.39 |
| 6905818 | Ift80 | 0.39 |
| 6977712 | Podnl1 | 0.38 |
| 6857461 | Cdc42ep3 | 0.38 |
| 6996659 | B230380D07Rik | 0.38 |
| 6779355 | Zrsr1 | 0.38 |
| 6874584 | Frmd4a | 0.38 |
| 6888495 | Olfr1110 | 0.38 |
| 6946038 | Tmem176a | 0.38 |
| 6905897 | B3galnt1 | 0.38 |
| 6971280 | Sult1a1 | 0.38 |
| 6801636 | Rtn1 | 0.38 |
| 6815749 | Rnf180 | 0.38 |
| 6765275 | Traf5 | 0.38 |
| 6791528 | Dusp3 | 0.38 |
| 6748886 | Il1rl2 | 0.38 |
| 6765474 | Cnksr3 | 0.38 |
| 6807156 | Hrh2 | 0.38 |
| 6964199 | Sbk1 | 0.38 |
| 6849991 | Rgl2 | 0.38 |
| 6881249 | Cdc25b | 0.38 |
| 6767088 | Tspyl4 | 0.38 |
| 6852144 | Lbh | 0.38 |
| 6969972 | Olfr601 | 0.38 |
| 6888631 | Olfr1243 | 0.38 |
| 6986987 | Phxr4 | 0.38 |
| 6808977 | EG218444 | 0.38 |
| 6864763 | Centd3 | 0.38 |
| 6925165 | Macf1 | 0.38 |
| 6960249 | Klk1b5 | 0.38 |
| 6831527 | Psca | 0.38 |
| 6764209 | Darc | 0.38 |
| 6937466 | Sorcs2 | 0.38 |
| 6813742 | Ctla2a | 0.38 |
| 6972660 | Ncr1 | 0.38 |
| 6957420 | Klrc2 | 0.38 |
| 6875592 | Apbb1ip | 0.38 |
| 6886356 | Zeb2 | 0.38 |
| 6932336 | Afp | 0.38 |
| 6935082 | Lrch4 | 0.37 |
| 6878031 | Itga6 | 0.37 |
| 6929908 | Acox3 | 0.37 |
| 6876205 | C230093N12Rik | 0.37 |
| 6884302 | Pcmtd2 | 0.37 |
| 6754691 | F5 | 0.37 |
| 6869223 | B430203M17Rik | 0.37 |
| 6881101 | Zc3h6 | 0.37 |
| 6791015 | Pdk2 | 0.37 |
| 6867653 | Clcf1 | 0.37 |
| 6980100 | Cd209e | 0.37 |
| 6876464 | Olfr352 | 0.37 |
| 6790531 | Ypel2 | 0.37 |
| 6795779 | Klhdc1 | 0.37 |
| 6844530 | 2510009E07Rik | 0.37 |
| 6899251 | Pbxip1 | 0.37 |
| 6758646 | Inpp1 | 0.37 |
| 6926084 | Npal3 | 0.37 |
| 6829361 | Tiaf2 | 0.37 |
| 6929655 | Khk | 0.37 |
| 6850341 | Olfr126 | 0.37 |
| 6785079 | Ttyh2 | 0.37 |
| 6882538 | Acss2 | 0.37 |
| 6813887 | Ctsl | 0.37 |
| 6969782 | EG330602 | 0.37 |
| 6965558 | V1rg11 | 0.37 |
| 6946339 | Chn2 | 0.37 |
| 6831821 | Pscd4 | 0.37 |
| 6907262 | Fcgr1 | 0.37 |
| 6791418 | Hap1 | 0.37 |
| 6755179 | F11r | 0.37 |
| 6754537 | Tnfsf18 | 0.36 |
| 6903157 | Pag1 | 0.36 |
| 6841142 | Cd200r2 | 0.36 |
| 6755294 | Olfr433 | 0.36 |
| 6831800 | Csf2rb | 0.36 |
| 6994773 | Olfr938 | 0.36 |
| 6867806 | Slc25a45 | 0.36 |
| 6764211 | Cadm3 | 0.36 |
| 6789484 | Nlrp1a | 0.36 |
| 6876217 | Sh2d3c | 0.36 |
| 6791229 | Plxdc1 | 0.36 |
| 6782105 | Mgl1 | 0.36 |
| 6870991 | Ankrd13d | 0.36 |
| 6803102 | Rps6ka5 | 0.36 |
| 6824142 | Tspan14 | 0.36 |
| 6818104 | 8030431A06Rik | 0.36 |
| 6959303 | Sertad3 | 0.36 |
| 6765454 | C030002C11Rik | 0.36 |
| 6870980 | Tbc1d10c | 0.36 |
| 6762784 | Rgs2 | 0.36 |
| 6766350 | Ifngr1 | 0.36 |
| 6921012 | Aqp3 | 0.36 |
| 6867566 | Cpt1a | 0.36 |
| 6762944 | Pla2g4a | 0.36 |
| 6917997 | Camk2n1 | 0.36 |
| 6923147 | Ifnb1 | 0.36 |
| 6759718 | Tns1 | 0.36 |
| 6812212 | Serpinb6a | 0.36 |
| 6960578 | Nav2 | 0.36 |
| 6975658 | Slc7a2 | 0.35 |
| 6958984 | Dmpk | 0.35 |
| 6941650 | Oas1c | 0.35 |
| 6914007 | Orm1 | 0.35 |
| 6775146 | Susd2 | 0.35 |
| 6988643 | Bcl9l | 0.35 |
| 6849950 | Adamts10 | 0.35 |
| 6748217 | Lmbrd1 | 0.35 |
| 6896032 | Cp | 0.35 |
| 6838808 | Zfp385a | 0.35 |
| 6755896 | Dusp10 | 0.35 |
| 6755210 | Cd84 | 0.35 |
| 6931529 | Atp10d | 0.35 |
| 6987470 | Bbs9 | 0.35 |
| 6952284 | Grm8 | 0.35 |
| 6978156 | Rbl2 | 0.35 |
| 6949086 | Ttll3 | 0.35 |
| 6930335 | Cpeb2 | 0.35 |
| 6798335 | Adam6 | 0.35 |
| 6980968 | Ap3m2 | 0.35 |
| 6849481 | Itpr3 | 0.35 |
| 6784280 | Tmem106a | 0.35 |
| 6896353 | Tnfsf10 | 0.35 |
| 6777249 | Kcnmb4 | 0.35 |
| 6790244 | Slfn8 | 0.35 |
| 6958269 | Itpr2 | 0.35 |
| 6783882 | Sp6 | 0.35 |
| 6962745 | Gab2 | 0.35 |
| 6763090 | Rgl1 | 0.35 |
| 6869543 | Gpr120 | 0.35 |
| 6784205 | Cnp | 0.35 |
| 6999249 | Tgfbr2 | 0.35 |
| 6857797 | Zfp36l2 | 0.35 |
| 6950539 | Ptpro | 0.35 |
| 6922301 | Akna | 0.35 |
| 6834560 | BC052328 | 0.35 |
| 6782139 | Rnf167 | 0.35 |
| 6870489 | Pdcd4 | 0.35 |
| 6817645 | Duxbl | 0.35 |
| 6962491 | Sytl2 | 0.35 |
| 6972411 | Tnfrsf26 | 0.34 |
| 6768898 | Vpreb3 | 0.34 |
| 6960248 | Klk1b4 | 0.34 |
| 6965107 | Olfr61 | 0.34 |
| 6781111 | Slc36a1 | 0.34 |
| 6840400 | Hes1 | 0.34 |
| 6941844 | Gpr109a | 0.34 |
| 6875666 | Pnpla7 | 0.34 |
| 6946558 | Herc3 | 0.34 |
| 6751362 | Ugt1a10 | 0.34 |
| 6888696 | Ptprj | 0.34 |
| 6789359 | Centb1 | 0.34 |
| 6840432 | BC022623 | 0.34 |
| 6962325 | Olfr295 | 0.34 |
| 6811068 | Lgals8 | 0.34 |
| 6839420 | Snx29 | 0.34 |
| 6908332 | Amy2 | 0.34 |
| 6917656 | Paqr7 | 0.34 |
| 6788928 | Zfp287 | 0.34 |
| 6846105 | Trat1 | 0.34 |
| 6965314 | Tspan32 | 0.34 |
| 6768207 | Prf1 | 0.34 |
| 6907137 | Tnfaip8l2 | 0.34 |
| 6964377 | B230325K18Rik | 0.34 |
| 6775559 | Slc41a2 | 0.34 |
| 6950103 | Clec2i | 0.34 |
| 6817903 | Chdh | 0.34 |
| 6988220 | Esam1 | 0.34 |
| 6939241 | Kdr | 0.34 |
| 6855240 | Olfr99 | 0.34 |
| 6966143 | Plekhg2 | 0.34 |
| 6891064 | Rassf2 | 0.34 |
| 6925519 | Tlr12 | 0.34 |
| 6966282 | Hcst | 0.34 |
| 6933973 | Slc24a6 | 0.34 |
| 6832719 | Cntn1 | 0.34 |
| 6890448 | Gatm | 0.34 |
| 6778057 | Dgka | 0.33 |
| 6850271 | Gabbr1 | 0.33 |
| 7021194 | Tspan7 | 0.33 |
| 6832580 | C730034F03Rik | 0.33 |
| 6969978 | Olfr610 | 0.33 |
| 6926987 | Spsb1 | 0.33 |
| 6892699 | Mafb | 0.33 |
| 6860929 | Snx24 | 0.33 |
| 6963566 | A630005I04Rik | 0.33 |
| 6791238 | Fbxl20 | 0.33 |
| 6872651 | Sgms1 | 0.33 |
| 6917696 | D4Wsu53e | 0.33 |
| 6845444 | Slc15a2 | 0.33 |
| 6954617 | Vamp5 | 0.33 |
| 6820219 | P2ry5 | 0.33 |
| 6796158 | Syne2 | 0.33 |
| 6900287 | ENSMUSG00000074335 | 0.33 |
| 6847878 | ORF28 | 0.33 |
| 6824967 | Lats2 | 0.33 |
| 6970084 | Olfr666 | 0.33 |
| 6807328 | Fbxl21 | 0.33 |
| 6955935 | AA589522 | 0.33 |
| 6849622 | Fgd2 | 0.33 |
| 6983056 | Psd3 | 0.33 |
| 7018804 | Cysltr1 | 0.33 |
| 6780551 | Havcr2 | 0.33 |
| 6996438 | Aph1c | 0.33 |
| 6807007 | Edg3 | 0.33 |
| 6954988 | Sema4f | 0.33 |
| 6968804 | Nmb | 0.33 |
| 6960404 | Dbp | 0.33 |
| 6873503 | Sh3pxd2a | 0.33 |
| 7015229 | Arhgap6 | 0.33 |
| 6788314 | Pdlim4 | 0.33 |
| 6923694 | Dock7 | 0.32 |
| 6933476 | Ttc28 | 0.32 |
| 6785294 | Tmc8 | 0.32 |
| 6887196 | Ifih1 | 0.32 |
| 6871117 | Ctsw | 0.32 |
| 6789604 | Olfr1 | 0.32 |
| 6899016 | Arhgef11 | 0.32 |
| 7018564 | Cxcr3 | 0.32 |
| 6999682 | Fyco1 | 0.32 |
| 6875964 | Gbgt1 | 0.32 |
| 6779855 | Stk10 | 0.32 |
| 6902880 | Gpr177 | 0.32 |
| 6819096 | OTTMUSG00000015027 | 0.32 |
| 6959968 | 1600014C10Rik | 0.32 |
| 6933474 | Ttc28 | 0.32 |
| 6792406 | Cd300lf | 0.32 |
| 6792390 | RP23 | 0.32 |
| 6880035 | D2Ertd127e | 0.32 |
| 6869324 | Ifit2 | 0.32 |
| 6769241 | 9030607L17Rik | 0.32 |
| 6887179 | Dpp4 | 0.32 |
| 6817616 | 4931406H21Rik | 0.32 |
| 6969631 | Aqp11 | 0.32 |
| 6759396 | Idh1 | 0.32 |
| 6857639 | AI605517 | 0.32 |
| 6792614 | Socs3 | 0.32 |
| 6940236 | Antxr2 | 0.32 |
| 6785173 | 2210020M01Rik | 0.32 |
| 6768157 | 4632428N05Rik | 0.32 |
| 6855246 | H2 | 0.32 |
| 6914009 | Orm2 | 0.32 |
| 6987932 | Kcnj1 | 0.32 |
| 7020286 | Mageh1 | 0.32 |
| 6950384 | Crebl2 | 0.32 |
| 6769282 | Tbxa2r | 0.32 |
| 6850552 | Enpp5 | 0.32 |
| 6832005 | Grap2 | 0.32 |
| 6833305 | Acvrl1 | 0.32 |
| 6757898 | Lincr | 0.32 |
| 6945788 | Tas2r143 | 0.32 |
| 6876740 | A430068E04Rik | 0.31 |
| 6868096 | Ms4a4b | 0.31 |
| 6887079 | Cd302 | 0.31 |
| 6758704 | Pgap1 | 0.31 |
| 6990922 | Sh3bgrl2 | 0.31 |
| 6879637 | Lmo2 | 0.31 |
| 6838754 | Calcoco1 | 0.31 |
| 6850128 | Ltb | 0.31 |
| 6941637 | Tpcn1 | 0.31 |
| 6748893 | Il18rap | 0.31 |
| 6977151 | Klf2 | 0.31 |
| 6996678 | Aqp9 | 0.31 |
| 6790046 | Evi2a | 0.31 |
| 6815345 | Hexb | 0.31 |
| 6989917 | Parp16 | 0.31 |
| 6955268 | Arhgap25 | 0.31 |
| 6881100 | Zc3h6 | 0.31 |
| 6888397 | Olfr1006 | 0.31 |
| 6785317 | Socs3 | 0.31 |
| 6815268 | F2rl1 | 0.31 |
| 6993708 | Fbxl12 | 0.31 |
| 6847540 | App | 0.31 |
| 6807228 | Rgs14 | 0.31 |
| 6891880 | Cd93 | 0.31 |
| 6868098 | Ms4a6c | 0.31 |
| 6787099 | Il9r | 0.31 |
| 6825679 | 9930012K11Rik | 0.31 |
| 6837008 | Csf2rb2 | 0.31 |
| 6965984 | Cyp2s1 | 0.31 |
| 6957406 | Clec7a | 0.31 |
| 6794552 | Tmem195 | 0.31 |
| 6941657 | Oas1a | 0.31 |
| 6940841 | Tgfbr3 | 0.31 |
| 6978695 | Cdh5 | 0.30 |
| 6870424 | Add3 | 0.30 |
| 6968780 | Fes | 0.30 |
| 6774384 | Tspan15 | 0.30 |
| 6955376 | Abtb1 | 0.30 |
| 6855825 | A530064D06Rik | 0.30 |
| 6856676 | Rab31 | 0.30 |
| 6877139 | Fmnl2 | 0.30 |
| 6765327 | G0s2 | 0.30 |
| 6926023 | Man1c1 | 0.30 |
| 6806435 | Gcnt2 | 0.30 |
| 6751442 | Centg2 | 0.30 |
| 6960516 | Tmem86a | 0.30 |
| 6991538 | Pcolce2 | 0.30 |
| 6977975 | Adcy7 | 0.30 |
| 6767336 | Cdc2l6 | 0.30 |
| 6790960 | Acsf2 | 0.30 |
| 6957437 | Klra10 | 0.30 |
| 6871545 | Ms4a14 | 0.30 |
| 6777510 | Irak3 | 0.30 |
| 7013389 | Klhl4 | 0.30 |
| 6975052 | Rnf122 | 0.30 |
| 6993761 | Cdkn2d | 0.30 |
| 6938631 | Rell1 | 0.30 |
| 6837787 | Plxnb2 | 0.30 |
| 6995899 | Scamp5 | 0.30 |
| 6759621 | Fn1 | 0.30 |
| 6883640 | Spo11 | 0.30 |
| 6957443 | Klra10 | 0.30 |
| 6798271 | Adssl1 | 0.30 |
| 6957442 | Klra10 | 0.30 |
| 6805430 | Cmah | 0.30 |
| 6936076 | Adam22 | 0.30 |
| 6763787 | Rcsd1 | 0.30 |
| 6850308 | Olfr110 | 0.30 |
| 6979626 | 9330133O14Rik | 0.30 |
| 6993025 | Ccbp2 | 0.29 |
| 6970053 | Trim12 | 0.29 |
| 6988898 | D930028F11Rik | 0.29 |
| 6796196 | Plekhg3 | 0.29 |
| 6934002 | Oas1d | 0.29 |
| 6787918 | Psme2 | 0.29 |
| 6791230 | Arl5c | 0.29 |
| 7017573 | Xlr5a | 0.29 |
| 6854487 | Dusp1 | 0.29 |
| 6850055 | Gpsm3 | 0.29 |
| 6774021 | 9530009G21Rik | 0.29 |
| 7011949 | Abcd1 | 0.29 |
| 6799874 | Hbp1 | 0.29 |
| 6913193 | 1300002K09Rik | 0.29 |
| 6775472 | Gna15 | 0.29 |
| 6836954 | Apol7b | 0.29 |
| 6980103 | Cd209b | 0.29 |
| 6925369 | Eif2c4 | 0.29 |
| 6915818 | Raver2 | 0.29 |
| 6869754 | Frat1 | 0.29 |
| 6763146 | Npl | 0.29 |
| 6775288 | 2610008E11Rik | 0.29 |
| 6804898 | Lyst | 0.29 |
| 6955778 | Frmd4b | 0.29 |
| 6835938 | Fbxo32 | 0.28 |
| 6782808 | Centa2 | 0.28 |
| 6806831 | Rnf144b | 0.28 |
| 6940595 | Hsd17b11 | 0.28 |
| 6878035 | Pdk1 | 0.28 |
| 6992994 | Vipr1 | 0.28 |
| 6852130 | Ypel5 | 0.28 |
| 6932367 | Ppbp | 0.28 |
| 6996432 | Aph1b | 0.28 |
| 6949844 | Acrbp | 0.28 |
| 6990525 | BC031353 | 0.28 |
| 6791437 | Dhx58 | 0.28 |
| 6888376 | Olfr994 | 0.28 |
| 6839340 | Ciita | 0.28 |
| 6765321 | Traf3ip3 | 0.28 |
| 6853662 | Qk | 0.28 |
| 6782286 | Itgae | 0.28 |
| 6952926 | Parp12 | 0.28 |
| 6864700 | Cd14 | 0.28 |
| 6839919 | Klhl24 | 0.28 |
| 6951882 | Tcfec | 0.28 |
| 6983820 | ENSMUSG00000074215 | 0.28 |
| 6886678 | Rnd3 | 0.28 |
| 7022706 | LOC100039393 | 0.28 |
| 6799897 | Pik3cg | 0.28 |
| 6847709 | Krtap16 | 0.28 |
| 6783337 | Bzrap1 | 0.28 |
| 6975367 | Ppp1r3b | 0.28 |
| 6816160 | Gzma | 0.28 |
| 6987384 | AB124611 | 0.28 |
| 6958078 | St8sia1 | 0.28 |
| 6935120 | BC055004 | 0.28 |
| 6957365 | Klrb1b | 0.28 |
| 6767402 | Sesn1 | 0.27 |
| 6957428 | Klri2 | 0.27 |
| 6962094 | Tm6sf1 | 0.27 |
| 6849891 | Cyp4f16 | 0.27 |
| 6881895 | BC039771 | 0.27 |
| 6880776 | Sema6d | 0.27 |
| 6921379 | Trim14 | 0.27 |
| 6906749 | Sema4a | 0.27 |
| 6784042 | Nr1d1 | 0.27 |
| 6762092 | Cd55 | 0.27 |
| 6887324 | Scn3a | 0.27 |
| 6791960 | Pecam1 | 0.27 |
| 6755714 | Tmem63a | 0.27 |
| 6916540 | Pik3r3 | 0.27 |
| 6855727 | A330017A19Rik | 0.27 |
| 6983076 | 9530019H20Rik | 0.27 |
| 6805108 | Naip3 | 0.27 |
| 6769033 | Icosl | 0.27 |
| 6848179 | Dscam | 0.27 |
| 6815027 | Rasgrf2 | 0.27 |
| 6916937 | Mycl1 | 0.27 |
| 6892486 | Sla2 | 0.27 |
| 6908345 | Amy2 | 0.27 |
| 6803525 | A130014H13Rik | 0.27 |
| 6986722 | Mmp12 | 0.27 |
| 6899746 | Hist2h2be | 0.26 |
| 6805180 | Aoah | 0.26 |
| 6956507 | Srgap3 | 0.26 |
| 6868017 | Rab3il1 | 0.26 |
| 6781368 | Nlrp3 | 0.26 |
| 6988855 | Cadm1 | 0.26 |
| 6918125 | Padi2 | 0.26 |
| 6805106 | Tcrg | 0.26 |
| 6894345 | Olfr1138 | 0.26 |
| 6957432 | Klra17 | 0.26 |
| 6769272 | Matk | 0.26 |
| 6783762 | Zfp652 | 0.26 |
| 6845366 | Dirc2 | 0.26 |
| 6782579 | 1300007F04Rik | 0.26 |
| 6806073 | Nqo2 | 0.26 |
| 6878949 | Olfr1265 | 0.26 |
| 6769181 | Hmha1 | 0.26 |
| 6939985 | Naaa | 0.26 |
| 6849466 | A930001N09Rik | 0.26 |
| 6868884 | Smarca2 | 0.26 |
| 6951282 | AI987662 | 0.26 |
| 6837127 | Unc84b | 0.26 |
| 6803161 | Lgmn | 0.26 |
| 6870958 | Aldh3b1 | 0.26 |
| 6781104 | Gm2a | 0.26 |
| 6992280 | Ube1l | 0.26 |
| 6797544 | D12Ertd647e | 0.26 |
| 6905422 | P2ry13 | 0.26 |
| 6941649 | Oas3 | 0.26 |
| 6856278 | Dennd1c | 0.25 |
| 6818915 | Tlr11 | 0.25 |
| 6930006 | Jakmip1 | 0.25 |
| 6991192 | Nt5e | 0.25 |
| 6916023 | Ppap2b | 0.25 |
| 6815535 | Naip1 | 0.25 |
| 6764175 | Slamf8 | 0.25 |
| 6959474 | Rasgrp4 | 0.25 |
| 6960140 | Siglecg | 0.25 |
| 6958974 | Pglyrp1 | 0.25 |
| 6999688 | Ccr1 | 0.25 |
| 6937288 | Mxd4 | 0.25 |
| 6816247 | Itga2 | 0.25 |
| 6890981 | Siglec1 | 0.25 |
| 6921190 | Olfr159 | 0.25 |
| 6924288 | 2210012G02Rik | 0.25 |
| 6880718 | Sqrdl | 0.25 |
| 6872646 | Asah2 | 0.25 |
| 6998434 | Acpp | 0.25 |
| 6818956 | Ang | 0.25 |
| 6965950 | Ceacam2 | 0.25 |
| 6862627 | Zfp516 | 0.25 |
| 6923142 | Gdap6 | 0.25 |
| 6988819 | 2900052N01Rik | 0.25 |
| 6857769 | Haao | 0.25 |
| 6867618 | Aldh3b2 | 0.25 |
| 6965901 | Atp1a3 | 0.25 |
| 6990427 | Ccpg1 | 0.25 |
| 6921157 | Sit1 | 0.24 |
| 6813096 | Fgd3 | 0.24 |
| 6782989 | Slfn1 | 0.24 |
| 6844210 | Fgd4 | 0.24 |
| 6753417 | Kif21b | 0.24 |
| 6781925 | Pik3r5 | 0.24 |
| 7010645 | Il13ra1 | 0.24 |
| 6933812 | Tesc | 0.24 |
| 6895856 | Car2 | 0.24 |
| 6940363 | Hpse | 0.24 |
| 6775471 | Edg6 | 0.24 |
| 6775762 | Stab2 | 0.24 |
| 6976901 | Lpl | 0.24 |
| 6769597 | Igf1 | 0.24 |
| 6751623 | Gpr35 | 0.24 |
| 6837935 | Abcd2 | 0.24 |
| 6879994 | Olfr1280 | 0.24 |
| 6843601 | Mefv | 0.24 |
| 6879020 | Sfpi1 | 0.24 |
| 6941146 | Adrbk2 | 0.24 |
| 6854310 | Tmprss8 | 0.24 |
| 6785384 | Rnf213 | 0.24 |
| 6783785 | Gngt2 | 0.24 |
| 6784765 | Axin2 | 0.24 |
| 6959421 | Gmfg | 0.24 |
| 6938679 | Tlr6 | 0.24 |
| 6880694 | RP23 | 0.24 |
| 6766839 | A130091G23Rik | 0.24 |
| 6765551 | Syne1 | 0.24 |
| 6799524 | Rnf144a | 0.23 |
| 6898972 | Cd1d2 | 0.23 |
| 6808221 | Slc12a7 | 0.23 |
| 6956765 | Rassf4 | 0.23 |
| 6976765 | March1 | 0.23 |
| 6945611 | Rab19 | 0.23 |
| 6782422 | Rtn4rl1 | 0.23 |
| 6811689 | Hist1h2ac | 0.23 |
| 6962880 | Arrb1 | 0.23 |
| 6774264 | Ddit4 | 0.23 |
| 6838716 | Itgb7 | 0.23 |
| 6962027 | Zscan2 | 0.23 |
| 6863783 | B4galt6 | 0.23 |
| 6932540 | Ccng2 | 0.23 |
| 6870580 | Tcf7l2 | 0.23 |
| 7017600 | L1cam | 0.23 |
| 6841201 | Gcet2 | 0.23 |
| 6755237 | Kcnj10 | 0.23 |
| 6950391 | Cdkn1b | 0.22 |
| 6763208 | Mr1 | 0.22 |
| 6784062 | Igfbp4 | 0.22 |
| 6929119 | Fgl2 | 0.22 |
| 6949202 | Pparg | 0.22 |
| 7017663 | Gab3 | 0.22 |
| 6885855 | A130092J06Rik | 0.22 |
| 6965982 | Axl | 0.22 |
| 6815522 | Naip2 | 0.22 |
| 6824838 | Tgm1 | 0.22 |
| 6879185 | C230071H18Rik | 0.22 |
| 6811368 | A530099J19Rik | 0.22 |
| 6876380 | Gsn | 0.22 |
| 6800020 | Hdac9 | 0.22 |
| 6988315 | Olfr930 | 0.22 |
| 6942956 | 2210010N04Rik | 0.22 |
| 6809655 | Cd180 | 0.22 |
| 6959600 | Tmem149 | 0.22 |
| 6942491 | Orai2 | 0.22 |
| 6755378 | Kmo | 0.22 |
| 6917389 | Sdc3 | 0.22 |
| 6943142 | Flt3 | 0.22 |
| 6866852 | Slc14a1 | 0.22 |
| 6939076 | Txk | 0.22 |
| 6915619 | Nfia | 0.22 |
| 6983799 | Cd97 | 0.21 |
| 6766577 | Vnn3 | 0.21 |
| 6782451 | Slc43a2 | 0.21 |
| 6878448 | Itga4 | 0.21 |
| 6882352 | Hck | 0.21 |
| 6761701 | Marco | 0.21 |
| 6806701 | Mylip | 0.21 |
| 6957679 | Hebp1 | 0.21 |
| 6764650 | Ephx1 | 0.21 |
| 6783321 | Sept4 | 0.21 |
| 6970166 | EG209380 | 0.21 |
| 6894951 | C230071H18Rik | 0.21 |
| 6776404 | Phxr2 | 0.21 |
| 6777784 | Slc16a7 | 0.21 |
| 6780696 | Trim7 | 0.21 |
| 6762345 | Btg2 | 0.21 |
| 6791233 | Cacnb1 | 0.21 |
| 6930383 | Cd38 | 0.21 |
| 6884520 | Cugbp2 | 0.21 |
| 6789544 | Spns3 | 0.21 |
| 6854231 | A630033E08Rik | 0.21 |
| 6789325 | Cd68 | 0.21 |
| 6838565 | Pou6f1 | 0.21 |
| 6789329 | Tnfsf12 | 0.21 |
| 6792122 | Abca9 | 0.21 |
| 6893057 | Sulf2 | 0.21 |
| 6857645 | Slc8a1 | 0.21 |
| 6850819 | Trem3 | 0.21 |
| 6762429 | Nav1 | 0.20 |
| 6897845 | Sucnr1 | 0.20 |
| 6820323 | 5031414D18Rik | 0.20 |
| 7020314 | Kctd12b | 0.20 |
| 6907784 | Olfml3 | 0.20 |
| 6963137 | Trim34 | 0.20 |
| 6980606 | 2610019F03Rik | 0.20 |
| 6931759 | Kit | 0.20 |
| 6841140 | Cd200r4 | 0.20 |
| 7013206 | A630033H20Rik | 0.20 |
| 6959223 | B3gnt8 | 0.20 |
| 6952470 | AB041803 | 0.20 |
| 6892899 | Slpi | 0.20 |
| 6998893 | Tmie | 0.20 |
| 6760289 | 5033414K04Rik | 0.20 |
| 6980101 | Cd209d | 0.19 |
| 6833232 | ENSMUSG00000058057 | 0.19 |
| 6950147 | Clec1b | 0.19 |
| 6933627 | Oasl1 | 0.19 |
| 6933997 | Oas1b | 0.19 |
| 6970857 | Gprc5b | 0.19 |
| 6915844 | Dnajc6 | 0.19 |
| 6852181 | Ehd3 | 0.19 |
| 6856756 | Ptprm | 0.19 |
| 6964274 | AI467606 | 0.19 |
| 7010092 | Gpr34 | 0.19 |
| 6857183 | Xdh | 0.19 |
| 6791063 | Abi3 | 0.19 |
| 6840115 | BB163080 | 0.19 |
| 6992475 | Als2cl | 0.19 |
| 6755091 | Sh2d1b1 | 0.19 |
| 7017601 | Arhgap4 | 0.19 |
| 6837375 | Cyp2d22 | 0.19 |
| 6868196 | Olfr1445 | 0.19 |
| 6816025 | 9830130M13Rik | 0.19 |
| 6796691 | Fos | 0.19 |
| 6808339 | Mctp1 | 0.19 |
| 6943476 | Rbm47 | 0.19 |
| 6882730 | Lbp | 0.19 |
| 6876944 | 2310010M24Rik | 0.19 |
| 6935970 | Pftk1 | 0.19 |
| 7023919 | LOC100040031 | 0.19 |
| 6957044 | Apobec1 | 0.19 |
| 6829549 | Cmbl | 0.19 |
| 6780572 | Timd4 | 0.19 |
| 6953800 | Nod1 | 0.19 |
| 6792373 | AF251705 | 0.18 |
| 6963456 | Ampd3 | 0.18 |
| 6989015 | Il18 | 0.18 |
| 6850537 | Cyp39a1 | 0.18 |
| 6841019 | Zbtb20 | 0.18 |
| 6911337 | Lyn | 0.18 |
| 6980578 | Rasa3 | 0.18 |
| 6852399 | Qpct | 0.18 |
| 6943387 | N4bp2l1 | 0.18 |
| 6849766 | Abcg1 | 0.18 |
| 6998069 | BC043934 | 0.18 |
| 6870979 | AI790298 | 0.18 |
| 6892579 | Tgm2 | 0.18 |
| 6844601 | BC106179 | 0.18 |
| 6805288 | V1ri3 | 0.18 |
| 6886039 | Stom | 0.18 |
| 6917789 | Cnr2 | 0.18 |
| 6969753 | Slco2b1 | 0.18 |
| 6938678 | Tlr1 | 0.18 |
| 6899835 | Fmo5 | 0.18 |
| 6753089 | Slc45a3 | 0.18 |
| 6764231 | BC094916 | 0.17 |
| 6768618 | Slc16a9 | 0.17 |
| 6982102 | Tlr3 | 0.17 |
| 6852836 | Epas1 | 0.17 |
| 6861751 | D18Ertd653e | 0.17 |
| 6994927 | Sorl1 | 0.17 |
| 6818958 | Rnase6 | 0.17 |
| 6940431 | Wdfy3 | 0.17 |
| 6849474 | Phf1 | 0.17 |
| 6792649 | Timp2 | 0.17 |
| 6828522 | Lifr | 0.17 |
| 6900385 | Sort1 | 0.17 |
| 6791422 | Jup | 0.17 |
| 6757322 | EG241041 | 0.17 |
| 6783040 | OTTMUSG00000000971 | 0.17 |
| 6784587 | Ace3 | 0.16 |
| 6813407 | Dok3 | 0.16 |
| 6754143 | Rnasel | 0.16 |
| 6962133 | A530021J07Rik | 0.16 |
| 6830506 | Col14a1 | 0.16 |
| 6985252 | Hp | 0.16 |
| 6973527 | Lair1 | 0.16 |
| 6764274 | Ifi203 | 0.16 |
| 6763196 | Cacna1e | 0.16 |
| 6823653 | Il17rb | 0.16 |
| 6820472 | Epsti1 | 0.16 |
| 6914190 | Tlr4 | 0.16 |
| 6941645 | Dtx1 | 0.16 |
| 6828480 | Fyb | 0.16 |
| 6850831 | A530064D06Rik | 0.16 |
| 6949732 | Clec4b1 | 0.16 |
| 6968735 | Anpep | 0.16 |
| 7008556 | Ear1 | 0.16 |
| 6911010 | Sort1 | 0.16 |
| 6949856 | Vamp1 | 0.16 |
| 6991461 | Slc9a9 | 0.15 |
| 6784844 | Kcnj16 | 0.15 |
| 6841136 | Cd200r1 | 0.15 |
| 6866021 | Adrb2 | 0.15 |
| 6918015 | Pla2g2d | 0.15 |
| 6762804 | Rgs18 | 0.15 |
| 6899760 | Txnip | 0.15 |
| 6785632 | Tcn2 | 0.15 |
| 6931961 | Lphn3 | 0.15 |
| 6907941 | Bclp2 | 0.15 |
| 6992855 | Itga9 | 0.15 |
| 6949730 | Clec4a4 | 0.15 |
| 6899372 | S100a4 | 0.15 |
| 6966808 | Cd33 | 0.15 |
| 6892964 | Pltp | 0.15 |
| 6964246 | Ypel3 | 0.15 |
| 6993865 | Acp5 | 0.15 |
| 6883125 | Mmp9 | 0.15 |
| 6764040 | Fcgr3 | 0.15 |
| 6964247 | Tbx6 | 0.15 |
| 6939069 | Cnga1 | 0.15 |
| 6940432 | Wdfy3 | 0.15 |
| 6787925 | 9930111J21Rik | 0.15 |
| 6957025 | Klrg1 | 0.15 |
| 6898719 | Ctso | 0.14 |
| 6768868 | Ggt5 | 0.14 |
| 6963006 | Pde2a | 0.14 |
| 6888752 | Nr1h3 | 0.14 |
| 6977648 | Tbc1d9 | 0.14 |
| 6840637 | Itgb5 | 0.14 |
| 6851897 | Epb4 | 0.14 |
| 6784829 | Map2k6 | 0.14 |
| 6983299 | Cyp4f18 | 0.14 |
| 6869334 | Ifit1 | 0.14 |
| 6869635 | Entpd1 | 0.14 |
| 6824763 | Slc7a8 | 0.14 |
| 6957758 | Art4 | 0.14 |
| 6950582 | Mgst1 | 0.14 |
| 6822443 | Dnase1l3 | 0.14 |
| 6777957 | Lrp1 | 0.14 |
| 6958071 | St8sia1 | 0.14 |
| 6815529 | Birc1f | 0.14 |
| 6927456 | Cnga1 | 0.14 |
| 6945584 | Tbxas1 | 0.14 |
| 6957458 | Klra2 | 0.13 |
| 6883186 | Eya2 | 0.13 |
| 6766455 | Sgk1 | 0.13 |
| 6933441 | A630023P12Rik | 0.13 |
| 6840112 | B630019A10Rik | 0.13 |
| 6750566 | Cyp27a1 | 0.13 |
| 6784527 | Itgb3 | 0.13 |
| 6798285 | Pld4 | 0.13 |
| 6815523 | Naip5 | 0.13 |
| 6813474 | Tifab | 0.13 |
| 6825600 | Adamdec1 | 0.13 |
| 6899374 | S100a6 | 0.13 |
| 6792368 | Cd300c | 0.13 |
| 6869509 | Hhex | 0.13 |
| 6755146 | Fcgr4 | 0.13 |
| 6820113 | Gfra2 | 0.13 |
| 6779827 | Nsg2 | 0.13 |
| 6843550 | Mx1 | 0.12 |
| 6792392 | Cd300e | 0.12 |
| 6769445 | Tcp11l2 | 0.12 |
| 6828326 | Sepp1 | 0.12 |
| 6749935 | Adam23 | 0.12 |
| 6890838 | Il1b | 0.12 |
| 6855084 | Aif1 | 0.12 |
| 6940658 | Abcg3 | 0.12 |
| 6801506 | Pygl | 0.12 |
| 6753067 | Ctse | 0.12 |
| 6848199 | Mx1 | 0.12 |
| 6964798 | Ptpre | 0.12 |
| 6993153 | Ccr2 | 0.12 |
| 6973679 | A430078G23Rik | 0.12 |
| 6854043 | Fpr1 | 0.12 |
| 6949766 | Cd163 | 0.12 |
| 6785114 | Rab37 | 0.12 |
| 6793961 | Cmpk2 | 0.12 |
| 6811694 | Hfe | 0.11 |
| 6965609 | Gpr77 | 0.11 |
| 6792367 | Cd300lb | 0.11 |
| 6751535 | Ramp1 | 0.11 |
| 6775236 | Trpm2 | 0.11 |
| 6905408 | P2ry14 | 0.11 |
| 6755189 | Cd244 | 0.11 |
| 6748889 | Il18r1 | 0.11 |
| 6973587 | Apoe | 0.11 |
| 6892032 | Acss1 | 0.11 |
| 6946370 | 2410066E13Rik | 0.11 |
| 7020800 | Tlr8 | 0.11 |
| 6782979 | Slfn5 | 0.11 |
| 6957427 | Klri1 | 0.11 |
| 6760518 | Arl4c | 0.11 |
| 6960198 | Klk1b27 | 0.11 |
| 6837415 | Nfam1 | 0.11 |
| 6906635 | Cd1d1 | 0.11 |
| 6785111 | Cd300a | 0.11 |
| 6920954 | Ddx58 | 0.10 |
| 6811370 | Gpr141 | 0.10 |
| 6970060 | A530023O14Rik | 0.10 |
| 6954269 | Ptgds2 | 0.10 |
| 6790944 | Abcc3 | 0.10 |
| 6824728 | Slc7a7 | 0.10 |
| 6762024 | Cxcr4 | 0.10 |
| 6772009 | Sash1 | 0.10 |
| 6921670 | Abca1 | 0.10 |
| 6972491 | Ccnd1 | 0.10 |
| 6878038 | Rapgef4 | 0.10 |
| 6765460 | Cr2 | 0.10 |
| 7019499 | Xkrx | 0.10 |
| 6790290 | Ccl6 | 0.10 |
| 6767468 | Scml4 | 0.10 |
| 6872010 | Anxa1 | 0.10 |
| 6910592 | Ifi44 | 0.10 |
| 6868229 | Olfr1484 | 0.10 |
| 6934162 | P2rx7 | 0.09 |
| 6799525 | Rsad2 | 0.09 |
| 6980091 | Cd209a | 0.09 |
| 6849951 | Myo1f | 0.09 |
| 6903360 | Sirpb1 | 0.09 |
| 6977260 | Hmox1 | 0.09 |
| 6960834 | Siglech | 0.09 |
| 6829612 | Pgcp | 0.09 |
| 6827820 | Dzip1 | 0.09 |
| 6876430 | Ptgs1 | 0.09 |
| 6964380 | Itgam | 0.09 |
| 6942580 | Pilrb1 | 0.09 |
| 6850534 | Pla2g7 | 0.09 |
| 6762094 | Cd55 | 0.09 |
| 6969874 | Art2a | 0.08 |
| 6768572 | Tmem26 | 0.08 |
| 6768076 | Smpdl3a | 0.08 |
| 7019818 | Tsc22d3 | 0.08 |
| 6950148 | Clec9a | 0.08 |
| 6750546 | Slc11a1 | 0.08 |
| 6917549 | Fgr | 0.08 |
| 6966935 | Fcgrt | 0.08 |
| 6933625 | Oasl2 | 0.08 |
| 6869327 | Ifit3 | 0.08 |
| 6991531 | Paqr9 | 0.07 |
| 6951281 | Samd9l | 0.07 |
| 6957744 | 1100001H23Rik | 0.07 |
| 6949727 | Clec4a3 | 0.07 |
| 6792832 | Cd7 | 0.07 |
| 6778284 | Pik3ip1 | 0.07 |
| 6807336 | Tgfbi | 0.07 |
| 6840129 | Rtp4 | 0.07 |
| 6959584 | Tyrobp | 0.07 |
| 6749933 | Adam23 | 0.07 |
| 6941647 | Oas2 | 0.07 |
| 6764049 | Fcer1g | 0.07 |
| 6950170 | Klrd1 | 0.07 |
| 6978232 | Capns2 | 0.07 |
| 6949744 | Clec4n | 0.07 |
| 6917120 | Csf3r | 0.07 |
| 6954615 | Vamp5 | 0.07 |
| 6777310 | Lyz1 | 0.07 |
| 6977019 | Lrrc25 | 0.07 |
| 6972192 | Irf7 | 0.07 |
| 6828403 | C6 | 0.07 |
| 6943168 | Slc46a3 | 0.06 |
| 7013185 | Tlr13 | 0.06 |
| 6885432 | Fcna | 0.06 |
| 6950137 | Clec12a | 0.06 |
| 6935701 | Alox5ap | 0.06 |
| 6881139 | Sirpa | 0.06 |
| 6939761 | Igj | 0.06 |
| 6970952 | Igsf6 | 0.06 |
| 6867860 | Rasgrp2 | 0.06 |
| 6792371 | 4732429D16Rik | 0.06 |
| 6966818 | Siglece | 0.06 |
| 6881087 | Mertk | 0.06 |
| 6833937 | Il7r | 0.05 |
| 6850821 | Treml4 | 0.05 |
| 6849761 | Dnahc8 | 0.05 |
| 6765325 | Hsd11b1 | 0.05 |
| 6908486 | Vcam1 | 0.05 |
| 6926167 | C1qa | 0.05 |
| 6908461 | S1pr1 | 0.05 |
| 6949722 | Clec4a1 | 0.05 |
| 6869691 | Dntt | 0.05 |
| 6836325 | Tmem71 | 0.04 |
| 6775864 | Spic | 0.04 |
| 7006322 | Ear10 | 0.04 |
| 6964382 | Itgad | 0.04 |
| 6878045 | Rapgef4 | 0.04 |
| 6926166 | C1qc | 0.04 |
| 6942579 | Pilra | 0.04 |
| 6976609 | Ddx60 | 0.03 |
| 6998397 | Trf | 0.03 |
| 6851186 | Emr4 | 0.03 |
| 6898995 | Cd5l | 0.03 |
| 6851324 | Emr1 | 0.03 |
| 6993138 | Ccr9 | 0.03 |
| 6861358 | Csf1r | 0.03 |
| 6875181 | Mrc1 | 0.03 |
| 6777309 | Lyz1 | 0.02 |
| 6976237 | Hpgd | 0.02 |
| 6926165 | C1qb | 0.02 |
| 6969878 | Art2b | 0.02 |
| 6872785 | OTTMUSG00000016644 | 0.02 |
| 6993151 | Ccr3 | 0.01 |
| 6758435 | Slc40a1 | 0.01 |

**140 genes with greater than 2-fold change in transcript-level expression: TCR/CD28 activation as compared to TCR activation**

| **TCID** | **Symbol** | **Fold change** |
| --- | --- | --- |
| 6788333 | Il3 | 6.48 |
| 6862922 | Cd226 | 5.28 |
| 6904309 | Il2 | 4.98 |
| 7010183 | Maoa | 3.92 |
| 6916190 | Orc1l | 3.90 |
| 7018524 | Slc7a3 | 3.89 |
| 6760009 | Serpine2 | 3.86 |
| 7010871 | Sh2d1a | 3.53 |
| 6990042 | Car12 | 3.36 |
| 6781029 | Acsl6 | 3.31 |
| 6910938 | Cth | 3.30 |
| 7011413 | Cd40lg | 3.17 |
| 6790648 | Akap1 | 3.11 |
| 6755387 | Exo1 | 3.11 |
| 6789977 | Tmem97 | 2.93 |
| 6954982 | Hk2 | 2.88 |
| 6765235 | Dtl | 2.87 |
| 6958193 | Bcat1 | 2.85 |
| 6757744 | Bag2 | 2.83 |
| 7013857 | Cenpi | 2.82 |
| 6976002 | Dctd | 2.81 |
| 6884441 | Mcm10 | 2.80 |
| 6966600 | Ccne1 | 2.76 |
| 6933598 | Ung | 2.74 |
| 6824743 | Jub | 2.68 |
| 6854449 | 9530058B02Rik | 2.67 |
| 6869577 | Hells | 2.65 |
| 6775206 | Adarb1 | 2.62 |
| 6788791 | Shmt1 | 2.61 |
| 6925054 | Ctps | 2.58 |
| 6950115 | Klrb1f | 2.57 |
| 6977814 | Gpt2 | 2.55 |
| 7015392 | 2010204K13Rik | 2.53 |
| 6785684 | Nefh | 2.52 |
| 6901745 | Adh4 | 2.51 |
| 6758361 | Kdelc1 | 2.49 |
| 6759648 | Pecr | 2.44 |
| 6992409 | Nme6 | 2.43 |
| 6856231 | Ptprs | 2.43 |
| 6768324 | Dna2 | 2.43 |
| 6881340 | Mcm8 | 2.42 |
| 6792792 | Pycr1 | 2.42 |
| 6901962 | Ccbl2 | 2.40 |
| 6985703 | Mphosph6 | 2.39 |
| 6877924 | Bbs5 | 2.39 |
| 6785641 | 1700020C11Rik | 2.39 |
| 6992367 | Prkar2a | 2.33 |
| 6789237 | Rangrf | 2.33 |
| 6919209 | Tnfrsf4 | 2.33 |
| 6851204 | Chaf1a | 2.32 |
| 6849845 | Rrp1b | 2.31 |
| 6854690 | Ppil1 | 2.31 |
| 6765153 | Smyd2 | 2.31 |
| 6897908 | P2ry1 | 2.29 |
| 6854971 | Angptl4 | 2.29 |
| 6790318 | Tada2l | 2.28 |
| 6906840 | Cks1b | 2.27 |
| 6990859 | Irak1bp1 | 2.27 |
| 6768901 | Zfp280b | 2.25 |
| 6964253 | Hirip3 | 2.20 |
| 6983950 | Neto2 | 2.19 |
| 6952872 | Zc3hav1l | 2.17 |
| 6822959 | Ube2e2 | 2.16 |
| 6876150 | Exosc2 | 2.16 |
| 6998614 | Hemk1 | 2.10 |
| 7018220 | Pola1 | 2.10 |
| 6880853 | Dut | 2.09 |
| 6783137 | Myo19 | 2.07 |
| 6962880 | Arrb1 | 0.50 |
| 6815433 | Fcho2 | 0.50 |
| 6791238 | Fbxl20 | 0.50 |
| 6934854 | Gats | 0.48 |
| 6753067 | Ctse | 0.47 |
| 6904367 | EG381438 | 0.46 |
| 6934186 | A930024E05Rik | 0.46 |
| 6849474 | Phf1 | 0.46 |
| 6758646 | Inpp1 | 0.45 |
| 6754143 | Rnasel | 0.44 |
| 6785294 | Tmc8 | 0.43 |
| 6769181 | Hmha1 | 0.43 |
| 6876380 | Gsn | 0.42 |
| 6966143 | Plekhg2 | 0.42 |
| 6861751 | D18Ertd653e | 0.42 |
| 6959421 | Gmfg | 0.41 |
| 6992280 | Ube1l | 0.41 |
| 6856278 | Dennd1c | 0.41 |
| 6933997 | Oas1b | 0.40 |
| 6790531 | Ypel2 | 0.39 |
| 6799874 | Hbp1 | 0.39 |
| 6792649 | Timp2 | 0.39 |
| 6784527 | Itgb3 | 0.39 |
| 6905818 | Ift80 | 0.38 |
| 6952470 | AB041803 | 0.38 |
| 6942956 | 2210010N04Rik | 0.38 |
| 6951253 | 4833442J19Rik | 0.38 |
| 6844601 | BC106179 | 0.38 |
| 6782979 | Slfn5 | 0.37 |
| 6765321 | Traf3ip3 | 0.37 |
| 6915650 | Inadl | 0.37 |
| 6841019 | Zbtb20 | 0.37 |
| 6767336 | Cdc2l6 | 0.36 |
| 6838754 | Calcoco1 | 0.36 |
| 6849466 | A930001N09Rik | 0.35 |
| 6980578 | Rasa3 | 0.35 |
| 6899835 | Fmo5 | 0.34 |
| 6943387 | N4bp2l1 | 0.34 |
| 6867860 | Rasgrp2 | 0.34 |
| 6911679 | Trp53inp1 | 0.34 |
| 6899746 | Hist2h2be | 0.33 |
| 6839919 | Klhl24 | 0.33 |
| 6768076 | Smpdl3a | 0.32 |
| 6765325 | Hsd11b1 | 0.32 |
| 6767468 | Scml4 | 0.32 |
| 6757898 | Lincr | 0.32 |
| 6836954 | Apol7b | 0.32 |
| 6905408 | P2ry14 | 0.31 |
| 6791960 | Pecam1 | 0.31 |
| 6973679 | A430078G23Rik | 0.31 |
| 6871062 | Npas4 | 0.30 |
| 6962491 | Sytl2 | 0.28 |
| 6932540 | Ccng2 | 0.28 |
| 6970166 | EG209380 | 0.27 |
| 6964246 | Ypel3 | 0.27 |
| 6769445 | Tcp11l2 | 0.27 |
| 6946370 | 2410066E13Rik | 0.27 |
| 6764231 | BC094916 | 0.26 |
| 6785632 | Tcn2 | 0.26 |
| 6993138 | Ccr9 | 0.26 |
| 6945611 | Rab19 | 0.26 |
| 6778284 | Pik3ip1 | 0.25 |
| 6972411 | Tnfrsf26 | 0.24 |
| 6772594 | Myb | 0.23 |
| 6907204 | Ecm1 | 0.22 |
| 6945339 | Tmem140 | 0.21 |
| 6764650 | Ephx1 | 0.21 |
| 6908461 | S1pr1 | 0.15 |
| 6836325 | Tmem71 | 0.14 |
| 6969878 | Art2b | 0.14 |
| 6849761 | Dnahc8 | 0.14 |
| 6840112 | B630019A10Rik | 0.10 |
