# Supplementary material for: CD28 Costimulation Regulates Genome-Wide Effects on Alternative Splicing
Source: PLoS One. 2012 Jun 29;7(6):e40032. doi: 10.1371/journal.pone.0040032 (PMC3386953; doi:10.1371/journal.pone.0040032)
Supplement: Table S4 — Unique transcripts spliced only in naïve T cells, TCR-activated T cells, or TCR/CD28 T cells. (DOC) [file pone.0040032.s007.doc]

**Table S4.**

**Unique transcripts spliced only in naïve T cells, TCR-activated T cells, and TCR/CD28 T cells**

| **Naïve and TCR/Activated** | **Naïve and TCR/CD28 Activated** | **TCR and TCR/CD28 Activated** |
| --- | --- | --- |
| 0610009D07Rik | 0610007C21Rik | 1110003E01Rik |
| 1110004F10Rik | 0610007P22Rik | 1110007C09Rik |
| 1110057K04Rik | 1110031B06Rik | 1110059E24Rik |
| 1200009I06Rik | 1200014M14Rik | 1300010F03Rik |
| 1500011H22Rik | 1200016B10Rik | 1300018I17Rik |
| 1700021K19Rik | 1300018I05Rik | 1500010J02Rik |
| 1700102P08Rik | 130004C03 | 1700034H14Rik |
| 1810007M14Rik | 1500002O20Rik | 1810015C04Rik |
| 1810037C20Rik | 1500005I02Rik | 2310008H04Rik |
| 2010005J08Rik | 1500011K16Rik | 2310014G06Rik |
| 2010100O12Rik | 1500041N16Rik | 2310035C23Rik |
| 2310003L22Rik | 1700019D03Rik | 2310066E14Rik |
| 2310007F21Rik | 1700027J05Rik | 2310067B10Rik |
| 2310008H09Rik | 1700052N19Rik | 2610003J06Rik |
| 2310021P13Rik | 1700123O20Rik | 2610027C15Rik |
| 2310079N02Rik | 1810006K21Rik | 2610205E22Rik |
| 2410002I01Rik | 1810009O10Rik | 2810403A07Rik |
| 2410127L17Rik | 1810034K20Rik | 2810405J04Rik |
| 2510039O18Rik | 1810036I24Rik | 4930402H24Rik |
| 2610024E20Rik | 2210404J11Rik | 4933421E11Rik |
| 2810002N01Rik | 2310037I24Rik | 4933434E20Rik |
| 2810004N23Rik | 2310039H08Rik | 5730419I09Rik |
| 4632415K11Rik | 2310044H10Rik | 5830417I10Rik |
| 4921505C17Rik | 2400003C14Rik | 5930434B04Rik |
| 4930402E16Rik | 2410018C20Rik | 9130019O22Rik |
| 4930412F15Rik | 2410089E03Rik | 9130401M01Rik |
| 4931406P16Rik | 2610002M06Rik | 9430016H08Rik |
| 4932432K03Rik | 2610304G08Rik | A430093F15Rik |
| 5031414D18Rik | 2610507B11Rik | A430108C13Rik |
| 6230416J20Rik | 2810012G03Rik | A630042L21Rik |
| 6330416L07Rik | 2810021J22Rik | A930037G23Rik |
| 6430706D22Rik | 2810022L02Rik | Abca5 |
| 9430023L20Rik | 2810441K11Rik | Abcc5 |
| 9630033F20Rik | 3110002H16Rik | Abcg1 |
| A130012F09 | 3110003A22Rik | Acot7 |
| A630033H20Rik | 3110048E14Rik | Actr5 |
| A630049H14Rik | 3110062M04Rik | Adam8 |
| Abcb6 | 3830406C13Rik | Add3 |
| Abhd11 | 4631426J05Rik | Adfp |
| Ablim1 | 4632411B12Rik | Agpat5 |
| Acadsb | 4833413D08Rik | Akt2 |
| Accs | 4833418A01Rik | Aldoa |
| Acot11 | 4921513D23Rik | Alg8 |
| Acss2 | 4930427A07Rik | Ambra1 |
| Adarb1 | 4930451I11Rik | Ank |
| Aebp2 | 4930505N22Rik | Ankra2 |
| Aff1 | 4933427D14Rik | Anp32a |
| Aff4 | 5033414D02Rik | Anp32b |
| Agpat6 | 5230400G24Rik | Ap1s3 |
| Agtpbp1 | 5730453I16Rik | Ap2b1 |
| Ahdc1 | 5730472N09Rik | Ap4s1 |
| Ahi1 | 6030443O07Rik | Apoa1bp |
| AI480653 | 6030446N20Rik | Apobec3 |
| Akna | 6530403A03Rik | Apoo |
| Aldh9a1 | 6720456B07Rik | Appl1 |
| Alg1 | 6720456H20Rik | Arf3 |
| Als2cr13 | 9130404D08Rik | Arf4 |
| Ambn | A530088H08Rik | Arhgap22 |
| Amfr | A630028G03Rik | Arhgap28 |
| Ammecr1l | A930008G19Rik | Arhgap8 |
| Ankrd27 | A930024E05Rik | Arid2 |
| Ankrd52 | Aars2 | Arl4a |
| Ap1m1 | Abat | Arsa |
| Ap4b1 | Abca2 | Asah2 |
| Apaf1 | Abca3 | Asl |
| Apol7a | Abcc10 | Atg4b |
| Aqp3 | Abcg2 | Athl1 |
| Arcn1 | Abhd10 | Atic |
| Arhgap26 | Abhd2 | Atp11c |
| Arhgap5 | Abi3bp | Atp1b3 |
| Arhgef12 | Acad11 | Atp2a2 |
| Arhgef2 | Acadvl | Atp5g3 |
| Arhgef6 | Acat1 | Atp6ap2 |
| Arid4b | Acsl1 | Atp6v1a |
| Arl1 | Actb | Auh |
| Arl5a | Actg1 | Aurkb |
| Asb7 | Actn1 | Azin1 |
| Ascc2 | Actn2 | B230312A22Rik |
| Atad1 | Actn4 | B2m |
| Atad2 | Actr6 | B3gnt2 |
| Atf1 | Adamts7 | B4galnt1 |
| Atf7ip | Adar | Bag5 |
| Atg16l1 | Adcy2 | BC021381 |
| Atg9a | Aes | BC028454 |
| Atp1a2 | Aff3 | BC031353 |
| Atp5a1 | Aggf1 | Bcar3 |
| Atp5d | Agps | Bcl2a1b |
| Atp8a1 | Ahctf1 | Bcl9l |
| Atxn1 | Ahsa2 | Bdh2 |
| AU042671 | AI314760 | Bfar |
| Bag3 | AI597468 | Bnip2 |
| Bat3 | AI842396 | Brca2 |
| Bat4 | Akap10 | Brd4 |
| Bbs9 | Akr1b8 | Brd8 |
| BC025546 | Aktip | Bsdc1 |
| BC037438 | Aldh16a1 | Bsg |
| BC050196 | Aldh1a1 | Btf3 |
| BC066107 | Aldh6a1 | Bzw1 |
| BC085271 | Alg12 | C130006E23 |
| Birc3 | Alg2 | Camk4 |
| Bmp2k | Als2 | Capn1 |
| Bnip3l | Amz2 | Car7 |
| Brf1 | Anapc5 | Cbx6 |
| Btbd14b | Ankib1 | Ccar1 |
| Bub1b | Ankk1 | Ccdc111 |
| Bzw2 | Ankrd11 | Ccdc49 |
| C030011O14Rik | Ap1g1 | Ccdc62 |
| C030046G05 | Ap1s2 | Cd2 |
| C630004H02Rik | Ap2a2 | Cd300lf |
| C79407 | Ap3b1 | Cd320 |
| C80913 | Apbb1 | Cd74 |
| Cacna2d2 | Apip | Cd82 |
| Calu | Aplp2 | Cd97 |
| Cap1 | Aptx | Cdc25c |
| Carm1 | Aqr | Cdk2ap1 |
| Cbl | Arfrp1 | Cdk4 |
| Cbx2 | Arhgap1 | Cdk5rap1 |
| Ccbl1 | Arhgap10 | Cebpz |
| Ccdc127 | Arhgef3 | Centa1 |
| Ccdc25 | Arhgef4 | Centb1 |
| Ccdc43 | Arid1a | Cep290 |
| Ccdc5 | Arid4a | Cep63 |
| Ccdc52 | Arid5b | Chst12 |
| Cck | Arih1 | Cilp2 |
| Ccnt2 | Arih2 | Clcc1 |
| Cct5 | Arl8a | Clk1 |
| Cct7 | Arl8b | Clpb |
| Cd22 | Arnt | Cnot4 |
| Cd3e | Astn2 | Cnpy3 |
| Cd4 | Atg2a | Cog3 |
| Cd5 | Atp11a | Copz1 |
| Cd53 | Atp13a5 | Crot |
| Cd72 | Atp1a1 | Cs |
| Cd9 | Atp2b4 | Cse1l |
| Cdc20 | Atp2c1 | Csf1 |
| Cdc25b | Atp5c1 | Csf2rb2 |
| Cdk5rap2 | Atp5f1 | Cwc15 |
| Cdk8 | Atp5s | Cxcl16 |
| Cenpb | Atp6v1g1 | Cyp4v3 |
| Cept1 | Atp6v1h | D11Wsu99e |
| Cflar | Atp9b | D12Ertd551e |
| Chfr | Atrn | D15Wsu75e |
| Chmp4b | Atxn7 | D3Ucla1 |
| Chordc1 | Axin1 | D5Wsu178e |
| Chst10 | B130054P17 | Daam2 |
| Chst2 | B230208H17Rik | Dbf4 |
| Ciapin1 | Bach1 | Dclre1a |
| Clcf1 | Bai1 | Dcun1d1 |
| Cln3 | Banp | Ddb1 |
| Clptm1 | Bat2 | Ddef1 |
| Clptm1l | BB085087 | Ddx1 |
| Cmtm7 | BC019943 | Ddx18 |
| Cnn3 | BC027231 | Ddx21 |
| Cnot1 | BC032265 | Ddx25 |
| Coasy | BC037034 | Ddx39 |
| Cog6 | BC043098 | Ddx5 |
| Copb2 | BC051019 | Ddx51 |
| Cope | Bcl2 | Ddx55 |
| Cops7b | Bcl2l13 | Ddx58 |
| Cops8 | Bcor | Dennd4b |
| Coq9 | Becn1 | Dhrs7 |
| Coro1b | Birc6 | Dhrs7b |
| Cpsf1 | Bnip3 | Dicer1 |
| Crebl1 | Bptf | Dlg3 |
| Crtc3 | Brd9 | Dnajc11 |
| Csf2rb | Bscl2 | Dnajc12 |
| Csnk2b | Bst2 | Dnajc2 |
| Ctbp1 | Btbd1 | Dnmt3b |
| Ctdsp1 | Btbd7 | Dock11 |
| Cth | C130090K23Rik | Dpf2 |
| Cul1 | C1galt1c1 | Dph1 |
| Cul5 | C77563 | Dpm1 |
| Cyld | Calm3 | Dtnb |
| D030056L22Rik | Camk2d | Dtymk |
| D10Wsu52e | Camkk2 | Dysf |
| D14Ertd436e | Camta2 | Ebag9 |
| D14Ertd668e | Capn2 | Echdc1 |
| D1Ertd622e | Capn9 | Edem1 |
| D6Wsu163e | Capza2 | Eef1d |
| D930015E06Rik | Capzb | EG434197 |
| Dap3 | Casc4 | EG624362 |
| Dars | Cbfa2t2 | EG625603 |
| Dars2 | Cbfb | EG667723 |
| Dclk2 | Cby1 | Egf |
| Dcun1d5 | Cc2d1b | Eif2s3x |
| Ddi2 | Ccdc109b | Eif3c |
| Ddx17 | Ccdc134 | Elac2 |
| Ddx19a | Ccdc67 | Eml4 |
| Ddx27 | Ccdc79 | ENSMUSG00000073995 |
| Def8 | Ccdc93 | ENSMUSG00000075466 |
| Dek | Ccdc98 | Epb4 |
| Derl1 | Ccl5 | Eps15l1 |
| Dgat1 | Ccng1 | Erlin1 |
| Dhx29 | Ccnk | Ets1 |
| Dhx36 | Ccnl2 | Evi2a |
| Dhx38 | Ccnt1 | Exosc1 |
| Diablo | Ccr7 | Exosc3 |
| Dlat | Cd163l1 | Exosc7 |
| Dnajb5 | Cd2ap | F730014I05Rik |
| Dnajc17 | Cd3g | F730047E07Rik |
| Dnmt3a | Cd86 | Fancc |
| Dpysl2 | Cdadc1 | Fance |
| Drg1 | Cdc14b | Fanci |
| Dtx4 | Cdc16 | Fastkd5 |
| Dvl1 | Cdc42 | Fat3 |
| Dynlrb1 | Cdc42se1 | Fau |
| E430028B21Rik | Cdgap | Fbxl10 |
| Ech1 | Cdkn2aipnl | Fcer2a |
| Eef1g | Cdon | Flcn |
| Eef2k | Cdr2 | Fnbp1 |
| Efhd2 | Cep110 | Foxm1 |
| Ehbp1 | Cep135 | Foxp3 |
| Eif1b | Chd9 | Foxred2 |
| Eif2a | Chrng | Fras1 |
| Eif2ak4 | Ciao1 | Fryl |
| Eif2b4 | Cklf | Ftsj1 |
| Eif4a1 | Clasp2 | Ftsj3 |
| Eif4a3 | Clcn6 | Fyn |
| Eif4g1 | Cldn15 | Galnt1 |
| Elavl1 | Clec2d | Gars |
| Elovl1 | Clnk | Gatc |
| ENSMUSG00000072880 | Cnbp | Gcnt1 |
| ENSMUSG00000073391 | Cnot10 | Gdi2 |
| ENSMUSG00000074056 | Cnot6l | Ghitm |
| ENSMUSG00000074777 | Cnot8 | Glcci1 |
| Entpd7 | Cnpy2 | Gm1027 |
| Erap1 | Col1a1 | Gnl3 |
| Ercc3 | Commd8 | Gorasp1 |
| Ergic2 | Copa | Gpaa1 |
| Esrra | Cpne1 | Gps2 |
| Etfa | Cpne3 | Gramd1b |
| Etfb | Crkrs | Grn |
| Exoc5 | Crmp1 | Gsr |
| Exosc10 | Crtam | Gstz1 |
| Ezh1 | Cry2 | Gtf2h1 |
| Ezr | Csrp2bp | Gtpbp6 |
| Fap | Ctnnbl1 | H2 |
| Farsb | Ctps2 | Hagh |
| Fastkd1 | Cyb5r1 | Hars |
| Fdxr | D10Ertd641e | Hax1 |
| Fgf1 | D10Ertd709e | Hdgf |
| Fgfbp3 | D14Ertd449e | Heatr1 |
| Fgfr1op2 | D19Wsu162e | Herpud2 |
| Fis1 | D1Ertd704e | Hist1h2ae |
| Fkbp9 | D6Wsu116e | Hivep1 |
| Flii | Dapp1 | Hn1 |
| Flt3l | Ddx24 | Hrb |
| Foxo1 | Ddx46 | Hrc |
| Foxo3a | Ddx50 | Hsp90aa1 |
| Frmd4a | Decr1 | Hsp90aa1 |
| Frmd4b | Decr2 | Hsp90b1 |
| Frs2 | Depdc5 | Hspa5 |
| Ftl1 | Dhx15 | Hspa9 |
| Furin | Dhx16 | Hspd1 |
| Fxr1 | Dhx30 | Hyou1 |
| G3bp1 | Diap2 | Iah1 |
| G6pdx | Dis3l2 | Igf1r |
| Gad2 | Dlg1 | Igtp |
| Gadd45gip1 | Dmtf1 | Ik |
| Galns | Dnaja1 | Ikbkap |
| Gatad2b | Dnaja2 | Il15ra |
| Gbp6 | Dnajb10 | Il18r1 |
| Gemin5 | Dnajb12 | Ilf3 |
| Gfer | Dnase1 | Incenp |
| Gfpt1 | Dnm1l | Ints2 |
| Glrx2 | Dock3 | Ipo4 |
| Glrx5 | Dolpp1 | Ipo5 |
| Gm22 | Dph5 | Ipo7 |
| Gm650 | Dpy19l1 | Ipo9 |
| Gmds | Dullard | Iqcb1 |
| Gnb1l | Dusp19 | Irf4 |
| Golga1 | Dusp2 | Irf5 |
| Gorasp2 | Dusp22 | Itm2a |
| Gphn | Dynlt1 | Itpk1 |
| Gprc6a | E330026B02Rik | Itpr3 |
| Grina | Edf1 | Jag1 |
| Gripap1 | EG668319 | Jag2 |
| Gss | Egln1 | Jarid1c |
| Gtse1 | Ehd4 | Jmjd2b |
| H6pd | Eif2b2 | Kars |
| Hars2 | Eif2c2 | Kif15 |
| Hcls1 | Eif2c3 | Kif20a |
| Hdac2 | Eif3f | Kif21b |
| Hdac6 | Eif3m | Klc1 |
| Herc2 | Eif4a2 | Klhl5 |
| Herc4 | Elmo1 | Kpna2 |
| Hgsnat | Elmod2 | Krr1 |
| Hhat | Emg1 | Krt10 |
| Hmg20a | ENSMUSG00000056742 | Lag3 |
| Hmga1 | ENSMUSG00000072618 | Lasp1 |
| Hnrnpl | ENSMUSG00000073567 | Ldlrap1 |
| Hprt1 | ENSMUSG00000074023 | Lnp |
| Hps1 | ENSMUSG00000075275 | Lrrc1 |
| Hsd17b10 | Epc1 | Ly6f |
| Hspa4 | Eral1 | Lypla2 |
| Htf9c | Erbb2ip | Lysmd3 |
| Idh3g | Ercc2 | Macf1 |
| Ifi47 | Esco1 | Maff |
| Ifnar1 | Etaa1 | Maml1 |
| Ifrg15 | Etfdh | Mat2a |
| Ift52 | Ets2 | Mcm6 |
| Ift57 | Exoc3 | Med20 |
| Ihpk1 | Exoc6 | Mettl3 |
| Il10ra | Exoc7 | Mgrn1 |
| Il6st | Exosc5 | Mif |
| Inadl | Ext1 | Mkl1 |
| Inpp5b | Eya3 | Mknk1 |
| Inppl1 | Faah | Mms19 |
| Ipp | Fbrs | Mon1b |
| Iqgap1 | Fbxl11 | Mpped1 |
| Itfg1 | Fbxl19 | Mrpl18 |
| Itgal | Fbxo28 | Mrpl55 |
| Itgb2 | Fbxo42 | Mrps16 |
| Itpr2 | Fbxw4 | Mrps25 |
| Itsn2 | Fbxw7 | Mrps34 |
| Jak1 | Fermt3 | Mrps35 |
| Katnb1 | Fgfr1op | Mtmr12 |
| Kcnk1 | Fgfr4 | Mvd |
| Kcnk12 | Fip1l1 | Myd116 |
| Kctd10 | Fmr1 | Myh9 |
| Kctd20 | Fnbp4 | Nap1l1 |
| Kif12 | Fndc3a | Nars |
| Kif23 | Fnip1 | Nat13 |
| Klf2 | Fntb | Nat5 |
| Klk1b8 | Foxp4 | Nckap1l |
| Lage3 | Fto | Ncl |
| Larp5 | Ftsj2 | Ndufs3 |
| Ldhb | Fubp1 | Ndufs6 |
| Ldoc1l | Fus | Ndufv2 |
| Lmo2 | Fut8 | Nebl |
| Lonp1 | G3bp2 | Nek4 |
| Lphn1 | Gabrr2 | Nfe2l3 |
| Lrig1 | Ganab | Nhp2l1 |
| Lrp8 | Gas5 | Nme1 |
| Lrpprc | Gbe1 | Nmt1 |
| Lsm4 | Gbp3 | Nol10 |
| Lsp1 | Gcap14 | Nol12 |
| Ly6e | Gcn5l2 | Nola1 |
| Lztfl1 | Ggcx | Npepps |
| Lztr1 | Ghdc | Nphp3 |
| Maf1 | Git1 | Nrk |
| Man2b1 | Glod4 | Nsun2 |
| Map2k3 | Glud1 | Nub1 |
| Map2k7 | Glul | Nufip1 |
| Map3k1 | Gm711 | Numa1 |
| Map3k3 | Gnb1 | Nup62 |
| Map3k5 | Gng2 | Nxt1 |
| Mapkapk5 | Gnpat | Oas1a |
| March6 | Gnpda2 | Odc1 |
| Mast3 | Gnptab | Odz2 |
| Mcm3ap | Golga3 | Opa3 |
| Me2 | Golga5 | Orai2 |
| Mea1 | Gosr1 | Osgin2 |
| Med29 | Gpd1 | OTTMUSG00000009332 |
| Mfsd10 | Gpi1 | P4ha1 |
| Mfsd8 | Gpr108 | Pa2g4 |
| Mical3 | Gpsm1 | Pabpc4 |
| Mink1 | Gpsn2 | Pacsin1 |
| Mipep | Gpx1 | Pacsin2 |
| Mllt6 | Gtdc1 | Pak1ip1 |
| Mlph | Gtlf3b | Pan3 |
| Mrpl1 | Gtpbp5 | Parl |
| Mrps14 | Gulp1 | Parvg |
| Msh2 | Gys1 | Pdcd1lg2 |
| Mta1 | H2 | Pdcd4 |
| Mtap | H2afz | Pdlim5 |
| Myg1 | Hace1 | Per1 |
| Myo18a | Has3 | Pex1 |
| Napa | Hdac1 | Pex14 |
| Narg1 | Hdac3 | Pex2 |
| Nat2 | Hdac5 | Pex6 |
| Ncbp2 | Hdgfrp2 | Pfkp |
| Nckap1 | Heg1 | Pgam1 |
| Ncoa1 | Hgs | Pgk1 |
| Ncoa3 | Hiat1 | Pgk1 |
| Ndnl2 | Hibadh | Phf15 |
| Ndrg1 | Hibch | Pias3 |
| Ndufs1 | Hipk3 | Pim2 |
| Nedd9 | Hira | Pin1 |
| Nek1 | Hist1h1a | Pink1 |
| Nek6 | Hist1h1e | Pitpnm1 |
| Nfe2l1 | Hist1h2bc | Pkn1 |
| Nfkbie | Hist1h4k | Pldn |
| Nfrkb | Hlf | Plk3 |
| Nhp2l1 | Hltf | Pnpla7 |
| Nln | Hnrnpr | Polg |
| Nme2 | Hnrpm | Polr2a |
| Nnt | Hoxa7 | Polr2e |
| Nol9 | Hrh1 | Polr3d |
| Npas2 | Ier2 | Polr3h |
| Nploc4 | Ift122 | Pom121 |
| Nudt7 | Ift172 | Pon2 |
| Nudt9 | Igf2r | Ppa2 |
| Numa1 | Il16 | Ppcdc |
| Nup160 | Ilkap | Ppp2r1a |
| Nup37 | Immp1l | Ppp2r2a |
| Nup50 | Impa1 | Ppp3ca |
| Nup98 | Impact | Prdx3 |
| Ofd1 | Impdh1 | Prkce |
| Orai1 | Ing1 | Prmt3 |
| Orc3l | Ing3 | Pros1 |
| OTTMUSG00000010657 | Ipmk | Pscd2 |
| Otud5 | Iqsec2 | Pscd3 |
| Panx1 | Irak1 | Psenen |
| Papd1 | Isca2 | Psma6 |
| Papola | Itch | Psma7 |
| Park7 | Itfg3 | Psmb5 |
| Parp3 | Itga1 | Psmc3 |
| Paxip1 | Itga2b | Psmd2 |
| Pcm1 | Itgav | Ptbp1 |
| Pcna | Itk | Ptch1 |
| Pctk2 | Ivns1abp | Ptges3 |
| Pde7a | Iws1 | Ptk2 |
| Pdk2 | Jak2 | Ptrh1 |
| Pds5b | Jak3 | Rab1b |
| Pgs1 | Jarid1a | Rab37 |
| Pik3c2a | Jmjd1a | Rabepk |
| Pik3cd | Jmjd5 | Rad52 |
| Pim3 | Josd2 | Ralgds |
| Pisd | Kcnab2 | Ran |
| Pitpnb | Kctd9 | Rars |
| Piwil2 | Kdsr | Rasa1 |
| Pla2g1b | Kifc1 | Rbbp6 |
| Plcb4 | Kirrel3 | Rbbp7 |
| Plekhg2 | Klhl20 | Rbm22 |
| Plod3 | Kynu | Rbms1 |
| Pmpca | Laptm5 | Rbmx2 |
| Pnpla2 | Lass2 | Rbpj |
| Pnpla8 | Leprotl1 | Rbpms |
| Pold3 | Letmd1 | Rdbp |
| Polr3e | Lgals9 | Rdh11 |
| Ppfibp1 | Limd1 | Rdx |
| Ppid | Limd2 | Recql4 |
| Ppil3 | Limk1 | Relt |
| Ppm1d | Lmf2 | Rfwd2 |
| Ppp1r13b | Lnpep | Rfx3 |
| Ppp2r5c | Lnx1 | Rgl2 |
| Ppp5c | LOC435333 | Rgnef |
| Prkab1 | Lpgat1 | Rgs10 |
| Prkcbp1 | Lrp6 | Rgs3 |
| Prkci | Lrrc14 | Rhbdd1 |
| Prkcsh | Lrrc16a | Rin3 |
| Prmt2 | Lrrc49 | Riok2 |
| Prpf3 | Lrrc8d | Rnf170 |
| Prpf38a | Luc7l | Rpl24 |
| Prpf39 | Luc7l2 | Rps19bp1 |
| Psd | Lxn | Rps2 |
| Psd4 | Ly6a | Rps25 |
| Psma2 | Ly6k | Rps25 |
| Psmb3 | Lyrm5 | Rps9 |
| Psmb8 | M6pr | Rras |
| Psme4 | Mad1l1 | Rtel1 |
| Pten | Mak10 | Sap18 |
| Ptk2b | Maml3 | Sart3 |
| Ptplb | Man2a1 | Serpinb9b |
| Ptpmt1 | Manba | Serpine1 |
| Ptpro | Map2k1ip1 | Sestd1 |
| Pum1 | Map2k4 | Sfrs5 |
| Pum2 | Map2k5 | Sh3glb2 |
| Pus10 | Map3k12 | Si |
| Pycr2 | Map3k7ip2 | Siah1a |
| Qars | Map4k1 | Slc19a2 |
| Qsox2 | Map4k4 | Slc23a1 |
| Rab33b | Mapk1 | Slc25a15 |
| Rab8a | Mapk9 | Slc25a26 |
| Ralb | Mapkapk2 | Slc25a39 |
| Ralgps2 | March2 | Slc25a4 |
| Rangap1 | Mare | Slc30a4 |
| Rasa3 | Mbc2 | Slc36a1 |
| Rasal1 | Mbnl1 | Slc38a9 |
| Rassf2 | Mboat1 | Slc39a14 |
| Rbbp6 | mCG_10796 | Slc4a2 |
| Rbl2 | Mcts1 | Slc9a1 |
| Rbm14 | Mdm2 | Slc9a9 |
| Rbm16 | Med12 | Slfn4 |
| Rbm18 | Med13l | Smarca2 |
| Rcbtb2 | Med14 | Smpd4 |
| Rcn1 | Med21 | Snd1 |
| Rest | Med23 | Snrpd1 |
| Rfk | Med8 | Socs1 |
| Rfng | Mettl6 | Socs6 |
| Rhot1 | Mfap5 | Spcs3 |
| Rhot2 | Mga | Sppl3 |
| Riok1 | Mgat4b | Spred1 |
| Rmnd5b | Mib1 | Ss18l1 |
| Rnasen | Mier1 | St3gal4 |
| Rnf10 | Mkks | St7 |
| Rnf138 | Mkl2 | Stap1 |
| Rnf144a | Mkln1 | Stat3 |
| Rnf34 | Mkrn1 | Stk11ip |
| Rnmt | Mmab | Stra13 |
| Rnpc3 | Mobkl1b | Stra6 |
| Rorc | Mobkl3 | Strn4 |
| Rp2h | Mrp63 | Sufu |
| Rpl10a | Mrpl10 | Supt3h |
| Rpl3 | Mrpl33 | Tacc2 |
| Rpn2 | Mrpl49 | Tbce |
| Rpo1 | Mrps11 | Tcerg1 |
| Rps15 | Mrps24 | Tcf7 |
| Rps15a | Msra | Tcp1 |
| Rps2 | Mterfd2 | Tes |
| Rpsa | Mtm1 | Tgds |
| Rrad | Mtmr9 | Tgoln1 |
| Rraga | Mybpc3 | Timm13 |
| Rtcd1 | Myef2 | Timm23 |
| Rttn | Myl2 | Tiparp |
| Saal1 | Mysm1 | Tlr9 |
| Samd4b | N4bp2l2 | Tmbim4 |
| Saps1 | Naca | Tmco1 |
| Sars | Nagpa | Tmem106a |
| Sars | Nap1l4 | Tmem165 |
| Sash3 | Napepld | Tmem20 |
| Sbf1 | Nat11 | Tmem49 |
| Scamp1 | Ncoa5 | Tmem55a |
| Scarb1 | Ncor1 | Tmem59 |
| Scmh1 | Ncor1 | Tmem66 |
| Scrib | Ncor2 | Tmem93 |
| Scyl1 | Ndufa13 | Tnfrsf18 |
| Scyl1bp1 | Ndufs7 | Tnfrsf1b |
| Sdhd | Nedd4 | Tnnt3 |
| Sec16a | Nek7 | Tnpo1 |
| Sec61a1 | Net1 | Tob2 |
| Sec61g | Nf1 | Top2b |
| Senp2 | Nfix | Topbp1 |
| Senp7 | Nhlrc2 | Tpm1 |
| Serpinb1a | Nin | Trex1 |
| Serpinb1b | Nipa2 | Trib3 |
| Setd1a | Nipbl | Trp53 |
| Setdb1 | Nipsnap1 | Trp53bp2 |
| Sf3a1 | Nisch | Tsfm |
| Sf3a2 | Nkap | Ttll4 |
| Sf3b3 | Nmral1 | Tuba |
| Sfi1 | Nono | Tubb5 |
| Sfrs15 | Nrp1 | Txk |
| Sgpl1 | Nsf | Txlna |
| Sgsm2 | Nsun6 | Txnrd1 |
| Sidt2 | Numb | Ube2e3 |
| Sirt1 | Nup153 | Ube2z |
| Slc13a1 | Nup210 | Ube4a |
| Slc15a2 | Nup35 | Ubtf |
| Slc16a6 | Ociad1 | Uchl5 |
| Slc25a11 | Ogfod1 | Uimc1 |
| Slc25a23 | Ogg1 | Ulk1 |
| Slc25a44 | Opa1 | Ulk2 |
| Slc25a46 | ORF19 | Unkl |
| Slc30a9 | ORF34 | Usmg5 |
| Slc31a2 | Osbpl3 | Usp12 |
| Slc36a4 | Osgep | Usp52 |
| Slc38a10 | Ostm1 | Uxt |
| Slc38a8 | Otog | Vamp1 |
| Slc39a6 | OTTMUSG00000010173 | Vegfa |
| Slc5a6 | OTTMUSG00000010173 | Vprbp |
| Slc7a6os | Pabpn1 | Vps29 |
| Slit2 | Paf1 | Vrk2 |
| Smad7 | Pafah1b1 | Wdr35 |
| Smarca5 | Pak2 | Wdr40a |
| Smarcd2 | Pard3 | Wdr46 |
| Smu1 | Parg | Wdr51a |
| Snf8 | Parn | Wdsof1 |
| Snx10 | Patz1 | Xbp1 |
| Snx13 | Pccb | Xiap |
| Snx25 | Pcdha10 | Xrn2 |
| Snx4 | Pcyt2 | Ythdf3 |
| Snx5 | Pdcd2l | Zc3h12d |
| Sod1 | Pex12 | Zc3h18 |
| Spcs2 | Pex7 | Zc3hav1 |
| Sqstm1 | Phc3 | Zc3hc1 |
| Srebf2 | Phf14 | Zfp212 |
| Ssbp3 | Phf7 | Zfp27 |
| Ssr1 | Phkb | Zfp335 |
| St3gal1 | Phtf1 | Zfp422 |
| St3gal5 | Picalm | Zfp472 |
| Stag1 | Pigk | Zfp62 |
| Stambp | Pigl | Zfp655 |
| Stat2 | Pik3c3 | Zfp677 |
| Stim1 | Pip5k3 | Zfyve16 |
| Stt3a | Pknox1 | Zmat5 |
| Stt3b | Plaur | Znhit3 |
| Stx4a | Plcb1 | Zrsr2 |
| Suclg2 | Plekha7 | Zswim4 |
| Suds3 | Plk1 | Zwint |
| Suhw4 | Plxnc1 | Zyx |
| Supt16h | Pml |  |
| Sypl | Pnkp |  |
| Sys1 | Pnp1 |  |
| Syvn1 | Pnpla6 |  |
| Tada2l | Pole4 |  |
| Taf6 | Polr3c |  |
| Taf6l | Pomt1 |  |
| Tbc1d8 | Ppp1cc |  |
| Tbl1xr1 | Ppp3cb |  |
| Tbrg4 | Prdm1 |  |
| Tcea1 | Prdm16 |  |
| Tcn2 | Preb |  |
| Tcof1 | Prkar1a |  |
| Tex2 | Prkcz |  |
| Tex264 | Prkdc |  |
| Tfpi | Prnpip1 |  |
| Thoc1 | Prom2 |  |
| Thoc6 | Prpf4b |  |
| Timeless | Prpf6 |  |
| Tm9sf4 | Prpsap1 |  |
| Tmc2 | Prr13 |  |
| Tmed2 | Prune |  |
| Tmem101 | Psme2 |  |
| Tmem127 | Pstpip2 |  |
| Tmem134 | Ptdss1 |  |
| Tmem149 | Ptp4a3 |  |
| Tmem16f | Ptpn1 |  |
| Tmem186 | Ptpn11 |  |
| Tmem194 | Ptpn14 |  |
| Tmod3 | Pygb |  |
| Tnfrsf13c | Pygm |  |
| Tnfsf11 | R3hdm2 |  |
| Tnfsf8 | Rab21 |  |
| Tnks | Rab22a |  |
| Tnpo2 | Rab26 |  |
| Tnrc6b | Rab2a |  |
| Top1 | Rab3ip |  |
| Tox | Rab43 |  |
| Trabd | Rab5c |  |
| Traf3ip3 | Rabgef1 |  |
| Trappc4 | Rac1 |  |
| Trib1 | Rad17 |  |
| Trim25 | Rad51l3 |  |
| Trim32 | Raf1 |  |
| Trim56 | Raly |  |
| Trip12 | Ranbp2 |  |
| Trmt6 | Ranbp6 |  |
| Trp53inp1 | Rap1b |  |
| Trpc4ap | Rasa2 |  |
| Trrap | Rbak |  |
| Tsc1 | Rbck1 |  |
| Tsnax | Rbl1 |  |
| Tspan31 | Rbm25 |  |
| Ttyh1 | Rbm3 |  |
| Tuba1a | Rbm3 |  |
| Tuba1b | Rbm4 |  |
| Tubb2c | Rbm4b |  |
| Tuft1 | Rbm6 |  |
| Txndc11 | Reep5 |  |
| Txndc4 | Rer1 |  |
| Tyms | Rffl |  |
| Ubap2 | Rfx1 |  |
| Ube2r2 | Rhbdd2 |  |
| Ube2t | Rhbdf1 |  |
| Ubqln4 | Rhobtb2 |  |
| Uck1 | Rhof |  |
| Ugdh | Ric8 |  |
| Uqcrh | Ric8b |  |
| Usp28 | Rif1 |  |
| Usp46 | Rint1 |  |
| Usp8 | Riok3 |  |
| Utp6 | Ripk1 |  |
| Vac14 | Rnf139 |  |
| Vamp7 | Rnf185 |  |
| Vps33b | Rock2 |  |
| Vps37b | Rora |  |
| Wapal | Rpe |  |
| Wasf2 | Rpl10 |  |
| Wbscr16 | Rpl13 |  |
| Wdr12 | Rpl15 |  |
| Wdr26 | Rpl18 |  |
| Wdr3 | Rpl18a |  |
| Wdr68 | Rpl35 |  |
| Wdr78 | Rpl36al |  |
| Wdr79 | Rpl37 |  |
| Wsb1 | Rplp1 |  |
| Xpnpep1 | Rps10 |  |
| Xpo1 | Rps12 |  |
| Ybx1 | Rps18 |  |
| Ythdf2 | Rps2 |  |
| Zbtb1 | Rps25 |  |
| Zc3h14 | Rps6 |  |
| Zc3h6 | Rps7 |  |
| Zcchc9 | Rpsa |  |
| Zcrb1 | Rtn4 |  |
| Zcwpw1 | Rwdd3 |  |
| Zdhhc21 | Rxra |  |
| Zdhhc6 | Ryr2 |  |
| Zfp182 | Saps2 |  |
| Zfp189 | Saps3 |  |
| Zfp386 | Sart1 |  |
| Zfp426 | Sbno2 |  |
| Zfp457 | Scarb2 |  |
| Zfp51 | Scly |  |
| Zfp513 | Scnn1g |  |
| Zfp623 | Scyl2 |  |
| Zfp629 | Sdcbp |  |
| Zfp650 | Sdccag3 |  |
| Zfp654 | Sdf2 |  |
| Zfp668 | Sdha |  |
| Zfp809 | Sec11a |  |
| Zmynd11 | Sec11c |  |
| Znrf1 | Sec14l4 |  |
|  | Sec24a |  |
|  | Sec63 |  |
|  | Selenbp1 |  |
|  | Sema4d |  |
|  | Sep15 |  |
|  | Sephs2 |  |
|  | Serinc3 |  |
|  | Serpinb6c |  |
|  | Setd3 |  |
|  | Setd5 |  |
|  | Sfpq |  |
|  | Sfrs3 |  |
|  | Sfrs8 |  |
|  | Sgk2 |  |
|  | Sh3glb1 |  |
|  | Siae |  |
|  | Sip1 |  |
|  | Slain2 |  |
|  | Slc10a7 |  |
|  | Slc17a5 |  |
|  | Slc26a2 |  |
|  | Slc26a8 |  |
|  | Slc35c2 |  |
|  | Slc35d1 |  |
|  | Slc35e4 |  |
|  | Slc37a1 |  |
|  | Slk |  |
|  | Sltm |  |
|  | Smad1 |  |
|  | Smap2 |  |
|  | Smarcal1 |  |
|  | Smarcc2 |  |
|  | Smc1a |  |
|  | Smc5 |  |
|  | Smg6 |  |
|  | Smg7 |  |
|  | Snapc3 |  |
|  | Snx11 |  |
|  | Snx3 |  |
|  | Solh |  |
|  | Son |  |
|  | Sox13 |  |
|  | Sox6 |  |
|  | Sp3 |  |
|  | Spaca3 |  |
|  | Spns1 |  |
|  | Srgn |  |
|  | Srpk1 |  |
|  | Srr |  |
|  | Srrm1 |  |
|  | Srrm2 |  |
|  | Srxn1 |  |
|  | Ssfa2 |  |
|  | Ssh3 |  |
|  | St6galnac6 |  |
|  | Stambpl1 |  |
|  | Stat5b |  |
|  | Stat6 |  |
|  | Stk24 |  |
|  | Stk35 |  |
|  | Stx7 |  |
|  | Supt5h |  |
|  | Tbc1d12 |  |
|  | Tbc1d13 |  |
|  | Tbc1d14 |  |
|  | Tbc1d22b |  |
|  | Tbpl1 |  |
|  | Tbrg1 |  |
|  | Tcfe2a |  |
|  | Tcfeb |  |
|  | Tera |  |
|  | Terf2ip |  |
|  | Tia1 |  |
|  | Tial1 |  |
|  | Tinf2 |  |
|  | Tlk2 |  |
|  | Tln2 |  |
|  | Tmc3 |  |
|  | Tmem11 |  |
|  | Tmem128 |  |
|  | Tmem14c |  |
|  | Tmem173 |  |
|  | Tmem175 |  |
|  | Tmem179b |  |
|  | Tmem2 |  |
|  | Tmem60 |  |
|  | Tmem77 |  |
|  | Tmem8 |  |
|  | Tmem87b |  |
|  | Tnfaip3 |  |
|  | Tnfrsf13b |  |
|  | Tnfrsf23 |  |
|  | Tnfrsf25 |  |
|  | Tob1 |  |
|  | Tom1 |  |
|  | Topors |  |
|  | Tpp2 |  |
|  | Tpt1 |  |
|  | Trak1 |  |
|  | Trak2 |  |
|  | Trappc6b |  |
|  | Trex1 |  |
|  | Trim62 |  |
|  | Trp53bp1 |  |
|  | Trpc2 |  |
|  | Trpm6 |  |
|  | Tsc2 |  |
|  | Tsc22d1 |  |
|  | Tsc22d4 |  |
|  | Ttc1 |  |
|  | Ttc13 |  |
|  | Ttc5 |  |
|  | Ttll12 |  |
|  | Ttll5 |  |
|  | Tubb2b |  |
|  | Tubg2 |  |
|  | Twistnb |  |
|  | Txnl4b |  |
|  | Tyk2 |  |
|  | Tyw1 |  |
|  | Uba1 |  |
|  | Ubash3a |  |
|  | Ubb |  |
|  | Ube2d1 |  |
|  | Ube2f |  |
|  | Ube2i |  |
|  | Ube2j2 |  |
|  | Ube2q1 |  |
|  | Ube3a |  |
|  | Ubl7 |  |
|  | Ublcp1 |  |
|  | Ubqln1 |  |
|  | Ubr1 |  |
|  | Ubxd1 |  |
|  | Ubxd5 |  |
|  | Ubxd6 |  |
|  | Ubxd8 |  |
|  | Uevld |  |
|  | Uhrf2 |  |
|  | Unc119b |  |
|  | Upf1 |  |
|  | Urm1 |  |
|  | Urm1 |  |
|  | Usp16 |  |
|  | Usp25 |  |
|  | Usp37 |  |
|  | Usp45 |  |
|  | Usp48 |  |
|  | Usp7 |  |
|  | Utx |  |
|  | Uvrag |  |
|  | Vcl |  |
|  | Vegfb |  |
|  | Vezf1 |  |
|  | Vps28 |  |
|  | Vps41 |  |
|  | Vps8 |  |
|  | Vti1a |  |
|  | Wbp7 |  |
|  | Wdr31 |  |
|  | Wdr32 |  |
|  | Wdr45l |  |
|  | Wipf1 |  |
|  | Wipi2 |  |
|  | Xlr |  |
|  | Xpo7 |  |
|  | Yipf2 |  |
|  | Ykt6 |  |
|  | Zbtb25 |  |
|  | Zc3hav1l |  |
|  | Zdhhc1 |  |
|  | Zdhhc16 |  |
|  | Zdhhc18 |  |
|  | Zdhhc3 |  |
|  | Zfa |  |
|  | Zfand2a |  |
|  | Zfand6 |  |
|  | Zfp110 |  |
|  | Zfp142 |  |
|  | Zfp148 |  |
|  | Zfp207 |  |
|  | Zfp273 |  |
|  | Zfp282 |  |
|  | Zfp295 |  |
|  | Zfp313 |  |
|  | Zfp384 |  |
|  | Zfp395 |  |
|  | Zfp40 |  |
|  | Zfp410 |  |
|  | Zfp592 |  |
|  | Zfp61 |  |
|  | Zfp639 |  |
|  | Zfp740 |  |
|  | Zfp758 |  |
|  | Zfr |  |
|  | Zfyve26 |  |
|  | Zfyve27 |  |
|  | Zmat2 |  |
|  | Znrf2 |  |
|  | Zranb1 |  |
|  | Zswim6 |  |
|  | Zufsp |  |
|  | Zw10 |  |
