# Supplementary material for: CD28 Costimulation Regulates Genome-Wide Effects on Alternative Splicing
Source: PLoS One. 2012 Jun 29;7(6):e40032. doi: 10.1371/journal.pone.0040032 (PMC3386953; doi:10.1371/journal.pone.0040032)
Supplement: Table S5 — Unique Biological processes in transcripts differentially expressed or spliced between naïve T cells and TCR activated T cells, naïve T cells and TCR/CD28-activated T cells. (DOC) [file pone.0040032.s008.doc]

**Table S5**

Unique Biological processes in transcripts differentially expressed between naïve T cells and TCR-activated T cells

| **Gene Ontology Biological Process** | **GO ID** | **p-value of enrichment compared to whole genome** | **Gene symbols** |
| --- | --- | --- | --- |
| lymphocyte proliferation | GO:0046651 | 0.005 | Itgad Cxcr4 Il7r Impdh2 Hells P2rx7 Fkbp1a Itgam Cd40lg Slc11a1 |
| leukocyte proliferation | GO:0070661 | 0.006 | Itgad Cxcr4 Il7r Impdh2 Hells P2rx7 Fkbp1a Itgam Cd40lg Slc11a1 |
| mononuclear cell proliferation | GO:0032943 | 0.006 | Itgad Cxcr4 Il7r Impdh2 Hells P2rx7 Fkbp1a Itgam Cd40lg Slc11a1 |
| positive regulation of immune effector process | GO:0002699 | 0.015 | Fcgr1 P2rx7 Fcer1g Cd1d1 Lbp Cadm1 Sh2d1a Lag3 Fcgr3 |
| regulation of inflammatory response | GO:0050727 | 0.021 | Fcgr1 ENSMUSG00000075466 Il2 Fcer1g Aoah Lbp Tgm2 Il2ra Fcgr3 Adrb2 |
| positive regulation of transport | GO:0051050 | 0.022 | Tnfsf4 Il2 P2rx7 Fcer1g Ifng Tnf Lta Fcgr1 Sirpa Scamp5 Cd209b Slc11a1 Tnfrsf4 Sgk1 Fcgr3 Nlrp3 |
| positive regulation of lymphocyte mediated immunity | GO:0002708 | 0.025 | Fcgr1 P2rx7 Fcer1g Cd1d1 Cadm1 Sh2d1a Lag3 Fcgr3 |
| positive regulation of leukocyte mediated immunity | GO:0002705 | 0.025 | Fcgr1 P2rx7 Fcer1g Cd1d1 Cadm1 Sh2d1a Lag3 Fcgr3 |
| regulation of cell killing | GO:0031341 | 0.038 | Il7r P2rx7 Cd1d1 Cadm1 Sh2d1a Sh2d1b1 Lag3 |
| regulation of leukocyte mediated cytotoxicity | GO:0001910 | 0.038 | Il7r P2rx7 Cd1d1 Cadm1 Sh2d1a Sh2d1b1 Lag3 |
| positive regulation of inflammatory response | GO:0050729 | 0.048 | Fcgr1 ENSMUSG00000075466 Fcer1g Lbp Tgm2 Fcgr3 |
| positive regulation of adaptive immune response | GO:0002821 | 0.052 | Fcgr1 P2rx7 Fcer1g Cd1d1 Tnfsf12 Slc11a1 Fcgr3 |
| positive regulation of adaptive immune response based on somatic recombination of immune receptors built from immunoglobulin superfamily domains | GO:0002824 | 0.052 | Fcgr1 P2rx7 Fcer1g Cd1d1 Tnfsf12 Slc11a1 Fcgr3 |
| defense response to bacterium | GO:0042742 | 0.052 | Lyz1 P2rx7 Fcer1g Lyz1 Hck Ifng Tnf Fcgr1 Nod1 Lbp Fgr Slc11a1 Myo1f Irf8 |
| humoral immune response | GO:0006959 | 0.052 | Exo1 Ccr2 C1qa C1qc C1qb Igj Sh2d1a Tnf Lta |
| cytokine production | GO:0001816 | 0.059 | Edg3 Irf7 Nod1 Lbp Il18 Il12rb2 Slc11a1 Nlrp3 |
| regulation of isotype switching | GO:0045191 | 0.06 | Il2 Tbx21 Tnfsf12 Ifng Cd40 |
| response to bacterium | GO:0009617 | 0.064 | Lyz1 Fcer1g P2rx7 Lyz1 Hck Tlr11 Ifng Tnf Fcgr1 Nod1 Lbp Il12rb2 Fgr Myo1f Cd209b Slc11a1 Irf8 |
| regulation of secretion | GO:0051046 | 0.069 | Tnfsf4 Il2 P2rx7 Fcer1g Cd40lg Lif Ifng Tnf Rapgef4 Cd40 Rapgef4 Hmox1 Scamp5 Tnfrsf4 Nlrp3 |
| unsaturated fatty acid metabolic process | GO:0033559 | 0.08 | Ptgds2 Ptgs1 Hpgd Ggt5 Tbxas1 Cyp2d22 Alox5ap |
| positive regulation of response to external stimulus | GO:0032103 | 0.09 | Fcgr1 ENSMUSG00000075466 S1pr1 Fcer1g Lbp Tgm2 Fcgr3 |
| regulation of response to external stimulus | GO:0032101 | 0.09 | Fcgr1 ENSMUSG00000075466 Il2 S1pr1 Fcer1g Anxa2 Aoah Lbp Tgm2 Il2ra Fcgr3 Adrb2 |
| positive regulation of B cell activation | GO:0050871 | 0.1 | Cdkn1a Il2 Tbx21 Tnfsf12 Tnfrsf4 Ifng Cd40 |
| T cell proliferation | GO:0042098 | 0.118 | Itgad Cxcr4 P2rx7 Fkbp1a Itgam Slc11a1 |
| positive regulation of secretion | GO:0051047 | 0.124 | Tnfsf4 Il2 P2rx7 Fcer1g Scamp5 Tnfrsf4 Ifng Nlrp3 |
| multicellular organismal homeostasis | GO:0048871 | 0.143 | Kdr Acp5 P2rx7 Epas1 Cd7 Slc11a1 Adrb1 Adrb2 Hfe |
| regulation of interferon-gamma production | GO:0032649 | 0.145 | Cd1d1 Hspd1 Il18 Tlr8 Tlr3 Slc11a1 |
| positive regulation of cell killing | GO:0031343 | 0.169 | P2rx7 Cd1d1 Cadm1 Sh2d1a Lag3 |
| positive regulation of leukocyte mediated cytotoxicity | GO:0001912 | 0.169 | P2rx7 Cd1d1 Cadm1 Sh2d1a Lag3 |
| positive regulation of endocytosis | GO:0045807 | 0.184 | Fcgr1 Sirpa Fcer1g Slc11a1 Cd209b Fcgr3 |
| regulation of transcription factor activity | GO:0051090 | 0.189 | Tnfsf4 Hmox1 Tnfrsf4 Tlr3 Tnf Tgfbr3 Nfam1 Nlrp3 |
| cellular amino acid derivative biosynthetic process | GO:0042398 | 0.191 | Gatm Odc1 Chdh Ggt5 Srm Amd1 Nr4a2 |
| sphingolipid metabolic process | GO:0006665 | 0.223 | Kit Lypla3 Smpdl3a P2rx7 B4galt6 St6galnac4 Gm2a Crem |
| regulation of DNA recombination | GO:0000018 | 0.229 | Il2 Tbx21 Tnfsf12 Ifng Cd40 |
| induction of apoptosis by extracellular signals | GO:0008624 | 0.229 | Casp3 Il18 Fasl Tnf Gzmb |
| elevation of cytosolic calcium ion concentration | GO:0007204 | 0.231 | AI605517 Xcl1 S1pr1 P2rx7 Fkbp1a Tgm2 |
| antigen processing and presentation of peptide antigen | GO:0048002 | 0.231 | Fcgr1 Ctse Fcer1g Slc11a1 Fcgr3 Hfe |
| isoprenoid metabolic process | GO:0006720 | 0.231 | Idi2 Fdps Fdps Crabp2 Fdft1 Hmgcs1 Hmgcr Idi1 |
| regulation of cell migration | GO:0030334 | 0.23 | Cxcr4 Hif1a Ccr2 S1pr1 Abi3 Clic4 Itgb3 Cxcl10 Myo1f Tgfbr3 |
| biogenic amine metabolic process | GO:0006576 | 0.239 | Fabp5 Fabp5 Odc1 Epas1 Chdh Capns2 Cyp2d22 Srm Amd1 Nr4a2 |
| regulation of peptidyl-tyrosine phosphorylation | GO:0050730 | 0.243 | ENSMUSG00000075466 Il2 Il3 Sh2d1b1 Lif Ifng Il22 |
| membrane lipid metabolic process | GO:0006643 | 0.244 | Kit Lypla3 Smpdl3a P2rx7 B4galt6 St6galnac4 Gm2a Crem |
| antigen processing and presentation | GO:0019882 | 0.261 | Fcgr1 Ctse Fcer1g Cd1d1 Fcgrt Slc11a1 Ifng Fcgr3 Hfe |
| peptide metabolic process | GO:0006518 | 0.263 | Thop1 Mgst1 Ggt5 Ide Txnrd3 Ace3 |
| cytosolic calcium ion homeostasis | GO:0051480 | 0.263 | AI605517 Xcl1 S1pr1 P2rx7 Fkbp1a Tgm2 |
| icosanoid biosynthetic process | GO:0046456 | 0.268 | Ptgds2 Ptgs1 Ggt5 Tbxas1 Alox5ap |
| regulation of vesicle-mediated transport | GO:0060627 | 0.27 | Rapgef4 Fcgr1 Sirpa Fcer1g Hmox1 Scamp5 Slc11a1 Cd209b Fcgr3 Rapgef4 |
| positive regulation of protein amino acid phosphorylation | GO:0001934 | 0.287 | ENSMUSG00000075466 Il2 P2rx7 Il3 Sh2d1b1 Lif Ifng |
| biogenic amine biosynthetic process | GO:0042401 | 0.293 | Odc1 Chdh Srm Amd1 Nr4a2 |
| unsaturated fatty acid biosynthetic process | GO:0006636 | 0.293 | Ptgds2 Ptgs1 Ggt5 Tbxas1 Alox5ap |
| antigen processing and presentation of exogenous antigen | GO:0019884 | 0.346 | Fcgr1 Ctse Fcer1g Cd1d1 Fcgr3 |
| positive regulation of DNA metabolic process | GO:0051054 | 0.346 | Il2 Tbx21 Tnfsf12 Ifng Cd40 |
| positive regulation of phosphorylation | GO:0042327 | 0.356 | ENSMUSG00000075466 Il2 P2rx7 Il3 Sh2d1b1 Lif Ifng |
| DNA damage response, signal transduction | GO:0042770 | 0.356 | Mif Ccnd1 Hmox1 Tipin Brca1 Cdc2a Chek1 |
| negative regulation of defense response | GO:0031348 | 0.37 | Il2 Aoah Sh2d1b1 Il2ra Adrb2 |
| immunoglobulin production | GO:0002377 | 0.37 | Exo1 Il7r Cd40lg Tnfsf12 Ung |
| positive regulation of phosphate metabolic process | GO:0045937 | 0.391 | ENSMUSG00000075466 Il2 P2rx7 Il3 Sh2d1b1 Lif Ifng |
| positive regulation of phosphorus metabolic process | GO:0010562 | 0.391 | ENSMUSG00000075466 Il2 P2rx7 Il3 Sh2d1b1 Lif Ifng |
| regulation of B cell proliferation | GO:0030888 | 0.394 | Cdkn1a Il2 Casp3 Tnfrsf4 Cd40 |
| integrin-mediated signaling pathway | GO:0007229 | 0.399 | Itgad Itga2 Vav3 Itga9 Itgam Adam23 Itgb3 Itgb5 Adam23 |

Unique Biological processes in transcripts differentially expressed between naïve T cells and TCR/CD28 activated T cells

| **Gene Ontology Biological Process** | **GO ID** | **p-value of enrichment compared to whole genome** | **Gene symbols** |
| --- | --- | --- | --- |
| tRNA processing | GO:0008033 | < 0.001 | Pop1 Mettl1 Pus7l Trmt5 Wdr4 Pus10 Elac2 Pus1 Aars Pop4 Rpp38 Pop7 Rpp30 Qtrtd1 Trmt6 Rpp14 Adat2 Rpp25 Dus4l Rpp40 Nsun2 Tsen2 Trnt1 6720458F09Rik 1110008L16Rik |
| translational initiation | GO:0006413 | 0.015 | Eif2b5 Eif1ad Eif1ay Eif1ay Trmt6 Eif3eip Eif3c Eif3d Eif3i Eif3g Eif4e Eif2b1 Eif4e AA589522 Eif2s2 Eif3b |
| mitochondrion organization | GO:0007005 | 0.018 | Tmem70 Epas1 Dna2 Pim2 BB166591 1700020C11Rik Sod2 Poldip2 Gfm1 Timm9 Timm8b Msto1 Mrpl17 Aifm1 P2rx7 Tomm20 Casp3 Timm50 Timm8a1 Msto1 Myc Mrpl15 Sept4 Mtx2 Mtx1 Timm8a1 Timm10 |
| regulation of DNA replication | GO:0006275 | 0.034 | Hus1 Cdt1 Gmnn Dna2 Pcna Pcna Tipin Clspn Brca2 Gtpbp4 |
| negative regulation of DNA replication | GO:0008156 | 0.041 | Hus1 Cdt1 Gmnn Tipin Clspn Brca2 Gtpbp4 |
| ribonucleoprotein complex assembly | GO:0022618 | 0.042 | Prpf31 Nip7 Prmt7 Cugbp2 Tsr1 Prmt5 Eif3d Smn1 Gemin6 Lsm2 BC029722 Eif2a |
| ribonucleoside monophosphate metabolic process | GO:0009161 | 0.046 | Paics Impdh2 Atic Impdh2 Ampd3 Adss Nt5e Gart Ampd2 Prps1 |
| purine ribonucleoside monophosphate metabolic process | GO:0009167 | 0.046 | Paics Impdh2 Atic Impdh2 Ampd3 Adss Nt5e Gart Ampd2 |
| purine nucleoside monophosphate metabolic process | GO:0009126 | 0.046 | Paics Impdh2 Atic Impdh2 Ampd3 Adss Nt5e Gart Ampd2 |
| negative regulation of DNA metabolic process | GO:0051053 | 0.06 | Rad18 Hus1 Blm Cdt1 Gmnn Tipin Clspn Brca2 Gtpbp4 |
| DNA duplex unwinding | GO:0032508 | 0.061 | Top1mt Supv3l1 Rad51 Mcm7 Mcm4 Mcm6 Mcm2 |
| ribonucleoside monophosphate biosynthetic process | GO:0009156 | 0.086 | Paics Impdh2 Atic Impdh2 Ampd3 Adss Gart Ampd2 Prps1 |
| DNA geometric change | GO:0032392 | 0.088 | Top1mt Supv3l1 Rad51 Mcm7 Mcm4 Mcm6 Mcm2 |
| purine nucleoside monophosphate biosynthetic process | GO:0009127 | 0.088 | Paics Impdh2 Atic Impdh2 Ampd3 Adss Gart Ampd2 |
| purine ribonucleoside monophosphate biosynthetic process | GO:0009168 | 0.088 | Paics Impdh2 Atic Impdh2 Ampd3 Adss Gart Ampd2 |
| RNA modification | GO:0009451 | 0.104 | Pus7 Mettl1 Qtrtd1 Nola2 Pus7l Dnajb11 Wdr4 Dimt1 Apobec1 6720458F09Rik Ftsj3 Pus1 Aars |
| positive regulation of tumor necrosis factor production | GO:0032760 | 0.121 | Clec7a Nod1 Fcer1g Cd14 Lbp Tlr4 Tlr3 |
| L-serine metabolic process | GO:0006563 | 0.156 | Psat1 Phgdh Shmt2 Shmt1 Psph |
| positive regulation of interleukin-6 biosynthetic process | GO:0045410 | 0.156 | Tlr6 Tlr1 Tlr4 Ifng Il1b |
| regulation of type I interferon production | GO:0032479 | 0.158 | Ddx58 Hspd1 Tlr8 Tlr4 Tlr3 Polr3g Polr3d |
| DNA unwinding during replication | GO:0006268 | 0.169 | Top1mt Rad51 Mcm7 Mcm4 Mcm6 Mcm2 |
| negative regulation of MAP kinase activity | GO:0043407 | 0.181 | ENSMUSG00000075466 Spred1 Pdcd4 Spry1 Hmgcr Nup62 Il1b Irak3 |
| coenzyme biosynthetic process | GO:0009108 | 0.197 | Kmo Haao Pdss1 Nampt Dlat Mthfd1l Acss2 Acss1 Gphn 2610209A20Rik Ggt5 Coq4 Coq7 Mthfd1 Mthfd2 Fpgs |
| phagocytosis, recognition | GO:0006910 | 0.221 | Fcgr1 Sirpa Clec7a Scarb1 Cd209b Fcgr3 |
| serine family amino acid biosynthetic process | GO:0009070 | 0.222 | Psat1 Dhfr Phgdh Cth Psph |
| interphase of mitotic cell cycle | GO:0051329 | 0.246 | Tfdp1 Lats2 E2f6 Dnajc2 Rbbp8 Mtbp Anp32b Cdca5 Chek1 App Pola1 Slfn1 Ccnd1 |
| cellular response to oxidative stress | GO:0034599 | 0.252 | Prdx1 Sod2 Hif1a Epas1 Pycr1 Txnrd1 2010100O12Rik Prdx3 |
| interphase | GO:0051325 | 0.3 | Tfdp1 Lats2 E2f6 Dnajc2 Rbbp8 Mtbp Anp32b Cdca5 Chek1 App Pola1 Slfn1 Ccnd1 |
| positive regulation of activated T cell proliferation | GO:0042104 | 0.298 | Icosl Il2 Il12b Il18 Il2ra |
| prostanoid metabolic process | GO:0006692 | 0.34 | Ptges2 Ptges3 Ptgds2 Ptgs1 Hpgd Tbxas1 |
| prostaglandin metabolic process | GO:0006693 | 0.34 | Ptges2 Ptges3 Ptgds2 Ptgs1 Hpgd Tbxas1 |
| regulation of interferon-beta production | GO:0032648 | 0.34 | Ddx58 Tlr8 Tlr4 Tlr3 Polr3g Polr3d |
| L-amino acid transport | GO:0015807 | 0.345 | Slc7a5 Slc36a1 Slc7a2 Slc25a4 Slc43a2 Slc7a8 Slc11a1 |
| nucleoside metabolic process | GO:0009116 | 0.356 | Umps Qtrtd1 Hmgcr Nme6 Ppat Mtap Prps1 A430108C13Rik Nme1 Nme2 Pycrl Nme2 Nt5e Dut |
| cofactor biosynthetic process | GO:0051188 | 0.364 | Alad Kmo Haao Pdss1 Nampt Dlat Mthfd1l Acss2 Acss1 Gphn Hmbs Nubp1 2610209A20Rik Ggt5 Coq4 Coq7 Mthfd1 Mthfd2 Fpgs |
| prostanoid biosynthetic process | GO:0046457 | 0.384 | Ptges2 Ptges3 Ptgds2 Ptgs1 Tbxas1 |
| prostaglandin biosynthetic process | GO:0001516 | 0.384 | Ptges2 Ptges3 Ptgds2 Ptgs1 Tbxas1 |
| positive regulation of cytokine secretion | GO:0050715 | 0.384 | P2rx7 Scamp5 Glmn Ifng Nlrp3 |
| macrophage activation | GO:0042116 | 0.384 | Slc7a2 Lbp Tlr1 Tlr4 Slc11a1 |
| protein amino acid methylation | GO:0006479 | 0.383 | Etf1 Suv39h1 Carm1 Prmt7 Hemk1 Prmt1 Ezh2 Prmt5 Gspt1 Btg2 Suv39h2 |
| protein amino acid alkylation | GO:0008213 | 0.383 | Etf1 Suv39h1 Carm1 Prmt7 Hemk1 Prmt1 Ezh2 Prmt5 Gspt1 Btg2 Suv39h2 |
| regulation of interleukin-12 production | GO:0032655 | 0.403 | Ltb Tlr6 Il10 Ifng Irak3 Cd40 |
| immunoglobulin production during immune response | GO:0002381 | 0.403 | Exo1 Icosl Ercc1 Tnfsf12 Nbn Swap70 |
| positive regulation of translation | GO:0045727 | 0.403 | Cdk4 Niban Eif2b5 Eif5a Tnf Ptms |
| double-strand break repair | GO:0006302 | 0.416 | Hus1 Sod2 Pola1 Xrcc6 Xrcc5 Ercc1 Rad54l Rad51 Xrcc2 Nbn Brca2 |
| heterocycle biosynthetic process | GO:0018130 | 0.453 | Alad Pycrl Gphn Hmbs Pycr1 2610209A20Rik Mthfd1l Aldh18a1 Mthfd1 Pycr2 A430108C13Rik Qdpr |
| protein localization in mitochondrion | GO:0070585 | 0.455 | Tomm20 Mtx2 Timm9 Mtx1 Timm8a1 Timm8a1 Timm8b Timm10 |
| protein targeting to mitochondrion | GO:0006626 | 0.455 | Tomm20 Mtx2 Timm9 Mtx1 Timm8a1 Timm8a1 Timm8b Timm10 |
| detection of biotic stimulus | GO:0009595 | 0.463 | Clec7a Nod1 Tlr1 Tlr4 Cd209b |
| negative regulation of glucose transport | GO:0010829 | 0.463 | Ltb ENSMUSG00000075466 Tnf Lta Il1b |
| peptidyl-arginine modification | GO:0018195 | 0.463 | Prmt7 Prmt1 Padi2 Prmt5 Art2b |
| positive regulation of stress-activated protein kinase signaling pathway | GO:0070304 | 0.467 | Nod1 Il3 Tlr4 Tlr3 Tnf Il1b |
| positive regulation of JNK cascade | GO:0046330 | 0.467 | Nod1 Il3 Tlr4 Tlr3 Tnf Il1b |
| regulation of cyclin-dependent protein kinase activity | GO:0000079 | 0.467 | Cdkn1a Lats2 Cdkn1b Casp3 Bccip Gtpbp4 |
| tRNA modification | GO:0006400 | 0.467 | Mettl1 6720458F09Rik Qtrtd1 Pus1 Aars Wdr4 |
| negative regulation of leukocyte activation | GO:0002695 | 0.485 | Cd274 Hmgb3 Il2 Pag1 Casp3 Hmox1 Il10 Glmn Il2ra Flt3 Lag3 Nfkbid |
| negative regulation of cell activation | GO:0050866 | 0.485 | Cd274 Hmgb3 Il2 Pag1 Casp3 Hmox1 Il10 Glmn Il2ra Flt3 Lag3 Nfkbid |
| meiotic cell cycle | GO:0051321 | 0.491 | Exo1 Mnd1 Nek2 Rad51 Cks2 Tubg1 Topbp1 Psmc3ip Nbn Suv39h2 Spo11 Brca2 Espl1 Sgol1 Psmd13 Sgol2 Syce2 Fanca Cks2 |
| nucleoside monophosphate metabolic process | GO:0009123 | 0.509 | Impdh2 Ampd3 Adcy7 Ampd2 Prps1 Cnp BC048355 Atic Paics Impdh2 Tyms Adss Tyms Nt5e Gart |
| protein targeting to membrane | GO:0006612 | 0.509 | Ssr3 Cacnb1 Timm9 Timm8a1 Timm8a1 Timm8b Macf1 Timm10 |
| somatic diversification of immune receptors via germline recombination within a single locus | GO:0002562 | 0.509 | Exo1 Icosl Xrcc6 Ercc1 Nbn Ung Swap70 |
| somatic cell DNA recombination | GO:0016444 | 0.509 | Exo1 Icosl Xrcc6 Ercc1 Nbn Ung Swap70 |
| antigen processing and presentation of peptide antigen via MHC class I | GO:0002474 | 0.532 | Fcgr1 Fcer1g Calr Mr1 Fcgr3 Hfe |
| cellular monovalent inorganic cation homeostasis | GO:0030004 | 0.532 | Mafg Aqp11 Atp1a3 Tesc Slc11a1 Sgk1 |
| somatic recombination of immunoglobulin genes during immune response | GO:0002204 | 0.541 | Exo1 Icosl Ercc1 Nbn Swap70 |
| isotype switching | GO:0045190 | 0.541 | Exo1 Icosl Ercc1 Nbn Swap70 |
| somatic diversification of immunoglobulins during immune response | GO:0002208 | 0.541 | Exo1 Icosl Ercc1 Nbn Swap70 |

Unique Biological processes in transcripts differentially spliced between naïve T cells and TCR-activated T cells

| **Gene Ontology Biological Process** | **GO ID** | **p-value of enrichment compared to whole genome** | **Gene symbols** |
| --- | --- | --- | --- |
| regulation of catabolic process | GO:0009894 | 0.392 | Gipc1 Serpinb1b Xpo1 Frap1 Gclc Cln3 Ndfip1 Pnpla2 Arntl Trim32 |
| gene silencing | GO:0016458 | 0.592 | Smarca4 Sirt2 Ncbp2 Smarca5 Scmh1 Piwil2 Tnrc6b Dnmt3a Sirt1 Crebl1 |
| microtubule-based movement | GO:0007018 | 0.928 | Kif2a Rhot2 Htt Tubb2c Dync1h1 Tuba1b Tuba1a Dynlrb1 Rhot1 Kif23 Kif3c Kif12 |
| mammary gland development | GO:0030879 | 0.97 | Bcl2l11 Lef1 Arhgap5 Scrib Med1 Ncoa3 Stat5a Tnfsf11 Tnfrsf11a Agpat6 |
| regulation of Rho protein signal transduction | GO:0035023 | 0.97 | Itsn2 Arhgef2 Frap1 4921505C17Rik Arhgef6 Plekhg2 Tiam1 Arhgef12 Scrib Vav1 |
| electron transport chain | GO:0022900 | 0.994 | Etfb Slc25a12 Ndufs1 Glrx2 Fdxr Uqcrh Fdx1l Ndufs2 Sdhd Etfa Ndufa12 |
| exocytosis | GO:0006887 | 0.999 | Exoc5 Exoc2 Vps33b Scrib Scamp1 Stx4a Tmed10 Arhgap17 Trpc2 Vamp7 |
| synaptic transmission | GO:0007268 | 1 | Gipc1 Atxn1 Scrib Gad2 Trappc4 Stx4a Atp1a2 Agtpbp1 Dvl1 Cln3 Park7 |
| cell morphogenesis involved in differentiation | GO:0000904 | 1 | Hprt1 Lef1 Hnrpab Ablim1 Mycbp2 Dvl1 Slit2 Nfatc1 Cck Sod1 Trim28 |
| cell-cell signaling | GO:0007267 | 1 | Gipc1 Atxn1 Gad2 Atp1a2 Frs2 Dvl1 Cln3 Fgf1 Htt Scrib Trappc4 Stx4a Agtpbp1 Dgat1 Park7 |

Unique Biological processes in transcripts differentially spliced between naïve T cells and TCR/CD28-activated T cells

| **Gene Ontology Biological Process** | **GO ID** | **p-value of enrichment compared to whole genome** | **Gene symbols** |
| --- | --- | --- | --- |
| nuclear mRNA splicing, via spliceosome | GO:0000398 | 0.009 | Prpf8 Sf3b1 Sfrs10 Mbnl1 ENSMUSG00000075538 Srrm1 Srpk2 Sip1 Hnrnpa1 Prpf6 Snrp70 Rbm5 |
| RNA splicing, via transesterification reactions | GO:0000375 | 0.009 | Prpf8 Sf3b1 Sfrs10 Mbnl1 ENSMUSG00000075538 Srrm1 Srpk2 Sip1 Hnrnpa1 Prpf6 Snrp70 Rbm5 |
| RNA splicing, via transesterification reactions with bulged adenosine as nucleophile | GO:0000377 | 0.009 | Prpf8 Sf3b1 Sfrs10 Mbnl1 ENSMUSG00000075538 Srrm1 Srpk2 Sip1 Hnrnpa1 Prpf6 Snrp70 Rbm5 |
| protein amino acid autophosphorylation | GO:0046777 | 0.107 | Frap1 Zap70 Dyrk1a Camkk2 Ern1 Pak2 Trpm7 Vrk1 Lck Camk2d Capza2 Stk4 Irak1 Crkrs |
| regulation of cellular response to stress | GO:0080135 | 0.109 | Bre Rnf168 Frap1 Dusp22 Axin1 Becn1 Pnp1 Eya3 Hipk3 Ccdc98 Map3k7 1300018I05Rik Cdc42se1 Irak1 Apbb1 |
| T cell differentiation | GO:0030217 | 0.12 | Satb1 Stat5b Ncor1 Zap70 Rps6 Ppp3cb Stat5a Bcl2 Ptprc Patz1 Prkdc Lck Sp3 Pknox1 Vav1 |
| regulation of mitotic cell cycle | GO:0007346 | 0.166 | Stat5b Mdm2 Rps6 Mycbp2 Bat2 Dlg1 Stat5a Bcl2 Ilkap Cdc23 Pml Cdc42 Cdc2l1 Zw10 Hdac3 Apbb1 Atm |
| response to ionizing radiation | GO:0010212 | 0.174 | Prkdc Bre Ccdc98 Rnf168 1300018I05Rik Bcl2 Pml Atm Eya3 Pnp1 Gpx1 |
| protein amino acid acylation | GO:0043543 | 0.174 | Myst4 Ing3 Csrp2bp Zdhhc1 Tcfe2a Zdhhc3 Gcn5l2 Apbb1 Ep400 Epc1 Yeats4 |
| glycerophospholipid metabolic process | GO:0006650 | 0.181 | Pik3c3 Pnpla6 Piga Pitpnc1 Ipmk Fig4 Serinc5 Sh3glb1 Pigk Pigl Chkb Alg12 C130090K23Rik Pip5k3 Pigo Ptdss1 |
| regulation of GTPase activity | GO:0043087 | 0.254 | Nf1 Tsc2 Git1 Frap1 Rabgap1l Tbc1d1 Als2 Tbc1d14 Pafah1b1 Arhgap1 Tbc1d22b Smap2 Tbc1d9b Vav1 Tbc1d13 Tbc1d12 |
| nucleoside triphosphate biosynthetic process | GO:0009142 | 0.266 | Atp5c1 Atp13a5 Atp6v1h Atp11a Atp6v0b Atp5b Pnp1 Atp1a1 Atp9b Tbpl1 Atp2b4 Atp5f1 Atp6v0d2 Atp2c1 Atp13a1 Atp5s |
| nucleus organization | GO:0006997 | 0.267 | Bnip3 Dullard Ndufa13 Sharpin Ahctf1 Pafah1b1 Acin1 Hmgb2 Pml Tbpl1 |
| ATP biosynthetic process | GO:0006754 | 0.343 | Atp5c1 Atp13a5 Atp6v1h Atp11a Atp6v0b Atp5b Atp1a1 Atp9b Atp2b4 Atp5f1 Atp6v0d2 Atp2c1 Atp13a1 Atp5s |
| purine ribonucleoside triphosphate biosynthetic process | GO:0009206 | 0.394 | Atp5c1 Atp13a5 Atp6v1h Atp11a Atp6v0b Atp5b Pnp1 Atp1a1 Atp9b Atp2b4 Atp5f1 Atp6v0d2 Atp2c1 Atp13a1 Atp5s |
| ribonucleoside triphosphate biosynthetic process | GO:0009201 | 0.394 | Atp5c1 Atp13a5 Atp6v1h Atp11a Atp6v0b Atp5b Pnp1 Atp1a1 Atp9b Atp2b4 Atp5f1 Atp6v0d2 Atp2c1 Atp13a1 Atp5s |
| purine nucleoside triphosphate biosynthetic process | GO:0009145 | 0.402 | Atp5c1 Atp13a5 Atp6v1h Atp11a Atp6v0b Atp5b Pnp1 Atp1a1 Atp9b Atp2b4 Atp5f1 Atp6v0d2 Atp2c1 Atp13a1 Atp5s |
| post-embryonic development | GO:0009791 | 0.448 | Hmgn1 Bcl2l11 Itpr1 Arid5b Bcl2 Invs Gnaq Zfa Psen1 Gnas Ercc2 Ccdc47 Myo1e Sox6 |
| purine ribonucleotide biosynthetic process | GO:0009152 | 0.483 | Atp5c1 Atp13a5 Atp6v1h Impdh1 Atp11a Atp6v0b Atp5b Pnp1 Atp1a1 Atp9b Atp2b4 Atp5f1 Atp6v0d2 Atp2c1 Atp13a1 Atp5s |
| ATP metabolic process | GO:0046034 | 0.484 | Atp5c1 Atp13a5 Atp6v1h Atp11a Atp6v0b Atp5b Atp1a1 Atp9b Atp2b4 Atp5f1 Atp6v0d2 Atp2c1 Atp13a1 Atp5s |
| regulation of Ras GTPase activity | GO:0032318 | 0.508 | Nf1 Git1 Frap1 Rabgap1l Tbc1d1 Als2 Tbc1d14 Pafah1b1 Tbc1d22b Smap2 Tbc1d9b Tbc1d13 Tbc1d12 |
| phosphoinositide metabolic process | GO:0030384 | 0.528 | Pik3c3 Pigk Pigl Piga Alg12 C130090K23Rik Pitpnc1 Ipmk Pip5k3 Pigo Fig4 |
| purine ribonucleoside triphosphate metabolic process | GO:0009205 | 0.526 | Atp5c1 Atp13a5 Atp6v1h Atp11a Atp6v0b Atp5b Pnp1 Atp1a1 Atp9b Atp2b4 Atp5f1 Atp6v0d2 Atp2c1 Atp13a1 Atp5s |
| ribonucleotide biosynthetic process | GO:0009260 | 0.558 | Atp5c1 Atp13a5 Atp6v1h Impdh1 Atp11a Atp6v0b Atp5b Pnp1 Atp1a1 Atp9b Atp2b4 Atp5f1 Atp6v0d2 Atp2c1 Atp13a1 Atp5s |
| regulation of lymphocyte differentiation | GO:0045619 | 0.565 | Stat5b Lck Il4ra Ptpn6 Zap70 Ndfip1 Stat5a Pnp1 Ptprc Ap3b1 |
| positive regulation of protein modification process | GO:0031401 | 0.587 | Ube2d1 Frap1 Aktip Psen1 Ncor1 Pias1 Jak2 Men1 Bcl2 Arnt Pml Rb1cc1 |
| glycerolipid biosynthetic process | GO:0045017 | 0.604 | Sh3glb1 Pigk Pigl Chkb Piga Alg12 C130090K23Rik Pigo Gnpat Ptdss1 |
| purine nucleoside triphosphate metabolic process | GO:0009144 | 0.602 | Atp5c1 Atp13a5 Atp6v1h Atp11a Atp6v0b Atp5b Pnp1 Atp1a1 Atp9b Atp2b4 Atp5f1 Atp6v0d2 Atp2c1 Atp13a1 Atp5s |
| myeloid cell differentiation | GO:0030099 | 0.629 | Ncor1 Rps6 Acin1 Ostm1 Smad5 Pml Cbfb Sp3 Add1 Psen1 Ercc2 Pknox1 Jak2 Sox6 |
| fat cell differentiation | GO:0045444 | 0.664 | Mkks Bnip3 Sdf4 Prdm16 Cby1 Tbl1x Aldh6a1 Med1 Ncor2 Gpx1 |
| regulation of cell cycle process | GO:0010564 | 0.703 | Cdc2l1 Zw10 Mycbp2 Bat2 Rock2 Ilkap Pml Cdc23 Apbb1 Atm Cdc42 |
| MAPKKK cascade | GO:0000165 | 0.751 | Nf1 Ptpn11 Spag9 Map2k5 Map2k1ip1 Mapk9 Map2k4 Ptprc Map3k7 Psen1 Mapk1 Dusp19 Smad1 Rab26 Rb1cc1 |
| ossification | GO:0001503 | 0.766 | Nf1 Ubb Col1a1 Bcl2 Men1 Smad5 Cbfb Pex7 Sp3 Gnas Ercc2 Hdac5 Tnfrsf11a Smad1 |
| purine nucleotide biosynthetic process | GO:0006164 | 0.791 | Atp5c1 Adcy2 Atp13a5 Atp6v1h Impdh1 Atp11a Atp6v0b Atp5b Atp1a1 Pnp1 Atp9b Atp2b4 Atp5f1 Atp6v0d2 Atp2c1 Atp13a1 Atp5s |
| nucleosome assembly | GO:0006334 | 0.834 | Hist1h1e H2afz Myst4 Smarca4 Hist1h1a H3f3b 6430706D22Rik H2afy Nap1l4 Tspyl2 |
| negative regulation of cellular protein metabolic process | GO:0032269 | 0.834 | Ptpn6 Eif2c3 Psen1 Gclc RP23 Tia1 Impact Vps28 Eif2c2 Ptprc |
| regulation of gene expression, epigenetic | GO:0040029 | 0.834 | Smarca4 Eif2c3 Sirt2 Eed Mbd1 Aof1 Rbm3 H2afy Eif2c2 Rbm3 Epc1 |
| chromatin assembly | GO:0031497 | 0.865 | Hist1h1e H2afz Myst4 Smarca4 Hist1h1a H3f3b 6430706D22Rik H2afy Nap1l4 Tspyl2 |
| telencephalon development | GO:0021537 | 0.879 | Mkks Nf1 Rac1 Hdac1 Lef1 Htt Psen1 Ncor2 Pafah1b1 Gcn5l2 |
| nucleosome organization | GO:0034728 | 0.879 | Hist1h1e H2afz Myst4 Smarca4 Hist1h1a H3f3b 6430706D22Rik H2afy Nap1l4 Tspyl2 |
| protein-DNA complex assembly | GO:0065004 | 0.879 | Hist1h1e H2afz Myst4 Smarca4 Hist1h1a H3f3b 6430706D22Rik H2afy Nap1l4 Tspyl2 |
| gastrulation | GO:0007369 | 0.901 | Lef1 Htt Prkar1a Lrp6 Rps6 D3Ertd300e Arfrp1 Hira Ext1 Smad1 |
| heart morphogenesis | GO:0003007 | 0.901 | Smarca4 Psen1 Ncor2 ENSMUSG00000072684 Mybpc3 Egln1 Rxra Mib1 Col5a1 Col11a1 |
| calcium ion homeostasis | GO:0055074 | 0.907 | Pygm Itpr1 Bcl2 Ptprc Ryr2 Lck Gnb1 Psen1 Trpc2 Ccdc47 Atp2c1 Trpc2 Clstn1 |
| bone development | GO:0060348 | 0.908 | Nf1 Ubb Col1a1 Bcl2 Men1 Smad5 Cbfb Pex7 Sp3 Gnas Ercc2 Hdac5 Tnfrsf11a Smad1 |
| lipoprotein metabolic process | GO:0042157 | 0.911 | Pigk Pigl Piga Zdhhc1 Alg12 C130090K23Rik Zdhhc3 Pigo Rabggta Atm |
| negative regulation of protein metabolic process | GO:0051248 | 0.911 | Ptpn6 Eif2c3 Psen1 Gclc RP23 Tia1 Impact Vps28 Eif2c2 Ptprc |
| gonad development | GO:0008406 | 0.917 | Stat5b Bcl2l11 Fndc3a Zfa Jak3 Eif2b2 Fancg Bcl2 Stat5a Hmgb2 Ube3a |
| germ cell development | GO:0007281 | 0.935 | Mkks Fndc3a Frap1 Zfa Pafah1b1 Fancg Bcl2 Smad5 Rps6kb1 Hmgb2 Tial1 Tbpl1 |
| cellular calcium ion homeostasis | GO:0006874 | 0.952 | Ryr2 Pygm Lck Gnb1 Itpr1 Psen1 Trpc2 Atp2c1 Bcl2 Clstn1 Trpc2 Ptprc |
| negative regulation of cellular component organization | GO:0051129 | 0.957 | Nrp1 Clasp2 Cit Psen1 Mid1 Capza2 Ttc3 Rtn4 Capzb Spnb2 Atm |
| cell fate commitment | GO:0045165 | 0.96 | Cdon Prdm1 Tcfe2a Fgfr4 Bcl2 Men1 Smad5 Pml Cdc42 Prkdc Myl2 Itgb1 Smarca4 Psen1 Smad1 Sox6 |
| response to peptide hormone stimulus | GO:0043434 | 0.961 | Stat5b Frap1 Eif2b2 Capza2 RP23 Jak2 Col1a1 Bcl2 Stat5a Phip Ptpn1 |
| response to drug | GO:0042493 | 0.973 | Lck Aldh1a1 Tcfe2a Capza2 Abcb1a Gnas Hiat1 Bcl2 Hadha Atp1a1 |
| embryonic appendage morphogenesis | GO:0035113 | 0.976 | Fto Gnaq Lef1 Fbxw4 Smarca4 Psen1 Lrp6 Med1 Gnas Mbnl1 Lmbr1 |
| embryonic limb morphogenesis | GO:0030326 | 0.976 | Fto Gnaq Lef1 Fbxw4 Smarca4 Psen1 Lrp6 Med1 Gnas Mbnl1 Lmbr1 |
| response to light stimulus | GO:0009416 | 0.979 | Nf1 Hmgn1 Ube4b Gnb1 Sdf4 Htt Ric8 Ercc2 Bcl2 Pml Apbb1 |
| placenta development | GO:0001890 | 0.978 | Sp3 Lef1 Prdm1 Tcfeb Rps6 Mapk1 Med1 Birc6 Arnt Egln1 |
| development of primary sexual characteristics | GO:0045137 | 0.987 | Stat5b Bcl2l11 Fndc3a Zfa Jak3 Eif2b2 Fancg Bcl2 Stat5a Hmgb2 Ube3a |
| regulation of cell morphogenesis | GO:0022604 | 0.994 | Nrp1 Cit Limk1 Psen1 Epb4 Epb4 Ttc3 Cdc42se1 Rtn4 Sema4d |
| limb morphogenesis | GO:0035108 | 0.997 | Fto Gnaq Lef1 Fbxw4 Smarca4 Psen1 Lrp6 Med1 Gnas Mbnl1 Lmbr1 |
| appendage morphogenesis | GO:0035107 | 0.997 | Fto Gnaq Lef1 Fbxw4 Smarca4 Psen1 Lrp6 Med1 Gnas Mbnl1 Lmbr1 |
| circulatory system process | GO:0003013 | 0.997 | Fli1 Myl2 Camk2d Gclc Capza2 Hsn2 Smad5 Mybpc3 Nisch Atp1a1 Gpx1 |
| blood circulation | GO:0008015 | 0.997 | Fli1 Myl2 Camk2d Gclc Capza2 Hsn2 Smad5 Mybpc3 Nisch Atp1a1 Gpx1 |
| developmental growth | GO:0048589 | 0.997 | Smarca4 Psen1 Med1 Ints1 ENSMUSG00000072684 Bcl2 Taf8 Plaur Gpx1 Ube3a |
| limb development | GO:0060173 | 0.998 | Fto Gnaq Lef1 Fbxw4 Smarca4 Psen1 Lrp6 Med1 Gnas Mbnl1 Lmbr1 |
| appendage development | GO:0048736 | 0.998 | Fto Gnaq Lef1 Fbxw4 Smarca4 Psen1 Lrp6 Med1 Gnas Mbnl1 Lmbr1 |
| anion transport | GO:0006820 | 0.999 | Clcn6 Htt Slc25a12 Psen1 Slc4a8 Capza2 Slc20a2 Clic1 Gabrr2 Slc25a22 Slc26a2 Slc26a8 |
| regulation of cell size | GO:0008361 | 0.999 | Tsc2 Nrp1 Frap1 Limk1 Ndufa13 Rtn4 Bcl2 Pml Apbb1 Rb1cc1 |
| anterior/posterior pattern formation | GO:0009952 | 1 | Prkdc Hipk1 Lef1 Htt Sf3b1 Psen1 Lrp6 Hoxa7 Mllt3 Gcn5l2 Mib1 Atm |
| cell-cell adhesion | GO:0016337 | 1 | Pard3 Cdon Fndc3a Rapgef1 Cd164 Dlg1 Bcl2 Cldn15 Ptprc Cdc42 Col11a1 Cyfip2 Lef1 Psen1 Cd2ap Atp2c1 Clstn1 Pcdha10 |
| Wnt receptor signaling pathway | GO:0016055 | 1 | Zranb1 Lef1 Fbxw4 Lrp6 Tax1bp3 Axin1 Csnk1d AI314760 Aes Invs |
| regulation of neurogenesis | GO:0050767 | 1 | Nf1 Nrp1 Cit Limk1 Psen1 Ttc3 Rtn4 Bcl2 Sema4d Mib1 |
| regulation of cell development | GO:0060284 | 1 | Nf1 Cdon Nrp1 Cit Limk1 Psen1 Ttc3 Rtn4 Bcl2 Sema4d Hdac5 Mib1 |
| urogenital system development | GO:0001655 | 1 | Nf1 Bcl2l11 Cd44 Arid5b Myo1e Dlg1 Bcl2 Stat5a Rxra Invs Ube3a |
| extracellular structure organization | GO:0043062 | 1 | Nf1 Smarca4 Als2 Ercc2 Aplp2 Apbb1 Gnpat Abi3bp Col5a1 Col11a1 Pomt1 |
| regulation of nervous system development | GO:0051960 | 1 | Nf1 Nrp1 Cit Limk1 Psen1 Ttc3 Rtn4 Bcl2 Sema4d Mib1 |
| regionalization | GO:0003002 | 1 | Hoxa7 Mib1 Prkdc Hipk1 Ift172 Htt Lef1 Lrp6 Psen1 Sf3b1 Mllt3 Gcn5l2 Arl13b Atm |
| regulation of system process | GO:0044057 | 1 | Nf1 Ryr2 Ptpn11 Htt Psen1 Bat2 Mybpc3 Nisch Atp1a1 Ncdn Lnpep |
| cell projection morphogenesis | GO:0048858 | 1 | Mkks Rac1 Ift172 Ptpn11 Nrp1 Als2 Mycbp2 Bcl2 Apbb1 Pcnt Numb |
| neuron projection development | GO:0031175 | 1 | Rac1 Ptpn11 Nrp1 Cit Exoc7 Als2 Mycbp2 Bcl2 Apbb1 Numb |
| sensory perception | GO:0007600 | 1 | Mkks Gnb1 Scnn1g Axin1 Lxn Wdr1 Gabrr2 Diap1 Opa1 Gtf3c2 Otog Gpx1 |
