# Supplementary material for: CD28 Costimulation Regulates Genome-Wide Effects on Alternative Splicing
Source: PLoS One. 2012 Jun 29;7(6):e40032. doi: 10.1371/journal.pone.0040032 (PMC3386953; doi:10.1371/journal.pone.0040032)
Supplement: Table S6 — Biological processes of the 1,047 transcripts differentially spliced between TCR-activated and TCR/CD28-activated T cells. (DOC) [file pone.0040032.s009.doc]

**Table S6**

**Biological processes of the 1,047 transcripts differentially spliced between TCR-activated T cells and TCR/CD28-**activated T cells

| **Gene Ontology Biological Process** | **GO ID** | **p-value of enrichment compared to whole genome** | **Gene symbols** |
| --- | --- | --- | --- |
| RNA processing | GO:0006396 | 1.23E-09 | Ftsj1 Exosc3 Sart3 Prpf40b Bat1a Isy1 AL118220 Tgs1 Sf3b1 Pa2g4 Txnl4a Cdc5l Hnrpk Syncrip Cwc15 Ddx20 Cstf2 Wdsof1 Thoc7 Ddx51 Nsun2 Rpl10a Snrpd1 Zmat5 Sfrs7 Sfrs2 Sfrs10 Ftsj3 Pnpt1 Ints12 BC028454 Bop1 Hnrnpa1 Ddx39 Nola1 Rnps1 Krr1 Cdk5rap1 Ints1 Snrp70 Dicer1 Elac2 Hnrpll Usp52 Ssu72 Exosc7 Ssb Exosc1 Btbd14b Dep1 Cpsf4 Pabpc1 Ptbp1 Ints8 Ints7 Rpp30 Nhp2l1 Ddx5 Rbm22 Ints2 Xrn2 Hnrnpa2b1 AI316844 Sfrs5 Rbm28 Pdcd11 |
| nucleocytoplasmic transport | GO:0006913 | 2.56E-06 | Kpna2 Kpnb1 Cse1l Hspa9 Nup214 Xpo6 Tpr Ddx25 Npm1 Ipo13 Spnb2 Ran Ipo9 Htt Tnpo1 Ipo5 Trp53 Ppp3ca Ipo4 Camk4 Nxt1 Hnrnpa1 Adam10 Ipo7 |
| nuclear transport | GO:0051169 | 2.64E-06 | Kpna2 Kpnb1 Cse1l Hspa9 Nup214 Xpo6 Tpr Ddx25 Npm1 Ipo13 Spnb2 Ran Ipo9 Htt Tnpo1 Ipo5 Trp53 Ppp3ca Ipo4 Camk4 Nxt1 Hnrnpa1 Adam10 Ipo7 |
| nuclear import | GO:0051170 | 7.41E-06 | Kpnb1 Kpna2 Cse1l Nup214 Xpo6 Tpr Ipo13 Spnb2 Ran Ipo9 Htt Tnpo1 Ipo5 Trp53 Ppp3ca Ipo4 Hnrnpa1 Ipo7 |
| protein import into nucleus | GO:0006606 | 2.39E-05 | Kpnb1 Kpna2 Cse1l Nup214 Xpo6 Tpr Ipo13 Spnb2 Ran Ipo9 Htt Tnpo1 Ipo5 Trp53 Ppp3ca Ipo4 Ipo7 |
| mRNA metabolic process | GO:0016071 | 2.21E-05 | Rnps1 Prpf40b Hnrpdl Snrp70 Isy1 Bat1a Hnrpll Mettl3 AL118220 Usp52 Sf3b1 Pan3 Ssu72 Txnl4a Cdc5l Dep1 Cpsf4 Pabpc1 Ptbp1 Hnrpk Syncrip Cwc15 Ddx20 Cstf2 Thoc7 Nhp2l1 Vegfa Ddx5 Snrpd1 Zmat5 Rbm22 Sfrs7 Sfrs2 Sfrs10 Pnpt1 Xrn2 Hnrnpa2b1 Auh Sfrs5 Rbm28 Eif3e Hnrnpa1 Ddx39 |
| protein localization in nucleus | GO:0034504 | 6.36E-05 | Kpnb1 Kpna2 Cse1l Nup214 Xpo6 Tpr Ipo13 Spnb2 Ran Ipo9 Htt Tnpo1 Ipo5 Trp53 Ppp3ca Ipo4 Ipo7 |
| protein import | GO:0017038 | 1.45E-04 | Kpnb1 Kpna2 Cse1l Nup214 Xpo6 Tpr Grpel2 Ipo13 Spnb2 Ran Ipo9 Htt Tnpo1 Timm13 Ipo5 Trp53 Ppp3ca Ipo4 Ipo7 |
| protein targeting | GO:0006605 | 3.55E-04 | Gipc1 Kpna2 Kpnb1 Cse1l Hspa9 Nup214 Atg4b Xpo6 Tpr Grpel2 Ipo13 Spnb2 Ran Ipo9 Htt Tnpo1 Timm13 Ipo5 Trp53 Ppp3ca Ipo4 Nxt1 Ipo7 Macf1 |
| protein localization in organelle | GO:0033365 | 3.71E-04 | Kpnb1 Kpna2 Cse1l Nup214 Xpo6 Tpr Grpel2 Ipo13 Spnb2 Ran Ipo9 Htt Tnpo1 Timm13 Ipo5 Trp53 Ppp3ca Ipo4 Ipo7 Macf1 |
| mRNA processing | GO:0006397 | 4.01E-04 | Rnps1 Prpf40b Snrp70 Bat1a Isy1 Hnrpll AL118220 Usp52 Sf3b1 Ssu72 Txnl4a Cdc5l Dep1 Cpsf4 Pabpc1 Ptbp1 Hnrpk Syncrip Cwc15 Ddx20 Cstf2 Thoc7 Nhp2l1 Ddx5 Snrpd1 Zmat5 Rbm22 Sfrs7 Sfrs2 Xrn2 Sfrs10 Hnrnpa2b1 Sfrs5 Rbm28 Hnrnpa1 Ddx39 |
| protein catabolic process | GO:0030163 | 3.95E-04 | Edem1 Ddb1 Aup1 Mgrn1 Pias3 Usp3 Rnf20 Siah1a Rnf41 Hectd3 Smurf1 Rad23a Ube2e3 Rnf111 Usp47 Ube2k Psmc3 Anapc1 Zc3hc1 Psenen Vprbp Mycbp2 Mib2 Ube3c Senp6 Psen1 Psma7 Cnot4 Usp4 Rfwd2 Socs6 Fbxl10 Psmc6 Foxred2 Dcun1d1 Xiap Cdc23 Ube4a Usp52 Tbl1x March7 Ube2z Psmb5 Map1lc3b Adam10 Med20 Rnf123 Uchl5 Hsp90b1 Atg4b Trim33 Cast Usp12 Prkcq Socs1 Malt1 Mid1 Atg3 Myh9 Psma6 |
| cellular macromolecule catabolic process | GO:0044265 | 3.87E-04 | Edem1 Ddb1 Aup1 Mgrn1 Pias3 Usp3 Rnf20 Siah1a Rnf41 Hectd3 Smurf1 Rad23a Ube2e3 Rnf111 Usp47 Atm Ube2k Anapc1 Zc3hc1 Psenen Vprbp Mycbp2 Mib2 Ube3c Senp6 Psen1 Pnpt1 Psma7 Eif3e Cnot4 Usp4 Rfwd2 Rnps1 Socs6 Fbxl10 Foxred2 Dcun1d1 Xiap Cdc23 Ube4a Usp52 Pan3 March7 Tbl1x Ube2z Psmb5 Map1lc3b Adam10 Med20 Rnf123 Uchl5 Hsp90b1 Atg4b Trim33 Prkcq Socs1 Usp12 Malt1 Mid1 Atg3 Xrn2 Myh9 Auh Psma6 |
| macromolecule catabolic process | GO:0009057 | 4.87E-04 | Edem1 Ddb1 Aup1 Mgrn1 Pias3 Usp3 Rnf20 Siah1a Rnf41 Hectd3 Smurf1 Rad23a Ube2e3 Rnf111 Usp47 Atm Ube2k Psmc3 Anapc1 Zc3hc1 Psenen Vprbp Mycbp2 Mib2 Ube3c Senp6 Psen1 Pnpt1 Psma7 Eif3e Cnot4 Usp4 Rfwd2 Rnps1 Socs6 Fbxl10 Psmc6 Foxred2 Dcun1d1 Xiap Cdc23 Ube4a Usp52 Pan3 March7 Tbl1x Ube2z Psmb5 Map1lc3b Adam10 Med20 Rnf123 Uchl5 Hsp90b1 Atg4b Trim33 Cast Prkcq Socs1 Usp12 Malt1 Mid1 Atg3 Xrn2 Myh9 Auh Psma6 |
| RNA splicing | GO:0008380 | 4.75E-04 | Rnps1 Prpf40b Snrp70 Bat1a Isy1 AL118220 Sf3b1 Txnl4a Cdc5l Dep1 Pabpc1 Ptbp1 Hnrpk Syncrip Cwc15 Ddx20 Thoc7 Nhp2l1 Ddx5 Snrpd1 Zmat5 Sfrs7 Rbm22 Sfrs2 Sfrs10 Hnrnpa2b1 Sfrs5 Rbm28 Hnrnpa1 Ddx39 |
| ncRNA metabolic process | GO:0034660 | 5.45E-04 | Krr1 Cdk5rap1 Ftsj1 Exosc3 Ints1 Tpr Hars Elac2 Nars Exosc7 Pa2g4 Exosc1 Btbd14b Gars Ints8 Rpp30 Ints7 Rars Wdsof1 Ddx51 Nsun2 Ints2 Ftsj3 AI316844 Ints12 Farsa Bop1 Kars Pdcd11 Nola1 |
| proteolysis involved in cellular protein catabolic process | GO:0051603 | 7.41E-04 | Edem1 Ddb1 Aup1 Mgrn1 Pias3 Usp3 Rnf20 Siah1a Rnf41 Hectd3 Smurf1 Rad23a Ube2e3 Rnf111 Usp47 Ube2k Anapc1 Zc3hc1 Psenen Vprbp Mycbp2 Mib2 Ube3c Senp6 Psen1 Psma7 Cnot4 Usp4 Rfwd2 Socs6 Fbxl10 Foxred2 Dcun1d1 Xiap Cdc23 Ube4a Usp52 Tbl1x March7 Ube2z Psmb5 Map1lc3b Adam10 Med20 Rnf123 Uchl5 Hsp90b1 Atg4b Trim33 Usp12 Prkcq Socs1 Malt1 Mid1 Atg3 Myh9 Psma6 |
| cellular protein catabolic process | GO:0044257 | 8.28E-04 | Edem1 Ddb1 Aup1 Mgrn1 Pias3 Usp3 Rnf20 Siah1a Rnf41 Hectd3 Smurf1 Rad23a Ube2e3 Rnf111 Usp47 Ube2k Anapc1 Zc3hc1 Psenen Vprbp Mycbp2 Mib2 Ube3c Senp6 Psen1 Psma7 Cnot4 Usp4 Rfwd2 Socs6 Fbxl10 Foxred2 Dcun1d1 Xiap Cdc23 Ube4a Usp52 Tbl1x March7 Ube2z Psmb5 Map1lc3b Adam10 Med20 Rnf123 Uchl5 Hsp90b1 Atg4b Trim33 Usp12 Prkcq Socs1 Malt1 Mid1 Atg3 Myh9 Psma6 |
| cell aging | GO:0007569 | 2.73E-03 | Htt Mif Pdcd4 Wrn Trp53 BC028454 ENSMUSG00000072684 Npm1 Nup62 Brca2 |
| intracellular transport | GO:0046907 | 2.63E-03 | Ap2b1 Ap4s1 Cog3 Kpna2 Hspa9 Nup214 Chmp7 Timm23 Xpo6 Myo9b Ddx25 Tpr Vps4b Timm17b Grpel2 Tmed10 Spnb2 Ran Ap1s3 Cux1 Ipo9 Htt Timm13 Klc1 Trp53 Ppp3ca Ipo4 Adam10 Gipc1 Kpnb1 Cse1l Atg4b Npm1 Ipo13 Tnpo1 Vps29 Psen1 Cd74 Myh9 Ipo5 Copz1 Camk4 Nxt1 Hnrnpa1 Ipo7 Macf1 Ucp2 |
| ncRNA processing | GO:0034470 | 3.34E-03 | Krr1 Cdk5rap1 Exosc3 Ftsj1 Ints8 Ints1 Ints7 Rpp30 Wdsof1 Ddx51 Nsun2 Dicer1 Elac2 Ints2 Ftsj3 AI316844 Exosc7 Pa2g4 Ints12 Exosc1 Bop1 Btbd14b Pdcd11 Nola1 |
| cellular protein complex assembly | GO:0043623 | 3.35E-03 | Kpnb1 Cse1l Ptk2 Elp2 Xpo6 Tbca Tbce Tubb5 Ipo13 Tes Ipo9 1110034A24Rik Tnpo1 Calr Ipo5 Diap1 Tuba4a Ipo4 Ipo7 |
| protein transport | GO:0015031 | 3.73E-03 | Ap2b1 Cog3 Timm23 Nup214 Xpo6 Tpr Timm17b Tmed10 Spnb2 Arf3 Kif20a Timm13 Trp53 Eps15 Pldn Gipc1 Kpnb1 Cse1l Gdi2 Gorasp1 Pitpnm1 Pex1 Zfyve20 Vps29 Psen1 Cd74 Sft2d1 Erp29 Ipo7 Rab1b Ap4s1 Exoc2 Kpna2 Pex14 Hspa9 Chmp7 Arf4 Vps4b Grpel2 Ran Ap1s3 Pom121 Ipo9 1700034H14Rik Htt Rab37 Ppp3ca Ipo4 Nup62 Atg4b D3Ucla1 Ipo13 Tnpo1 Atg3 Myh9 Ipo5 Copz1 Fras1 Nxt1 Lcp2 Lman2 Macf1 Cep290 |
| protein localization | GO:0008104 | 4.37E-03 | Ap2b1 Cog3 Kifap3 Timm23 Nup214 Xpo6 Tpr Timm17b Tmed10 Spnb2 Sufu Kif20a Arf3 Timm13 Trp53 Ulk1 Eps15 Pldn Gipc1 Kpnb1 Cse1l Gdi2 Rdx Gorasp1 Pitpnm1 Pex1 Pex2 Zfyve20 Vps29 Psen1 Cd74 Sft2d1 Erp29 Ipo7 Rab1b Ap4s1 Exoc2 Kpna2 Pex14 Hspa9 Chmp7 Arf4 Vps4b Grpel2 Ran Ap1s3 Pom121 Ipo9 1700034H14Rik Htt Rab37 Ppp3ca Ipo4 Nup62 Atg4b D3Ucla1 Npm1 Ipo13 Tnpo1 Atg3 Myh9 Ipo5 Sin3a Copz1 Fras1 Nxt1 Lcp2 Lman2 Macf1 Cep290 |
| establishment of protein localization | GO:0045184 | 4.41E-03 | Ap2b1 Cog3 Timm23 Nup214 Xpo6 Tpr Timm17b Tmed10 Spnb2 Arf3 Kif20a Timm13 Trp53 Eps15 Pldn Gipc1 Kpnb1 Cse1l Gdi2 Gorasp1 Pitpnm1 Pex1 Zfyve20 Vps29 Psen1 Cd74 Sft2d1 Erp29 Ipo7 Rab1b Ap4s1 Exoc2 Kpna2 Pex14 Hspa9 Chmp7 Arf4 Vps4b Grpel2 Ran Ap1s3 Pom121 Ipo9 1700034H14Rik Htt Rab37 Ppp3ca Ipo4 Nup62 Atg4b D3Ucla1 Ipo13 Tnpo1 Atg3 Myh9 Ipo5 Copz1 Fras1 Nxt1 Lcp2 Lman2 Macf1 Cep290 |
| modification-dependent macromolecule catabolic process | GO:0043632 | 4.41E-03 | Edem1 Ddb1 Aup1 Pias3 Mgrn1 Usp3 Rnf20 Siah1a Rnf41 Hectd3 Smurf1 Rad23a Ube2e3 Rnf111 Usp47 Ube2k Anapc1 Zc3hc1 Vprbp Mycbp2 Mib2 Ube3c Senp6 Psma7 Cnot4 Usp4 Rfwd2 Socs6 Fbxl10 Foxred2 Dcun1d1 Xiap Cdc23 Ube4a Usp52 Tbl1x March7 Ube2z Psmb5 Map1lc3b Med20 Rnf123 Uchl5 Hsp90b1 Atg4b Trim33 Usp12 Socs1 Malt1 Mid1 Atg3 Psma6 |
| modification-dependent protein catabolic process | GO:0019941 | 4.41E-03 | Edem1 Ddb1 Aup1 Pias3 Mgrn1 Usp3 Rnf20 Siah1a Rnf41 Hectd3 Smurf1 Rad23a Ube2e3 Rnf111 Usp47 Ube2k Anapc1 Zc3hc1 Vprbp Mycbp2 Mib2 Ube3c Senp6 Psma7 Cnot4 Usp4 Rfwd2 Socs6 Fbxl10 Foxred2 Dcun1d1 Xiap Cdc23 Ube4a Usp52 Tbl1x March7 Ube2z Psmb5 Map1lc3b Med20 Rnf123 Uchl5 Hsp90b1 Atg4b Trim33 Usp12 Socs1 Malt1 Mid1 Atg3 Psma6 |
| DNA metabolic process | GO:0006259 | 5.40E-03 | Dbf4 Ddb1 Apex2 Trex1 Mbd1 Dnajc2 Eif4e3 Fanci Ccdc111 Ssbp1 Hmgb2 Mcm6 Dclre1a Rad23a Rbms1 Fancc Trp53 Top2b Atm Smc3 Pols LOC433762 Dnmt3b Mms19 Gtf2h1 Wrn Polg Rad52 Topbp1 Brca2 Uimc1 Obfc2a Rtel1 Orc2l Rad50 Aof1 Xrn2 Sin3a Rbbp7 BC028454 Smc6 Trpc2 Fanca Recql4 |
| intracellular protein transport | GO:0006886 | 7.68E-03 | Ap2b1 Ap4s1 Cog3 Kpna2 Hspa9 Nup214 Timm23 Xpo6 Tpr Timm17b Grpel2 Tmed10 Spnb2 Ap1s3 Ran Ipo9 Htt Timm13 Trp53 Ppp3ca Ipo4 Gipc1 Kpnb1 Cse1l Atg4b Ipo13 Tnpo1 Cd74 Ipo5 Copz1 Nxt1 Ipo7 Macf1 |
| aging | GO:0007568 | 0.012 | Wrn Polg Npm1 D3Ucla1 Brca2 Htt Mif Pdcd4 Calr Trp53 ENSMUSG00000072684 BC028454 Nup62 |
| cellular macromolecular complex assembly | GO:0034622 | 0.012 | Ptk2 Elp2 Xpo6 Tbca Tbce H2afy Hist1h2ae Tes Ipo9 Dicer1 Calr Tuba4a Ipo4 Nap1l1 Rpl24 Kpnb1 Myst4 Cse1l Tubb5 Ipo13 Smarca2 1110034A24Rik Tnpo1 Ipo5 Diap1 Eif3s10 Ipo7 |
| cellular protein localization | GO:0034613 | 0.014 | Ap2b1 Ap4s1 Cog3 Kpna2 Hspa9 Nup214 Timm23 Xpo6 Tpr Timm17b Grpel2 Tmed10 Spnb2 Ap1s3 Ran Ipo9 Htt Timm13 Trp53 Ppp3ca Ipo4 Gipc1 Kpnb1 Cse1l Atg4b Ipo13 Tnpo1 Cd74 Ipo5 Sin3a Copz1 Nxt1 Ipo7 Macf1 |
| cellular macromolecular complex subunit organization | GO:0034621 | 0.015 | Ptk2 Wasl Elp2 Xpo6 Tbca Tbce H2afy Hist1h2ae Tes Ipo9 Dicer1 Calr Tuba4a Ipo4 Nap1l1 Rpl24 Kpnb1 Myst4 Cse1l Tubb5 Ipo13 Smarca2 1110034A24Rik Tnpo1 Xrn2 Ipo5 Diap1 Eif3s10 Ipo7 |
| cellular macromolecule localization | GO:0070727 | 0.015 | Ap2b1 Ap4s1 Cog3 Kpna2 Hspa9 Nup214 Timm23 Xpo6 Tpr Timm17b Grpel2 Tmed10 Spnb2 Ap1s3 Ran Ipo9 Htt Timm13 Trp53 Ppp3ca Ipo4 Gipc1 Kpnb1 Cse1l Atg4b Ipo13 Tnpo1 Cd74 Ipo5 Sin3a Copz1 Nxt1 Ipo7 Macf1 |
| translation | GO:0006412 | 0.015 | Kpna2 Mrpl55 Tpr Rpl30 Rps28 Gspt1 Eif4e2 Hars Nars Mrps16 Rps2 Rpl19 Gars Mrpl17 Rpl24 Rars Rpl36a Fau Eef1d Eif3c Rpl10a Mrpl4 Eif2s3x Rpl19 Rpl10 Tsfm Ptrh1 Eif4b Farsa Rps9 Hbs1l Kars Eif3e Eif3s10 |
| phosphorylation | GO:0016310 | 0.018 | Mknk2 Atp6v1a C130006E23 Pink1 Pim2 Spnb2 Rps6kb1 Scyl3 Ptprc Rps6kb2 Igf1r Hsf1 Stk4 Ulk1 Brd4 Atm Mknk1 Spag9 ENSMUSG00000075466 Pdpk1 Cdc2l1 Araf Riok2 Pgk1 Egf Atp5g3 Psen1 Cd74 Csk Pctk1 Nrk Camk4 Cdk4 Prkaa1 Ptk2 Stk17b Pgk1 Tes Fyn Vrk1 Trim28 Lck Prkcd Akt2 Pan3 Csnk1d Pkn1 Adam10 Vrk2 Zap70 Txk Atp6v0b Prkce Mvd Pak4 Atp5b Nme1 Prkcq Trib3 Atp6v0d2 Nek4 Aurkb Rps6ka1 Clk1 Plk3 |
| cell cycle | GO:0007049 | 0.029 | Rb1 Siah1a Trp53bp2 Numa1 Nfatc1 Pcnt EG667723 Dclre1a Trp53 Txnl4a Cdc5l Incenp Atm Rpl24 Smc3 Anapc1 Zc3hc1 Tubb5 Topbp1 Cdc2l1 Zfp655 Itgb1 Rad50 Cdk2ap1 BC028454 Ccar1 Ccnd3 Tacc2 Fanca Dbf4 Cdk4 Calm1 Cdc25c Dnajc2 Vps4b Fanci Pin1 Ncapd3 Ran Cdc23 Mcm6 Ppp3ca Mapre2 Pols Arhgap8 Anp32b Npm1 Brca2 Appl1 Zwint Myh9 Nek4 Sin3a Aurkb Plk3 Macf1 |
| regulation of cell cycle | GO:0051726 | 0.029 | Dbf4 Cdk4 Cdk5rap1 Nup214 Rb1 Cdc23 Ptprc Hectd3 Sin3b Calr Kras Trp53 Rpl24 Atm Mycbp2 Npm1 Brca2 Zfp655 Uimc1 Cdc2l1 Itgb1 Obfc2a Atf5 Ccnd3 Bop1 Recql4 |
| phosphorus metabolic process | GO:0006793 | 0.029 | Mknk2 Atp6v1a C130006E23 Pink1 Pim2 Spnb2 Rps6kb1 Scyl3 Ptprc Gm1066 EG667723 Rps6kb2 Igf1r Hsf1 Stk4 Ulk1 Brd4 Atm Mknk1 Spag9 ENSMUSG00000075466 Pdpk1 Cdc2l1 Araf Riok2 Pgk1 Egf Atp5g3 Psen1 Cd74 Csk Pctk1 Nrk Camk4 Cdk4 Ppa2 Inpp5a Prkaa1 Ptk2 Cdc25c Stk17b Pgk1 Fyn Tes Vrk1 Trim28 Lck Prkcd Akt2 Ppm1b Pan3 Csnk1d Ppp3ca Pkn1 Ptpn4 Adam10 Vrk2 Zap70 Txk Atp6v0b Prkce Mvd Pak4 Atp5b Nme1 Prkcq Trib3 Atp6v0d2 Nek4 Aurkb Rps6ka1 Mtmr12 Clk1 Plk3 |
| phosphate metabolic process | GO:0006796 | 0.029 | Mknk2 Atp6v1a C130006E23 Pink1 Pim2 Spnb2 Rps6kb1 Scyl3 Ptprc Gm1066 EG667723 Rps6kb2 Igf1r Hsf1 Stk4 Ulk1 Brd4 Atm Mknk1 Spag9 ENSMUSG00000075466 Pdpk1 Cdc2l1 Araf Riok2 Pgk1 Egf Atp5g3 Psen1 Cd74 Csk Pctk1 Nrk Camk4 Cdk4 Ppa2 Inpp5a Prkaa1 Ptk2 Cdc25c Stk17b Pgk1 Fyn Tes Vrk1 Trim28 Lck Prkcd Akt2 Ppm1b Pan3 Csnk1d Ppp3ca Pkn1 Ptpn4 Adam10 Vrk2 Zap70 Txk Atp6v0b Prkce Mvd Pak4 Atp5b Nme1 Prkcq Trib3 Atp6v0d2 Nek4 Aurkb Rps6ka1 Mtmr12 Clk1 Plk3 |
| ribosome biogenesis | GO:0042254 | 0.029 | Krr1 Ftsj1 Exosc3 Wdsof1 Npm1 Nhp2l1 Ddx51 Ipo9 Ftsj3 Exosc7 Pa2g4 Exosc1 Bop1 Ipo4 Pdcd11 Nola1 Rpl24 |
| cell cycle phase | GO:0022403 | 0.030 | Dbf4 Cdc25c Rb1 Dnajc2 Siah1a Ncapd3 Nfatc1 Cdc23 Ran Pcnt Dclre1a Txnl4a Ppp3ca Incenp Smc3 Rpl24 Mapre2 Pols Zc3hc1 Anapc1 Tubb5 Anp32b Topbp1 Brca2 Zfp655 Itgb1 Zwint Rad50 Myh9 Sin3a Nek4 BC028454 Aurkb Tacc2 Fanca |
| ribonucleoprotein complex biogenesis | GO:0022613 | 0.036 | Krr1 Exosc3 Ftsj1 Wdsof1 Npm1 Nhp2l1 Ddx51 Dicer1 Ipo9 Ftsj3 Exosc7 Pa2g4 Exosc1 Bop1 Ipo4 Pdcd11 Eif3s10 Nola1 Rpl24 |
| nucleobase, nucleoside, nucleotide and nucleic acid transport | GO:0015931 | 0.050 | Nup214 Ddx25 Eny2 Npm1 Thoc7 Pom121 Bat1a Hnrnpa2b1 Slc25a4 Nxt1 Dep1 Hnrnpa1 Nup62 |
| protein complex assembly | GO:0006461 | 0.059 | Ptk2 Wasl Elp2 Xpo6 Tbca Tbce Tes Ipo9 Calr Igf1r Tuba4a Ipo4 Spag9 Atpaf1 Kpnb1 Cse1l Tubb5 Ncoa6 Npm1 Ipo13 1110034A24Rik Tnpo1 Cd74 Ipo5 Diap1 Ipo7 |
| protein complex biogenesis | GO:0070271 | 0.059 | Ptk2 Wasl Elp2 Xpo6 Tbca Tbce Tes Ipo9 Calr Igf1r Tuba4a Ipo4 Spag9 Atpaf1 Kpnb1 Cse1l Tubb5 Ncoa6 Npm1 Ipo13 1110034A24Rik Tnpo1 Cd74 Ipo5 Diap1 Ipo7 |
| protein amino acid phosphorylation | GO:0006468 | 0.059 | Mknk2 C130006E23 Pink1 Pim2 Spnb2 Rps6kb1 Scyl3 Ptprc Rps6kb2 Igf1r Hsf1 Ulk1 Stk4 Brd4 Atm Mknk1 Spag9 ENSMUSG00000075466 Pdpk1 Cdc2l1 Araf Egf Riok2 Psen1 Cd74 Csk Pctk1 Nrk Camk4 Cdk4 Prkaa1 Ptk2 Stk17b Tes Fyn Trim28 Vrk1 Lck Prkcd Akt2 Pan3 Csnk1d Pkn1 Adam10 Vrk2 Zap70 Txk Prkce Pak4 Prkcq Trib3 Nek4 Rps6ka1 Aurkb Clk1 Plk3 |
| nucleic acid transport | GO:0050657 | 0.060 | Nup214 Hnrnpa2b1 Ddx25 Eny2 Thoc7 Npm1 Dep1 Nxt1 Pom121 Hnrnpa1 Nup62 Bat1a |
| RNA transport | GO:0050658 | 0.060 | Nup214 Hnrnpa2b1 Ddx25 Eny2 Thoc7 Npm1 Dep1 Nxt1 Pom121 Hnrnpa1 Nup62 Bat1a |
| establishment of RNA localization | GO:0051236 | 0.060 | Nup214 Hnrnpa2b1 Ddx25 Eny2 Thoc7 Npm1 Dep1 Nxt1 Pom121 Hnrnpa1 Nup62 Bat1a |
| protein amino acid autophosphorylation | GO:0046777 | 0.060 | Lck Vrk2 Zap70 Ptk2 Igf1r Ulk1 Stk4 Tes Fyn Clk1 Trim28 Vrk1 |
| macromolecular complex assembly | GO:0065003 | 0.060 | Ptk2 Wasl Xpo6 Elp2 Tbca Tbce H2afy Tes Hist1h2ae Ipo9 Dicer1 Calr Igf1r Tuba4a Ipo4 Nap1l1 Rpl24 Atpaf1 Spag9 Kpnb1 Myst4 Cse1l Tubb5 Npm1 Ncoa6 Ipo13 Smarca2 1110034A24Rik Tnpo1 Cd74 Ipo5 Diap1 Eif3s10 Ipo7 |
| RNA localization | GO:0006403 | 0.065 | Nup214 Hnrnpa2b1 Ddx25 Eny2 Thoc7 Npm1 Dep1 Nxt1 Pom121 Hnrnpa1 Nup62 Bat1a |
| macromolecular complex subunit organization | GO:0043933 | 0.064 | Ptk2 Wasl Xpo6 Elp2 Abcg1 Tbca Tbce H2afy Tes Hist1h2ae Ipo9 Dicer1 Calr Igf1r Tuba4a Ipo4 Nap1l1 Rpl24 Atpaf1 Spag9 Kpnb1 Myst4 Cse1l Tubb5 Npm1 Ncoa6 Ipo13 Smarca2 1110034A24Rik Tnpo1 Cd74 Xrn2 Ipo5 Diap1 Eif3s10 Ipo7 |
| cellular response to stress | GO:0033554 | 0.064 | Ddb1 Apex2 Trex1 Harbi1 Fanci Dhx9 Hmgb2 Hnrpll Dclre1a Rad23a Myd116 Fancc Trp53 Txnrd1 Atm Smc3 Hspa5 Mms19 Gtf2h1 Col4a3bp Wrn Rad52 D3Ucla1 Topbp1 Brca2 Uimc1 Mif Rtel1 Obfc2a Rad50 Psen1 Xrn2 Nrk Ccdc47 Smc6 Trpc2 Fanca Ctsd Prdx3 |
| DNA repair | GO:0006281 | 0.076 | Ddb1 Apex2 Trex1 Fanci Hmgb2 Dclre1a Rad23a Fancc Trp53 Smc3 Atm Wrn Gtf2h1 Mms19 Rad52 Topbp1 Brca2 Uimc1 Rtel1 Obfc2a Rad50 Xrn2 Smc6 Fanca Trpc2 |
| ubiquitin-dependent protein catabolic process | GO:0006511 | 0.128 | Uchl5 Edem1 Hsp90b1 Usp3 Siah1a Foxred2 Usp12 Ube4a Smurf1 Rad23a Usp52 Tbl1x Psmb5 Psma7 Usp47 Psma6 Usp4 Med20 |
| rRNA processing | GO:0006364 | 0.129 | Krr1 Ftsj1 Exosc3 Ftsj3 Exosc7 Pa2g4 Exosc1 Wdsof1 Bop1 Pdcd11 Ddx51 Nola1 |
| cell cycle process | GO:0022402 | 0.133 | Dbf4 Cdc25c Rb1 Dnajc2 Siah1a Ncapd3 Nfatc1 Cdc23 Ran Pcnt Dclre1a Txnl4a Ppp3ca Incenp Smc3 Rpl24 Mapre2 Pols Zc3hc1 Anapc1 Tubb5 Anp32b Npm1 Topbp1 Brca2 Zfp655 Itgb1 Zwint Rad50 Myh9 Sin3a Nek4 BC028454 Aurkb Tacc2 Fanca Macf1 |
| rRNA metabolic process | GO:0016072 | 0.138 | Krr1 Ftsj1 Exosc3 Ftsj3 Exosc7 Pa2g4 Exosc1 Wdsof1 Bop1 Pdcd11 Ddx51 Nola1 |
| chromosome organization | GO:0051276 | 0.265 | Fbxl10 Phf21a Rb1 Rnf20 Sap18 Brd8 H2afy Acin1 Ncapd3 Hist1h2ae Yeats4 Cbx6 Hnrpll Jmjd2b Phf15 Trp53 Actl6a Nap1l1 Smc3 Myst4 Cbx1 Dnmt3b Wrn Chd1 Eny2 Smarce1 Smarca2 Irf4 Brca2 Uimc1 Cdyl Rtel1 Aof1 Rbbp7 BC028454 Jarid1c |
| mRNA transport | GO:0051028 | 0.298 | Nup214 Ddx25 Eny2 Thoc7 Dep1 Nxt1 Pom121 Hnrnpa1 Nup62 Bat1a |
| microtubule-based process | GO:0007017 | 0.303 | Kifap3 Ptk2 Tbce Tubb5 Kif21b Npm1 Tes Kif3c Ktn1 Pcnt Pex1 Actr10 Htt Kif20a Xrn2 Klc1 Myh9 Tuba4a Uxt Tacc2 Kif15 Smc3 |
| positive regulation of T cell activation | GO:0050870 | 0.343 | Prkcq Lck Malt1 Zap70 Cd74 Hsp90aa1 Pdcd1lg2 Hspd1 Foxp3 Ptprc Hsp90aa1 |
| protein folding | GO:0006457 | 0.381 | Cct6b Hsp90b1 Hspa9 Cct2 Pin1 Tbce Grpel2 Ppia Hsp90aa1 Tcp1 Calr Cd74 Hsp90aa1 Hspd1 Uxt Hsp90ab1 |
| response to DNA damage stimulus | GO:0006974 | 0.378 | Ddb1 Apex2 Trex1 Fanci Hmgb2 Dclre1a Rad23a Fancc Trp53 Smc3 Atm Wrn Gtf2h1 Mms19 Rad52 Topbp1 Brca2 Uimc1 Rtel1 Obfc2a Mif Psen1 Rad50 Xrn2 Smc6 Trpc2 Fanca |
| mitotic cell cycle | GO:0000278 | 0.380 | Dbf4 Pols Anapc1 Zc3hc1 Cdc25c Rb1 Dnajc2 Anp32b Ncapd3 Nfatc1 Cdc23 Ran Itgb1 Dclre1a Zwint Nek4 Sin3a Txnl4a Ppp3ca Aurkb Incenp Smc3 Rpl24 Mapre2 |
| cell death | GO:0008219 | 0.388 | Bag5 Stk17b Bnip3l Siah1a Trp53bp2 Acin1 Traf1 Pim2 Gspt1 Xiap Tnfrsf1b Cyfip2 Lck Htt Serpinb9b Myd116 Dpf2 Ube2z Shisa5 Zc3h12a Sharpin Trp53 Stk4 Nup62 Atm Tnfrsf18 Bnip2 Itm2b Fig4 Cdc2l1 Malt1 Bfar Pdcd4 Ebag9 Psen1 Trib3 Relt Asah2 Api5 Ccar1 Fastkd5 Pdcd11 |
| hemopoiesis | GO:0030097 | 0.440 | Stap1 Zap70 Rb1 Eif4e3 Ncoa6 Acin1 Cd300lf Rbpj Vegfa Runx1 Ptprc Irf4 Brca2 Lck Malt1 Psen1 Add1 Cd74 Myh9 Foxp3 Trp53 Myo1e Prdx3 Tiparp |
| negative regulation of cellular component organization | GO:0051129 | 0.442 | Psen1 Mid1 Ptk2 Ttc3 Rdx Foxp3 Ulk1 Pacsin1 Spnb2 Pacsin2 Recql4 Atm |
| in utero embryonic development | GO:0001701 | 0.444 | Nmt1 Ints1 Runx1 Maff Lef1 Arnt2 Add1 Btf3 Trp53 Hsf1 Adam10 Psmc3 Tpm1 Col4a3bp RP23 Ncoa6 Eno1 Grn Brca2 Cdc2l1 Itgb1 Myh9 Sin3a Myo1e ENSMUSG00000072684 |
| biopolymer methylation | GO:0043414 | 0.448 | Prmt3 Mettl3 Tgs1 Ftsj1 Dnmt3b Mbd1 Aof1 Ftsj3 Ilf3 Gspt1 |
| death | GO:0016265 | 0.445 | Bag5 Stk17b Bnip3l Siah1a Trp53bp2 Acin1 Traf1 Pim2 Gspt1 Xiap Tnfrsf1b Cyfip2 Lck Htt Serpinb9b Myd116 Dpf2 Ube2z Shisa5 Zc3h12a Sharpin Trp53 Stk4 Nup62 Atm Tnfrsf18 Bnip2 Itm2b Fig4 Cdc2l1 Malt1 Bfar Pdcd4 Ebag9 Psen1 Trib3 Relt Asah2 Api5 Ccar1 Fastkd5 Pdcd11 |
| regulation of T cell activation | GO:0050863 | 0.441 | Zap70 Pdcd1lg2 Hsp90aa1 Ptprc Prkcq Lck Malt1 Cd74 Csk Hsp90aa1 Hspd1 Foxp3 Lag3 Nfkbid |
| negative regulation of signal transduction | GO:0009968 | 0.450 | Dgkz Socs6 ENSMUSG00000075466 Hspa5 Pak1ip1 Tax1bp3 Trim33 Ptprc Socs1 Prkcd Sufu Igf1r A130054J05Rik Trp53 Rgs3 Nup62 Rgs10 Nfkbid |
| chromatin organization | GO:0006325 | 0.468 | Fbxl10 Phf21a Rnf20 Rb1 Sap18 Brd8 H2afy Hist1h2ae Cbx6 Yeats4 Hnrpll Jmjd2b Phf15 Actl6a Nap1l1 Myst4 Cbx1 Dnmt3b Chd1 Eny2 Smarce1 Smarca2 Irf4 Uimc1 Cdyl Aof1 Rbbp7 Jarid1c |
| M phase | GO:0000279 | 0.478 | Cdc25c Siah1a Ncapd3 Cdc23 Ran Pcnt Dclre1a Txnl4a Incenp Smc3 Rpl24 Mapre2 Pols Anapc1 Zc3hc1 Tubb5 Topbp1 Brca2 Zwint Rad50 Myh9 Nek4 BC028454 Aurkb Tacc2 Fanca |
| posttranscriptional regulation of gene expression | GO:0010608 | 0.479 | Cdk4 Snd1 Gipc1 Mknk2 Ddx25 D3Ucla1 Eif4e2 Vegfa Tnfrsf1b Dicer1 Ipo9 Myd116 Gatc Pa2g4 Dep1 Mknk1 |
| positive regulation of immune system process | GO:0002684 | 0.499 | Fcer2a Zap70 Eif4e3 Pdcd1lg2 Ptprc Hsp90aa1 Prkcq Lck Malt1 Psen1 Cd74 Hsp90aa1 B2m Il27ra Hspd1 Foxp3 Cd247 H2 Lag3 Polr3d Pldn |
| regulation of cell cycle process | GO:0010564 | 0.497 | Dbf4 Cdc2l1 Hectd3 Calr Mycbp2 Npm1 Cdc23 Recql4 Atm Brca2 |
| regulation of lymphocyte activation | GO:0051249 | 0.567 | Zap70 Pdcd1lg2 Ptprc Hsp90aa1 Prkcq Lck Malt1 Cd74 Csk Hsp90aa1 Il27ra Hspd1 Foxp3 Lag3 Nfkbid Pldn |
| programmed cell death | GO:0012501 | 0.580 | Bag5 Stk17b Bnip3l Siah1a Trp53bp2 Traf1 Pim2 Acin1 Gspt1 Xiap Cyfip2 Lck Htt Myd116 Dpf2 Ube2z Shisa5 Zc3h12a Sharpin Trp53 Stk4 Atm Tnfrsf18 Bnip2 Itm2b Cdc2l1 Malt1 Bfar Pdcd4 Ebag9 Psen1 Trib3 Relt Asah2 Api5 Ccar1 Fastkd5 Pdcd11 |
| negative regulation of catalytic activity | GO:0043086 | 0.576 | ENSMUSG00000075466 Cdk5rap1 Spred1 Psen1 Pdcd4 Trib3 Gabbr1 Rb1 Ints1 Xiap Nup62 Ptprc |
| chromatin modification | GO:0016568 | 0.573 | Myst4 Fbxl10 Dnmt3b Phf21a Rnf20 Rb1 Chd1 Sap18 Eny2 H2afy Brd8 Smarce1 Irf4 Yeats4 Cbx6 Uimc1 Jmjd2b Phf15 Aof1 Rbbp7 Actl6a Jarid1c |
| RNA biosynthetic process | GO:0032774 | 0.593 | Stat3 Gtf2h1 Elp2 Ccdc111 Med24 Supt3h Ncoa6 Polr2e Polr3h Nfat5 Xrn2 Polr2a Trp53 Polr3d |
| methylation | GO:0032259 | 0.612 | Prmt3 Mettl3 Tgs1 Ftsj1 Dnmt3b Mbd1 Aof1 Ftsj3 Ilf3 Gspt1 |
| positive regulation of lymphocyte activation | GO:0051251 | 0.622 | Prkcq Lck Malt1 Zap70 Cd74 Hsp90aa1 Pdcd1lg2 Hspd1 Foxp3 Ptprc Hsp90aa1 Pldn |
| protein modification by small protein conjugation or removal | GO:0070647 | 0.624 | Rbbp6 Uimc1 Usp12 Smurf1 Ube4a Mgrn1 Pias3 Trim33 Siah1a Mib2 Rnf41 Gspt1 |
| apoptosis | GO:0006915 | 0.642 | Bag5 Stk17b Bnip3l Siah1a Trp53bp2 Traf1 Pim2 Acin1 Gspt1 Xiap Cyfip2 Lck Htt Myd116 Dpf2 Ube2z Shisa5 Zc3h12a Sharpin Trp53 Stk4 Atm Tnfrsf18 Bnip2 Itm2b Cdc2l1 Malt1 Bfar Pdcd4 Ebag9 Psen1 Trib3 Asah2 Api5 Ccar1 Pdcd11 Fastkd5 |
| negative regulation of cell communication | GO:0010648 | 0.642 | Dgkz Socs6 ENSMUSG00000075466 Hspa5 Pak1ip1 Tax1bp3 Trim33 Ptprc Socs1 Prkcd Sufu Igf1r A130054J05Rik Trp53 Rgs3 Nup62 Rgs10 Nfkbid |
| protein modification by small protein conjugation | GO:0032446 | 0.643 | Rbbp6 Smurf1 Ube4a Mgrn1 Pias3 Trim33 Siah1a Mib2 Rnf41 Gspt1 |
| negative regulation of molecular function | GO:0044092 | 0.644 | Cdk5rap1 ENSMUSG00000075466 Ints1 Rb1 Xiap Ptprc Spred1 Psen1 Trib3 Pdcd4 Gabbr1 Foxp3 Nup62 Nfkbid |
| embryonic development ending in birth or egg hatching | GO:0009792 | 0.660 | Nmt1 Ambra1 Ints1 Trp53bp2 Runx1 Maff Col11a1 Sufu Lef1 Htt Arnt2 Btf3 Add1 Trp53 Hsf1 Atm Adam10 Psmc3 Tpm1 Col4a3bp RP23 Ncoa6 Eno1 Grn Brca2 Cdc2l1 Itgb1 Psen1 Myh9 Sin3a Myo1e ENSMUSG00000072684 Tcf7 Ptch1 |
| purine nucleoside triphosphate biosynthetic process | GO:0009145 | 0.665 | Atp6v1a Atp5g3 Atp1b3 Atp6v0d2 Atp6v0b Atp2a2 Atp11c Atp13a1 Atp5b A430108C13Rik Nme1 |
| purine ribonucleotide biosynthetic process | GO:0009152 | 0.662 | Paics Atic Atp6v1a Atp5g3 Atp1b3 Atp6v0d2 Atp6v0b Atp2a2 Atp11c Atp13a1 Atp5b Nme1 |
| regulation of leukocyte activation | GO:0002694 | 0.675 | Zap70 Pdcd1lg2 Ptprc Hsp90aa1 Prkcq Lck Malt1 Cd74 Csk Hsp90aa1 Il27ra Hspd1 Foxp3 Lag3 Nfkbid Pldn |
| transcription | GO:0006350 | 0.675 | Pias3 Zfp472 Phf21a Rb1 Yeats4 Tshz2 Sin3b Lef1 Jmjd2b Dpf2 Hsf1 Cdc5l Anp32a Mms19 Rbpj RP23 Rfx3 Zfp27 Arhgap22 Ccar1 Jarid1c Rdbp Ets1 Mbd1 Zfp326 Wasl Brd8 Bach2 Hivep1 Trim28 Cbx6 Polr3h Maml1 Tbl1x Foxp3 Tcerg1 Per1 Ppp1r10 Med20 Myst4 Gtf2h1 Ncoa6 Ncoa7 Irf4 Xrn2 A130054J05Rik Polr2a Rbbp7 Irf5 ENSMUSG00000072684 Sfrs5 Tcf7 Gtf3c4 Snd1 Bzw1 Eif4e3 Elp2 Sap18 Clock Nfatc1 Zfp59 Runx1 Hdgf Ikbkap Tgs1 Trp53 Pa2g4 Nfe2l3 Actl6a Gtf3c2 Stat3 Zfp212 Mycbp2 Supt3h Cggbp1 Polr2e Zfp62 Uimc1 Nfat5 Cdyl Atf5 Mkl1 Zfp677 Med4 Cnot4 Ubtf Fbxl10 Foxm1 Tle4 Xbp1 Hnrpdl Mcm6 Cux1 Maff Hnrpab Arnt2 Btf3 Zfp422 Arid5a Cebpz Polr3d Bcl9l Trim33 Cc2d1a 9130019O22Rik Med24 Eny2 Ilf3 Stat1 Zfp58 Trib3 Actr5 Sin3a Ccnl1 Ewsr1 Psip1 Cep290 |
| nucleoside triphosphate biosynthetic process | GO:0009142 | 0.672 | Atp6v1a Atp5g3 Atp1b3 Atp6v0d2 Atp6v0b Atp2a2 Atp11c Atp13a1 Atp5b A430108C13Rik Nme1 |
| positive regulation of leukocyte activation | GO:0002696 | 0.672 | Prkcq Lck Malt1 Zap70 Cd74 Hsp90aa1 Pdcd1lg2 Hspd1 Foxp3 Ptprc Hsp90aa1 Pldn |
| negative regulation of macromolecule metabolic process | GO:0010605 | 0.674 | Snd1 Tle4 Phf21a Rb1 Eif4e3 Bnip3l Hivep1 Cux1 Ptprc Dicer1 Trim28 Sin3b Hnrpab Lef1 Sufu Prkcd Tbl1x Pa2g4 Trp53 Foxp3 Per1 Gipc1 ENSMUSG00000075466 Myst4 Cbx1 Dnmt3b RP23 Ddx20 Npm1 Rbpj Brca2 Socs1 Uimc1 Rfx3 Pdcd4 Psen1 Sin3a Rbbp7 Lag3 |
| hemopoietic or lymphoid organ development | GO:0048534 | 0.690 | Stap1 Zap70 Rb1 Eif4e3 Ncoa6 Acin1 Cd300lf Rbpj Vegfa Runx1 Ptprc Irf4 Brca2 Lck Malt1 Psen1 Add1 Cd74 Myh9 Foxp3 Trp53 Myo1e Prdx3 Tiparp |
| regulation of cell activation | GO:0050865 | 0.691 | Zap70 Pdcd1lg2 Ptprc Hsp90aa1 Prkcq Lck Malt1 Cd74 Csk Hsp90aa1 Il27ra Hspd1 Foxp3 Lag3 Nfkbid Pldn |
| transmembrane transport | GO:0055085 | 0.691 | Atp6v1a Pex14 Nup214 Slc9a9 Timm23 Slc25a15 Abcc5 Timm17b Grpel2 Pom121 Abcb8 1700034H14Rik Timm13 Slc25a39 Slc9a1 Nup62 Slc23a2 Slc39a14 Slc25a26 Abcb1a Slc23a1 Atp6v0b Slc19a2 D3Ucla1 2210010L05Rik Atp5b Slc30a4 Slc35b4 Atp5g3 Atp6v0d2 Slc25a4 Tpcn2 D2Ertd127e Trpc2 Clcn4 Ucp2 |
| positive regulation of cell activation | GO:0050867 | 0.698 | Prkcq Lck Malt1 Zap70 Cd74 Hsp90aa1 Pdcd1lg2 Hspd1 Foxp3 Ptprc Hsp90aa1 Pldn |
| myeloid cell differentiation | GO:0030099 | 0.698 | Stap1 Psen1 Add1 Rb1 Myh9 Acin1 Ncoa6 Cd300lf Vegfa Prdx3 Irf4 |
| transcription, DNA-dependent | GO:0006351 | 0.703 | Stat3 Gtf2h1 Elp2 Supt3h Med24 Ncoa6 Polr2e Polr3h Nfat5 Xrn2 Polr2a Trp53 Polr3d |
| ribonucleotide biosynthetic process | GO:0009260 | 0.707 | Paics Atic Atp6v1a Atp5g3 Atp1b3 Atp6v0d2 Atp6v0b Atp2a2 Atp11c Atp13a1 Atp5b Nme1 |
| chordate embryonic development | GO:0043009 | 0.723 | Nmt1 Ambra1 Ints1 Runx1 Maff Col11a1 Sufu Lef1 Htt Arnt2 Btf3 Add1 Trp53 Hsf1 Atm Adam10 Psmc3 Tpm1 Col4a3bp RP23 Ncoa6 Eno1 Grn Brca2 Cdc2l1 Itgb1 Psen1 Myh9 Sin3a Myo1e ENSMUSG00000072684 Tcf7 Ptch1 |
| glucose metabolic process | GO:0006006 | 0.734 | Prkaa1 Pfkp Pdhb Eno1 D3Ucla1 Pgk1 Pdha1 Phka2 Aldoa Pgk1 Pgam1 Ptges3 Ogdh Pck2 Pkm2 |
| hexose metabolic process | GO:0019318 | 0.748 | Prkaa1 Renbp Pfkp Pdhb Eno1 D3Ucla1 Pgk1 Pdha1 Phka2 Aldoa Pgk1 Pgam1 Ptges3 Mpi Ogdh Pck2 Pkm2 |
| tRNA metabolic process | GO:0006399 | 0.752 | Elac2 Cdk5rap1 Nars Tpr Rpp30 Farsa Rars Btbd14b Nsun2 Kars Hars Gars |
| one-carbon metabolic process | GO:0006730 | 0.767 | Prmt3 Mat2a Mettl3 Tgs1 Ftsj1 Dnmt3b Mbd1 Aof1 Ftsj3 Car7 Ilf3 Gspt1 |
| regulation of small GTPase mediated signal transduction | GO:0051056 | 0.770 | Csf1 Dgkz Tbc1d20 Rabgap1l Tbc1d1 Rapgef1 Centa1 Ralgds Ddef1 Pscd3 Rgl2 Hrb Tiam1 Rasa1 Rgnef Pscd2 Kras A130054J05Rik Nup62 Garnl1 |
| regulation of apoptosis | GO:0042981 | 0.774 | Ndufs3 Ambra1 Ints1 Stk17b Bnip3l Trp53bp2 Pim2 Traf1 Gspt1 Xiap Ptprc Lck Htt Prkcd Kras Shisa5 Trp53 Dpm1 Nup62 Atm ENSMUSG00000075466 Zc3hc1 Wrn Ddx20 Bcl2a1b Itm2b Vegfa Brca2 1110007C09Rik Malt1 Rasa1 Bfar Psen1 Atf5 Cd74 Mkl1 Api5 Sin3a Ccar1 Tcf7 Nfkbid |
| regulation of cellular protein metabolic process | GO:0032268 | 0.772 | Cdk4 Gipc1 ENSMUSG00000075466 Mknk2 Pias3 RP23 Ddx25 Prkce D3Ucla1 Eif4e2 Ptprc Socs1 Egf Prkcd Myd116 Psen1 Pdcd4 Gatc Foxp3 Trp53 Pa2g4 Dep1 Mknk1 |
| anti-apoptosis | GO:0006916 | 0.770 | Zc3hc1 Htt Bfar Atf5 Mkl1 Api5 Bnip3l Pim2 Xiap Vegfa |
| T cell activation | GO:0042110 | 0.768 | Lck Malt1 Psen1 Zap70 Cd74 Hspd1 Myh9 Trp53 Foxp3 Ccnd3 Fyn Ptprc |
| generation of precursor metabolites and energy | GO:0006091 | 0.772 | Ndufs3 Atp6v1a Pfkp Ndufs6 Pdhb Atp6v0b Pgk1 Eno1 Pdha1 Atp5b Cs Phka2 Aldoa Pgk1 Atp5g3 Pgam1 Ptges3 Atp6v0d2 Ogdh Ndufv2 Dlst Fdx1l Pkm2 |
| immune system development | GO:0002520 | 0.792 | Stap1 Zap70 Rb1 Eif4e3 Ncoa6 Acin1 Cd300lf Rbpj Vegfa Runx1 Ptprc Irf4 Brca2 Lck Malt1 Psen1 Add1 Cd74 Myh9 Foxp3 Trp53 Myo1e Prdx3 Tiparp |
| purine ribonucleotide metabolic process | GO:0009150 | 0.793 | Paics Atic Atp6v1a Atp5g3 Atp1b3 Atp6v0d2 Atp6v0b Atp2a2 Atp11c Atp13a1 Atp5b Nme1 |
| positive regulation of immune response | GO:0050778 | 0.792 | Fcer2a Zap70 Ptprc Lck Malt1 Psen1 Il27ra B2m Foxp3 Cd247 H2 Polr3d Lag3 |
| protein amino acid glycosylation | GO:0006486 | 0.791 | St3gal4 Galnt1 Psen1 Galnt2 Ddost D3Ucla1 Dpm1 Alg8 Ogt B3gnt2 |
| glycosylation | GO:0070085 | 0.791 | St3gal4 Galnt1 Psen1 Galnt2 Ddost D3Ucla1 Dpm1 Alg8 Ogt B3gnt2 |
| biopolymer glycosylation | GO:0043413 | 0.791 | St3gal4 Galnt1 Psen1 Galnt2 Ddost D3Ucla1 Dpm1 Alg8 Ogt B3gnt2 |
| cell proliferation | GO:0008283 | 0.789 | Cse1l Col4a3bp Ints1 Fyn Vegfa Tnfrsf1b Ptprc Brca2 Appl1 Prkcd Malt1 Psen1 Cd74 Ptges3 Asah2 Trp53 Bop1 Ccnd3 Tacc2 Txnrd1 Ptch1 |
| purine nucleoside triphosphate metabolic process | GO:0009144 | 0.794 | Atp6v1a Atp5g3 Atp1b3 Atp6v0d2 Atp6v0b Atp2a2 Atp11c Atp13a1 Atp5b A430108C13Rik Nme1 |
| regulation of programmed cell death | GO:0043067 | 0.794 | Ndufs3 Ambra1 Ints1 Stk17b Bnip3l Trp53bp2 Pim2 Traf1 Gspt1 Xiap Ptprc Lck Htt Prkcd Kras Shisa5 Trp53 Dpm1 Nup62 Atm ENSMUSG00000075466 Zc3hc1 Wrn Ddx20 Bcl2a1b Itm2b Vegfa Brca2 1110007C09Rik Malt1 Rasa1 Bfar Psen1 Atf5 Cd74 Mkl1 Api5 Sin3a Ccar1 Tcf7 Nfkbid |
| protein kinase cascade | GO:0007243 | 0.812 | Spag9 ENSMUSG00000075466 Stat3 Mknk2 C130006E23 Stk17b Pink1 Rps6kb1 Ptprc Socs1 Egf Malt1 Spred1 Rps6kb2 Psen1 Cd74 Nrk Rps6ka1 Slc9a1 Mknk1 |
| regulation of cell death | GO:0010941 | 0.812 | Ndufs3 Ambra1 Ints1 Stk17b Bnip3l Trp53bp2 Pim2 Traf1 Gspt1 Xiap Ptprc Lck Htt Prkcd Kras Shisa5 Trp53 Dpm1 Nup62 Atm ENSMUSG00000075466 Zc3hc1 Wrn Ddx20 Bcl2a1b Itm2b Vegfa Brca2 1110007C09Rik Malt1 Rasa1 Bfar Psen1 Atf5 Cd74 Mkl1 Api5 Sin3a Ccar1 Tcf7 Nfkbid |
| purine ribonucleoside triphosphate biosynthetic process | GO:0009206 | 0.811 | Atp6v1a Atp5g3 Atp1b3 Atp6v0d2 Atp6v0b Atp2a2 Atp11c Atp13a1 Atp5b Nme1 |
| ribonucleoside triphosphate biosynthetic process | GO:0009201 | 0.811 | Atp6v1a Atp5g3 Atp1b3 Atp6v0d2 Atp6v0b Atp2a2 Atp11c Atp13a1 Atp5b Nme1 |
| homeostatic process | GO:0042592 | 0.825 | Socs6 Slc9a9 Hrc Rb1 Acin1 Fyn Serinc5 Ptprc Lck Htt Add1 Fancc Trp53 Foxp3 Ppp3ca Dep1 Slc9a1 Txnrd1 Csf1 ENSMUSG00000075466 Stat3 Col4a3bp Wrn RP23 Ldlrap1 Atp2a2 Npm1 Vegfa Txndc16 Tpp1 Pex2 Slc30a4 Rtel1 Psen1 Nxn Ccdc47 BC028454 Trpc2 Clstn1 Gsr Prdx3 Ptch1 |
| purine nucleotide biosynthetic process | GO:0006164 | 0.839 | Atp6v1a Atp1b3 Atp6v0b Atp2a2 Atp5b A430108C13Rik Nme1 Atic Paics Atp5g3 Atp6v0d2 Atp13a1 Atp11c |
| regulation of mitotic cell cycle | GO:0007346 | 0.853 | Dbf4 Cdc2l1 Zfp655 Hectd3 Mycbp2 Trp53 Cdc23 Atm Rpl24 Brca2 |
| mitochondrion organization | GO:0007005 | 0.853 | Htt Col4a3bp Timm13 Polg Sharpin Trp53 Ssbp1 Grpel2 Pim2 Mrpl17 |
| ribonucleotide metabolic process | GO:0009259 | 0.851 | Paics Atic Atp6v1a Atp5g3 Atp1b3 Atp6v0d2 Atp6v0b Atp2a2 Atp11c Atp13a1 Atp5b Nme1 |
| regulation of organelle organization | GO:0033043 | 0.852 | Rdx Mycbp2 Npm1 Spnb2 Cdc23 Tes Cdc2l1 Hectd3 Rasa1 Mid1 Foxp3 Trp53 Recql4 Atm |
| regulation of transferase activity | GO:0051338 | 0.870 | Csf1 Dgkz Spag9 Cdk5rap1 ENSMUSG00000075466 Rb1 Npm1 Serinc5 Ptprc Egf Spred1 Psen1 Trib3 Pdcd4 Cd74 Nrk Nup62 |
| nucleoside triphosphate metabolic process | GO:0009141 | 0.872 | Atp6v1a Atp5g3 Atp1b3 Atp6v0d2 Atp6v0b Atp2a2 Atp11c Atp13a1 Atp5b A430108C13Rik Nme1 |
| leukocyte differentiation | GO:0002521 | 0.874 | Zap70 Eif4e3 Cd300lf Rbpj Ptprc Irf4 Lck Malt1 Psen1 Cd74 Myh9 Foxp3 Trp53 |
| regulation of protein kinase activity | GO:0045859 | 0.881 | Csf1 Dgkz Spag9 Cdk5rap1 ENSMUSG00000075466 Rb1 Npm1 Ptprc Egf Spred1 Psen1 Trib3 Pdcd4 Cd74 Nrk Nup62 |
| regulation of translation | GO:0006417 | 0.879 | Cdk4 Myd116 Mknk2 Gatc Ddx25 Pa2g4 D3Ucla1 Eif4e2 Dep1 Mknk1 |
| leukocyte activation | GO:0045321 | 0.877 | Zap70 Eif4e3 Rbpj Fyn Ptprc Irf4 Lck Prkcd Malt1 Psen1 Blnk Cd74 Myh9 Hspd1 Foxp3 Trp53 Ccnd3 Lcp2 |
| DNA replication | GO:0006260 | 0.887 | Dbf4 Pols Wrn Dnajc2 Polg Ccdc111 Ssbp1 Mcm6 Brca2 Orc2l Rbms1 Rbbp7 Sin3a |
| purine ribonucleoside triphosphate metabolic process | GO:0009205 | 0.886 | Atp6v1a Atp5g3 Atp1b3 Atp6v0d2 Atp6v0b Atp2a2 Atp11c Atp13a1 Atp5b Nme1 |
| purine nucleotide metabolic process | GO:0006163 | 0.886 | Atp6v1a Atp1b3 Atp6v0b Atp2a2 Atp5b A430108C13Rik Nme1 Atic Paics Atp5g3 Atp6v0d2 Pde8a Atp11c Atp13a1 |
| proteolysis | GO:0006508 | 0.887 | Edem1 Ddb1 Aup1 Mgrn1 Pias3 Usp3 Rnf20 Siah1a Rnf41 Fancc Hectd3 Smurf1 Capn1 Rad23a Pmpcb Ube2e3 Spcs3 Rnf111 Ela1 Usp47 Ube2k Anapc1 Zc3hc1 Psenen Vprbp Mycbp2 Mib2 Tpp1 Rnpep Ube3c Senp6 Psen1 Psma7 Cnot4 Usp4 Rfwd2 Socs6 Fbxl10 Foxred2 Dcun1d1 Xiap Ctsa Cdc23 Ube4a Usp52 March7 Tbl1x Ube2z Psmb5 Adam8 Map1lc3b Adam10 Med20 Rnf123 Uchl5 Hsp90b1 Atg4b Trim33 Npepps Prkcq Socs1 Usp12 Malt1 Mid1 Atg3 Myh9 Ctsd Psma6 |
| cytoskeleton organization | GO:0007010 | 0.887 | Lpin1 Ptk2 Wasl Tbce Tes Pcnt Daam2 Epb4 Calr Kras Smc3 Arhgap8 Tubb5 Npm1 Diap3 Itgb1 Lasp1 Hrb Rgnef Myh9 Diap1 Arhgap17 Uxt Svil Tacc2 |
| response to nutrient levels | GO:0031667 | 0.888 | 1110034A24Rik Psen1 Harbi1 Skiv2l Arsa Trp53 Rpl36a Ctsd Runx1 Asl Brca2 |
| microtubule-based movement | GO:0007018 | 0.889 | Actr10 Htt Kif20a Klc1 Tubb5 Tuba4a Kif21b Kif3c Kif15 Ktn1 |
| ribonucleoside triphosphate metabolic process | GO:0009199 | 0.889 | Atp6v1a Atp5g3 Atp1b3 Atp6v0d2 Atp6v0b Atp2a2 Atp11c Atp13a1 Atp5b Nme1 |
| lymphocyte activation | GO:0046649 | 0.889 | Zap70 Eif4e3 Rbpj Fyn Ptprc Lck Prkcd Malt1 Psen1 Blnk Cd74 Myh9 Hspd1 Foxp3 Trp53 Ccnd3 |
| monosaccharide metabolic process | GO:0005996 | 0.889 | Prkaa1 Renbp Pfkp Pdhb Eno1 D3Ucla1 Pgk1 Pdha1 Phka2 Aldoa Pgk1 Pgam1 Ptges3 Mpi Ogdh Pck2 Pkm2 |
| cell division | GO:0051301 | 0.895 | Cdk4 Pols Anapc1 Zc3hc1 Cdc25c Rb1 Vps4b Ncapd3 Cdc23 Ran Brca2 Cdc2l1 Dclre1a Zwint Myh9 Nek4 Txnl4a Aurkb Ccnd3 Incenp Smc3 Mapre2 |
| glycoprotein biosynthetic process | GO:0009101 | 0.902 | St3gal4 Galnt1 Psen1 Galnt2 Ddost Chst12 D3Ucla1 Dpm1 Alg8 Ogt B3gnt2 |
| regulation of cell proliferation | GO:0042127 | 0.903 | Cdk4 Odc1 Kifap3 Foxm1 Serpine1 Ambra1 Rb1 Eif4e3 Pin1 Tes Ptprc Kras Trp53 Foxp3 Hsf1 Nup62 Csf1 ENSMUSG00000075466 Zap70 Tax1bp3 Pdcd1lg2 Npm1 Rbpj Vegfa Smarca2 Grn Prkcq Egf Itgb1 Mif Atf5 Csk Gnl3 ENSMUSG00000072684 Fanca Tcf7 Ptch1 Recql4 |
| glycoprotein metabolic process | GO:0009100 | 0.905 | Galnt1 Ddost D3Ucla1 Man1a2 Alg8 Col11a1 St3gal4 Psen1 Galnt2 Dpm1 Chst12 B3gnt2 Ogt |
| regulation of kinase activity | GO:0043549 | 0.904 | Csf1 Dgkz Spag9 Cdk5rap1 ENSMUSG00000075466 Rb1 Npm1 Ptprc Egf Spred1 Psen1 Trib3 Pdcd4 Cd74 Nrk Nup62 |
| negative regulation of apoptosis | GO:0043066 | 0.911 | Zc3hc1 Ints1 Bnip3l Pim2 Xiap Vegfa Htt Bfar Rasa1 Atf5 Cd74 Mkl1 Kras Api5 Sin3a Trp53 Dpm1 Nup62 Atm |
| nucleobase, nucleoside and nucleotide biosynthetic process | GO:0034404 | 0.917 | Atp6v1a Atp1b3 Aprt Atp6v0b Atp2a2 Dtymk Atp5b A430108C13Rik Nme1 Atic Paics Atp5g3 Atp6v0d2 Atp11c Atp13a1 |
| nucleobase, nucleoside, nucleotide and nucleic acid biosynthetic process | GO:0034654 | 0.917 | Atp6v1a Atp1b3 Aprt Atp6v0b Atp2a2 Dtymk Atp5b A430108C13Rik Nme1 Atic Paics Atp5g3 Atp6v0d2 Atp11c Atp13a1 |
| M phase of mitotic cell cycle | GO:0000087 | 0.921 | Pols Anapc1 Zc3hc1 Cdc25c Ncapd3 Cdc23 Ran Dclre1a Zwint Nek4 Txnl4a Aurkb Incenp Rpl24 Smc3 Mapre2 |
| regulation of Ras protein signal transduction | GO:0046578 | 0.934 | Csf1 Dgkz Tbc1d20 Rabgap1l Tbc1d1 Centa1 Ddef1 Pscd3 Tiam1 Hrb Rgnef Pscd2 A130054J05Rik Kras Nup62 |
| positive regulation of response to stimulus | GO:0048584 | 0.934 | Fcer2a ENSMUSG00000075466 Zap70 Ptprc Uimc1 Lck Malt1 Psen1 Il27ra B2m Foxp3 Cd247 H2 Lag3 Polr3d |
| negative regulation of programmed cell death | GO:0043069 | 0.934 | Zc3hc1 Ints1 Bnip3l Pim2 Xiap Vegfa Htt Bfar Rasa1 Atf5 Cd74 Mkl1 Kras Api5 Sin3a Trp53 Dpm1 Nup62 Atm |
| negative regulation of cell death | GO:0060548 | 0.938 | Zc3hc1 Ints1 Bnip3l Pim2 Xiap Vegfa Htt Bfar Rasa1 Atf5 Cd74 Mkl1 Kras Api5 Sin3a Trp53 Dpm1 Nup62 Atm |
| negative regulation of RNA metabolic process | GO:0051253 | 0.937 | Cbx1 Dnmt3b Phf21a Tle4 Rb1 Eif4e3 Ddx20 Npm1 Rbpj Hivep1 Cux1 Trim28 Sin3b Hnrpab Sufu Lef1 Tbl1x Sin3a Foxp3 Trp53 Pa2g4 Rbbp7 Per1 |
| negative regulation of gene expression | GO:0010629 | 0.951 | Snd1 Tle4 Phf21a Rb1 Eif4e3 Bnip3l Hivep1 Cux1 Trim28 Dicer1 Sin3b Sufu Lef1 Hnrpab Tbl1x Pa2g4 Trp53 Foxp3 Per1 Myst4 Cbx1 Dnmt3b Ddx20 Rbpj Uimc1 Rfx3 Pdcd4 Sin3a Rbbp7 |
| negative regulation of nucleobase, nucleoside, nucleotide and nucleic acid metabolic process | GO:0045934 | 0.960 | Tle4 Phf21a Eif4e3 Rb1 Hivep1 Cux1 Trim28 Sin3b Sufu Lef1 Hnrpab Tbl1x Pa2g4 Trp53 Foxp3 Per1 Myst4 Cbx1 Dnmt3b Ddx20 Npm1 Rbpj Brca2 Uimc1 Rfx3 Pdcd4 Sin3a Rbbp7 |
| microtubule cytoskeleton organization | GO:0000226 | 0.959 | Ptk2 Myh9 Tubb5 Tbce Uxt Npm1 Tacc2 Tes Smc3 Pcnt |
| chemical homeostasis | GO:0048878 | 0.962 | Socs6 Slc9a9 Hrc Fyn Serinc5 Ptprc Lck Htt Trp53 Ppp3ca Dep1 Slc9a1 ENSMUSG00000075466 Stat3 Col4a3bp RP23 Ldlrap1 Atp2a2 Vegfa Pex2 Slc30a4 Psen1 Ccdc47 Clstn1 Trpc2 Ptch1 |
| nucleotide biosynthetic process | GO:0009165 | 0.962 | Atp6v1a Atp1b3 Atp6v0b Atp2a2 Dtymk Atp5b A430108C13Rik Nme1 Atic Paics Atp5g3 Atp6v0d2 Atp11c Atp13a1 |
| regulation of transcription | GO:0045449 | 0.962 | Zfp472 Pias3 Phf21a Rb1 Yeats4 Tshz2 Sin3b Lef1 Jmjd2b Dpf2 Hsf1 Cdc5l Anp32a Nufip1 Mms19 Rbpj RP23 Zfp27 Rfx3 Pdcd4 Arhgap22 Ccar1 Jarid1c Rdbp Nfkbid Ets1 Mbd1 Zfp326 Wasl 5830417I10Rik Brd8 Bach2 Hmgb2 Hivep1 Trim28 Cbx6 Maml1 Tbl1x Foxp3 Tcerg1 Per1 Nup62 Med20 Myst4 Dnmt3b Gtf2h1 Ncoa6 Ncoa7 Irf4 Malt1 Xrn2 A130054J05Rik Rbbp7 Sfrs5 Irf5 ENSMUSG00000072684 Tcf7 Snd1 Bzw1 Eif4e3 Elp2 Sap18 Abcg1 Clock Nfatc1 Zfp59 Runx1 Plagl1 Hdgf Ikbkap Sufu Tgs1 Pa2g4 Trp53 Tmpo Nfe2l3 Actl6a Garnl1 Stat3 Zfp212 Mycbp2 Ddx20 Cggbp1 Vegfa Zfp62 Uimc1 Zfp655 Nfat5 Cdyl Atf5 Ss18l1 Mkl1 Zfp677 Med4 Cnot4 Ubtf Fbxl10 Foxm1 Tle4 Arid2 Dnajc2 Xbp1 Hnrpdl Mcm6 Cux1 Maff Hnrpab Arnt2 Btf3 Zfp422 Pde8a Arid5a Dpm1 Cebpz Cbx1 Bcl9l Trim33 Cc2d1a Eny2 9130019O22Rik Med24 Ilf3 Stat1 Zfp58 Ddx5 Smarca2 Prkcq Trib3 Actr5 Sin3a Ccnl1 Ewsr1 Psip1 Pdcd11 Cep290 |
| nitrogen compound biosynthetic process | GO:0044271 | 0.962 | Mat2a Odc1 Atp6v1a Atp1b3 Aprt Atp6v0b Azin1 Atp2a2 Dtymk Atp5b Nme1 Hsp90aa1 A430108C13Rik Mocs2 Paics Htt Atic Atp5g3 Hsp90aa1 Atp6v0d2 Atp11c Atp13a1 Asl |
| negative regulation of nitrogen compound metabolic process | GO:0051172 | 0.965 | Tle4 Phf21a Eif4e3 Rb1 Hivep1 Cux1 Trim28 Sin3b Sufu Lef1 Hnrpab Tbl1x Pa2g4 Trp53 Foxp3 Per1 Myst4 Cbx1 Dnmt3b Ddx20 Npm1 Rbpj Brca2 Uimc1 Rfx3 Pdcd4 Sin3a Rbbp7 |
| mitosis | GO:0007067 | 0.964 | Pols Anapc1 Zc3hc1 Cdc25c Ncapd3 Cdc23 Ran Dclre1a Zwint Nek4 Txnl4a Aurkb Incenp Smc3 Mapre2 |
| nuclear division | GO:0000280 | 0.964 | Pols Anapc1 Zc3hc1 Cdc25c Ncapd3 Cdc23 Ran Dclre1a Zwint Nek4 Txnl4a Aurkb Incenp Smc3 Mapre2 |
| heart development | GO:0007507 | 0.968 | Tpm1 Col4a3bp Eif4e3 Trp53bp2 Ncoa6 Nfatc1 Rbpj Dicer1 Col11a1 Col5a1 Sin3b Sufu Itgb1 Psen1 ENSMUSG00000072684 Ptch1 Atm |
| negative regulation of transcription | GO:0016481 | 0.970 | Tle4 Phf21a Eif4e3 Rb1 Hivep1 Trim28 Cux1 Sin3b Sufu Lef1 Hnrpab Tbl1x Pa2g4 Trp53 Foxp3 Per1 Myst4 Cbx1 Dnmt3b Ddx20 Rbpj Uimc1 Rfx3 Pdcd4 Rbbp7 Sin3a |
| negative regulation of transcription, DNA-dependent | GO:0045892 | 0.972 | Cbx1 Dnmt3b Phf21a Tle4 Rb1 Eif4e3 Ddx20 Rbpj Hivep1 Cux1 Trim28 Sin3b Hnrpab Lef1 Sufu Tbl1x Sin3a Foxp3 Trp53 Pa2g4 Rbbp7 Per1 |
| cell activation | GO:0001775 | 0.972 | Zap70 Eif4e3 Rbpj Fyn Ptprc Irf4 Lck Prkcd Malt1 Psen1 Blnk Cd74 Myh9 Hspd1 Foxp3 Trp53 Ccnd3 Lcp2 |
| response to extracellular stimulus | GO:0009991 | 0.975 | 1110034A24Rik Psen1 Harbi1 Skiv2l Arsa Trp53 Rpl36a Ctsd Runx1 Asl Brca2 |
| blood vessel development | GO:0001568 | 0.976 | Foxm1 Ptk2 Rapgef1 Plxnd1 Ncoa6 Rbpj Vegfa Dicer1 Col5a1 Rasa1 Psen1 Myh9 Zc3h12a Arhgap22 Myo1e ENSMUSG00000072684 Dep1 Tiparp |
| induction of apoptosis | GO:0006917 | 0.979 | Ndufs3 ENSMUSG00000075466 Bnip3l Stk17b Trp53bp2 Ddx20 Itm2b Brca2 Lck Prkcd Shisa5 Trp53 Atm |
| induction of programmed cell death | GO:0012502 | 0.979 | Ndufs3 ENSMUSG00000075466 Bnip3l Stk17b Trp53bp2 Ddx20 Itm2b Brca2 Lck Prkcd Shisa5 Trp53 Atm |
| negative regulation of cell differentiation | GO:0045596 | 0.979 | Ptk2 Ttc3 Abcg1 Rbpj Dicer1 Itgb1 Psen1 Cd74 Jag1 Trp53 Ulk1 Nphp3 Iqcb1 Nfkbid |
| organelle fission | GO:0048285 | 0.979 | Pols Anapc1 Zc3hc1 Cdc25c Ncapd3 Cdc23 Ran Dclre1a Zwint Nek4 Txnl4a Aurkb Incenp Smc3 Mapre2 |
| positive regulation of apoptosis | GO:0043065 | 0.979 | Ndufs3 ENSMUSG00000075466 Ambra1 Bnip3l Stk17b Trp53bp2 Ddx20 Itm2b Ptprc Brca2 Lck Prkcd Psen1 Shisa5 Trp53 Ccar1 Atm Nfkbid |
| cation homeostasis | GO:0055080 | 0.980 | ENSMUSG00000075466 Slc9a9 Hrc Atp2a2 Ptprc Slc30a4 Lck Htt Psen1 Trp53 Ccdc47 Slc9a1 Clstn1 Trpc2 |
| response to abiotic stimulus | GO:0009628 | 0.981 | Mat2a Sdf4 Arsa Trp53bp2 Dhx9 Fyn Pdpk1 Brca2 Uimc1 Htt Obfc2a Kras Strbp Trp53 Hsf1 Pkn1 Slc9a1 Atm |
| positive regulation of programmed cell death | GO:0043068 | 0.982 | Ndufs3 ENSMUSG00000075466 Ambra1 Bnip3l Stk17b Trp53bp2 Ddx20 Itm2b Ptprc Brca2 Lck Prkcd Psen1 Shisa5 Trp53 Ccar1 Atm Nfkbid |
| vesicle-mediated transport | GO:0016192 | 0.982 | Ap2b1 Ap4s1 Cog3 Exoc2 Arf4 Chmp7 Vps4b Tmed10 Pacsin2 Pacsin1 Ap1s3 Cux1 Eps15l1 Htt Arf3 Ulk1 Gars Pldn Vamp1 Ldlrap1 Fnbp1 Abca7 Nme1 8030493G06Rik Vps29 Psen1 Copz1 Txlna Arhgap17 Trpc2 Vamp4 |
| vasculature development | GO:0001944 | 0.983 | Foxm1 Ptk2 Rapgef1 Plxnd1 Ncoa6 Rbpj Vegfa Dicer1 Col5a1 Rasa1 Psen1 Myh9 Zc3h12a Arhgap22 Myo1e ENSMUSG00000072684 Dep1 Tiparp |
| negative regulation of macromolecule biosynthetic process | GO:0010558 | 0.984 | Tle4 Phf21a Eif4e3 Rb1 Hivep1 Cux1 Trim28 Sin3b Sufu Lef1 Hnrpab Tbl1x Pa2g4 Trp53 Foxp3 Per1 Myst4 Cbx1 Dnmt3b Ddx20 Rbpj Brca2 Uimc1 Rfx3 Pdcd4 Rbbp7 Sin3a Lag3 |
| positive regulation of cell death | GO:0010942 | 0.984 | Ndufs3 ENSMUSG00000075466 Ambra1 Bnip3l Stk17b Trp53bp2 Ddx20 Itm2b Ptprc Brca2 Lck Prkcd Psen1 Shisa5 Trp53 Ccar1 Atm Nfkbid |
| regulation of growth | GO:0040008 | 0.987 | Ndufs3 Csf1 Socs6 Stat3 Ptk2 Wrn Dnajc2 Siah1a D3Ucla1 Brd8 Yeats4 Socs1 Trp53 Ube2e3 Actl6a Hsf1 Ulk1 Ptch1 |
| carboxylic acid biosynthetic process | GO:0046394 | 0.988 | Mat2a Rnpep Pex2 Prkaa1 Htt Cd74 Ptges3 Dep1 Asl Fancc Prkab2 |
| organic acid biosynthetic process | GO:0016053 | 0.988 | Mat2a Rnpep Pex2 Prkaa1 Htt Cd74 Ptges3 Dep1 Asl Fancc Prkab2 |
| negative regulation of cell proliferation | GO:0008285 | 0.988 | ENSMUSG00000075466 Kifap3 Tax1bp3 Ambra1 Rb1 Pdcd1lg2 Npm1 Rbpj Tes Smarca2 Csk Foxp3 Trp53 Hsf1 Nup62 Ptch1 |
| regulation of phosphorylation | GO:0042325 | 0.988 | Csf1 Dgkz Spag9 ENSMUSG00000075466 Cdk5rap1 Rb1 RP23 Prkce Npm1 Ptprc Socs1 Egf Prkcd Spred1 Psen1 Trib3 Pdcd4 Cd74 Nrk Nup62 |
| positive regulation of molecular function | GO:0044093 | 0.990 | Csf1 Dgkz Spag9 Calm1 Psenen Homer1 Npm1 Gspt1 Hmgb2 Serinc5 Ptprc Irf4 Prkcq Lck Malt1 Egf Psen1 Cd74 Trp53 Nrk Brd4 |
| positive regulation of cell differentiation | GO:0045597 | 0.991 | Csf1 Zap70 Rb1 Acin1 Runx1 Hsp90aa1 Ptprc Lck Tiam1 Cd74 Hsp90aa1 Jag1 Foxp3 Ets1 |
| small GTPase mediated signal transduction | GO:0007264 | 0.992 | Bcar3 Rras Kifap3 Gdi2 Arf4 Rapgef1 Tax1bp3 Ralgds Arhgdia Rgl2 Tiam1 Arf3 Kras Arl4a Rab37 Ulk1 A930013N22Rik Rab1b |
| actin filament-based process | GO:0030029 | 0.992 | Lpin1 Wasl Myo9b Arhgap8 Diap3 Daam2 Itgb1 Epb4 Calr Kras Myh9 Diap1 Arhgap17 |
| di-, tri-valent inorganic cation homeostasis | GO:0055066 | 0.992 | Slc30a4 Lck ENSMUSG00000075466 Htt Psen1 Hrc Ccdc47 Atp2a2 Clstn1 Trpc2 Ptprc |
| negative regulation of cellular biosynthetic process | GO:0031327 | 0.992 | Tle4 Phf21a Eif4e3 Rb1 Hivep1 Cux1 Trim28 Sin3b Sufu Lef1 Hnrpab Tbl1x Pa2g4 Trp53 Foxp3 Per1 Myst4 Cbx1 Dnmt3b Ddx20 Rbpj Brca2 Uimc1 Rfx3 Pdcd4 Rbbp7 Sin3a Lag3 |
| regulation of protein modification process | GO:0031399 | 0.992 | Socs1 Prkcd Egf ENSMUSG00000075466 Psen1 Pdcd4 Pias3 RP23 Trp53 Foxp3 Prkce Ptprc |
| gland development | GO:0048732 | 0.992 | Csf1 Plxnd1 Cd44 Xbp1 Rbpj Vegfa Tes Nme1 Brca2 Lef1 Egf Igf1r Ptch1 Gsdma3 |
| anion transport | GO:0006820 | 0.993 | Htt Psen1 Ank Slc4a8 Slc4a2 Slc20a2 Slc25a4 D2Ertd127e Clcn4 Tes |
| response to hormone stimulus | GO:0009725 | 0.993 | Mat2a Lpin1 Akt2 Stat3 Tbl1x RP23 Arsa Scap Phip Hmgb2 Tes Asl |
| phospholipid metabolic process | GO:0006644 | 0.993 | Smpd4 Cds2 Lpcat1 Pitpnc1 Gpaa1 Dpm1 Pigo D5Wsu178e Fig4 Agpat5 Serinc5 Pnpla7 |
| negative regulation of biosynthetic process | GO:0009890 | 0.993 | Tle4 Phf21a Eif4e3 Rb1 Hivep1 Cux1 Trim28 Sin3b Sufu Lef1 Hnrpab Tbl1x Pa2g4 Trp53 Foxp3 Per1 Myst4 Cbx1 Dnmt3b Ddx20 Rbpj Brca2 Uimc1 Rfx3 Pdcd4 Rbbp7 Sin3a Lag3 |
| regulation of phosphate metabolic process | GO:0019220 | 0.995 | Csf1 Dgkz Spag9 ENSMUSG00000075466 Cdk5rap1 Rb1 RP23 Prkce Npm1 Ptprc Socs1 Egf Prkcd Spred1 Psen1 Trib3 Pdcd4 Cd74 Nrk Nup62 |
| regulation of phosphorus metabolic process | GO:0051174 | 0.995 | Csf1 Dgkz Spag9 ENSMUSG00000075466 Cdk5rap1 Rb1 RP23 Prkce Npm1 Ptprc Socs1 Egf Prkcd Spred1 Psen1 Trib3 Pdcd4 Cd74 Nrk Nup62 |
| positive regulation of biosynthetic process | GO:0009891 | 0.995 | Cdk4 Foxm1 Zfp326 Rb1 Arid2 Abcg1 Clock Hmgb2 Tlr9 Runx1 Plagl1 Trim28 Hsp90aa1 Hnrpab Lef1 Maml1 Arnt2 Trp53 Foxp3 Nup62 Nufip1 Myst4 Stat3 Eny2 Npm1 D3Ucla1 Vegfa Ddx5 Irf4 Prkcq Nfat5 Hsp90aa1 Mkl1 ENSMUSG00000072684 Hsp90ab1 Ets1 |
| intracellular signaling cascade | GO:0007242 | 0.995 | Bcar3 Mknk2 Kifap3 C130006E23 Pink1 Nfatc1 Rps6kb1 Ptprc Rps6kb2 Arf3 Ptges3 Kras Trp53 Ulk1 A930013N22Rik Atm Mknk1 Dgkz Spag9 ENSMUSG00000075466 Stat3 Gdi2 Rapgef1 Arhgdia Uimc1 Araf Egf Tiam1 Mif Psen1 Cd74 Csk Arl4a Nrk Rab1b Socs6 Rras Arf4 Myo9b Stk17b Prkcd Spred1 Rab37 Slc9a1 Zap70 Tax1bp3 Ralgds Prkce Brca2 Prkcq Socs1 Rgl2 Malt1 Rgnef Rps6ka1 Cilp2 |
| blood vessel morphogenesis | GO:0048514 | 0.994 | Ptk2 Foxm1 Plxnd1 Rbpj Vegfa Dicer1 Rasa1 Zc3h12a Myh9 Arhgap22 ENSMUSG00000072684 Myo1e Dep1 Tiparp |
| actin cytoskeleton organization | GO:0030036 | 0.994 | Lpin1 Itgb1 Calr Epb4 Wasl Kras Myh9 Arhgap8 Diap1 Arhgap17 Diap3 Daam2 |
| positive regulation of transferase activity | GO:0051347 | 0.996 | Dgkz Csf1 Spag9 Egf Psen1 Cd74 Nrk Npm1 Serinc5 Ptprc |
| regulation of cytokine production | GO:0001817 | 0.997 | Prkcq Ddx58 Il27ra Hspd1 Foxp3 Hsf1 Tlr9 Lag3 Polr3d Irf4 |
| reproductive cellular process | GO:0048610 | 0.997 | Itgb1 Hrb Aof1 Fancc Strbp Arsa Ddx25 ENSMUSG00000072684 Trpc2 Rps6kb1 Hmgb2 Brca2 |
| cellular homeostasis | GO:0019725 | 0.997 | Socs6 ENSMUSG00000075466 Npm1 Atp2a2 Fyn Serinc5 Ptprc Txndc16 Pex2 Lck Htt Psen1 Add1 Nxn Trp53 Ppp3ca Dep1 Trpc2 Gsr Clstn1 Txnrd1 Prdx3 |
| cellular amino acid derivative metabolic process | GO:0006575 | 0.998 | Mat2a Hnrpll Odc1 Htt P4ha1 Oaz1 Dhps Azin1 Gsr Pnpla7 |
| ion homeostasis | GO:0050801 | 0.998 | ENSMUSG00000075466 Slc9a9 Hrc Atp2a2 Fyn Serinc5 Ptprc Pex2 Slc30a4 Lck Htt Psen1 Trp53 Ppp3ca Ccdc47 Dep1 Trpc2 Slc9a1 Clstn1 |
| positive regulation of cellular biosynthetic process | GO:0031328 | 0.998 | Cdk4 Foxm1 Zfp326 Rb1 Arid2 Clock Hmgb2 Tlr9 Runx1 Plagl1 Trim28 Hsp90aa1 Lef1 Hnrpab Maml1 Arnt2 Trp53 Foxp3 Nup62 Nufip1 Myst4 Stat3 Eny2 Npm1 D3Ucla1 Vegfa Ddx5 Irf4 Prkcq Nfat5 Hsp90aa1 Mkl1 ENSMUSG00000072684 Hsp90ab1 Ets1 |
| cellular component morphogenesis | GO:0032989 | 0.998 | Ptk2 Col4a3bp Mycbp2 Ssbp1 Tbce Nfatc1 Rbpj Pcnt Trim28 Hnrpab Itgb1 Lef1 Hrb Rgnef Add1 Myh9 Stk4 Ulk1 Top2b Rpl24 B3gnt2 Macf1 |
| cell morphogenesis involved in differentiation | GO:0000904 | 0.998 | Ptk2 Mycbp2 Tbce Nfatc1 Rbpj Trim28 Lef1 Hnrpab Rgnef Myh9 Ulk1 Top2b Rpl24 B3gnt2 |
| organophosphate metabolic process | GO:0019637 | 0.998 | Smpd4 Cds2 Lpcat1 Pitpnc1 Gpaa1 Dpm1 Pigo D5Wsu178e Fig4 Agpat5 Serinc5 Pnpla7 |
| negative regulation of transcription from RNA polymerase II promoter | GO:0000122 | 0.999 | Tle4 Phf21a Eif4e3 Rb1 Ddx20 Hivep1 Cux1 Trim28 Lef1 Sufu Sin3a Foxp3 Trp53 Rbbp7 Per1 |
| regulation of cellular component size | GO:0032535 | 0.999 | Cdk4 Ndufs3 Rasa1 Dnajc2 Xrn2 Rdx Trp53 Npm1 Ulk1 Spnb2 Slc9a1 |
| response to radiation | GO:0009314 | 0.999 | Mat2a Uimc1 Sdf4 Htt Obfc2a Kras Trp53bp2 Trp53 Atm Brca2 |
| positive regulation of nitrogen compound metabolic process | GO:0051173 | 0.999 | Foxm1 Zfp326 Rb1 Arid2 Clock Hmgb2 Runx1 Plagl1 Trim28 Hsp90aa1 Ptprc Lef1 Hnrpll Hnrpab Arnt2 Maml1 Trp53 Foxp3 Nup62 Nufip1 Myst4 Stat3 Eny2 Vegfa Ddx5 Irf4 Uimc1 Nfat5 Hsp90aa1 Mkl1 ENSMUSG00000072684 Hsp90ab1 Ets1 |
| positive regulation of cell proliferation | GO:0008284 | 0.999 | Csf1 Odc1 Foxm1 Zap70 Eif4e3 Pdcd1lg2 Npm1 Rbpj Vegfa Ptprc Grn Prkcq Egf Itgb1 Kras Foxp3 ENSMUSG00000072684 Recql4 |
| lipid biosynthetic process | GO:0008610 | 0.999 | B4galnt1 Prkaa1 Lpcat1 Mvd Agpat5 Serinc5 Fancc Pex2 Rnpep Cds2 Cd74 Ptges3 Gpaa1 Dpm1 Dep1 Pigo D5Wsu178e Prkab2 |
| immune response | GO:0006955 | 0.999 | Eif4e3 Bnip3l H2 Tlr9 Tnfrsf1b Ptprc Cnpy3 Polr3h Prkcd Ddx58 B2m Mpa2l Trp53 Foxp3 Samhd1 H2 Polr3d Tgtp Il18r1 Cd300lf Vegfa 2210010L05Rik Oas1a Malt1 H2 Mif Psen1 Cd74 OTTMUSG00000005523 |
| response to endogenous stimulus | GO:0009719 | 0.999 | Mat2a Lpin1 Akt2 Stat3 Tbl1x RP23 Arsa Scap Phip Hmgb2 Tes Asl |
| response to organic substance | GO:0010033 | 0.999 | Edem1 Mat2a Lpin1 Sdf4 Ttc3 Herpud2 Hmgb2 Tes Fyn Dicer1 Hsp90aa1 Akt2 Myd116 Tbl1x B2m Skiv2l Hsf1 Slc9a1 Stat3 RP23 Arsa D3Ucla1 Ppp2r2a Stat1 Malt1 Hsp90aa1 Scap Phip Trpc2 Asl Prdx3 |
| cell morphogenesis | GO:0000902 | 0.999 | Ptk2 Col4a3bp Mycbp2 Tbce Nfatc1 Rbpj Pcnt Trim28 Lef1 Hnrpab Rgnef Add1 Myh9 Stk4 Ulk1 Top2b Rpl24 B3gnt2 Macf1 |
| tissue morphogenesis | GO:0048729 | 0.999 | Csf1 Tpm1 Plxnd1 Cd44 Col11a1 Htt Lef1 Sufu Igf1r Jag1 Fras1 Txnrd1 Ptch1 Gsdma3 Macf1 |
| fatty acid metabolic process | GO:0006631 | 0.999 | Rnpep Prkaa1 Acot7 Crot Cd74 Ptges3 Acsl5 Dep1 Fancc Prkab2 Bdh2 Lypla2 |
| positive regulation of cell communication | GO:0010647 | 0.999 | Csf1 Gipc1 Zap70 Igf1r Kras Jag1 Pink1 Pim2 Pdcd11 Vegfa Nup62 Ptprc |
| secretion by cell | GO:0032940 | 0.999 | Gipc1 Exoc2 Htt Psen1 Tmed10 Txlna Arhgap17 Trpc2 Gars Lcp2 Erp29 Pldn |
| positive regulation of signal transduction | GO:0009967 | 0.999 | Csf1 Gipc1 Zap70 Igf1r Kras Jag1 Pim2 Pdcd11 Vegfa Nup62 Ptprc |
| sexual reproduction | GO:0019953 | 1.000 | B4galnt1 Stat3 Ddx25 Arsa Siah1a Herpud2 Hmgb2 Nhp2l1 Rps6kb1 Brca2 Hnrpll Htt Itgb1 Hrb Aof1 Fancc Xrn2 Strbp Hsf1 ENSMUSG00000072684 Dep1 Trpc2 Atm |
| regulation of protein kinase cascade | GO:0010627 | 1.000 | Socs1 Akt2 Pdcd4 Igf1r Elp2 Pim2 Pdcd11 Nup62 Ptprc Nfkbid |
| regulation of transcription from RNA polymerase II promoter | GO:0006357 | 1.000 | Foxm1 Phf21a Tle4 Rb1 Eif4e3 Arid2 Clock Hmgb2 Runx1 Hivep1 Plagl1 Cux1 Trim28 Ikbkap Sufu Lef1 Maml1 Arnt2 Tbl1x Trp53 Foxp3 Per1 Nufip1 Med20 Stat3 Ddx20 Rbpj Vegfa Irf4 Nfat5 Atf5 Sin3a Rbbp7 Med4 ENSMUSG00000072684 Ets1 |
| positive regulation of macromolecule metabolic process | GO:0010604 | 1.000 | Cdk4 Foxm1 Zfp326 Pias3 Rb1 Arid2 Clock Hmgb2 Tlr9 Runx1 Plagl1 Ptprc Trim28 Lef1 Hnrpll Hnrpab Maml1 Arnt2 Trp53 Foxp3 Nup62 Nufip1 ENSMUSG00000075466 Myst4 Stat3 Eny2 D3Ucla1 Vegfa Ddx5 Irf4 Uimc1 Prkcq Nfat5 Psen1 Mkl1 ENSMUSG00000072684 Ets1 |
| growth | GO:0040007 | 1.000 | Csf1 Psen1 Add1 Xrn2 Ints1 Trp53 Tbce ENSMUSG00000072684 Npm1 Ulk1 Slc9a1 Brca2 |
| positive regulation of nucleobase, nucleoside, nucleotide and nucleic acid metabolic process | GO:0045935 | 1.000 | Foxm1 Zfp326 Arid2 Rb1 Clock Hmgb2 Runx1 Plagl1 Trim28 Ptprc Lef1 Hnrpll Hnrpab Arnt2 Maml1 Trp53 Foxp3 Nufip1 Nup62 Myst4 Stat3 Eny2 Vegfa Ddx5 Irf4 Uimc1 Nfat5 Mkl1 ENSMUSG00000072684 Ets1 |
| positive regulation of macromolecule biosynthetic process | GO:0010557 | 1.000 | Cdk4 Foxm1 Zfp326 Arid2 Rb1 Clock Hmgb2 Tlr9 Runx1 Plagl1 Trim28 Lef1 Hnrpab Arnt2 Maml1 Trp53 Foxp3 Nufip1 Nup62 Myst4 Stat3 Eny2 D3Ucla1 Vegfa Ddx5 Irf4 Prkcq Nfat5 Mkl1 ENSMUSG00000072684 Ets1 |
| positive regulation of multicellular organismal process | GO:0051240 | 1.000 | Csf1 Tpm1 Ddx58 Psen1 Il27ra Hspd1 Pink1 Foxp3 Hsf1 Polr3d |
| tube development | GO:0035295 | 1.000 | Csf1 l7Rn6 Plxnd1 Eif4e3 Cd44 Slc23a1 Man1a2 Vegfa Tes Cux1 Dicer1 Sufu Egf Psen1 Ptges3 Ptch1 |
| regulation of hydrolase activity | GO:0051336 | 1.000 | Tbc1d20 Lck Rabgap1l Hrb Gdi2 Tbc1d1 Centa1 Ints1 Trp53 Ddef1 Gspt1 Xiap |
| positive regulation of developmental process | GO:0051094 | 1.000 | Csf1 Zap70 Rb1 Acin1 Runx1 Hsp90aa1 Ptprc Lck Tiam1 Cd74 Hsp90aa1 Jag1 Foxp3 Ets1 |
| gamete generation | GO:0007276 | 1.000 | B4galnt1 Ddx25 Siah1a Herpud2 Hmgb2 Rps6kb1 Brca2 Htt Itgb1 Hnrpll Hrb Aof1 Fancc Xrn2 Strbp Hsf1 ENSMUSG00000072684 Dep1 Atm |
| cell migration | GO:0016477 | 1.000 | Gipc1 ENSMUSG00000075466 Ptk2 Vegfa Fyn Tes Atp5b Pex2 Itgb1 Psen1 Myh9 Ulk1 Top2b Nup62 |
| multicellular organism reproduction | GO:0032504 | 1.000 | B4galnt1 Ddx25 Siah1a Herpud2 Hmgb2 Tes Vegfa Rps6kb1 Nme1 Brca2 Htt Itgb1 Hnrpll Hrb Aof1 Fancc Xrn2 Strbp Hsf1 ENSMUSG00000072684 Dep1 Trpc2 Atm |
| reproductive process in a multicellular organism | GO:0048609 | 1.000 | B4galnt1 Ddx25 Siah1a Herpud2 Hmgb2 Tes Vegfa Rps6kb1 Nme1 Brca2 Htt Itgb1 Hnrpll Hrb Aof1 Fancc Xrn2 Strbp Hsf1 ENSMUSG00000072684 Dep1 Trpc2 Atm |
| positive regulation of transcription | GO:0045941 | 1.000 | Foxm1 Zfp326 Arid2 Rb1 Clock Hmgb2 Runx1 Plagl1 Trim28 Lef1 Hnrpab Arnt2 Maml1 Trp53 Foxp3 Nufip1 Nup62 Myst4 Stat3 Eny2 Vegfa Ddx5 Irf4 Nfat5 Mkl1 ENSMUSG00000072684 Ets1 |
| cellular chemical homeostasis | GO:0055082 | 1.000 | Socs6 ENSMUSG00000075466 Atp2a2 Fyn Serinc5 Ptprc Pex2 Lck Htt Psen1 Trp53 Ppp3ca Dep1 Trpc2 Clstn1 |
| transmembrane receptor protein tyrosine kinase signaling pathway | GO:0007169 | 1.000 | Stap1 Egf Stat3 Tiam1 Ptk2 Igf1r Rapgef1 Myo1e Phip Vegfa Tiparp |
| positive regulation of gene expression | GO:0010628 | 1.000 | Foxm1 Zfp326 Arid2 Rb1 Clock Hmgb2 Runx1 Plagl1 Trim28 Lef1 Hnrpab Arnt2 Maml1 Trp53 Foxp3 Nufip1 Nup62 Myst4 Stat3 Eny2 Vegfa Ddx5 Irf4 Nfat5 Mkl1 ENSMUSG00000072684 Ets1 |
| cation transport | GO:0006812 | 1.000 | Slc36a1 Atp6v1a Slc9a9 Vps4b Nfatc1 Ptprc Lck Itpr3 Slc20a2 Ppp3ca Slc9a1 Slc38a9 Slc23a2 Slc39a14 Slc4a8 Atp1b3 Slc23a1 Atp6v0b Atp2a2 Atp5b Slc30a4 Psen1 Atp5g3 Atp6v0d2 Tpcn2 D2Ertd127e Trpc2 Atp13a1 |
| cell motion | GO:0006928 | 1.000 | Gipc1 ENSMUSG00000075466 Ptk2 Mycbp2 Vegfa Fyn Tes Atp5b Pex2 1110034A24Rik Itgb1 Psen1 Myh9 Strbp Ulk1 Top2b Nup62 Rpl24 B3gnt2 Macf1 |
| muscle organ development | GO:0007517 | 1.000 | Sin3b Tpm1 Itgb1 Homer1 Eif4e3 Ppp3ca D3Ucla1 Dicer1 Col11a1 Tiparp |
| membrane organization | GO:0016044 | 1.000 | Ldlrap1 Fnbp1 Pacsin1 Pacsin2 Ap1s3 Abca7 Nme1 Dysf Eps15l1 Htt 8030493G06Rik Timm13 Trp53 Ulk1 Pldn |
| positive regulation of RNA metabolic process | GO:0051254 | 1.000 | Stat3 Foxm1 Rb1 Arid2 Eny2 Clock Hmgb2 Vegfa Runx1 Plagl1 Trim28 Irf4 Hnrpab Nfat5 Lef1 Hnrpll Maml1 Arnt2 Foxp3 Trp53 ENSMUSG00000072684 Nufip1 Ets1 |
| secretion | GO:0046903 | 1.000 | Gipc1 Exoc2 Htt Psen1 Tmed10 Txlna Arhgap17 Trpc2 Gars Lcp2 Erp29 Pldn |
| cellular ion homeostasis | GO:0006873 | 1.000 | ENSMUSG00000075466 Atp2a2 Fyn Serinc5 Ptprc Pex2 Lck Htt Psen1 Trp53 Ppp3ca Dep1 Clstn1 Trpc2 |
| positive regulation of catalytic activity | GO:0043085 | 1.000 | Csf1 Dgkz Spag9 Psenen Npm1 Gspt1 Serinc5 Ptprc Lck Egf Psen1 Cd74 Trp53 Nrk |
| cell motility | GO:0048870 | 1.000 | Gipc1 ENSMUSG00000075466 Ptk2 Vegfa Fyn Tes Atp5b Pex2 1110034A24Rik Itgb1 Psen1 Myh9 Ulk1 Top2b Nup62 |
| localization of cell | GO:0051674 | 1.000 | Gipc1 ENSMUSG00000075466 Ptk2 Vegfa Fyn Tes Atp5b Pex2 1110034A24Rik Itgb1 Psen1 Myh9 Ulk1 Top2b Nup62 |
| monovalent inorganic cation transport | GO:0015672 | 1.000 | Slc23a2 Slc36a1 Slc38a9 Atp6v1a Slc9a9 Slc4a8 Atp1b3 Slc23a1 Vps4b Atp6v0b Atp5b Atp5g3 Atp6v0d2 Slc20a2 D2Ertd127e Slc9a1 |
| endocytosis | GO:0006897 | 1.000 | Eps15l1 8030493G06Rik Ldlrap1 Fnbp1 Ulk1 Pacsin1 Pacsin2 Ap1s3 Abca7 Nme1 |
| membrane invagination | GO:0010324 | 1.000 | Eps15l1 8030493G06Rik Ldlrap1 Fnbp1 Ulk1 Pacsin1 Pacsin2 Ap1s3 Abca7 Nme1 |
| positive regulation of transcription from RNA polymerase II promoter | GO:0045944 | 1.000 | Stat3 Foxm1 Rb1 Arid2 Clock Hmgb2 Vegfa Runx1 Plagl1 Irf4 Nfat5 Lef1 Arnt2 Maml1 Foxp3 Trp53 ENSMUSG00000072684 Nufip1 Ets1 |
| positive regulation of transcription, DNA-dependent | GO:0045893 | 1.000 | Stat3 Foxm1 Rb1 Arid2 Eny2 Clock Hmgb2 Vegfa Runx1 Plagl1 Trim28 Irf4 Hnrpab Nfat5 Lef1 Maml1 Arnt2 Foxp3 Trp53 ENSMUSG00000072684 Nufip1 Ets1 |
| neuron development | GO:0048666 | 1.000 | Ptk2 Mycbp2 Tbce Fig4 Vegfa Runx1 Htt Rgnef Psen1 Ss18l1 Ulk1 Top2b Rpl24 B3gnt2 Cep290 |
| cell part morphogenesis | GO:0032990 | 1.000 | Rgnef Ptk2 Col4a3bp Mycbp2 Tbce Ssbp1 Ulk1 Top2b Rpl24 Pcnt B3gnt2 |
| male gamete generation | GO:0048232 | 1.000 | B4galnt1 Ddx25 Siah1a Herpud2 Hmgb2 Brca2 Htt Hnrpll Hrb Xrn2 Strbp Hsf1 Dep1 |
| spermatogenesis | GO:0007283 | 1.000 | B4galnt1 Ddx25 Siah1a Herpud2 Hmgb2 Brca2 Htt Hnrpll Hrb Xrn2 Strbp Hsf1 Dep1 |
| neuron differentiation | GO:0030182 | 1.000 | Stat3 Ptk2 Mycbp2 Tbce Ppia Fig4 Rbpj Vegfa Runx1 Cux1 Htt Rgnef Psen1 Ss18l1 Jag1 Ulk1 Top2b Rpl24 B3gnt2 Cep290 |
| cell projection organization | GO:0030030 | 1.000 | Lpin1 Ptk2 Wasl Rdx Mycbp2 Tbce Pcnt Rgnef Ss18l1 Myh9 Ttc8 Ulk1 Top2b Rpl24 B3gnt2 Cep290 |
| defense response | GO:0006952 | 1.000 | Stat3 Cd44 Bnip3l Il18r1 Rbpj Tlr9 Tnfrsf1b Ptprc Cnpy3 Polr3h Malt1 Tapbp Mif Ddx58 Cd74 B2m Il27ra Skiv2l Samhd1 H2 Polr3d Nfkbid |
| reproductive developmental process | GO:0003006 | 1.000 | Csf1 Cd44 Ddx25 Vegfa Rps6kb1 Hmgb2 Brca2 Hrb Igf1r Fancc Strbp Fanca Tcf7 |
| regulation of system process | GO:0044057 | 1.000 | Gipc1 Tpm1 ENSMUSG00000075466 Htt Psen1 Hrc Kras Pink1 Ppp3ca Atp2a2 |
| ion transport | GO:0006811 | 1.000 | Slc36a1 Atp6v1a Slc9a9 Vps4b Nfatc1 Fxyd5 Tes Ptprc Lck Htt Itpr3 Slc4a2 Slc20a2 Ppp3ca Slc9a1 Slc23a2 Slc38a9 Slc39a14 Slc4a8 Ank Atp1b3 Slc23a1 Atp6v0b Atp2a2 Atp5b Slc30a4 Lasp1 Psen1 Atp5g3 Atp6v0d2 Tpcn2 Slc25a4 D2Ertd127e Trpc2 Atp13a1 Clcn4 |
| transmission of nerve impulse | GO:0019226 | 1.000 | Gipc1 Psen1 Ptk2 Slc25a4 Ppp3ca Camk4 Clstn1 Dep1 Tes Fyn Serinc5 |
| epithelium development | GO:0060429 | 1.000 | Csf1 Plxnd1 Cd44 Xbp1 Vegfa Rbpj Htt Sufu Igf1r Sharpin Jag1 Fras1 Ptch1 |
| enzyme linked receptor protein signaling pathway | GO:0007167 | 1.000 | Stap1 Stat3 Ptk2 Rapgef1 Spnb2 Vegfa Egf Tiam1 Igf1r Trp53 Myo1e Phip Tiparp |
| metal ion transport | GO:0030001 | 1.000 | Slc23a2 Slc38a9 Slc39a14 Slc9a9 Slc4a8 Atp1b3 Slc23a1 Vps4b Atp2a2 Nfatc1 Ptprc Slc30a4 Lck Itpr3 Slc20a2 Tpcn2 Ppp3ca D2Ertd127e Trpc2 Slc9a1 |
| regulation of RNA metabolic process | GO:0051252 | 1.000 | Zfp472 Phf21a Rb1 Eif4e3 Sap18 Clock Nfatc1 Zfp59 Runx1 Tnfrsf1b Plagl1 Yeats4 Ikbkap Tshz2 Sin3b Lef1 Sufu Trp53 Pa2g4 Nfe2l3 Hsf1 Nufip1 Stat3 Zfp212 Ddx20 Vegfa Rbpj RP23 Zfp655 Zfp27 Rfx3 Nfat5 Atf5 Ss18l1 Zfp677 Med4 Ets1 Foxm1 Tle4 Arid2 5830417I10Rik Xbp1 Bach2 Hmgb2 Hivep1 Snrp70 Cux1 Maff Trim28 Hnrpab Hnrpll Maml1 Arnt2 Tbl1x Pde8a Foxp3 Dpm1 Per1 Med20 Cbx1 Dnmt3b 9130019O22Rik Eny2 Npm1 Zfp58 Stat1 Irf4 Rbbp7 Sin3a Irf5 ENSMUSG00000072684 Tcf7 Pdcd11 |
| pattern specification process | GO:0007389 | 1.000 | Rfx3 Lef1 Sufu Htt Sf3b1 Psen1 Plxnd1 Trp53 Rnf111 Vegfa Atm Ptch1 |
| regulation of transcription, DNA-dependent | GO:0006355 | 1.000 | Zfp472 Phf21a Rb1 Eif4e3 Sap18 Clock Nfatc1 Zfp59 Runx1 Plagl1 Yeats4 Ikbkap Tshz2 Sin3b Lef1 Sufu Trp53 Pa2g4 Nfe2l3 Hsf1 Nufip1 Stat3 Zfp212 Ddx20 Vegfa Rbpj RP23 Zfp655 Rfx3 Zfp27 Nfat5 Atf5 Ss18l1 Zfp677 Med4 Ets1 Foxm1 Tle4 Arid2 5830417I10Rik Xbp1 Bach2 Hmgb2 Hivep1 Cux1 Trim28 Maff Hnrpab Maml1 Arnt2 Tbl1x Foxp3 Pde8a Dpm1 Per1 Med20 Cbx1 Dnmt3b 9130019O22Rik Eny2 Zfp58 Stat1 Irf4 Rbbp7 Sin3a Irf5 ENSMUSG00000072684 Tcf7 Pdcd11 |
| embryonic morphogenesis | GO:0048598 | 1.000 | Lnp Dicer1 Col11a1 Htt Lef1 Sufu Psen1 Trp53 D3Ertd300e Txnrd1 Tcf7 Ptch1 Cep290 Macf1 |
| behavior | GO:0007610 | 1.000 | ENSMUSG00000075466 Stat3 Aprt Phf21a Tbce Prkce Fig4 Runx1 Htt Psen1 Kras Strbp Trpc2 Cxcl16 Asl |
| response to wounding | GO:0009611 | 1.000 | Dysf Mif Stat3 Cd44 Pros1 Lnp Tlr9 Hps5 Tnfrsf1b Nfkbid Pldn |
| oxidation reduction | GO:0055114 | 1.000 | Ndufs3 Fbxl10 Rdh11 Ndufs6 Pdhb Foxred2 Pdha1 Cyp4v3 Bdh2 Hnrpll Dhrs7 Jmjd2b Aof1 P4ha1 Ogdh Nxn Nphp3 Ndufv2 Dhrs7b Gsr Jarid1c Txnrd1 Fdx1l Prdx3 |
| biological adhesion | GO:0022610 | 1.000 | Rapgef1 Cd44 Parvg Ptprc Zyx Col11a1 Col5a1 Cyfip2 Cd97 Siglec5 Itgb1 Lef1 Psen1 Cd2 Myh9 Adam8 Clstn1 |
| cell adhesion | GO:0007155 | 1.000 | Rapgef1 Cd44 Parvg Ptprc Zyx Col11a1 Col5a1 Cyfip2 Cd97 Siglec5 Itgb1 Lef1 Psen1 Cd2 Myh9 Adam8 Clstn1 |
| cognition | GO:0050890 | 1.000 | Htt Psen1 Itpr3 Kras Wdr1 Diap1 Iqcb1 Fyn Gtf3c2 B3gnt2 |
| neurological system process | GO:0050877 | 1.000 | Gipc1 Ptk2 Fyn Tes Serinc5 Tpp1 Htt Psen1 Itpr3 Kras Wdr1 Slc25a4 Diap1 Ppp3ca Camk4 Dep1 Iqcb1 Clstn1 Gtf3c2 B3gnt2 |
| cell surface receptor linked signal transduction | GO:0007166 | 1.000 | Spnb2 Tnfrsf1b Ptprc Lef1 Igf1r Gabbr1 Trp53 Cd247 Dgkz Gipc1 Stat3 Psenen Rapgef1 Homer1 Mib2 Vegfa Rbpj Egf Itgb1 Tiam1 Psen1 Nxn Myo1e Ptch1 Tiparp Stap1 Ptk2 Tle4 Fyn Cd97 Lck Htt Maml1 Csnk1d Adam8 Foxp3 Nup62 Hax1 Adam10 Zap70 Tax1bp3 Stat1 Malt1 Jag1 Phip Rgs3 Tcf7 Lag3 Macf1 |

**Biological processes of the 140 transcripts differentially expressed between TCR-activated T cells and TCR/CD28-activated T cells**

| **Gene Ontology Biological Process** | **GO ID** | **p-value of enrichment compared to whole genome** | **Gene symbols** |
| --- | --- | --- | --- |
| DNA metabolic process | GO:0006259 | 0.091 | Ccne1 Exo1 Pola1 Mcm10 Hells Dna2 Mcm8 Cd40lg Chaf1a Dtl Ung Orc1l |
| cell cycle | GO:0007049 | 0.957 | Ccne1 Exo1 Pola1 Hells Jub Ccng2 Trp53inp1 Mcm8 Cks1b Myb Chaf1a |
